# Amplicon sequencing and culture-dependent approaches reveal core bacterial endophytes aiding freezing stress tolerance in alpine Rosaceae plants

**DOI:** 10.1128/mbio.01418-24

**Published:** 2025-02-25

**Authors:** Malek Marian, Livio Antonielli, Ilaria Pertot, Michele Perazzolli

**Affiliations:** 1 Center Agriculture Food Environment (C3A), University of Trento, San Michele all'Adige, Italy; 2 Center for Health & Bioresources, Bioresources Unit, AIT Austrian Institute of Technology, Tulln, Austria; 3 Research and Innovation Centre, Fondazione Edmund Mach, San Michele all'Adige, Italy; University of California, Irvine, California, USA; Politechnika Poznanska, Poznan, Poland

**Keywords:** plant microbiome, core endophytic bacteria, psychrotolerant bacterial endophytes, alpine plants, freezing stress, Rosaceae plants, spring frost, cold adapted bacteria

## Abstract

Wild plants growing in alpine regions are associated with endophytic microbial communities that may support plant growth and survival under cold conditions. The structure and function of endophytic bacterial communities were characterized in flowers, leaves, and roots of three alpine Rosaceae plants in Alpine areas using a combined amplicon sequencing and culture-dependent approaches to determine the role of core taxa on plant freezing stress tolerance. Amplicon sequencing analysis revealed that plant tissue, collection site, and host plant are the main factors affecting the richness, diversity, and taxonomic structure of endophytic bacterial communities in alpine Rosaceae plants. Core endophytic bacterial taxa were identified as 31 amplicon sequence variants highly prevalent across all plant tissues. Psychrotolerant bacterial endophytes belonging to the core taxa of *Duganella*, *Erwinia*, *Pseudomonas*, and *Rhizobium* genera mitigated freezing stress in strawberry plants, demonstrating the beneficial role of endophytic bacterial communities and their potential use for cold stress mitigation in agriculture.

**IMPORTANCE:**

Freezing stress is one of the major abiotic stresses affecting fruit production in Rosaceae crops. Current strategies to reduce freezing damage include physical and chemical methods, which have several limitations in terms of costs, efficacy, feasibility, and environmental impacts. The use or manipulation of plant-associated microbial communities was proposed as a promising sustainable approach to alleviate cold stress in crops, but no information is available on the possible mitigation of freezing stress in Rosaceae plants. A combination of amplicon sequencing, culture-dependent, and plant bioassay approaches revealed the beneficial role of the endophytic bacterial communities in alpine Rosaceae plants. In particular, we showed that culturable psychrotolerant bacterial endophytes belonging to the core taxa of *Duganella*, *Erwinia*, *Pseudomonas*, and *Rhizobium* genera can mitigate freezing stress on strawberry seedlings. Overall, this study demonstrates the potential use of psychrotolerant bacterial endophytes for the development of biostimulants for cold stress mitigation in agriculture.

## INTRODUCTION

Fruit crops belonging to the Rosaceae family, such as apple, cherry, pear, and strawberry, are of relevant economic and agricultural value in temperate regions (1). Unfortunately, freezing stress is one of the major abiotic stresses affecting fruit production in Rosaceae crops, causing damage to flower and leaf tissues in the spring period (2, 3). On average, spring frost occurs at least once every 3 to 5 years and causes severe losses in many temperate regions in the European Union, United States, and China (4–6). Furthermore, global warming due to climate change will likely promote early spring-related phenological events in plants with a consequent increased probability of incurring severe spring frosts (6–8).

Current strategies implemented by growers to reduce freezing damage consist of physical methods (e.g., overhead irrigation, heaters, and wind machines) and chemical treatments (e.g., growth regulators, vitamins, and bactericides) (9, 10). However, these approaches have several limitations in terms of costs, efficacy, feasibility, and environmental impacts (11), indicating that sustainable mitigation strategies are required.

Plants are associated with highly diverse, complex, and dynamic microbial communities, which are hosted on (i.e., epiphytic) and inside (i.e., endophytic) plant tissues and can support plant growth under stress conditions (12). In particular, endophytic bacterial communities have intimate interactions and extensive exchange of metabolites with their plant host (13). Plant-associated microbial communities have complex and diverse structures, and identifying which members are functionally important for the host plant is still a key challenge in plant microbiome research. A small subset of plant-associated microbial communities constitutes the core microbial taxa and consists of a group of members present in almost all the communities associated with a given host plant across a wide range of environments with potential beneficial traits for the plant host (14). For example, core microbial taxa play important roles in promoting plant growth and health by fixing nitrogen (15) and/or suppressing plant diseases (16). The core microbial taxa may also include the so-called “hub taxa” which can be defined as highly connected nodes in co-occurrence network analysis (17, 18). It has also been shown that such hub taxa could be keystone taxa affecting microbial community assembly (19). Keystone taxa can further drive microbial community structure and support functions; for example, they can support soil microbial-mediated functions (e.g., nutrient cycling, organic matter decomposition, and gas emissions) in the presence of a loss of microbial biodiversity (20) or mediate microbial extracellular enzyme degradation and assimilation (21). Therefore, the identification of hub taxa and functional keystone taxa in the core microbiota of plants could be the first step to identify the key microbial community members to be further manipulated to improve plant stress tolerance. Recently, the use or manipulation of plant-associated microbial communities was proposed as a promising sustainable approach to alleviate cold stress in crops (22–24), but no information is available on the possible mitigation of freezing stress in Rosaceae plants.

Wild plants growing in alpine regions are associated with cold-adapted, highly complex, and host-specific microbial communities, which may promote plant growth and survival under cold conditions (25). For example, psychrotolerant (i.e., cold adapted) *Pseudomonas* spp. isolated from the leaf apoplast of five mountain plant species (*Colchicum speciousum*, *Draba nemorosa*, *Erodium cicutarium*, *Galanthus gracilis*, and *Scilla siberica*) improved bean (*Phaseolus vulgaris* L.) tolerance to freezing stress (26). Likewise, *Paraburkholderia phytofirmans* PsJN can mitigate freezing damage in *Arabidopsis thaliana* possibly by inducing cell wall strengthening (27). However, no information is currently available on the structure and function of endophytic bacterial communities associated with alpine Rosaceae plants (particularly reproductive organs, such as flowers) and factors that shape the community structure. The aim of this study was to demonstrate the function of core endophytic bacterial taxa of alpine Rosaceae plants on the mitigation of freezing stress in plants.

## RESULTS

### Plant tissue, collection site, and alpine Rosaceae plant are the main factors affecting the richness, diversity, and taxonomic structure of endophytic bacterial communities

**Fig 1:**
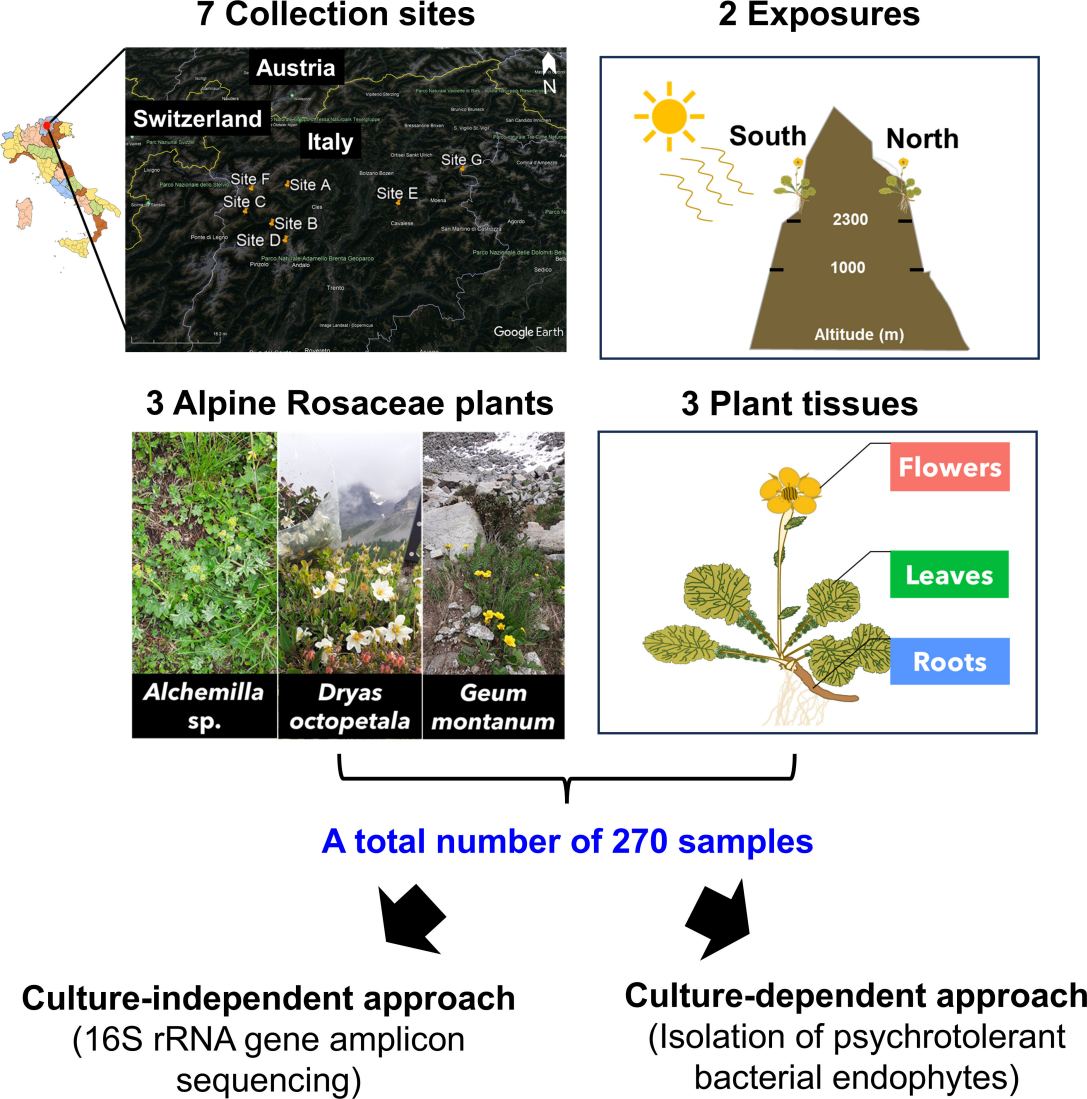
Experimental design of the study. A total number of 270 samples were analyzed through culture-independent and culture-dependent approach. Samples were obtained from three alpine Rosaceae plants (*Alchemilla* sp., *Dryas octopetala*, and *Geum montanum*) and three plant tissues (flowers, leaves, and roots) collected from seven sites (Val di Non, Val di Sole, Val di Pejo, Val Rendena, South Tyrol, Stelvio Park, and Val di Fassa, hereafter named Site A, B, C, D, E, F, and G, respectively) in Alpine areas of the Trentino-alto Adige Region (Italy) and two exposures (North and South). This map includes data from Google and Landsat / Copernicus. Imagery from the dates:1 January 2021.

The 16S rRNA amplicon sequencing analysis of the endophytic bacterial communities associated with alpine Rosaceae plants (270 samples; Fig. 1; Tables S1 and S2) produced a total of 5,888 amplicon sequence variants (ASVs) (13,074,650 filtered read counts; Table S3) with rarefaction curves that reached richness (observed ASVs) saturation (Fig. S1). Additionally, a total of 152 ASVs were defined as the most abundant ASVs having a mean relative abundance of >0.1% (Table S3).

**Fig 2:**
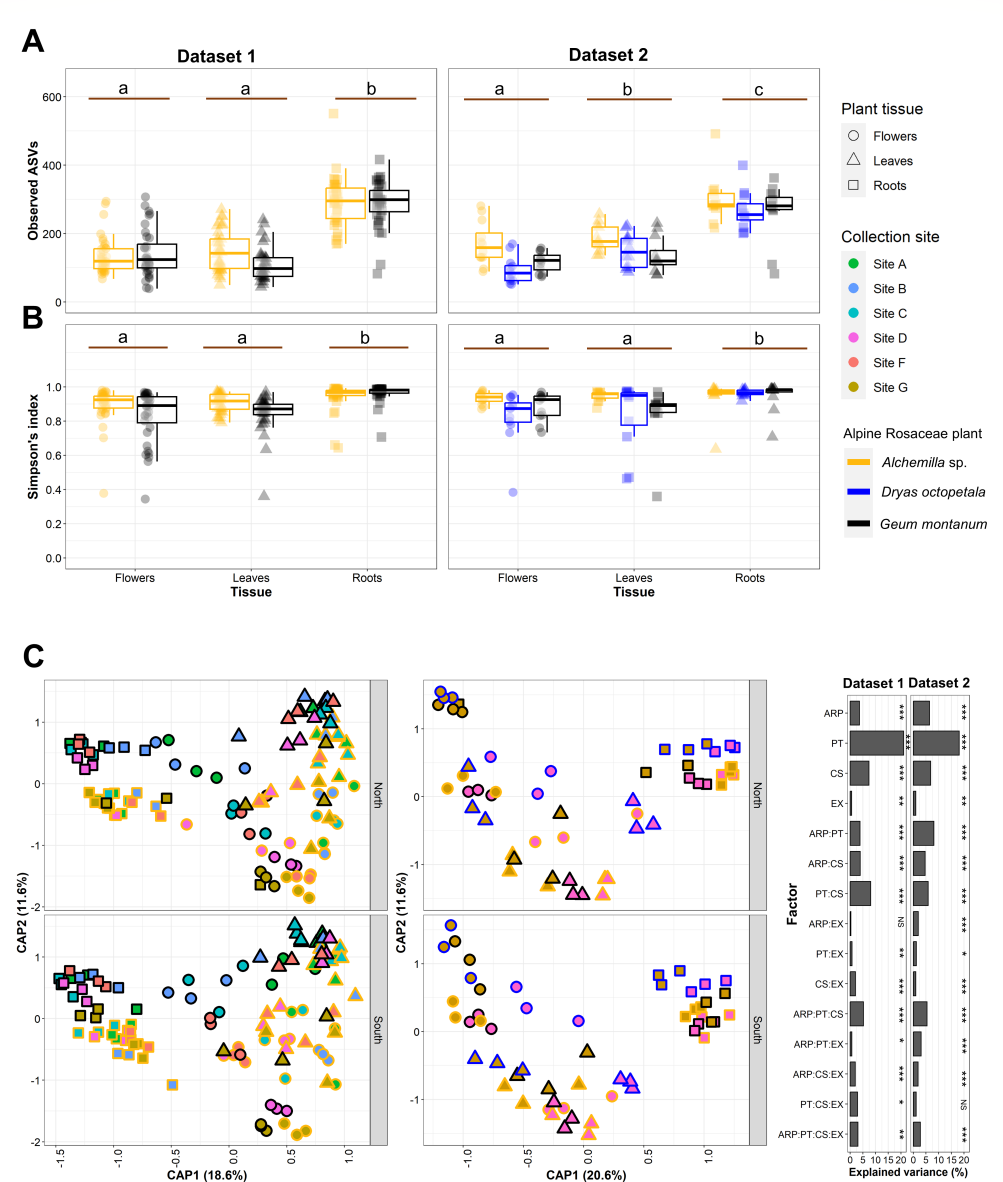
Diversity of endophytic bacterial communities associated with alpine Rosaceae plants. (**A**) Observed amplicon sequence variants (ASVs) (richness) and (**B**) Simpson’s index (alpha-diversity) are reported for the first dataset (dataset 1: *Alchemilla* sp. and *Geum montanum* from six collection sites) and the second dataset (dataset 2: *Alchemilla* sp., *D. octopetala*, and *G. montanum* from two collection sites) of samples. Lowercase letters indicate results of estimated marginal mean comparisons between tissues (Tables S4 and S5). (**C**) Constrained analysis of principle (CAP) coordinates based on Bray–Curtis dissimilarity matrices on samples collected from flower, leaf, and root tissues of alpine Rosaceae plants from different collection sites and exposure in datasets 1 and 2 . The percentage of explained variance of each factor (abbreviations: ARP, alpine Rosaceae plants; PT, plant tissue; CS, collection site; EX, exposure) and their interactions is shown on the right for datasets 1 and 2, calculated based on permutational multivariate analysis of variances on Bray–Curtis dissimilarities (***P < 0.001, **P < 0.01, *P < 0.05, NS = *P* > .05).

Bacterial richness and alpha-diversity (Simpson’s index) partially decreased from roots (average of 526.41 and 0.96) to leaves (average of 191.03 and 0.88) and to flowers (average of 179.24 and 0.86; Table S2). After splitting the data into two datasets (i.e., datasets 1 and 2), richness differed according to plant tissue (*F* = 290.76, *P* < 0.001) and collection site (*F* = 8.66, *P* < 0.001) in dataset 1 (*Alchemilla* sp. and *Geum montanum* from six collection sites), as well as according to alpine Rosaceae plants (*F* = 10.00, *P* < 0.001), plant tissue (*F* = 83.33, *P* < 0.001), and collection site (*F* = 12.79, *P* < 0.001) in dataset 2 (*Alchemilla* sp., *D. octopetala*, and *G. montanum* from two collection sites) according to the linear models (Table S4). Alpha-diversity in datasets 1 and 2 was influenced by plant tissue (*F* = 18.79, *P* < 0.001 and *F* = 5.04, *P* < 0.01, respectively), alpine Rosaceae plants (*F* = 6.18, *P* < 0.05 and *F* = 3.26, *P* < 0.05, respectively), and exposure (*F* = 6.71, *P* < 0.05 and *F* = 4.25, *P* < 0.05, respectively; Table S5). The collection site had also significant effect on alpha-diversity in dataset 1 (*F* = 3.19, *P* < 0.01). In particular, richness and alpha-diversity were higher in roots than in flowers and leaves according to estimated marginal mean comparisons in both datasets (Fig. 2A and B; Tables S4 and S5). Thus, plant tissue was also the main factor shaping richness and alpha-diversity in the conditional inference regression tree analysis (Fig. S2) and endophytic bacterial communities clustered according to plant tissue and alpine Rosaceae plant in the hierarchical clustering analysis (Fig. S3).

Constrained analysis of principal coordinates (CAP) ordinations based on Bray–Curtis dissimilarities demonstrated the differentiation of endophytic bacterial community structure according to plant tissue and alpine Rosaceae plant in both datasets (Fig. 2C). CAP permutation tests were used to assess the significance of constraints and showed that all factors significantly affected the beta-diversity in both datasets (Table S6). In particular, the taxonomic structure of endophytic bacterial communities was mainly affected by plant tissue (*F* = 44.45; coefficient of determination (R^2^) = 0.21; *P* < 0.001 and *F* = 21.28, R^2^ = 0.18, *P* < 0.001), collection site (*F* = 6.26, R^2^ = 0.07, *P* < 0.001 and *F* = 16.18, R^2^ = 0.07, *P* < 0.001), and alpine Rosaceae plants (*F* = 15.49, R^2^ = 0.04, *P* < 0.001 and *F* = 7.52, R^2^ = 0.06, *P* < 0.001), with a minor contribution of exposure (*F* = 2.40, R^2^ = 0.01, *P* < 0.01 and *F* = 2.28, R^2^ = 0.07, *P* < 0.01) in datasets 1 and 2, respectively, according to beta-diversity analysis carried out with permutational multivariate analysis of variances (PERMANOVA) on Bray–Curtis dissimilarities (Fig. 2C; Table S7). A dispersion in the ASV data was found, particularly within plant tissues (*F* = 48.80, *P* = <2.20 × 10^16^ and *F* = 5.12, *P* = 0.008 for datasets 1 and 2, respectively), according to permutational multivariate analysis of dispersion (PERMDISP2; Table S7), suggesting that differences observed by PERMANOVA may be attributed to either centroid or dispersion. The multivariate generalized linear models and model-based ordination on ASVs supported the results obtained with PERMANOVA, and the variation in the taxonomic structure of endophytic bacterial communities was mainly explained by plant tissue, followed by collection site, alpine Rosaceae plant, and exposure in both datasets (Fig. S4; Table S8).

Within each plant tissue, endophytic bacterial communities differed according to alpine Rosaceae plant, collection site, and exposure (Fig. S5; Table S9). In particular, the host−environment effects index (HEEI) values revealed that alpine Rosaceae plant influenced mainly root-associated communities (1.25 and 2.81 HEEI), whereas collection site influenced mainly leaf-associated communities (0.37 and 1.48 HEEI) and flower-associated communities (0.20 and 0.53 HEEI) in datasets 1 and 2, respectively (Table S9).

### Core endophytic bacterial taxa of alpine Rosaceae plants are selected by the host plant, and the indicator taxa are enriched in flower and leaf tissues compared to roots

**Fig 3:**
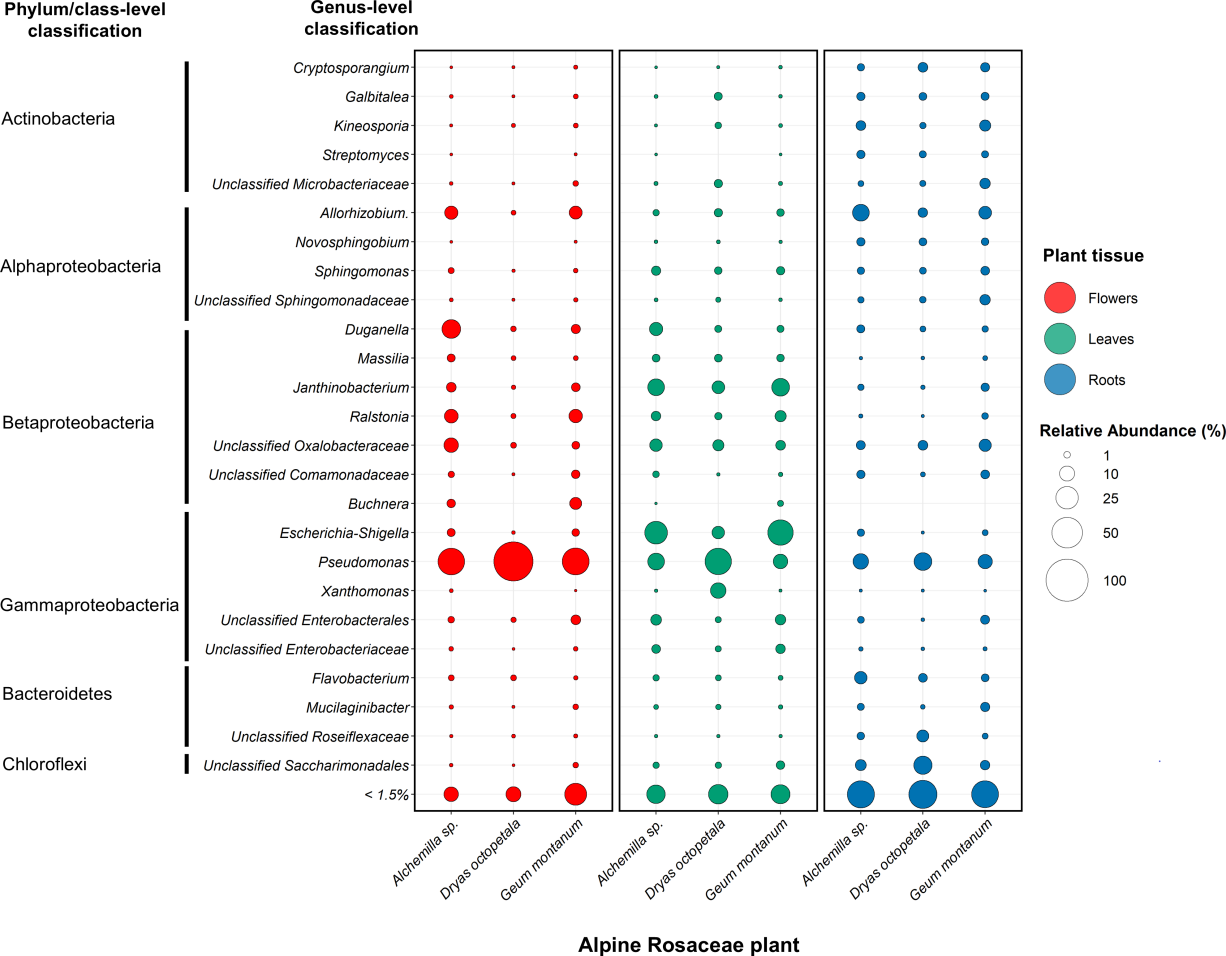
Taxonomic summary of the most abundant endophytic bacterial genera in alpine Rosaceae plants. Bubble plots represent the mean relative abundance of reads per taxon, after aggregating amplicon sequence variants (ASVs) by genus level in each alpine Rosaceae plant and tissue across all samples, collection sites, and exposures. Taxa are coded with different colors according to the tissue. Only taxa with >1.5 mean relative abundance are shown.

Endophytic bacterial communities associated with Rosaceae plants were dominated by Proteobacteria (80.5%), followed by Actinobacteria (9.61%), Bacteroidota (4.07%), Patescibacteria (2.84%), Chloroflexi (1.53%), and Deinococcota (0.53%) phyla in terms of relative abundance (Fig. S6A and S7A). Most taxa detected were shared among the three alpine Rosaceae plants, with differences in relative abundance among the three plant tissues. In particular, flower-associated communities were dominated by *Pseudomonadaceae* (46.84%), *Oxalobacteriaceae* (16.03%), *Burkholderiaceae* (8.09%), and *Rhizobiaceae* (5.96%); leaf-associated communities were dominated by *Enterobacteriaceae* (27.27%), *Oxalobacteriaceae* (22.58%), *Pseudomonadaceae* (16.03%), and *Burkholderiaceae* (4.47%); and root-associated communities were dominated by *Rhizobiaceae* (11.48%), *Pseudomonadaceae* (10.54%), *Oxalobacteriaceae* (7.14%), and *Microbacteriaceae* (6.8%) (Fig. S6B and S7B). At the genus level, flower-associated communities were dominated by *Pseudomonas* (46.8%), *Duganella* (7.93%), *Ralstonia* (6.27%), *Allorhizobium–Neorhizobium–Pararhizobium–Rhizobium* (5.78%), and *Buchnera* (3.1%); leaf-associated communities were dominated by *Escherichia–Shigella* (24.88%), *Pseudomonas* (16.03%), *Janthinobacterium* (12.22%), *Duganella* (3.65%), *Ralstonia* (3.5%), and *Sphingomonas* (2.27%); and root-associated communities were dominated by *Pseudomonas* (10.54%), *Allorhizobium–Neorhizobium–Pararhizobium–Rhizobium* (8.4%), *Flavobacterium* (3.7%), *Kineosporia* (3.37%), *Cryptosporangium* (2.37%), and *Galbitalea* (1.91%) (Fig. 3; Fig. S7C).

**Fig 4:**
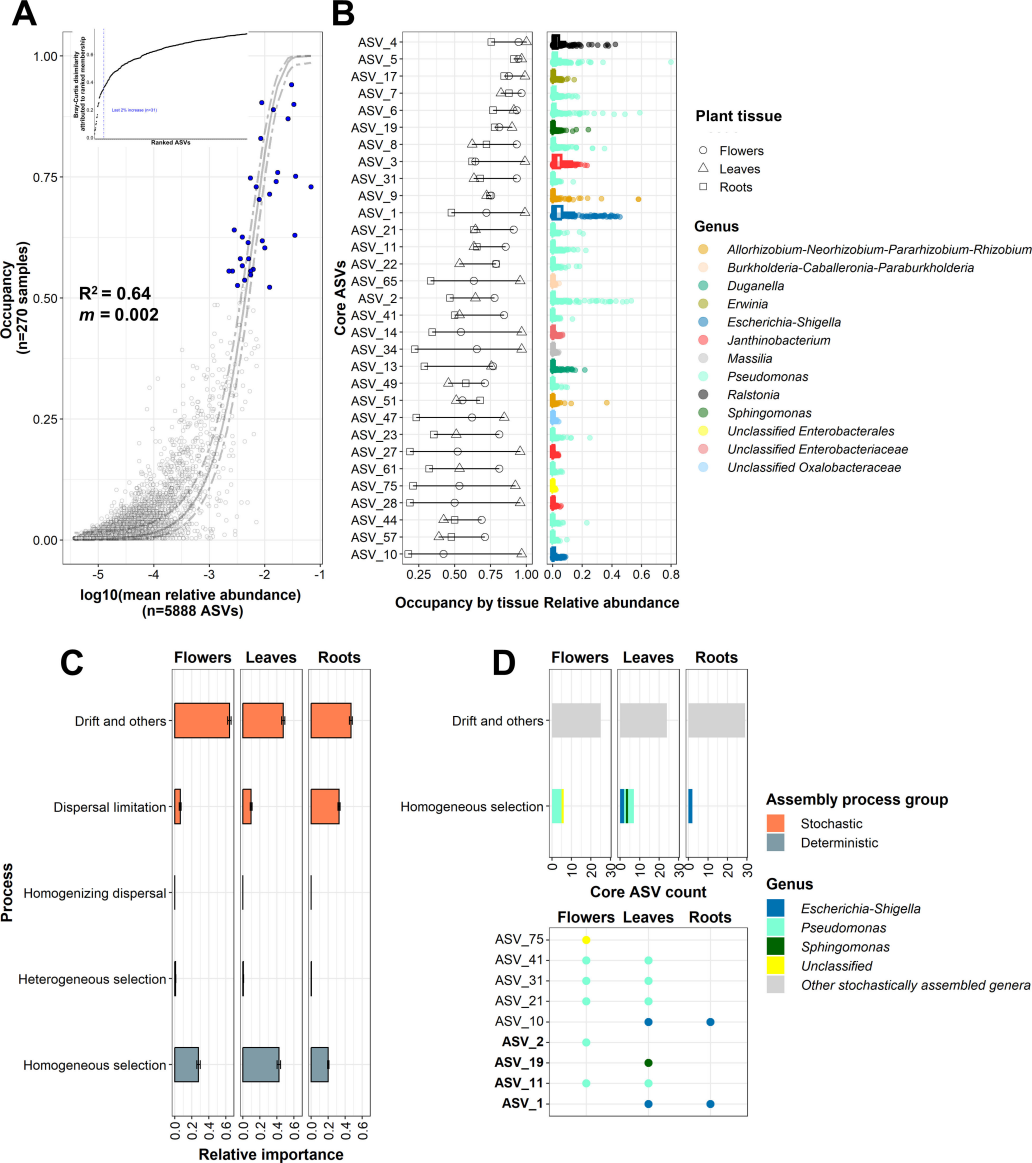
Abundance, occupancy, and structure of core bacterial taxa in alpine Rosaceae plants. (**A**) Abundance–occupancy distributions of amplicon sequence variants (ASVs) across flower, leaf, and, root tissue samples of *Alchemilla* sp., *D. octopetala*, and *G. montanum*. ASVs are represented by data points in grey and ASVs belonging to the core taxa are represented by blue dots. The solid line represents the fit of the Sloan neutral model, and the dashed line is the 95% confidence around the model prediction. R2 values (measurement of fit to neutral assembly process) and m-values (estimated migration rate) are indicated in the plot. The final 2% percent increase (represented by the blue dashed line) in beta-diversity based on Bray–Curtis dissimilarities due to the contribution of the prospective core set method used to identify core taxa is reported in the inset. The core taxa accounted for 24% of the total beta-diversity as shown in the inset. (**B**) Occupancy, relative abundance, and taxonomic annotation of ASVs of the core taxa are reported. The core taxa represented a small fraction (*n* = 31 ASVs) of the total community (*n* = 5,888 of total ASVs). (**C**) The relative importance of stochastic and deterministic processes in the community assembly of endophytic bacterial communities was calculated in flower, leaf, and root tissues. (**D**) The ASV count and taxonomic annotation at the genus level are reported for the core taxa assembled through homogeneous selection (i.e., selection under homogeneous abiotic and biotic environmental conditions leading to more similar structures among communities). Bold letters represent ASVs that were the top taxon within their respective phylogenetic bins.

Core endophytic bacterial taxa of alpine Rosaceae plants were composed of 31 ASVs highly prevalent across all plant tissues according to the abundance and occupancy distribution (Table S3). Although the core endophytic bacterial taxa represented a small fraction of the total community (0.53% of total ASVs), they accounted for 41% and 24% of the total relative abundance and beta-diversity, respectively, based on Bray–Curtis dissimilarities. Moreover, 12 ASVs of the core endophytic bacterial taxa were above the neutral prediction (ASVs selected by the host or with good dispersal capabilities), and 13 ASVs were below the predication (ASVs not selected by the host or with scarce dispersal capabilities) according to the Sloan neutral model prediction (95% confidence intervals; Fig. 4A).

The core endophytic bacterial taxa of alpine Rosaceae plants were composed of *Pseudomonas* (15 ASVs), *Janthinobacterium* (3 ASVs), *Escherichia–Shigella* (2 ASVs), *Allorhizobium–Neorhizobium–Pararhizobium–Rhizobium* (2 ASVs), *Burkholderia–Caballeronia–Paraburkholderia*, *Duganella*, *Erwinia*, *Massilia*, *Ralstonia*, and *Sphingomonas* genera, with one ASV each, and unclassified taxa (three ASVs; Fig. 4B). The assembly of the total endophytic bacterial communities was mainly driven by the stochastic processes of drift and dispersal limitation compared with the deterministic process of homogeneous selection (Fig. 4C), and the strong stochastic contribution was confirmed by pNST analysis (Fig. S8). Within each plant tissue, the assembly of core endophytic bacterial taxa was mainly driven by the drift process rather than homogeneous selection (Fig. 4D). However, some core endophytic bacterial taxa (ASV 1, 2, 10, 11) resulted under homogeneous selection (Fig. 4D) were the top taxa within their respective phylogenetic bins (relative abundance from 1 to 11% and relative importance from 0.02 to 23%; Table S10).

**Fig 5:**
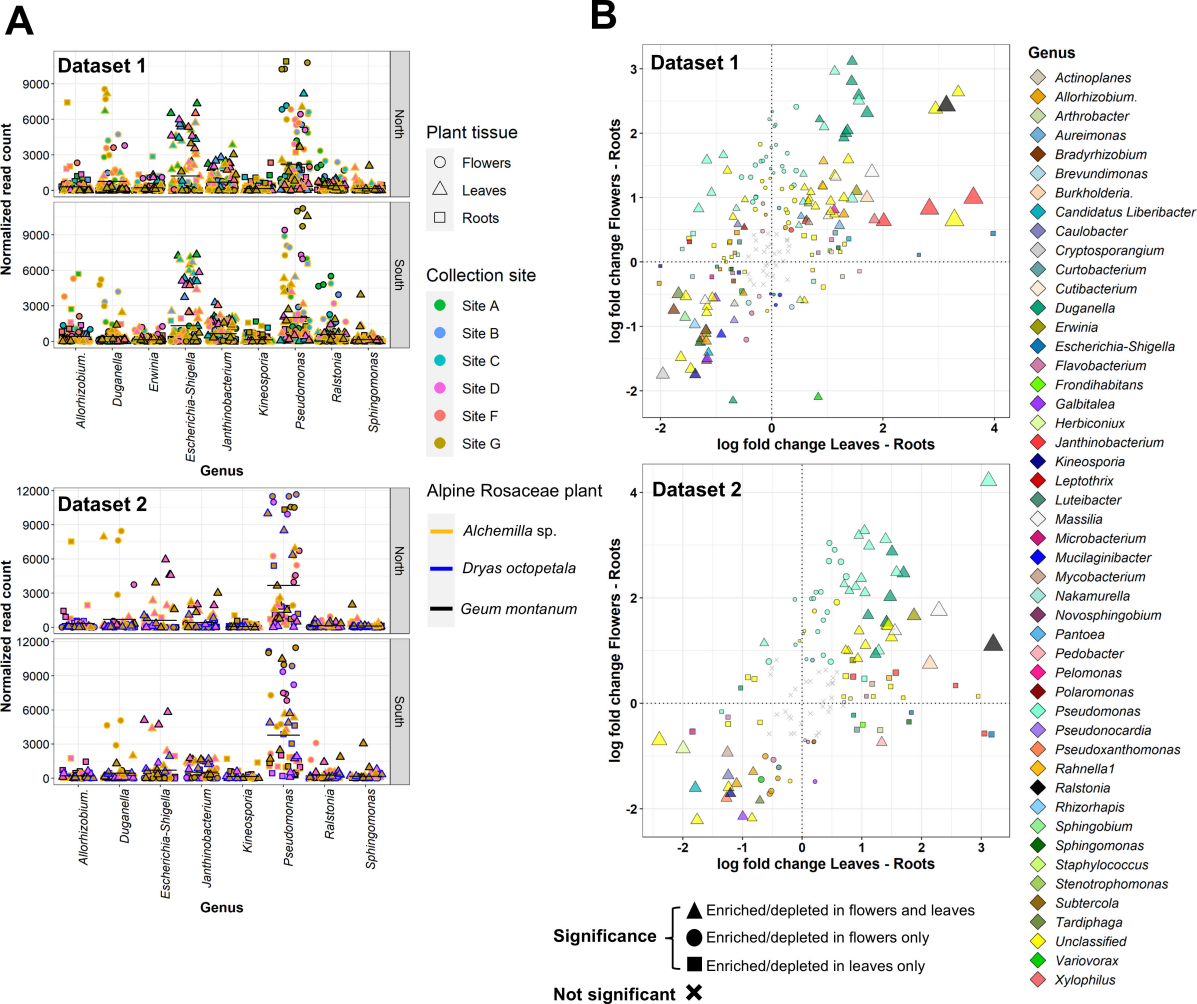
Identification of indicator and differentially abundant bacterial taxa enriched in different tissues of alpine Rosaceae plants. (**A**) A total of 331 and 296 amplicon sequence variants (ASVs) were identified as indicator ASVs by random forest models and were aggregated to 79 and 70 genera for the first dataset (*Alchemilla* sp. and *Geum montanum* from six collection sites) and the second dataset (*Alchemilla* sp., *D. octopetala*, and *G. montanum* from two collection sites), respectively. (**B**) Differentially abundant ASVs are presented as log fold changes obtained from the log-linear model in flowers and leaves compared with roots.

The indicator taxon analysis with random forest models revealed that indicator ASVs belonging to the *Pseudomonas*, *Escherichia–Shigella*, *Duganella*, *Ralstonia*, and *Janthinobacterium* genera were the most abundant indicator taxa (in terms of normalized read count), particularly in flower and leaf tissues rather than in root tissues (Fig. 5A; Table S11). Moreover, some indicator ASVs belonged to the core endophytic bacterial taxa, such as *Pseudomonas* (e.g., ASVs 2, 5, 6, 8, 11, 21, 61), *Duganella* (ASV 13), *Erwinia* (ASV 17), and *Ralstonia* (ASV 4). Differential abundance analysis confirmed that ASVs belonging to three (*Pseudomonas*, *Duganella*, and *Janthinobacterium*) and four (*Allorhizobium–Neorhizobium–Pararhizobium–Rhizobium*, *Caulobacter*, *Galbitalea,* and *Kineosporia*) genera were enriched and depleted in flower and leaf tissues compared with root tissues, respectively (Fig. 5B; Table S12).

### Core endophytic bacterial taxa of alpine Rosaceae plants are hub taxa in flowers and leaves

**Fig 6:**
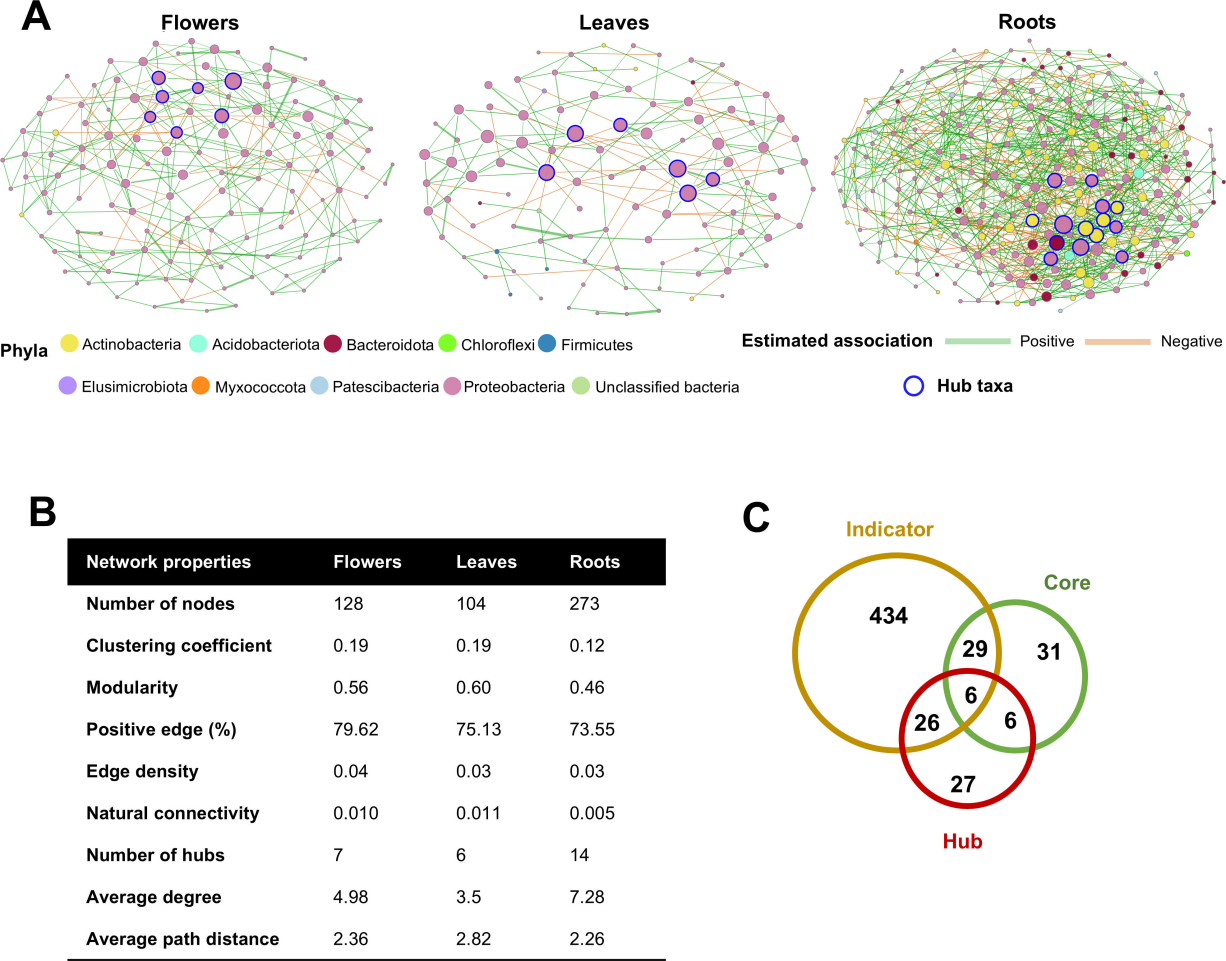
Endophytic bacterial co-occurrence networks of alpine Rosaceae plants. (**A**) Flowers, leaves, and roots networks. Each node corresponds to an amplicon sequence variant (ASV), and edges between nodes correspond to either positive (green) or negative (orange) correlations inferred from ASV abundance profiles. The thickness of each edge is proportional to the correlation coefficients of the connections. Node size reflects their eigenvector centrality, while nodes with blue outline color represent hub taxa. (**B**) Summary of network properties for each tissue (flowers, leaves, and roots). (**C**) Venn diagram showing the overlap in terms of number of ASVs between indicator, core, and hub taxa.

The co-occurrence network showed higher complexity and stronger connectivity in the roots than in the flowers and leaves (Fig. 6A; Table S13). In particular, higher number of hubs, higher average degree, and lower average path distance were detected in roots than in flowers and leaves (Fig. 6B). Venn diagram further showed that six ASVs were part of all three ASV groups (indicator, core, and hub taxa), while 6, 26, and 29 ASVs were part of at least two ASV groups (Fig. 6C). Comparative analysis of co-occurrence networks between similarity of most central nodes showed that flower and leaf networks were more similar (0.098–0.61 Jaccard index) compared with flower and root networks (0.0–0.194 Jaccard index) or leaf and root networks (0.0–0.267 Jaccard index) (Fig. S9; Table S13). Some ASVs of flower (ASVs 1 and 11) and leaf (ASVs 2, 4, 7, 10) hub taxa were also classified as core endophytic bacterial taxa, while root hub taxa comprised non-core taxa, such as *Sphingomonadaceae* (ASV 560, 280), *Microbacteriaceae* (ASV 200, 206), and *Sphingobacteriaceae* (ASV 314) (Fig. 6A; Table S13).

### Culturable bacterial endophytes belonging to the core endophytic bacterial taxa mitigate freezing stress in strawberry seedlings

**Fig 7:**
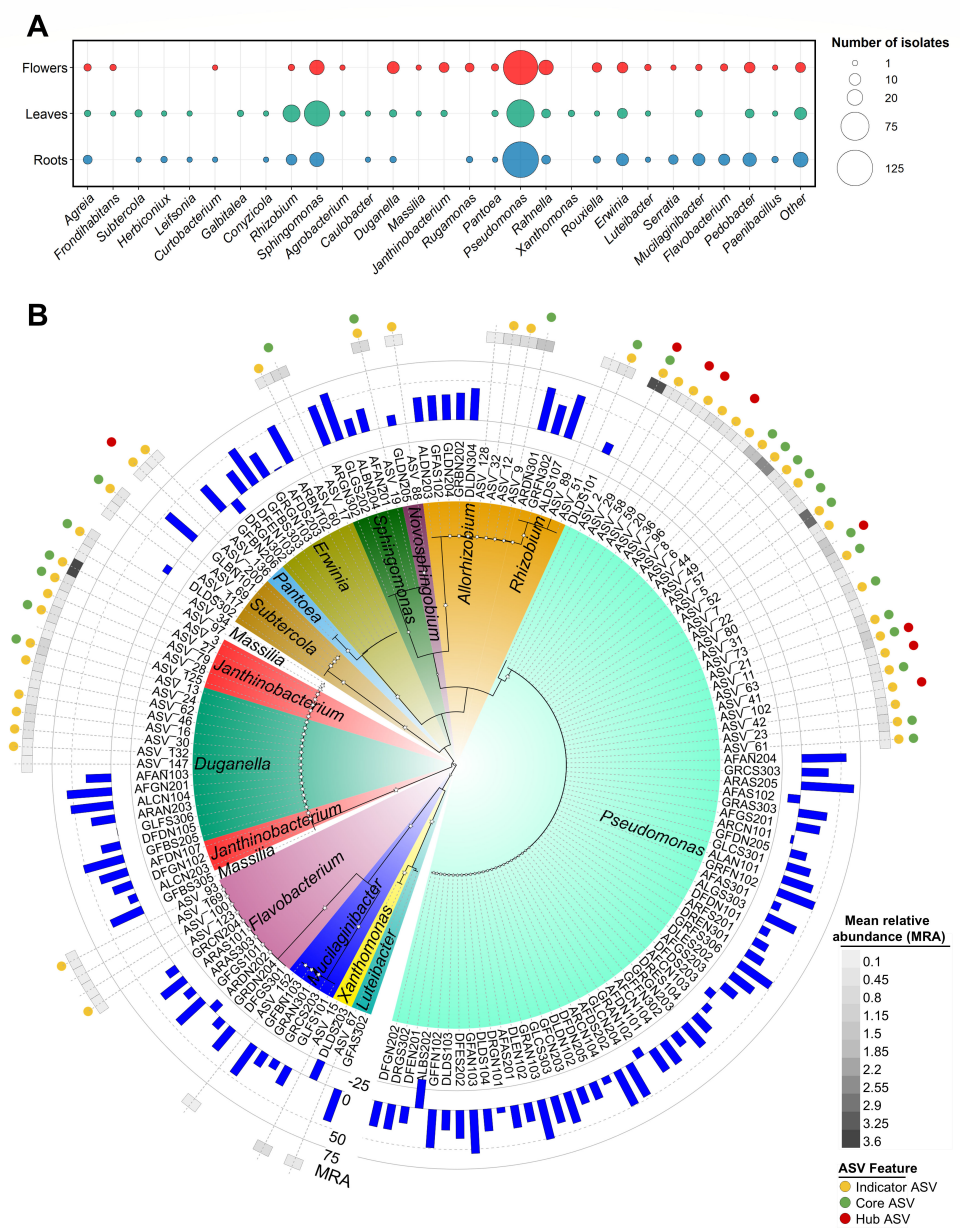
List of the psychrotolerant bacterial endophytes isolated from the selected alpine Rosaceae plants and their freezing protection ability. (**A**) Taxonomic summary of the culture collection of psychrotolerant bacterial endophytes (685 isolates) isolated from flowers, leaves, and roots of *Alchemilla* sp., *D. octopetala*, and *G. montanum*. (**B**) Phylogenetic tree of representative psychrotolerant bacterial endophytes (94 isolates) and the most abundant amplicon sequence variants (63 ASVs with mean relative abundance >0.1%). For each isolate, the blue bar represents the reduction in electrolyte leakage (%) obtained in bacterium-inoculated compared with mock-inoculated strawberry plants after freezing stress. For each ASV, gray square indicates the mean relative abundance of ASVs across all samples according to the scale legend. Colored circles indicate indicator taxa (yellow), core taxa (green), and hub taxa (red).

**Fig 8:**
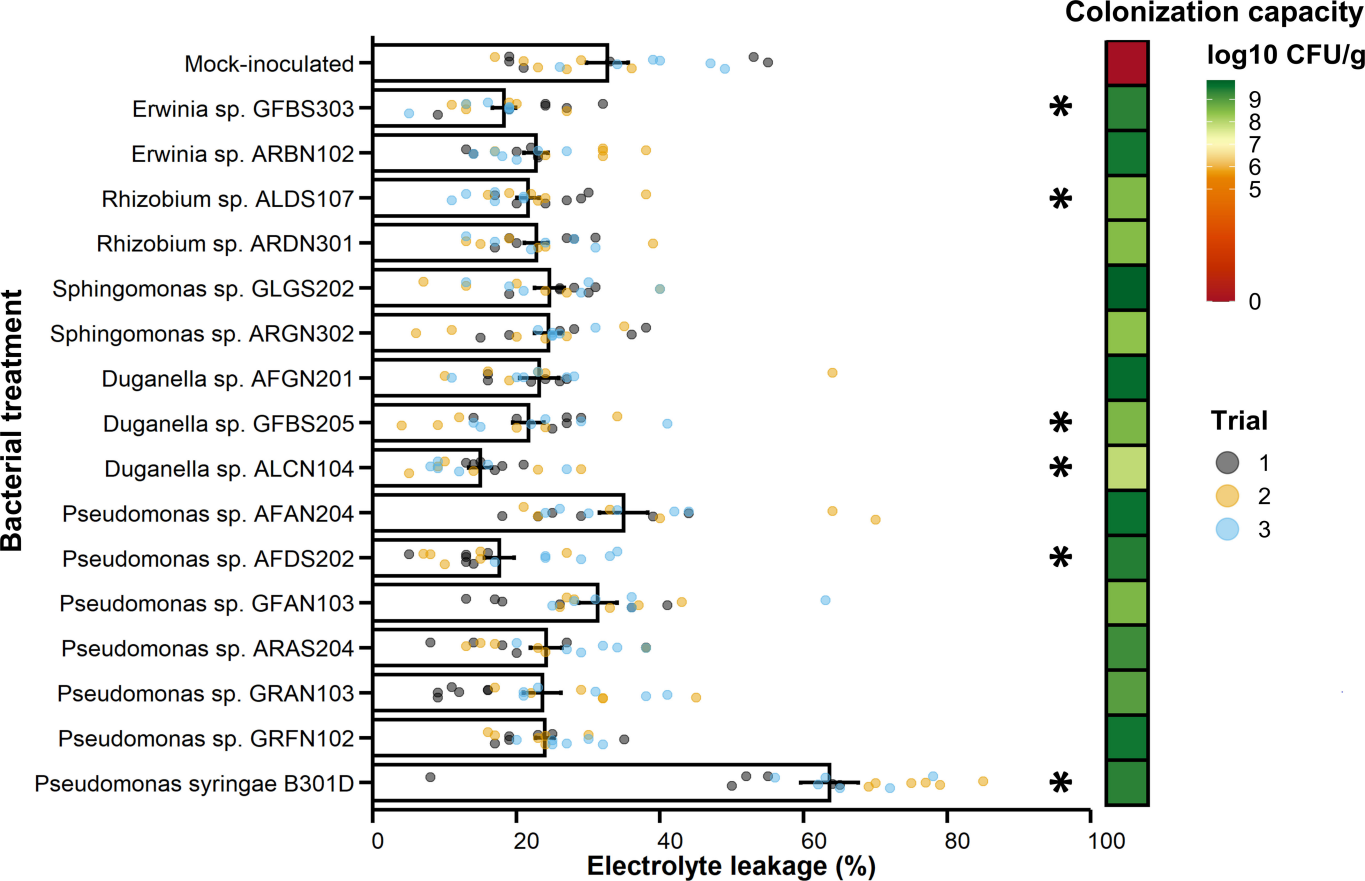
Freezing protection ability of the selected psychrotolerant bacterial endophytes isolated from the alpine Rosaceae plants. Electrolyte leakage was used to assess freezing damage in strawberry seedlings treated with MgSO_4_ (mock-inoculated) or inoculated with selected psychrotolerant bacterial endophytes of alpine Rosaceae plants. Significant differences between bacterium-inoculated and mock-inoculated samples are indicated by asterisks according to Dunnett’s test (*P* < 0.05). The colonization capacity (log10 CFU g^−1^) of the isolates was checked by dilution-plating and represented as a heatmap. Data from three independent experiments (represented by different colors) with six replicates (seedlings) each are reported. Error bars represent the standard error. *Pseudomonas syringae* B301D was used as a control for its ability to increase freezing damage (see Text S1).

To test the functional role of bacterial endophytes of alpine Rosaceae plants belonging to the core taxa, a large number of culturable psychrotolerant bacteria (*n* = 685 isolates) were isolated. The isolates belong to four phyla and 65 genera and were isolated from *Alchemilla* sp. (*n* = 296), *D. octopetala* (*n* = 132), and *G. montanum* (*n* = 257) (Fig. 7A; Table S14). The abundance of culturable psychrotolerant bacterial endophytes varied according to plant tissue, collection site, and alpine Rosaceae plant, and it was higher in root and flower tissues than in leaf tissues (Fig. S10; Table S15). The sequence search of the 16S rRNA Sanger sequences of culturable psychrotolerant bacterial endophytes against all recovered ASVs (35,300 ASVs before any filtering) revealed 94 isolates with more than 99% nucleotide identity with 63 most abundant ASVs (mean relative abundance >0.1%), and they were defined as representative psychrotolerant bacterial endophytes (Fig. 7B; Table S16). The majority (91.5%) of these representative bacteria were able to reduce electrolyte leakage in strawberry seedlings exposed to freezing stress, and the best performing isolates (reduction in electrolyte leakage >50%) belonged to the core taxa, such as *Duganella*, *Erwinia*, *Pseudomonas, Rhizobium*, and *Sphingomonas* (Fig. 7B). In the validation tests, *Duganella* sp. GFBS205, *Duganella* sp. ALCN104, *Erwinia* sp. GFBS303, *Pseudomonas* sp. AFDS202, and *Rhizobium* sp. ALDS107 confirmed the reduction in electrolyte leakage in strawberry seedlings exposed to freezing stress (Fig. 8). These bacterial isolates were able to colonize strawberry seedlings (Fig. 8) and to survive at −6°C on treated plants (data not shown).

## DISCUSSION

### Endophytic bacterial communities of alpine Rosaceae plants are affected by the plant tissue, collection site, and host plant

Endophytic bacterial communities associated with alpine Rosaceae plants were dominated by *Pseudomonadaceae* and *Oxalobacteriaceae* families across all three plant tissues (flower, leaf, and root), while enriched families were found in each plant tissue, such as *Burkholderiaceae* in flowers, *Enterobacteriaceae* in leaves, and *Rhizobiaceae* and *Microbacteriaceae* in roots. Similarly, several of these bacterial families are among the dominant endophytic taxa of other alpine plants, arctic plants (e.g., *Oxyria digyna* and *Saxifraga oppositifolia*) (28, 29) and many agronomic plants (e.g., canola, rapeseed, strawberry, tobacco, tomato, and wheat) (30–34), suggesting that such bacterial taxa are associated with plants.

Plant-associated bacterial endophytes originate from soils, rainfall, and aboveground plant niches, such as the phyllosphere (leaf surface) and anthosphere (the external environment of flowers), via wind- or insect- mediated transmission and, following their entrance, bacteria can spread systemically via the xylem to aboveground tissues (12). Bacterial richness and alpha-diversity partially decreased from roots to leaves and flowers of alpine Rosaceae plants. and only a small fraction of ASVs (less than 7%) was shared between the aboveground tissues and root tissues, suggesting a contribution of tissue-specific factors in endophytic bacterial community shaping, such as physical and chemical barriers, root exudates, and flower and leaf metabolites. Thus, the plant tissue explained the greatest amount of variation (approximately 18%–21% explained variance) in the community structure of alpine Rosaceae plants. Likewise, plant tissue showed a strong selective force in shaping plant-associated microbial communities in Antarctica and Arctic regions (35, 36), indicating that plant tissue could be a major factor affecting bacterial community structure in cold environments. In particular, ASVs belonging to the genera *Pseudomonas* (*Pseudomonadaceae*), *Escherichia–Shigella* (*Enterobacteriaceae*), *Duganella* and *Janthinobacterium* (*Oxalobacteriaceae*), and *Ralstonia* (*Burkholderiaceae*) were indicator taxa with higher abundance in aboveground tissues (flower and/or leaf tissues) compared with root tissues of alpine Rosaceae plants, indicating the possible recruitment of well-adapted bacterial taxa, such as *Escherichia–Shigella, Pseudomonas*, and *Ralstonia* previously found in aboveground tissues of strawberry and apple (33, 37, 38). For example, the *Escherichia–Shigella* taxa, although known to be inhabitants of the animal and human intestines, were characterized as one of the dominant taxa in the seeds of alpine plant species (39). The competitive colonization ability of this taxa in the interior plant tissues could be attributed to their high tolerance and potential to metabolize phytochemicals (33, 40). The effect of the plant tissue on the structure of endophytic bacterial communities indicated niche partitioning (i.e., separation among bacterial taxa in the tissue), possibly due to the variation in chemical and physical characteristics of each tissue (13).

In addition to the plant tissue, collection site (7% explained variance), alpine Rosaceae plants (5% explained variance), and exposure (less than 1% explained variance) affected the community structure of endophytic bacterial communities, which could be related to the different soil and host properties and climatic conditions. Likewise, previous studies reported the influence of environmental-related factors (e.g., soil fraction, soil texture, soil pH, and snow depth) and plant-related factors (e.g., plant species and plant neighborhood) on the structure of plant-associated bacterial communities in alpine regions (29, 41–44). For example, Brigham *et al*. (29) showed that plant species are the major driver of endophytic bacterial communities, indicating that multiple factors contribute to defining the taxonomic structure of endophytic bacterial communities of alpine Rosaceae plants. In particular, bacterial community assembly is governed by two complementary mechanisms of stochastic and deterministic processes (45). Stochastic processes may include drift (random changes in population size due to birth and death events), dispersal (movement of microorganisms between different locations), and diversification (evolutionary process that generates genetic variation), while deterministic processes may include selection (biotic and abiotic interactions resulting in fitness differences) (45). In alpine Rosaceae plants, the contribution in the assembly of endophytic bacterial communities was higher for stochastic processes of drift and dispersal compared with deterministic processes of homogeneous selection (i.e., tissue selection). Likewise, the main role of stochastic processes in shaping the bacterial community structure was previously found in alpine plants (e.g., *Elymus nutans*, *Festuca sinensis*, *Kobresia pygmaea*, and *Kobresia humilis*) (46, 47), and it is possibly associated with competitive exclusion, historical contingency (i.e., result of a disturbance effect, such as recurrent frosts) and adaptability of bacterial endophytes to variation in environmental conditions, as suggested for other plant hosts (48–54). However, the assembly of plant-associated bacterial communities can shift between stochastic and deterministic processes depending on plant genotype, age, and growth stage (21, 52, 55), indicating the possible contribution of both processes.

### Core endophytic bacterial taxa of alpine Rosaceae plants are hub taxa

The core endophytic bacterial taxa of alpine Rosaceae plants were composed of 31 ASVs belonging to the genera *Allorhizobium–Neorhizobium–Pararhizobium–Rhizobium*, *Burkholderia–Caballeronia–Paraburkholderia*, *Duganella, Erwinia, Escherichia–Shigella*, *Janthinobacterium*, *Massilia, Pseudomonas*, *Ralstonia*, and *Sphingomonas*. These taxa were previously found as a part of the core microbiome in seeds, leaves, and roots of alpine plants in the Andes, Qinghai Tibet Plateau (China), Rocky Mountains (USA), and European Alps (28, 29, 39, 44, 56), suggesting the presence of ubiquitous core taxa associated with alpine plants across divergent plant species, plant tissues, and environmental conditions. Although most of the core taxa were assembled through the drift process, several ASVs belonging to *Pseudomonas* were deterministically assembled by homogeneous selection in the aboveground tissues, suggesting their selective recruitment by alpine Rosaceae plants.

Hub taxa can be key drivers in the microbial community structure and function of a host plant, particularly if they are part of the core microbiome and are consistently present in an environment (17, 18). In alpine Rosaceae plants, hub taxa were found as part of the core taxa in aboveground tissues (flowers and leaves) but not in root tissues, and they belonged to the genera *Escherichia–Shigella*, *Pseudomonas*, and *Ralstonia*. Likewise, the genera *Escherichia–Shigella*, *Pseudomonas*, and *Ralstonia* were previously found as hub taxa of plant- and soil-associated microbial communities in agricultural lands and extreme environments (15, 57–60), suggesting that members of these genera may be adapted to a wide range of host plants and environmental conditions with a key contribution to defining microbial community structure and function. In particular, the strong interaction of hub taxa with other bacterial taxa in the co-occurrence networks of alpine Rosaceae plants revealed increased (e.g., nodes, hubs, and average degree) and decreased (e.g., average path distance) topological properties in roots compared with flowers and leaves, suggesting strong complexity, connectivity, and bacterial interactions in roots, as previously reported for various plants (e.g., strawberry) (58, 61, 62).

### Culturable psychrotolerant bacterial endophytes contribute to plant freezing stress tolerance

Culturable psychrotolerant bacterial endophytes represent a selection of endophytic bacterial communities associated with alpine Rosaceae plants, and they succeed in mitigating freezing stress in strawberry seedlings. In particular, *Erwinia* (*Erwiniaceae*), *Duganella* (*Oxalobacteriaceae*), *Pseudomonas* (*Pseudomonadaceae*), and *Rhizobium* (*Rhizobiaceae*) isolates reduced electrolyte leakage of strawberry seedlings exposed to freezing stress and belonged to core endophytic bacterial taxa according to the bioinformatics analysis. The functional importance of core bacterial taxa was previously shown for maize growth (15) and banana health (16), suggesting that core bacterial taxa are selected by the host plant to provide beneficial effects on plant growth and survival under stress conditions. Likewise, previous works showed that *Pseudomonadaceae* and *Oxalobacteraceae* families were enriched under cold stress in cold-sensitive (e.g., sorghum, taxus, lettuce, and maize) and cold-adapted (e.g., *Valerianella locusta*) plants (23, 24, 63, 64). Moreover, bacterial isolates belonging to *Pseudomonadaceae, Oxalobacteraceae*, and *Rhizobiaceae* promoted plant growth under cold stress and/or reduced cold stress-related damage in several plants (22, 26, 64–67). For example, inoculation with rhizobia (*Sinorhizobium meliloti*) led to a high proportion of undamaged nodules after freezing stress in alfalfa (67) and inoculation with *Pseudomonas* spp. (lacking the *ina* gene responsible for ice nucleation) improved freezing stress tolerance in Rosaceae plants (26, 68), corroborating that some plant-associated bacterial taxa contribute to cold stress mitigation.

This study focused on identifying functional bacteria in the plant endosphere that could contribute to host tolerance to freezing stress, and thus probably missing functionally relevant epiphytic bacteria in other niches such as the phyllosphere and rhizosphere. It was shown that microbial communities including bacterial communities inhabiting similar niches could improve the plant tolerance to cold stress (23, 69). Therefore, future studies characterizing the epiphytic and rhizosphere bacterial communities of alpine plants may lead to the identification of additional plant–bacterium interactions under freezing stress.

Some *Pseudomonas*-based biostimulants for the mitigation of frost damage are already available on the market, mostly applied to vegetative plant tissues (e.g., leaves) to control *ina*-producing bacterial populations (70). Our discovery of the potential of *Pseudomonas* and non-*Pseudomonas* species in freezing stress mitigation expands on this by offering isolates acting through mechanisms other than controlling *ina*-producing bacteria. The next step would be evaluating the effect and mode of action of the most promising isolates on vulnerable plant tissues, such as flowers, to further develop a cost-effective strategy to mitigate spring frost on fruit tree crops. Moreover, some taxa found in the collection of bacterial isolates were not detected by the amplicon sequencing, suggesting that additional primer pairs and/or DNA extraction methods are required to have a more complete overview of the endophytic bacterial communities associated with alpine plants.

### Conclusion

In agreement with previous studies on other species, we found that the selected alpine Rosaceae plants of this study are colonized by complex endophytic bacterial communities whose taxonomic structures are affected by plant tissue, collection site, and host plant. These communities are assembled through stochastic processes of drift and deterministic processes of homogeneous selection. Moreover, the identified core, hub, and indicator endophytic bacterial taxa could play important functions in alpine Rosaceae plants. In particular, culturable psychrotolerant bacterial endophytes isolated from these alpine Rosaceae plants can mitigate freezing stress in strawberry plants, suggesting that some members of endophytic bacterial communities can contribute to cold stress mitigation in the host plants. The bacterial isolates that provided efficient freezing mitigation belonged to *Duganella, Erwinia, Pseudomonas*, and *Rhizobium*, indicating that functional characterization of core endophytic bacterial taxa is the starting point for further manipulation of the plant microbiome through a stable synthetic community and for the development of efficient microbial biostimulants for cold stress mitigation in agriculture. Future studies should aim to understand the factors affecting the functionality of core microbiota as well as the mode of action, effective target site, and performance of the most promising psychrotolerant bacterial endophytes under controlled and field conditions.

## MATERIALS AND METHODS

### Plant material, experimental design, and sample processing

Samples of three alpine Rosaceae plants (*Alchemilla* sp., *D. octopetala*, and *G. montanum*) were collected in Alpine areas of the Trentino-alto Adige Region, Italy, specifically in seven sites and two exposures (i.e., exposure to solar radiation, North and South) (Fig. 1; Table S1; Text S1). The sampling was carried out between June–August 2021 and three replicates were collected. Flowers, leaves, and roots were cut from each sampled plant resulting in a total of 270 samples. The samples were surface disinfected, air-dried, and half of each sample was stored at −80°C for amplicon sequencing analysis, whereas the other half was used for the isolation of culturable bacteria (Text S1).

### DNA extraction, amplification, library preparation, and amplicon sequencing

DNA extraction, amplification, and amplicon sequencing was carried out as previously described with some modifications (71) (Text S1). Briefly, genomic DNA was extracted from surface-disinfected tissues using the FastDNA Spin Kit for Soil (MP Biomedical, Santa Ana, CA, USA). The bacterial V5–V7 region of 16S ribosomal DNA (rDNA) was amplified with a nested PCR approach with 799F/1392R and 799F/1175R primers for the first and second 16S amplification, respectively (72, 73), and sequenced on an Illumina MiSeq at Eurofins Genomics GmbH (Ebersberg, Germany).

### 16S rRNA gene amplicon sequence processing and analyses

16S rRNA gene amplicon sequence processing was carried out as previously described (71). Bacterial ASV tables were split into two datasets: (i) dataset for *Alchemilla* sp. and *G. montanum* from six collection sites (termed dataset 1; 216 samples) and (ii) dataset for *Alchemilla* sp., *D. octopetala*, and *G. montanum* from two collection sites (termed dataset 2; 108 samples). This approach was performed to obtain consistent numbers of alpine Rosaceae plants from the different collection sites since *D. octopetala* was not present in some collection sites (Table S1). The same following analyses (also in Text S1) were performed on both datasets. Alpha-diversity values were calculated by the multiple rarefaction method for both richness and diversity values by averaging the results inferred after 999 rarefactions using the rtk R package (74). Beta-diversity values were normalized using the multiple rarefaction method as described above. PERMANOVA global and partitioning test by tissues as well as CAP were conducted on Bray–Curtis dissimilarity matrices using the vegan R package (75). Core endophytic bacterial taxa were identified based on the abundance and occupancy distribution as previously described (76, 77). The relative importance of deterministic and stochastic assembly processes was investigated using the NST and iCAMP R packages (78, 79). Random forest models were performed using a machine learning algorithm, as previously described (71). For the differential abundance analysis, ANCOM-BC2 was used on nonrarefied count data from all ASVs (80). Co-occurrence networks were constructed using the NetCoMi v1.1.0 R package, as previously described (81, 82), and hub ASVs were identified as those that are significantly more connected within the network than other ASVs on the basis of centrality measures (18). Multiple sequence alignment for both Sanger (psychrotolerant bacterial endophytes) and ASVs (most abundant bacterial ASV) sequences was performed using MAFFT v7 (83). A phylogenetic tree was inferred from partial 16S rRNA gene sequences based on the neighbor-joining algorithm with the Jukes–Cantor correction and 1,000 resampling bootstraps (83). The tree was visualized and annotated using Archaeopteryx and iTOL v5, respectively (84, 85). All plots were generated using ggplot2 v3.4.2 (86) and patchwork v1.1.2 (87) R packages. A detailed description of the analysis of 16S rRNA gene amplicon sequencing is provided in Text S1 in the supplemental material.

### Isolation and taxonomic annotation of culturable psychrotolerant bacterial endophytes

Isolation and identification of the bacterial endophytes of alpine Rosaceae plants were performed as previously described (88, 89) (Text S1). To avoid redundancy, bacterial isolates were taxonomically annotated based on sequencing of the V6–V8 region of the 16S rRNA gene (71). To obtain a list of representative psychrotolerant bacterial endophytes corresponding to the most abundant ASVs (24), a custom-built database containing all the recovered ASV sequences was constructed using the makeblastdb function from the rBLAST package. Sanger sequences were then queried against the customized ASV database using BLAST (Text S1).

### Screening of psychrotolerant bacterial endophytes for their freezing protection ability

Representative psychrotolerant bacterial endophytes were tested for their freezing protection ability. Seeds of strawberry (*Fragaria* ×*ananassa*) cultivar Fresca (Moles Seeds Ltd, Essex, United Kingdom) were germinated as previously described (90) (Text S1). Germinated seeds were transferred to 12-well plates (Greiner-Bio one, Merck) containing solid full-strength Hoagland (Sigma-Aldrich, Merck) and placed in the growth chamber (Text S1). After 1 week, seedlings were treated with 10 µL of sterile 10 mM MgSO_4_ (mock-inoculated) or inoculated with a bacterial suspension (bacterium-inoculated) by distributing small droplets on the first two leaves (91). Seedlings were incubated in the growth chamber for 25 days and exposed to freezing stress as previously described (92) before measuring electrolyte leakage (Text S1). At the end of the freezing bioassay, the colonization of strawberry seedlings by psychrotolerant bacteria was evaluated according to Galambos et al. (93) (Text S1).

## Data Availability

Amplicon sequencing data are available from the NCBI Sequence Read Archive under the BioProject number PRJNA872124. Sanger sequencing data are available under the NCBI GenBank accession numbers OR809314–OR809998 (accession number for each bacterial isolate are reported in Table S14).

## SUPPLEMENTAL MATERIAL

Text S1 mbio.01418-24-s0001.pdf Supplemental methods.

Supplemental figures mbio.01418-24-s0002.pdf Fig. S1 to S10.

Supplemental tables mbio.01418-24-s0003.pdf Tables S1, S2, S4 to S13, S15, and S17.

Table S3 mbio.01418-24-s0004.xlsx Bacterial ASVs of endophytic bacterial communities associated with alpine Rosaceae plants.

Table S14 mbio.01418-24-s0005.xlsx Taxonomic annotation at the genus level for the culturable psychrotolerant bacterial endophytes from alpine Rosaceae plants.

Table S16 mbio.01418-24-s0006.xlsx Identification of representative psychrotolerant bacterial endophytes of Rosaceae plants.

ASM does not own the copyrights to Supplemental Material that may be linked to, or accessed through, an article. The authors have granted ASM a non-exclusive, world-wide license to publish the Supplemental Material files. Please contact the corresponding author directly for reuse.
