# Supplementary material for: Amplicon sequencing and culture-dependent approaches reveal core bacterial endophytes aiding freezing stress tolerance in alpine Rosaceae plants
Source: mBio. 2025 Feb 25;16(4):e01418-24. doi: 10.1128/mbio.01418-24 (PMC11980557; doi:10.1128/mbio.01418-24)
Supplement: Text S1 — Supplemental methods. [file mbio.01418-24-s0001.pdf]

Supplementary methods

Amplicon sequencing and culture-dependent approaches reveal core bacterial endophytes aiding freezing stress tolerance in alpine Rosaceae plants

Malek Marian,[a]#, Livio Antonielli,[b] Ilaria Pertot,[a,c] Michele Perazzolli[a,c]

[a]Center for Agriculture Food Environment (C3A), University of Trento, San Michele all'Adige, Italy

[b]Department of Health and Environment, Bioresources Unit, AIT Austrian Institute of Technology, Tulln an der Donau, Austria

[c]Research and Innovation Centre, Fondazione Edmund Mach, San Michele all'Adige, Italy

Running Head: Endophytic Bacterial Communities of Alpine Plants

#Address correspondence to: Malek Marian, malekmarian@hotmail.com

Plant material, experimental design, and sample processing

Samples of three alpine Rosaceae plants (*Alchemilla* sp., *Dryas octopetala*, and *Geum montanum*) were collected in Alpine areas of the Trentino-alto Adige Region, Italy, specifically in seven sites (Val di Non, Val di Sole, Val di Pejo, Val Rendena, South Tyrol, Stelvio Park, and Val di Fassa, hereafter named Site A, B, C, D, E, F, and G, respectively) and two exposures (i.e., exposure to solar radiation, North and South) (Fig. 1), to obtain alpine Rosaceae plants from different altitudes and climatic conditions (Table S1). The sampling was carried out from the middle of June to the middle of August 2021 to match the flowering period of each species at each site. For each alpine Rosaceae plant/site/exposure, three replicates were collected, each of which was collected from 15–20 randomly chosen plants with no visible signs of damage or disease, transported to the laboratory in a cool box, stored in a refrigerator at 4°C and processed within 24 hours after collection. A total of 90 plant samples (Table S1) were analyzed from three alpine Rosaceae plants: 36 samples from *Alchemilla* sp., 18 samples from *D. octopetala* and 36 samples from *G. montanum*.

Flowers, leaves, and roots were cut from each sampled plant resulting in a total of 270 samples. The samples were surface disinfected with 70% ethanol for 1 min, 1% sodium hypochlorite for 1 min (flowers), 2.5% sodium hypochlorite for 5 min (leaves), or 9% sodium hypochlorite for 10 min (roots), followed by 70% ethanol for 1 min and five washes with sterile distilled water (SDW) of 2 min each. Samples were air-dried under laminar flow for 30 min. Half of each sample was frozen with liquid nitrogen and stored at −80°C for DNA extraction and amplicon sequencing analysis, whereas the other half was used for the isolation of culturable bacteria. As a control for plant surface disinfection, the last washing solution (20 mL) was centrifuged (3,500 g for 10 min), the supernatant was discarded and aliquots (20 µL) of the remaining solution (500 µL) were plated on solid Reasoner’s 2A (R2A; Sigma-

Aldrich, Merck, Rahway, NJ, USA) to confirm the absence of bacterial growth seven days after incubation at 25°C.

DNA extraction, amplification, library preparation, and amplicon sequencing

Culture-independent analysis of DNA extraction, amplification, and sequencing was carried out as previously described with some modifications (1). Genomic DNA was extracted from surface-disinfected tissues using the FastDNA Spin Kit for Soil (MP Biomedical, Santa Ana, CA, USA) with a slight modification. In particular, surface-disinfected tissues (1 g) were homogenized in sterile stainless jars on liquid nitrogen using a mixer-mill disruptor (MM 400, Retsch, Haan, Germany) at 25 Hz for 10 s. Powdered frozen tissues were added to a lysing matric E tube (MP Biomedical) and homogenized with the FastPrep-24 Classic instrument (MP Biomedical) at a speed of 4.0 for 5 s (flowers) or 30 s (leaves and roots). The bacterial V5–V7 region of 16S ribosomal DNA (rDNA) was amplified with a nested PCR approach (2). The first bacterial 16S amplification was carried out with the primers 799 forward (799F) (5'-AACMGGATTAGATACCCKG-3') and 1392 reverse (1392R) (5'-ACGGGCGGTGTGTRC-3'), to exclude chloroplast 16S rDNA and to amplify bacterial and mitochondrial rRNA of 600 bp and 1,000 bp amplicon size, respectively (2, 3). Bacterial 16S amplicons were purified by agarose gel separation, followed by the NucleoSpin Gel and PCR Clean-up purification kit (Macherey-Nagel, Düren, Germany). The second 16S amplification was performed with the primers 799 forward (799F) (5'-AACMGGA TTA GAT ACC CKG-3') and 1175 reverse (1175R) (5'-ACGTCRTCCCCDCCTTCCT-3') including the specific overhang Illumina adapters (5'-ACACTCTTTCCCTACACGACGCTCTTCCGATCT-3' and 5'-GACTGGAGTTCAGACGTGTGCTCTTCCGATCT-3', respectively) for amplicon library construction, and 16S amplicons (500 bp amplicon size) were purified by agarose gel separation using the NucleoSpin Gel and PCR Clean-up purification kit (Macherey-Nagel). Bacterial 16S amplifications were obtained using the FastStart High-Fidelity PCR system

(Roche, Branford, CT, USA) with PCR amplification conditions optimized for each plant tissue (Table S17). A quality check was performed by gel electrophoresis to confirm the presence of the expected amplicon using 5 µL of PCR product. PCR product purification, quantification, library construction, and sequencing were conducted by Eurofins Genomics GmbH (Ebersberg, Germany) following their in-house NGSelect Amplicons approach with an index PCR to introduce indexed sequencing adaptors for sample discrimination after Illumina MiSeq (PE300 mode) sequencing.

16S rRNA gene amplicon sequence processing and analyses

Amplicon sequence processing. 16S rRNA gene amplicon sequence processing was carried out as previously described (1). Illumina reads were filtered with Bowtie2 v2.4.2 (4), sequence quality was checked with FastQC v0.11.9 and primers were cut using Cutadapt v3.4 (5). Sequences were quality filtered, trimmed, denoised (filtered read counts), and ASVs were generated with DADA2 v1.18.0 (6). Denoised forward and reverse ASV sequences were merged and chimera filtered. Bacterial ASVs were checked using Metaxa2 v2.2.3 (7) to ensure precise targeting of the specific V5 to V7 hypervariable regions of the 16S rRNA gene that our primers are intended to amplify and to mitigate the potential impacts of discrepancies during the merging process of reads by refining the selection of ASVs. Taxonomic classification and assignment were conducted using the SILVA v138.1 as the reference database (8) and the RDP classifier implemented in DADA2, respectively. A bacterial table of read counts was built and imported into the R-4.3.0 statistical environment for further analyses (9). After taxonomic classification, ASVs classified as plastid rRNA and other than archaea or bacteria were removed. ASVs with no reads or singletons, as well as very low-abundance ASVs with a maximum relative abundance below 0.1% per sample, were discarded before any further analysis.

Datasets. Bacterial ASV tables were split into two datasets. In particular, the design incorporated four categorical orthogonal factors in dataset 1, such as Tissue (three levels: flowers, leaves, and roots), alpine Rosaceae plants (two levels: *Alchemilla* and *Geum*), Collection site (six levels: Site A, B, C, D, F, and G) and Exposure (two levels: North and South), and four categorical orthogonal factors in dataset 2, such as Tissue (three levels: flowers, leaves, and roots), alpine Rosaceae plants (three levels: *Alchemilla*, *Dryas*, and *Geum*), Collection sites (two levels: Site D and G) and Exposure (two levels: North and South).

Alpha-diversity. Alpha-diversity values were calculated by the multiple rarefaction method for both richness (observed ASVs) and diversity (estimated with Simpson's index) values by averaging the results inferred after 999 rarefactions, starting with the lowest read counts in a sample (n = 13,869) for the complete dataset, using the rtk R package (10). The rarefaction was repeated for each dataset and then values were fitted to the linear models (LMs) together with the four factors (i.e., plant tissue, alpine Rosaceae plant, collection site, and exposure). LMs with the lowest root mean squared error (RMSE) identified using the caret R package (11) were chosen for the analysis of variance (ANOVA) followed by post-hoc analysis with estimated marginal mean comparisons using the emmeans R package (12). Conditional inference regression tree analysis was further applied to visualize the hierarchy among different factors on the alpha-diversity metrics (13).

Beta-diversity. Beta-diversity values were normalized using the multiple rarefaction method to account for differences in sequencing depth as described above for the alpha-diversity. Permutational multivariate analysis of variances (PERMANOVA) global test and PERMANOVA partitioning test by tissues were conducted on Bray–Curtis dissimilarity matrices using the adonis2 function from the vegan R package (14) to determine the differences in centroids of the bacterial communities across samples in dataset 1 and dataset 2. In addition, Intra-factor pairwise comparisons between levels were carried out using

pairwise.perm.manova function from the RVAideMemoire R package with *P*-value adjustment using Benjamini-Hochberg method (15). To identify the drivers of bacterial community structure in each tissue, permutational multivariate analysis of variances (PERMANOVA) tests were performed separately for the different tissues. In addition, the host-environment effects index (HEEI = relative contribution of alpine Rosaceae plants/relative contribution of the collection site) was calculated based on PERMANOVA (16). A constrained analysis of principal coordinates (CAP) based on Bray–Curtis dissimilarity matrices was carried out using the capscale function from the vegan R package, and the significance of constraints was confirmed by a permutational test (999 iterations). Differences in multivariate homogeneity of group dispersions (PERMIDISP2) were also evaluated using the betadisper function from the vegan package, followed by ANOVA and permutational test (999 iterations).

Multivariate generalized linear models (mGLMs). The distance-based PERMANOVA was complemented with mGLMs, which account for the mean-variance relationship of the data (17, 18). mGLMs were fitted to the rarefied counts of bacterial ASVs with an occupancy (frequency of detection) of 0.25 to reduce complexity and computation time. A negative binomial distribution of the data was graphically verified by plotting fitted vs residual values, as previously described (19). The manyglm function from the mvabund R package (16) was used with the full model (Tissue*alpine_Rosaceae_plant*Collection_site*Exposure), as it was identified to be the best-performing model based on the obtained Akaike information criterion (AIC) values. An analysis of deviance (Dev) was calculated with a likelihood-ratio test using a permutational test (1000 iterations, Monte Carlo resampling). A model-based ordination diagram was generated using the ordiplot function after performing generalized linear latent variable models with the variables using the gllvm R package (20). The model fit was restricted to five latent variables as identified by the lowest AIC values.

Hierarchical clustering. Prior to hierarchical clustering, rarefied count data were standardized with the Wisconsin double standardization method, and Bray–Curtis dissimilarity matrices were calculated using the vegdist function from the vegan package. Hierarchical clustering analysis was performed using the agnes function with the Ward. D method (identified as the strongest clustering structure by agglomerative coefficient test) from the cluster R package (21).

Abundance/occupancy distribution. Core endophytic bacterial taxa of Rosaceae plants (set of bacterial taxa that are characteristic of a host plant; 22) were identified based on the abundance and occupancy distribution using the same R code and function as previously described (23, 24). The mean relative abundance and occupancy of each taxon were calculated across the rarefied count data of the complete dataset. Taxa were then ranked by occupancy with an additional weight for taxa with an occupancy of 1 in a particular tissue. Bray–Curtis dissimilarities were calculated for the first ranked taxa as well as the complete dataset. The percent contribution of the prospective core set to beta-diversity was calculated by dividing the Bray-Curtis dissimilarities of the first ranked taxa by the Bray-Curtis dissimilarities of the complete dataset. The next ranked taxa were added sequentially to find the point in the ranking at which the addition of one more taxa offers diminishing returns on explanatory value for beta-diversity. The final 2% increase in the collective contribution of ranked taxa to the Bray–Curtis dissimilarities were used to infer core taxa members. Taxa abundance/occupancy data were further fitted to the Sloan neutral model [95% confidence intervals, goodness of fit (R2) and estimated migration rate (*m*; a measure of dispersal limitation)] to evaluate the importance of stochastic and deterministic processes in the overall bacterial community assembly.

NST and iCAMP (Infer Community Assembly Mechanisms by Phylogenetic-bin-based null model). The relative importance of each assembly process of homogeneous and

heterogeneous selection (i.e., deterministic), as well as dispersal limitation, homogenizing dispersal, drift and others (i.e., stochastic) was investigated using the NST and iCAMP R packages (25, 26). The dominance of deterministic and stochastic assemblies was determined with NST when the phylogenetic normalized stochasticity ratio (pNST) was lower and higher than 50%, respectively. The parameter settings for iCAMP were 0.2 phylogenetic distance, 24 minimal bin size requirement threshold and SES.RC as the null model significance test. The latter setting uses the beta net relatedness index (βNRI) for phylogenetic beta diversity and the modified RaupCrick for taxonomic beta-diversity. For the community assembly analysis, a phylogenetic tree was constructed by aligning all ASVs and calculating pairwise distance between them before tree inference using the msa, seqinr, and ape R packages (27, 28).

Random forest models. Random forest models were performed to identify taxa with strong associations with the four factors (i.e., plant tissue, alpine Rosaceae plant, collection site, and exposure) using a machine learning algorithm, as previously described (1). Rarefied count data were used as predictors, and factors were used as response data. Model performances were evaluated with repeated k-fold cross-validation (tenfold, 10 repetitions) and mtry values that determined the highest model accuracy were chosen and input to random forest analysis, as implemented in the caret R package (11). Variable importance was assessed with permutations (999 iterations) using the rfPermute R package.

ANCOM-BC2 (Analysis of compositions of microbiomes with bias correction 2). For the differential abundance analysis, ANCOM-BC2 was used on nonrarefied count data from all ASVs (29). ASVs with structural zeros (i.e., ASVs present in at least one group of flowers, leaves, and roots but absent in at least one group of other tissues) were not considered in further data analysis. Plant tissue, alpine Rosaceae plant, collection site, and exposure were implemented as four covariates in the model formula

(Tissue+alpine_Rosaceae_plant+Collection_site+Exposure), and ANCOM-BC2 primary analysis was used to determine ASVs that were differentially abundant according to covariates. All log-ratios for each taxon were then tested for significance using a two-sided Z-test with W, and *P*-values were adjusted using the Benjamini–Hochberg method (15).

Co-occurrence networks. Co-occurrence networks were constructed to characterize the relationship between core taxa and the whole community using the NetCoMi v1.1.0 package, as previously described (30, 31), and hub ASVs were identified as those that are significantly more connected within the network than other ASVs on the basis of centrality measures (32). In addition, networks were built by tissue to reveal differences in bacterial associations and interactions within functional taxa underlying plant tissue tolerance to freezing stress. To construct the correlation matrix, the SpiecEasi v1.1.2 tool (33) was used on nonrarefied count data as SpiecEasi performs centered log-ratio normalization internally. The data consisted of ASVs with 0.001% relative abundance and occupancy of 0.25 for each group of tissue (i.e., flowers, leaves, and roots). Association matrices were estimated using the Meinshausen and Bühlmann algorithm with the nlambda set to 100, sampled 100 times, lambda.min.ratio set to 10^{-1}, and with the stars model selection. Matrices were then used to construct the network using the netConstruct function and network properties were determined using the netAnalyze function. Hub nodes were identified based on eigenvector centrality values above 95% of the empirical distribution of all eigenvector centralities in the network (32). The network was visualized using the plot.microNetProps function. Quantitative network comparisons were performed with the netCompare function using 1000 permutations, and the Jaccard index was used to assess how different the sets of most central nodes (i.e., set of ASVs with eigenvector centrality values greater than 95% of the empirical distribution of all eigenvector centralities in the network) were between the two groups (0 if the sets were completely different and 1 for exactly equal sets).

Isolation and taxonomic annotation of culturable psychrotolerant bacterial endophytes

Isolation of culturable psychrotolerant bacteria. Isolation and identification of the bacterial endophytes of alpine Rosaceae plants were performed according to previously described protocols (34, 35). Surface-disinfected flower, leaf, or root tissues (1 g) originating from the same plant samples used for amplicon sequencing were homogenized in SDW using refrigerated (ice) sterile stainless steel with a mixer-mill disruptor (MM 400, Retsch) at 25 Hz for 30 s, 2 min, or 10 min for flowers, leaves, and roots, respectively. Culturable bacteria were isolated by plating serial dilutions of each suspension (100 μL aliquots) on solid R2A medium supplemented with 50 mg L^{-1} cycloheximide to prevent fungal contamination. Plates were incubated at 4°C to allow the growth of psychrotolerant isolates, and bacterial colony forming units (CFU) per unit of plant fresh weight (CFU g^{-1}) were assessed after 30 days. Three technical replicate plates were used for each dilution and each sample. Representative bacterial isolates were selected for each sample based on morphological visual observation of bacterial colonies (e.g., size, color, opacity, texture, form, elevation, and margin), as previously described (1). The purified colonies were suspended in 40% (v/v) glycerol and kept at −80°C until use.

Sanger sequencing. To avoid redundancy, bacterial isolates were taxonomically annotated based on sequencing of the V6–V8 region of the 16S rRNA gene. PCR amplification and DNA sequencing were performed with the universal primers 27 forward (5’-AGAGTTTGATCCTGGCTCAG-3’ and 1492 reverse (5’-GGTTACCTTGTTACGACTT-3’) using the conditions previously described (1). Briefly, the PCR product was purified using a NucleoSpin Gel and PCR Clean-up purification kit (Macherey-Nagel) and subjected to Sanger sequencing at Eurofins Genomics GmbH (Ebersberg, Germany). Forward and reverse sequences were assembled by using Chromas Pro software version 1.7

(Technelysium Pty Ltd., Tewantin, Australia), and taxonomy was assigned to the genus level by comparing sequences with those of the 16S ribosomal RNA database in the National Center for Biotechnology Information (NCBI) using the rBLAST and taxonomizr R packages (36, 37). To obtain a list of representative psychrotolerant bacterial endophytes and to further reduce the number of culturable isolates, we used a similar approach that focuses on isolates matching those identified as the most abundant ASVs, a useful strategy to obtain potential cold stress alleviating bacteria (38). For this, a custom-built database containing all the recovered ASV sequences (n = 35,300 ASVs before any filtering) was constructed using the makeblastdb function from the rBLAST package. Sanger sequences were then queried against the customized ASV database using BLAST, parameters of identity 95%, E-value 1×10^{-5}, max target 10 and max HSPS 10 were used with the predict function of the rBLAST package and taxa of culturable psychrotolerant bacterial endophytes corresponding to the most abundant ASVs (mean relative abundance > 0.1%) were considered as representative psychrotolerant bacterial endophytes.

Screening of psychrotolerant bacterial endophytes for their freezing protection ability

Bacterial inoculum preparation. Representative psychrotolerant bacterial endophytes were tested for their freezing protection ability. Each isolate was grown overnight (18 h) in liquid R2A medium at 25°C under orbital shaking at 200 rpm and bacterial cells were collected by centrifugation (3,500 × g for 10 min) and washing (three times) with sterile 10 mM $MgSO_4$. The bacterial suspension was then adjusted to an OD_{600} = 0.1 corresponding to approximately1.0 × 10^8 CFU mL^{-1}.

Seed germination. Seeds of strawberry (*Fragaria* × *ananassa*) cultivar Fresca (Moles Seeds Ltd, Essex, United Kingdom) were germinated as previously described (39). Seeds were stratified at 4°C for 3 weeks and scarified with cold 96% sulfuric acid (H_2SO_4) for 10 min,

followed by five washes with SDW (2 min each). Seeds were surface-disinfected with 70% ethanol for 1 min, 1% sodium hypochlorite for 1.5 min, and 70% ethanol for 1 min, followed by five washes with SDW. Surface-disinfected seeds were sown on two layers of filter paper (Whatman, GE Healthcare Life Sciences) in Petri dishes (90 mm in diameter) moistened with 5 mL of SDW. The plates were incubated in a growth chamber at 23°C ± 1°C for four days with a photoperiod of 14 h/10 h light/dark.

Bacterial treatment: Germinated seeds were transferred to 12-well plates (Greiner-Bio one, Merck) containing solid (7 g L−1 agar) full-strength Hoagland (2.75 mL well−1; pH adjusted to 6.5) (Sigma-Aldrich, Merck), sealed with parafilm and placed in the growth chamber. After 1 week, seedlings were treated with 10 µL of sterile 10 mM MgSO4 (mock-inoculated) or inoculated with a bacterial suspension (bacterium-inoculated) by distributing small droplets on the first two leaves (40). The leaf was chosen as the main target site due to the difficulty to screen and evaluate the freezing protection ability of a large number of bacterial isolates directly on flowers as it would be expensive, labor-intensive, and time-consuming. As an additional control, seedlings were inoculated with *Pseudomonas syringae* B301D (obtained from The Leibniz Institute DSMZ, Germany), as this strain possesses an *ina* gene and is thus capable of accelerating freezing stress damage to plants (41). Seedlings were incubated in the growth chamber for 25 days and exposed to freezing stress as previously described (42) before measuring electrolyte leakage.

Electrolyte leakage measurement. Seedlings plates were placed in a freezing chamber (MIR-154, Sanyo, Japan) at 4°C for 30 min, and the temperature was adjusted by a cooling rate of 2°C h^{-1} and then held at −6°C for 6 h in darkness. After the freezing treatment, seedlings were allowed to thaw in darkness for 4 h by increasing the temperature up to 4°C at a rate of 2°C h^{-1} before being transferred back to the growth chamber for 24 h. Electrolyte leakage was measured by submerging the seedlings in 15-mL tubes containing 10 mL of

SDW. Tubes were shaken overnight (18 h) at 150 rpm at 25°C, and the electrical conductivity (EC1) was measured using a hand-held conductivity meter (pH CO 1030, pH/Conductivity Tester VWR, Milan, Italy). Samples were autoclaved and the total electrical conductivity (EC2) was measured. Electrolyte leakage was calculated as follows: (EC1)/(EC2) × 100. The reduction in electrolyte leakage (%) was calculated as follows: [1 − (mean percent electrolyte leakage in the bacterial treatment)]/ (mean percent electrolyte leakage in the control treatment) × 100. In the first trial, six replicates (seedlings) were analyzed for each representative psychrotolerant bacterial endophyte, and the best performing isolates were selected. To validate the results, six replicates (seedlings) were analyzed for each best performing isolate of representative psychrotolerant bacterial endophytes, and the experiment was carried out three times.

Bacterial colonization. At the end of the freezing bioassay, the colonization of strawberry seedlings by psychrotolerant bacteria was evaluated according to Galambos *et al.* (43). Leaf tissues from three strawberry seedlings (0.5 g) were homogenized in sterile stainless jars using a mixer-mill disruptor at 25 Hz for 30 s in sterile 10 mM $MgSO_4$. Dilutions of the plant homogenate (10 µL aliquots) were spotted on solid R2A media. The plates were incubated at 25°C for three days, and the number of colonies of each bacterial isolate was counted. Five technical replicate plates were analyzed for each treatment and dilution and the experiment was carried out two times.

Statistical analysis. Colony forming unit (CFU) counts were Log_{10}-transformed and LMs followed by ANOVA, and post-hoc analysis was performed with estimated marginal mean comparisons using the caret R package and emmeans R package. Electrolyte leakage values were normalized by arcsine transformation followed by pairwise comparison between bacterium-inoculated and mock-inoculated samples with Dunnett's test using the DescTools

R package (44). Data were checked for normality and homoscedasticity using Shapiro–Wilk and Levene's test using the rstatix R package (45), respectively.

REFERENCES

1. Perazzolli M, Vicelli B, Antonielli L, Longa CMO, Bozza E, Bertini L, Caruso C, Pertot I. 2022. Simulated global warming affects endophytic bacterial and fungal communities of Antarctic pearlwort leaves and some bacterial isolates support plant growth at low temperatures. Sci Rep 12:18839. https://doi.org/10.1038/s41598-022-23582-2.
2. Mitter B, Pfaffenbichler N, Flavell R, Compant S, Antonielli L, Petric A, Berninger T, Naveed M, Sheibani-Tezerji R, Maltzahn G von, Sessitsch A. 2017. A new approach to modify plant microbiomes and traits by introducing beneficial bacteria at flowering into progeny seeds. Front Microbiol 8:11. https://doi.org/10.3389/fmicb.2017.00011.
3. Chelius MK, Triplett EW. 2001. The Diversity of Archaea and Bacteria in Association with the Roots of *Zea mays* L. Microb Ecol 41:252–263. https://doi.org/10.1007/s002480000087.
4. Langmead B, Salzberg SL. 2012. Fast gapped-read alignment with Bowtie 2. Nat Methods 9:357–359. https://doi.org/10.1038/nmeth.1923.
5. Martin M. 2011. Cutadapt removes adapter sequences from high-throughput sequencing reads. EMBnet J 17:10–12. https://doi.org/10.14806/ej.17.1.200
6. Callahan BJ, McMurdie PJ, Rosen MJ, Han AW, Johnson AJA, Holmes SP. 2016. DADA2: High-resolution sample inference from Illumina amplicon data. Nat Methods 13:581–583. https://doi.org/10.1038/nmeth.3869.
7. Bengtsson-Palme J, Thorell K, Wurzbacher C, Sjöling Å, Nilsson RH. 2016. Metaxa2 Diversity Tools: Easing microbial community analysis with Metaxa2. Ecol Inform 33:45–50. https://doi.org/10.1016/j.ecoinf.2016.04.004.
8. Quast C, Pruesse E, Yilmaz P, Gerken J, Schweer T, Yarza P, Peplies J, Glöckner FO. 2013. The SILVA ribosomal RNA gene database project: Improved data processing and web-based tools. Nucleic Acids Res 41:590–596. https://doi.org/10.1093/nar/gks1219.
9. R Core Team. 2023. R: A language and environment for statistical computing. R Foundation for Statistical Computing, Vienna, Austria. https://www.R-project.org/.
10. Saary P, Forslund K, Bork P, Hildebrand F. 2017. RTK: Efficient rarefaction analysis of large 972 datasets. Bioinformatics 33: 2594–2595. https://doi.org/10.1093/bioinformatics/btx206.
11. Kuhn M. 2008. Building Predictive Models in R Using the caret Package. J Stat Softw 28: 1–26. https://doi.org/10.18637/jss.v028.i05.
12. Lenth RV. 2023. emmeans: Estimated Marginal Means, aka Least-Squares Means. R package version 1.8.9. https://cran.r-project.org/web/packages/emmeans/index.html.
13. Hothorn T, Zeileis A. 2021. Predictive Distribution Modeling Using Transformation Forests. J Comput Graph Stat 30:1181–1196. https://doi.org/10.1080/10618600.2021.1872581.
14. Oksanen J, Simpson G, Blanchet F, Kindt R, Legendre P, Minchin P, O'Hara R, Solymos P, Stevens M, Szoecs E., Wagner H, Barbour M, Bedward M, Bolker B, Borcard D, Carvalho G, Chirico M, De Caceres M, Durand S, Evangelista HBA, FitzJohn R, Friendly M, Furneaux B, Hannigan G, Hill MO, Lahti L, McGlinn D, Ouellette MH, Cunha ER, Smith T, Stier A, Braak CJFT, Weedon J. 2023. vegan: Community Ecology Package. R package version 2.6-4, https://cran.r-project.org/web/packages/vegan/index.html.
15. Herve M. 2023. RVAideMemoire: Testing and Plotting Procedures for Biostatistics. R package version 0.9-83, https://CRAN.R-project.org/package = RVAideMemoire

16. Xiong C, Zhu YG, Wang JT, Singh B, Han LL, Shen JP, Li PP, Wang GB, Wu CF, Ge AH, et al. 2021. Host selection shapes crop microbiome assembly and network complexity. New Phytol 229:1091–1104. https://doi.org/10.1111/nph.16890.
17. Wang Y, Naumann U, Wright ST, Warton DI. 2012. Mvabund- an R package for model-based analysis of multivariate abundance data. Methods Ecol Evol 3:471–474. https://doi.org/10.1111/j.2041-210X.2012.00190.x.
18. Warton DI, Wright ST, Wang Y. 2012. Distance-based multivariate analyses confound location and dispersion effects. Methods Ecol Evol 3:89–101. https://doi.org/10.1111/j.2041-210X.2011.00127.x.
19. Bálint M, Bartha L, O'Hara RB, Olson MS, Otte J, Pfenninger M, Robertson AL, Tiffin P, Schmitt I. 2015. Relocation, high-latitude warming and host genetic identity shape the foliar fungal microbiome of poplars. Mol Ecol 24:235–248. https://doi.org/10.1111/mec.13018.
20. Niku J, Hui FKC, Taskinen S, Warton DI. 2019. Gllvm: Fast analysis of multivariate abundance data with generalized linear latent variable models in r. Methods Ecol Evol 10: 2173–2182. https://doi.org/10.1111/2041-210X.13303
21. Maechler M, Rousseeuw P, Struyf A, Hubert M, Hornik K. 2022. cluster: Cluster Analysis Basics and Extensions. R package version 2.1.4 — For new features, see the 'Changelog' file (in the package source), https://CRAN.R-project.org/package = cluster.
22. Trivedi P, Leach JE, Tringe SG, Sa T, Singh BK. 2020. Plant–microbiome interactions: from community assembly to plant health. Nat Rev Microbiol 18:607–621. https://doi.org/10.1038/s41579-020-0412-1.
23. Shade A, Stopnisek N. 2019. Abundance-occupancy distributions to prioritize plant core microbiome membership. Curr Opin Microbiol 49:50–58. https://doi.org/10.1016/j.mib.2019.09.008.
24. Stopnisek N, Shade A. 2021. Persistent microbiome members in the common bean rhizosphere: an integrated analysis of space, time, and plant genotype. ISME J 15:2708–2722. https://doi.org/10.1038/s41396-021-00955-5.
25. Ning D, Deng Y, Tiedje JM, Zhou J. 2019. A general framework for quantitatively assessing ecological stochasticity. Proc Natl Acad Sci USA 116:16892–16898. https://doi.org/10.1073/pnas.1904623116.
26. Ning D, Yuan M, Wu L, Zhang Y, Guo X, Zhou X, Yang Y, Arkin AP, Firestone MK, Zhou J. 2020. A quantitative framework reveals ecological drivers of grassland microbial community assembly in response to warming. Nat Commun 11:4717. https://doi.org/10.1038/s41467-020-18560-z.
27. Bodenhofer U, Bonatesta E, Horejs-Kainrath C, Hochreiter S. 2015. "msa: an R package for multiple sequence alignment." Bioinform 31:3997–3999.
28. Charif D, Lobry JR. 2007. SeqinR 1.0-2: a contributed package to the R project for statistical computing devoted to biological sequences retrieval and analysis p 207–232. In: Bastolla U, Porto M, Roman HE, Vendruscolo M. (eds), Structural Approaches to Sequence Evolution. Biological and Medical Physics, Biomedical Engineering. Springer Berlin Heidelberg. https://doi.org/10.1007/978-3-540-35306-5_10.
29. Lin H, Peddada SD. 2020. Analysis of compositions of microbiomes with bias correction. Nat Commun 11:3514. https://doi.org/10.1038/s41467-020-17041-7.
30. Olofintila OE, Noel ZA. 2023. Soybean and cotton spermosphere soil microbiome shows dominance of soilborne copiotrophs. Microbiol Spectr 11:e00377-23 https://doi.org/10.1128/spectrum.00377-23.
31. Peschel S, Müller CL, Von Mutius E, Boulesteix AL, Depner M. 2021. NetCoMi: Network construction and comparison for microbiome data in R. Brief Bioinform 22:bbaa290. https://doi.org/10.1093/bib/bbaa290.

32. Banerjee S, Schlaeppi K, van der Heijden MGA. 2018. Keystone taxa as drivers of microbiome structure and functioning. Nat Rev Microbiol 16:567–576. https://doi.org/10.1038/s41579-018-0024-1.
33. Kurtz ZD, Müller CL, Miraldi ER, Littman DR, Blaser MJ, Bonneau RA. 2015. Sparse and Compositionally Robust Inference of Microbial Ecological Networks. PLoS Comput Biol 11:1–25. https://doi.org/10.1371/journal.pcbi.1004226.
34. Nissinen RM, Männistö MK, van Elsas JD. 2012. Endophytic bacterial communities in three arctic plants from low arctic fell tundra are cold-adapted and host-plant specific.
35. Arrigoni E, Antonielli L, Pindo M, Pertot I, Perazzolli M. 2018. Tissue age and plant genotype affect the microbiota of apple and pear bark. Microbiol Res 211:57–68. https://doi.org/10.1016/j.micres.2018.04.002.
36. Hahsler M, Nagar A. 2019. rBLAST: R Interface for the Basic Local Alignment Search Tool. R package version 0.99.2, https://github.com/mhahsler/rBLAST.
37. Sherrill-Mix S. 2023. taxonomizr: Functions to Work with NCBI Accessions and Taxonomy. R package version 0.10.2, https://cran.r-project.org/web/packages/taxonomizr/index.html
38. Persyn A, Garcia Mendez S, Beirinckx S, De Meyer S, Willems A, De Tender C, Goormachtig S. 2022. Digging into the lettuce cold-specific root microbiome in search of chilling stress tolerance-conferring plant growth-promoting bacteria. Phytobiomes J 1–73. https://doi.org/10.1094/PBIOMES-07-22-0044-MF.
39. Ito Y, Maruo T, Ishikawa M, Shinohara Y. 2011. Effects of scarification with sulfuric acid and matric priming on seed germination of seed propagation type of F1 hybrid strawberry (*Fragaria* × *ananassa* Duch.). J Japanese Soc Hortic Sci 80: 32–37. https://doi.org/10.2503/jjshs1.80.32.
40. Vogel CM, Potthoff DB, Schäfer M, Barandun N, Vorholt JA. 2021. Protective role of the Arabidopsis leaf microbiota against a bacterial pathogen. Nat Microbiol 6:1537–1548. https://doi.org/10.1038/s41564-021-00997-7.
41. Proebsting EL, Gross DC. 1988. Field Evaluations of Frost Injury to Deciduous Fruit Trees as Influenced by Ice Nucleation-active *Pseudomonas syringae*. J Am Soc Hortic Sci 113:498–506. https://doi.org/10.21273/JASHS.113.4.498.
42. Jiang W, Pan R, Wu C, Xu L, Abdelaziz ME, Oelmüller R, Zhang W. 2020. *Piriformospora indica* enhances freezing tolerance and post-thaw recovery in Arabidopsis by stimulating the expression of CBF genes. Plant Signal Behav 15:1745472. https://doi.org/10.1080/15592324.2020.1745472.
43. Galambos N, Compant S, Moretto M, Sicher C, Puopolo G, Wäckers F, Sessitsch A, Pertot I, Perazzolli M. 2020. Humic Acid Enhances the Growth of Tomato Promoted by Endophytic Bacterial Strains Through the Activation of Hormone-, Growth-, and Transcription-Related Processes. Front Plant Sci 11:582267. https://doi.org/10.3389/fpls.2020.582267.
44. Signorell, A. 2023. Desctools: Tools for descriptive statistics. R package version 0.99.50. https://cran.r-project.org/web/packages/DescTools/index.html.
45. Kassambara A. 2023. rstatix: Pipe-Friendly Framework for Basic Statistical Tests. R package version 0.7.2. https://cran.r-project.org/web/packages/rstatix/index.html.
