# Supplementary material for: Amplicon sequencing and culture-dependent approaches reveal core bacterial endophytes aiding freezing stress tolerance in alpine Rosaceae plants
Source: mBio. 2025 Feb 25;16(4):e01418-24. doi: 10.1128/mbio.01418-24 (PMC11980557; doi:10.1128/mbio.01418-24)
Supplement: Supplemental figures — Fig. S1 to S10. [file mbio.01418-24-s0002.pdf]

Supplementary figures

Amplicon sequencing and culture-dependent approaches reveal core bacterial endophytes aiding freezing stress tolerance in alpine Rosaceae plants

Malek Marian,[a#], Livio Antonielli,[b] Ilaria Pertot,[a,c] Michele Perazzolli[a,c]

[a]Center for Agriculture Food Environment (C3A), University of Trento, San Michele all'Adige, Italy

[b]Department of Health and Environment, Bioresources Unit, AIT Austrian Institute of Technology, Tulln an der Donau, Austria

[c]Research and Innovation Centre, Fondazione Edmund Mach, San Michele all'Adige, Italy

Running Head: Endophytic Bacterial Communities of Alpine Plants

#Address correspondence to: Malek Marian, malekmarian@hotmail.com

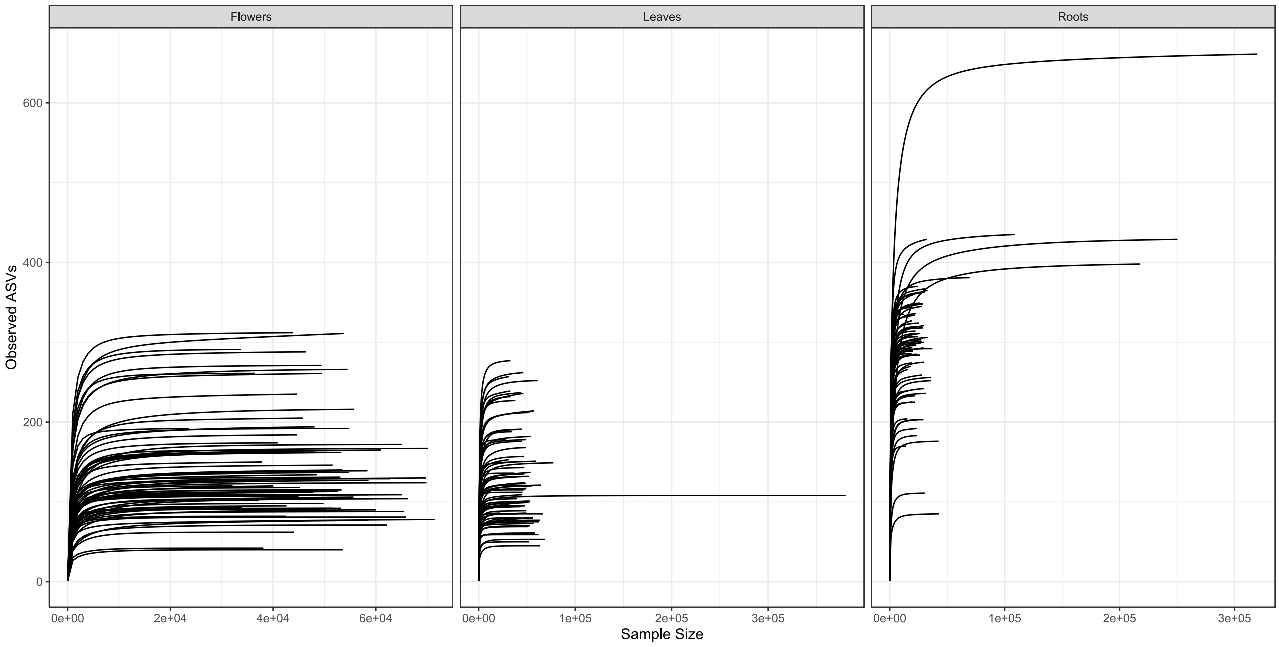

FIG S1 Rarefaction curves of endophytic bacterial communities associated with alpine Rosaceae plants. Curves were generated using the rarecurve function with 100 step size from the vegan R package. A total 270 samples; 108 from *Alchemilla* sp., 54 from *Dryas octopetala*, and 108 from *Geum montanum* from flower, leaf, and root tissues from seven collection sites and two exposures were analyzed.

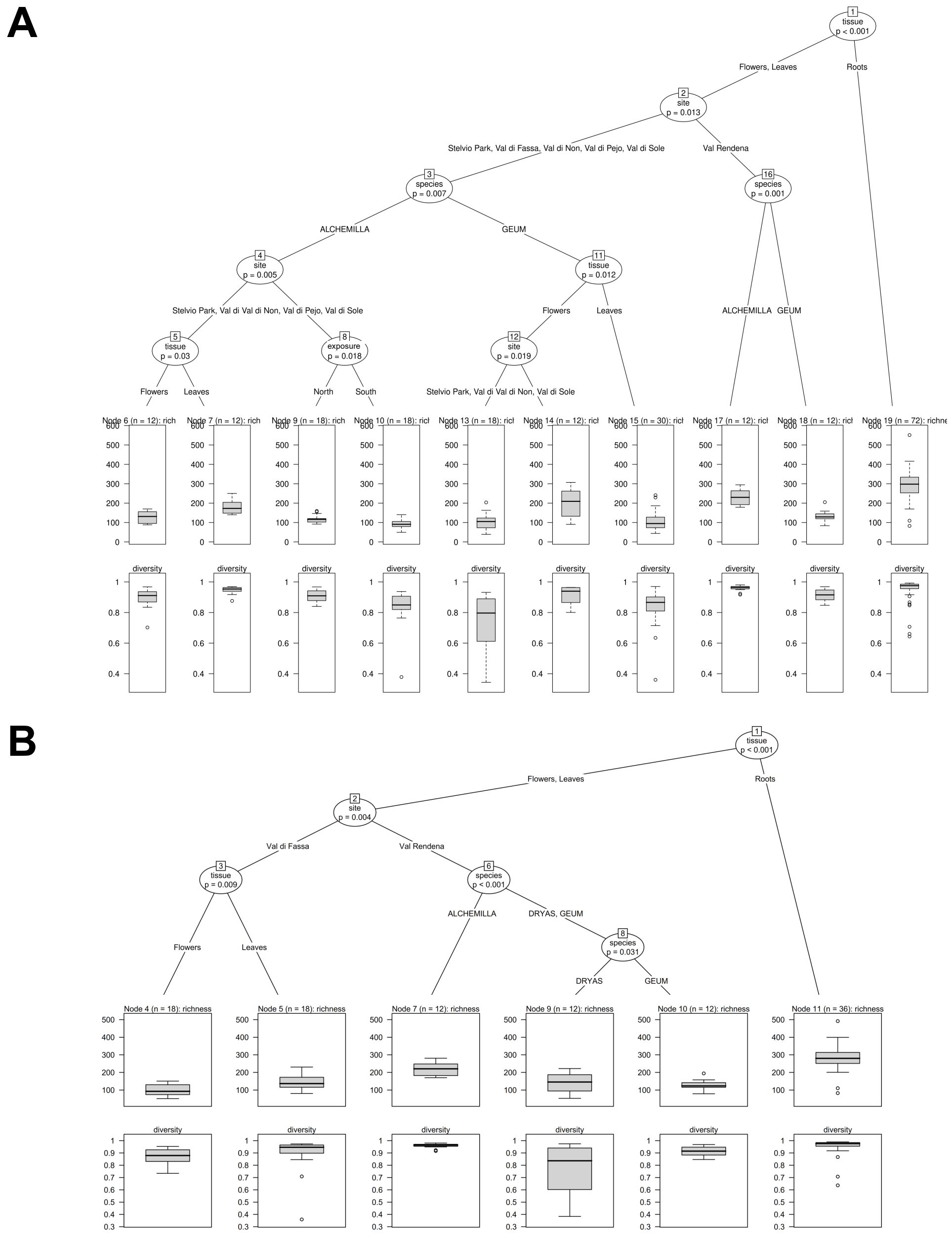

FIG S2 Conditional inference regression tree of bacterial alpha-diversity. Conditional inference regression trees were fitted onto the richness (observed ASVs) and diversity (Simpson's index) values from the first dataset (**A**; *Alchemilla* sp. and *Geum montanum* from six collection sites) and the second dataset (**B**; *Alchemilla* sp., *Dryas octopetala,* and *G. montanum* from two collection sites) of samples to test the global null hypothesis of independence for covariate variables (i.e., plant tissue, alpine Rosaceae plants, collection site, and exposure), using the ctree function from partykit v1.2 R package. Each node (circles) represents the variable that was split and gives p-values (determined using Bonferroni corrected p-values from permutation tests) associated with the split. Factors that were split for each variable are shown below each circle. Terminal nodes at the bottom of each tree include the mean alpha-diversity metrics of richness (top boxes) and diversity (bottom boxes) grouped within the splitting criteria.

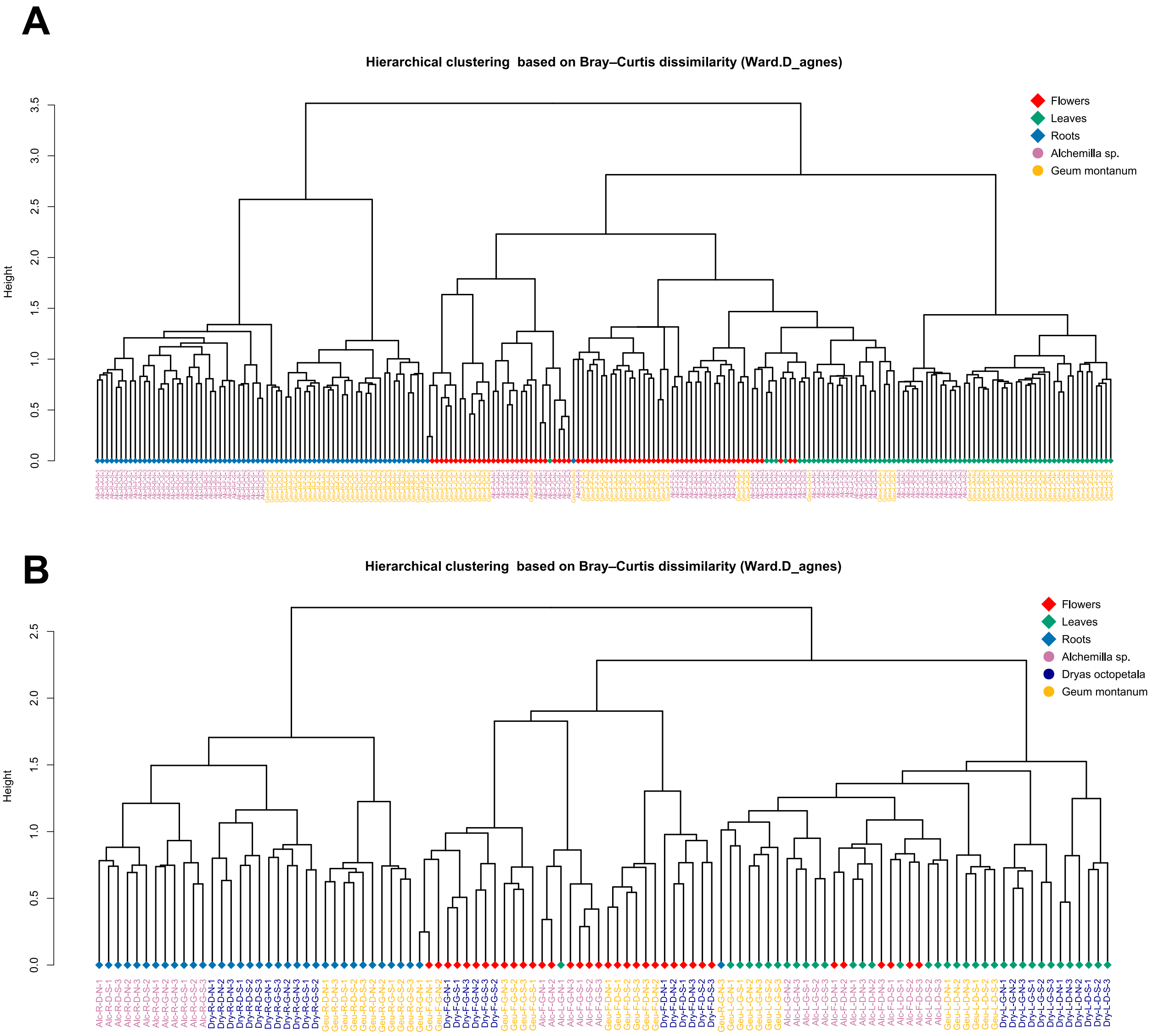

FIG S3 Hierarchical clustering of alpine Rosaceae plants samples. Hierarchical clustering of the first dataset (**A**; *Alchemilla* sp. and *Geum montanum* from six collection sites) and the second dataset (**B**; *Alchemilla* sp., *Dryas octopetala,* and *G. montanum* from two collection sites) of samples was based on Bray–Curtis dissimilarity matrices obtained from rarefied and Wisconsin-double standardized count data. Hierarchical clusters were constructed using agnes function with Ward.D method (identified as the strongest clustering structure by agglomerative coefficient test).

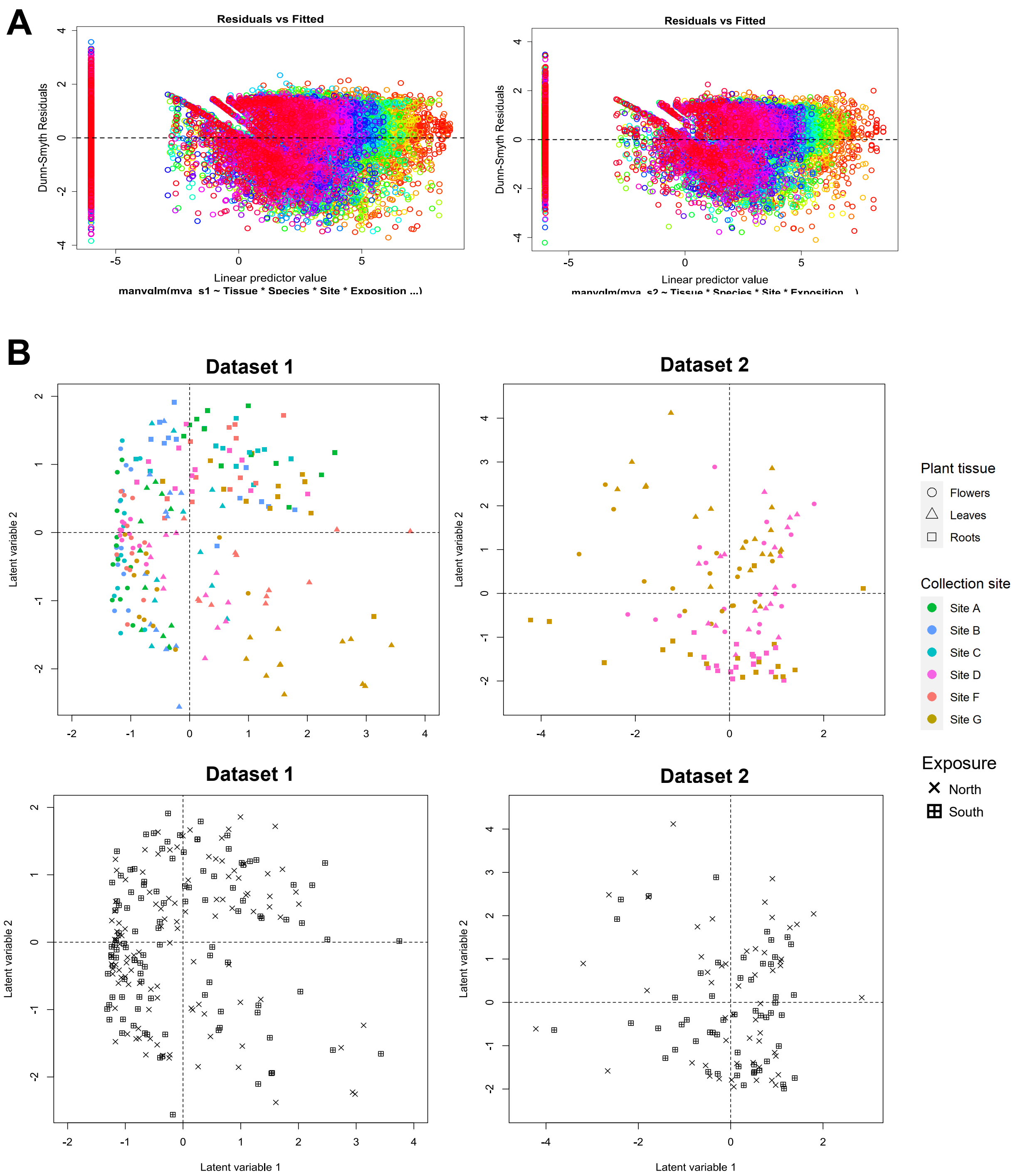

FIG S4 Multivariate generalized linear model (mGLM) analysis on endophytic bacterial community structure of alpine Rosaceae plants. (**A**) The residual plot shows the negative binomial distribution of the data and the ordination biplot displays the endophytic bacterial community structure of Rosaceae plants. (**B**) A model-based ordination with Gaussian copulas to visualize the GLM results (obtained through mvabund package) were computed using the ordiplot function from the gllvm R package. The first dataset (left panels; *Alchemilla* sp. and *Geum montanum* from six collection sites) and the second dataset (right panels; *Alchemilla* sp., *Dryas octopetala,* and *G. montanum* from two collection sites). Only ASVs with an occupancy of 0.25 were tested (131 ASVs and 161 ASVs for dataset 1 and dataset 2, respectively). Different shapes and shape colours show the different plant tissues and collection sites, respectively.

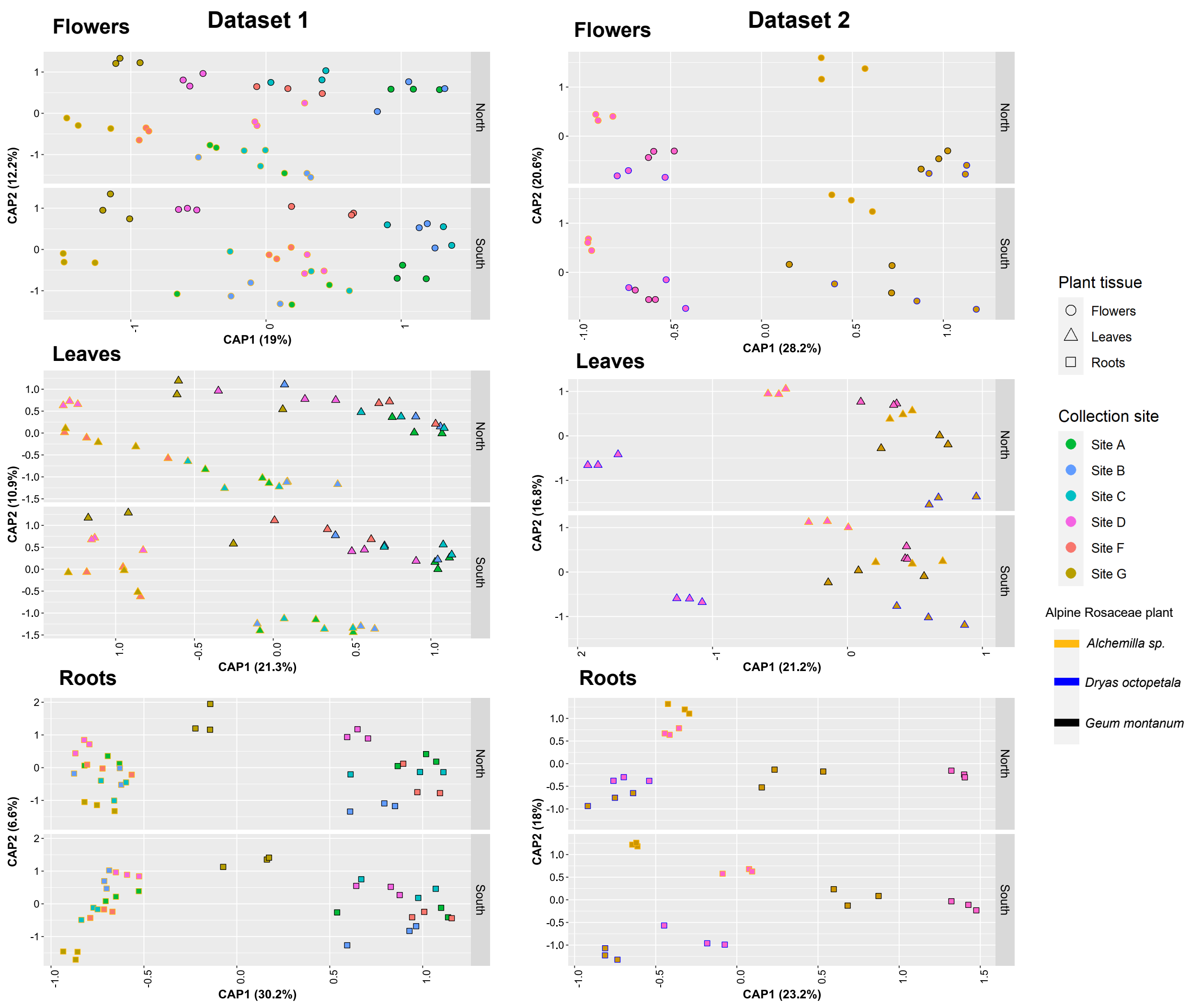

FIG S5 Constrained analysis of principle (CAP) coordinates based on Bray–Curtis dissimilarity matrices in each tissue of different alpine Rosaceae plants, collection sites, and exposures. The first dataset (left panels; *Alchemilla* sp. and *Geum montanum* from six collection sites) and the second dataset (right panels; *Alchemilla* sp., *Dryas octopetala,* and *G. montanum* from two collection sites) are shown. Different symbols, fill, and outline colours represent different plant tissue, alpine Rosaceae plants, and collection sites, respectively.

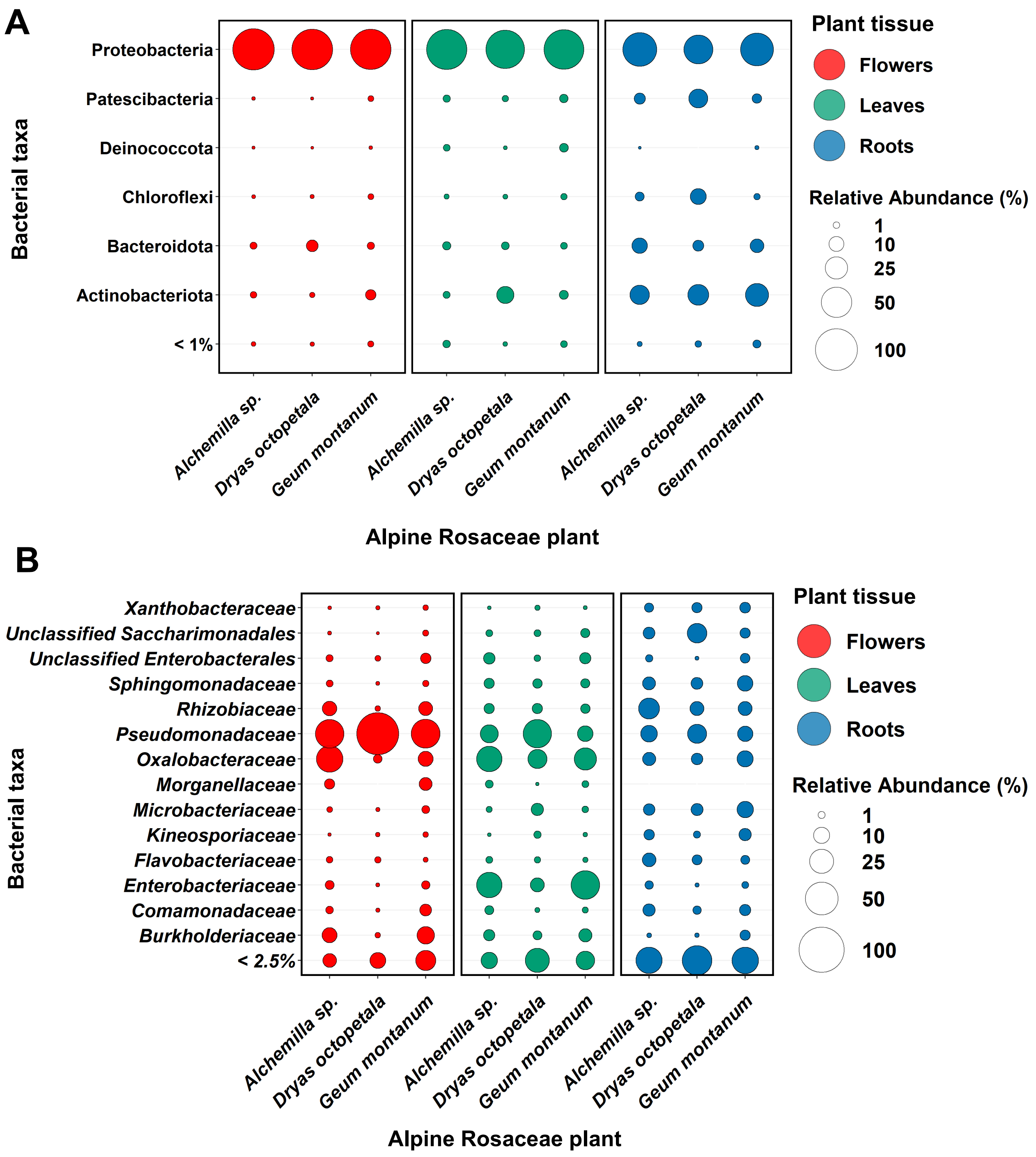

FIG S6 Taxonomic summaries of the endophytic bacterial community structure of alpine Rosaceae plants. Bubble plots of bacterial community classification at the (**A**) phylum level and (**B**) family level in different plant tissues and alpine Rosaceae plants across all collection sites and exposures. Only phyla and families with > 1% and > 2.5% mean relative abundance are shown, respectively. Different colours represent different tissues while bubble sizes represent mean relative abundance.

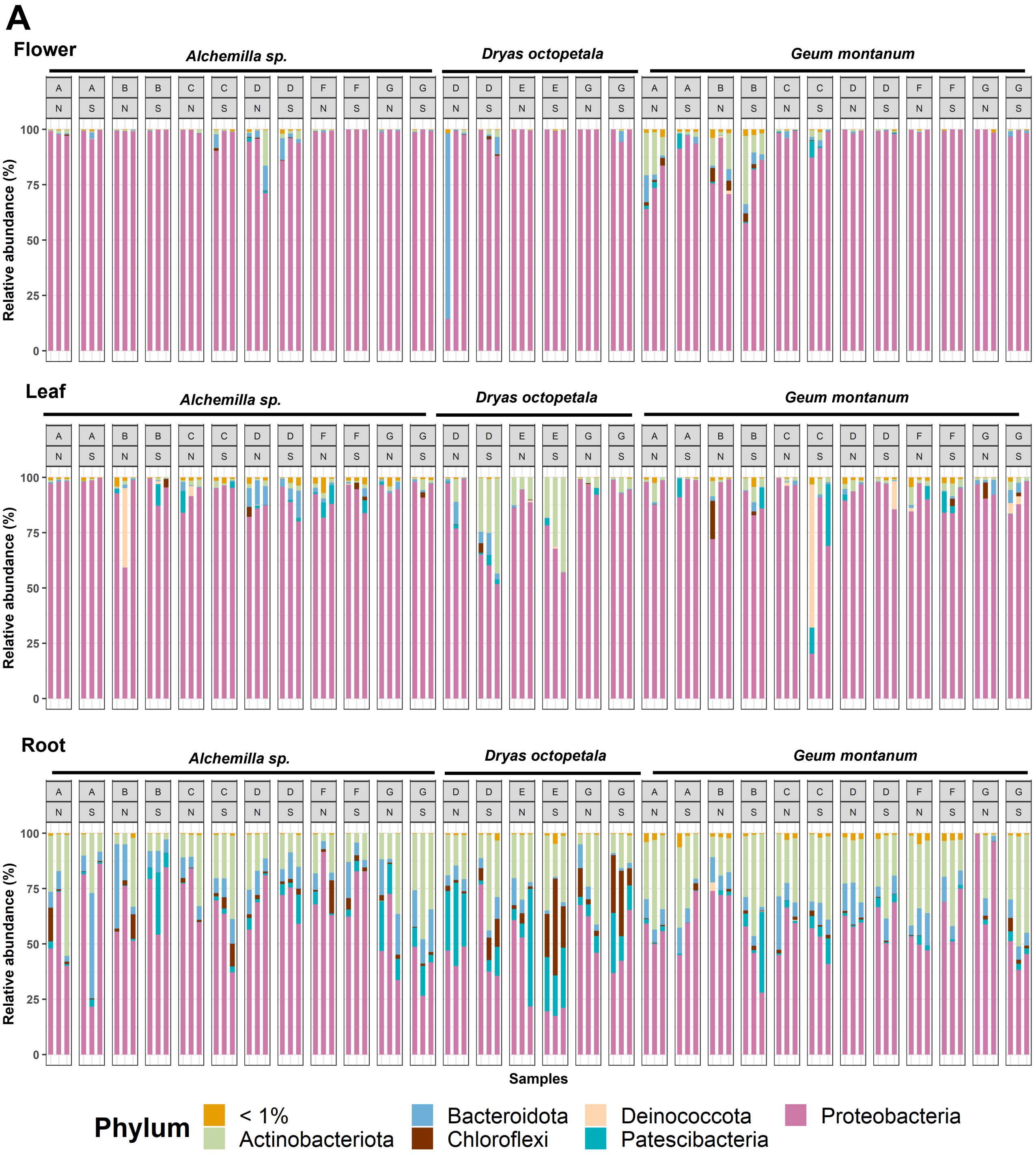
A
Flower
Leaf
Root
Alchemilla sp.
Dryas octopetala
Geum montanum
Relative abundance (%)
0
25
50
75
100
Samples
Phylum
< 1%
Actinobacteriota
Bacteroidota
Chloroflexi
Deinococcota
Patescibacteria
Proteobacteria

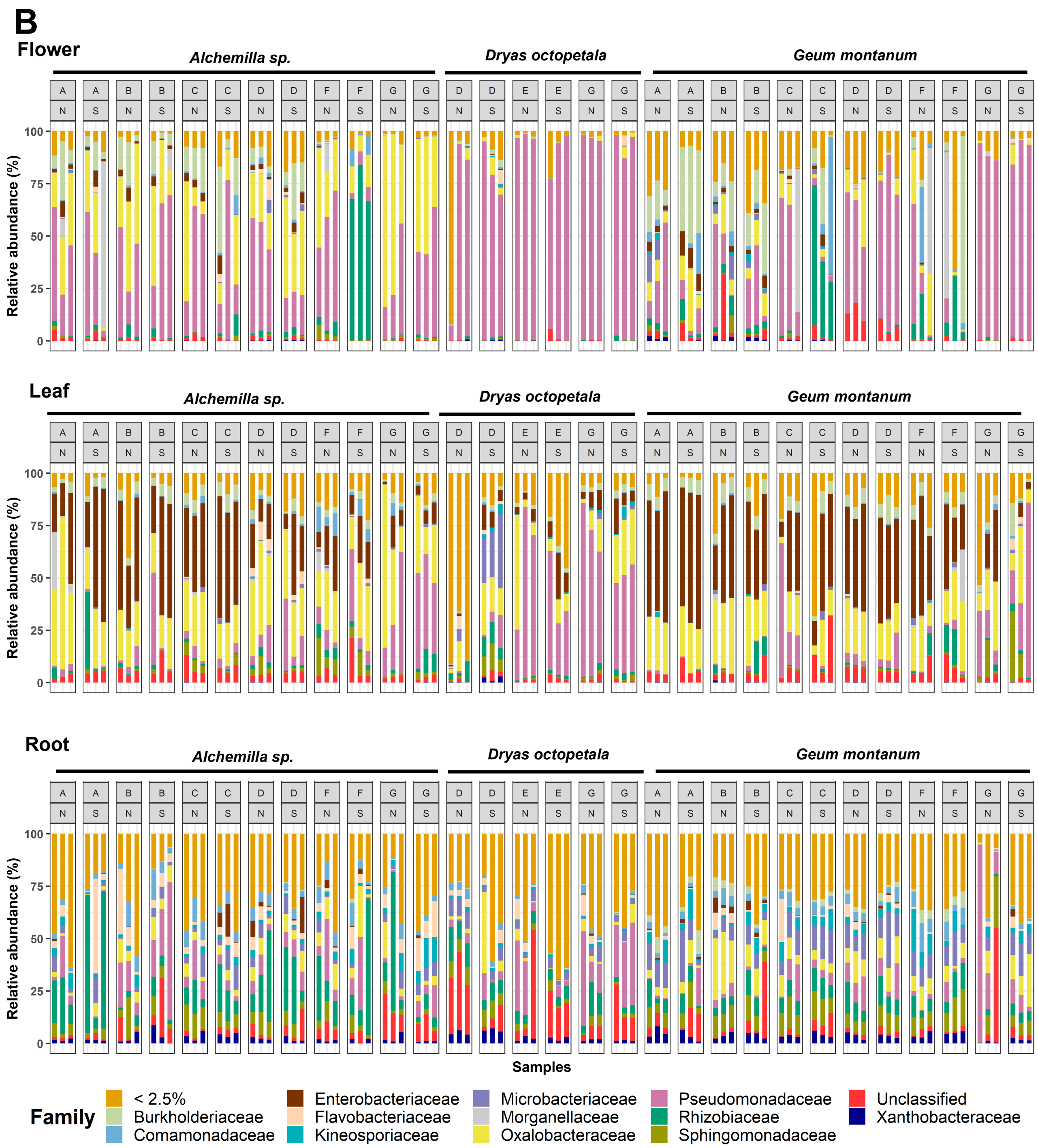

B
Flower
Leaf
Root
Alchemilla sp.
Dryas octopetala
Geum montanum
Relative abundance (%)
Samples
Family
< 2.5%
Burkholderiaceae
Comamonadaceae
Enterobacteriaceae
Flavobacteriaceae
Kineosporiaceae
Microbacteriaceae
Morganellaceae
Oxalobacteraceae
Pseudomonadaceae
Rhizobiaceae
Sphingomonadaceae
Unclassified
Xanthobacteraceae

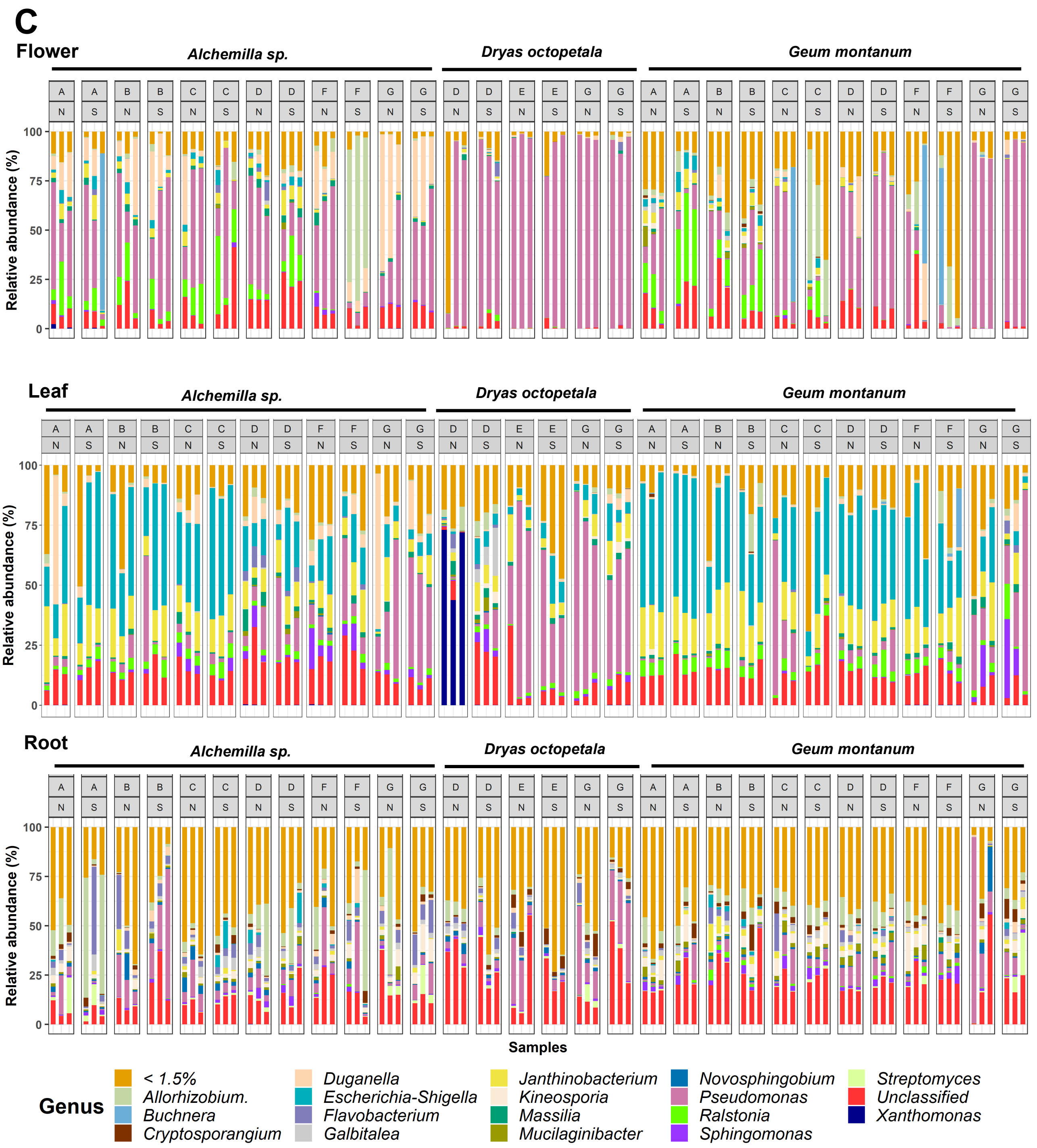

FIG S7 Relative abundance of the endophytic bacterial communities of alpine Rosaceae plants. Bar plots of relative abundance of bacterial communities of *Alchemilla* sp., *Dryas octopetala,* and *Geum montanum* at (**A**) phylum-level, (**B**) family-level, and (**C**) genus-level. Bar plots reports data of flowers, leaves, and roots for each collection site (Val di Non, Val di Sole, Val di Pejo, Val Rendena, South Tyrol, Stelvio Park, and Val di Fassa; A, B, C, D, E, F, and G, respectively) and exposure (N and S) indicated in the grey boxes. Only phyla, families, and genera with > 1%, > 2.5%, and > 1.5% relative abundance are shown, respectively.

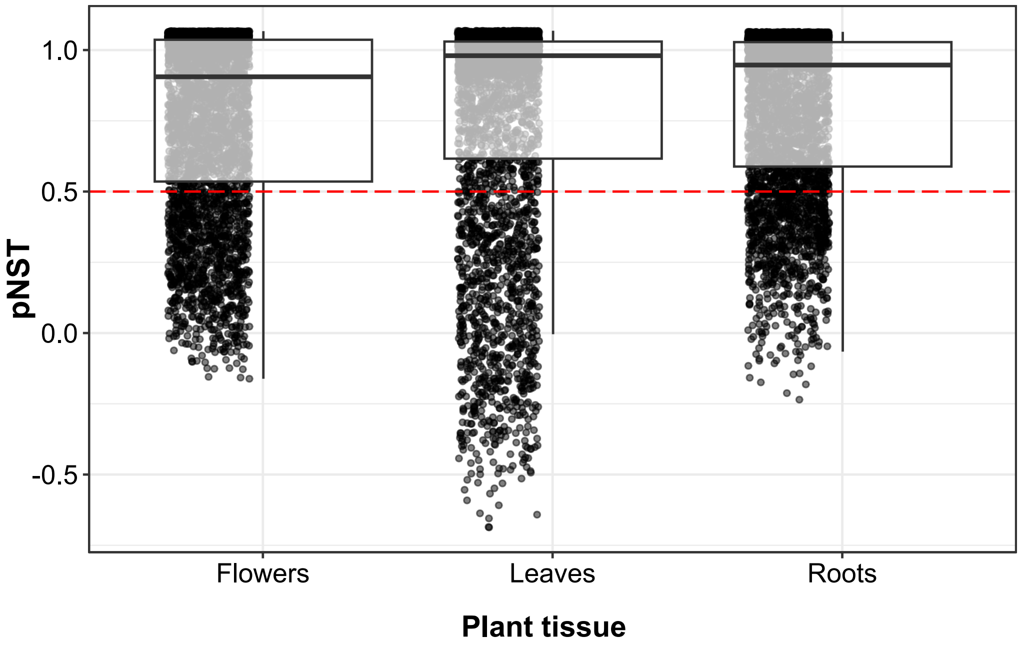

FIG S8 Phylogenetic Normalized Stochasticity Ratios (pNST) for determinism and stochasticity of endophytic bacterial community aggregation in different tissues of alpine Rosaceae plants. The red line indicates the boundary between more deterministic assembly (< 0.5) and more stochastic assembly (> 0.5). Black and grey dots represent each amplicon sequence variant and white squares indicate the median values of 0.75–0.79 in flower, leaf, and root tissues.

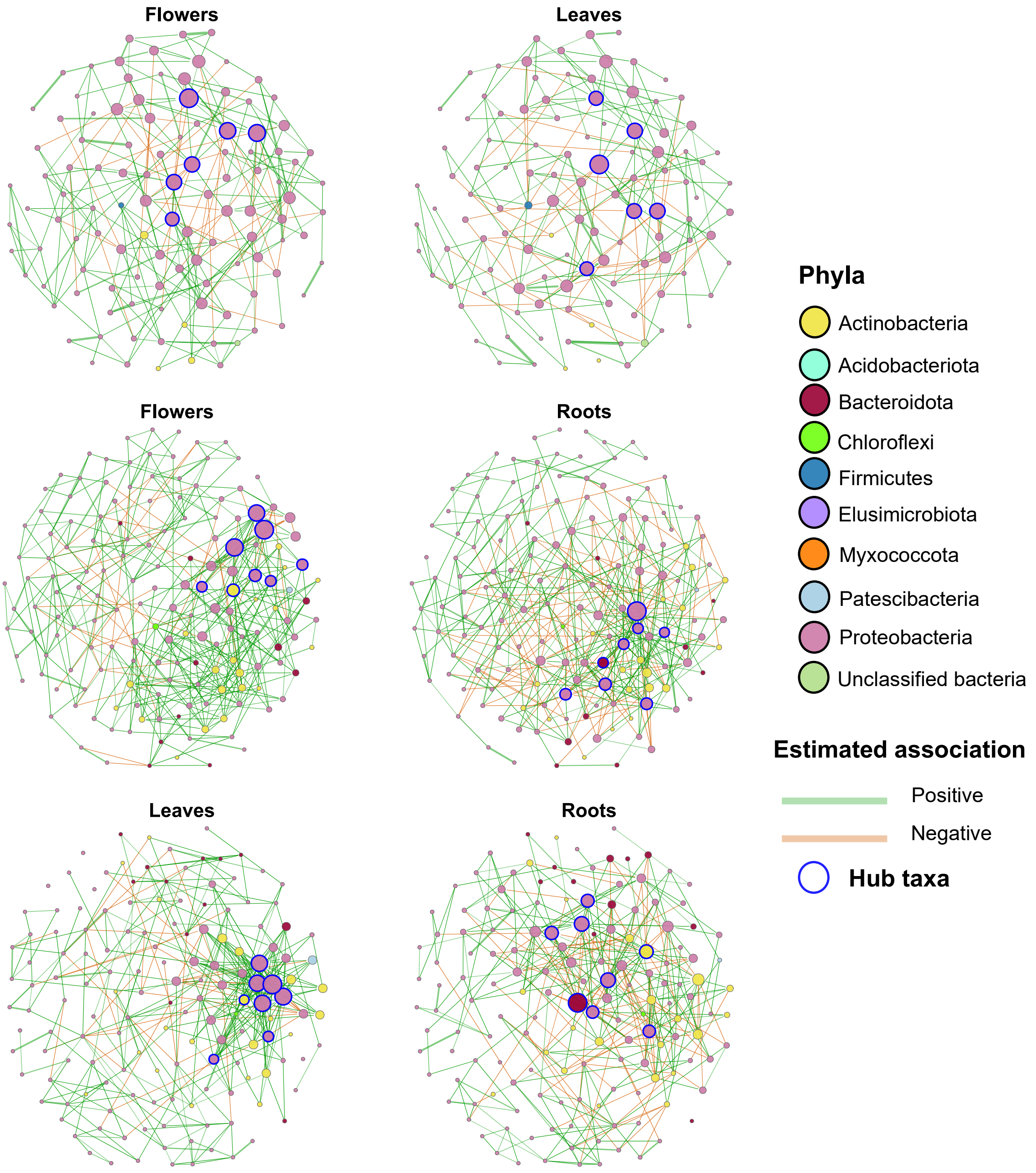

FIG S9 Co-occurrence network comparisons of endophytic bacterial communities alpine Rosaceae plants. Network comparison between (**A**) flower and root, (**B**) flower and leaf, and (**C**) leaf and root tissues. ASVs with a relative abundance greater than 0.001% and occupancy greater than 0.25 were used for network analysis using the SpiecEasi and NetCoMi R packages. Comparative analysis of networks was achieved between the similarity of most central nodes (i.e., set of ASVs with eigenvector centrality values greater than 95% of the empirical distribution of all eigenvector centralities in the network). Each node corresponds to an ASV, and edges between nodes correspond to either positive (green) or negative (orange) correlations. The thickness of each edge is proportional to the correlation coefficients of the connections. ASVs belonging to different bacterial phyla have distinct colour codes. Node size reflects their eigenvector centrality while nodes with thick blue outlines represent hub taxa.

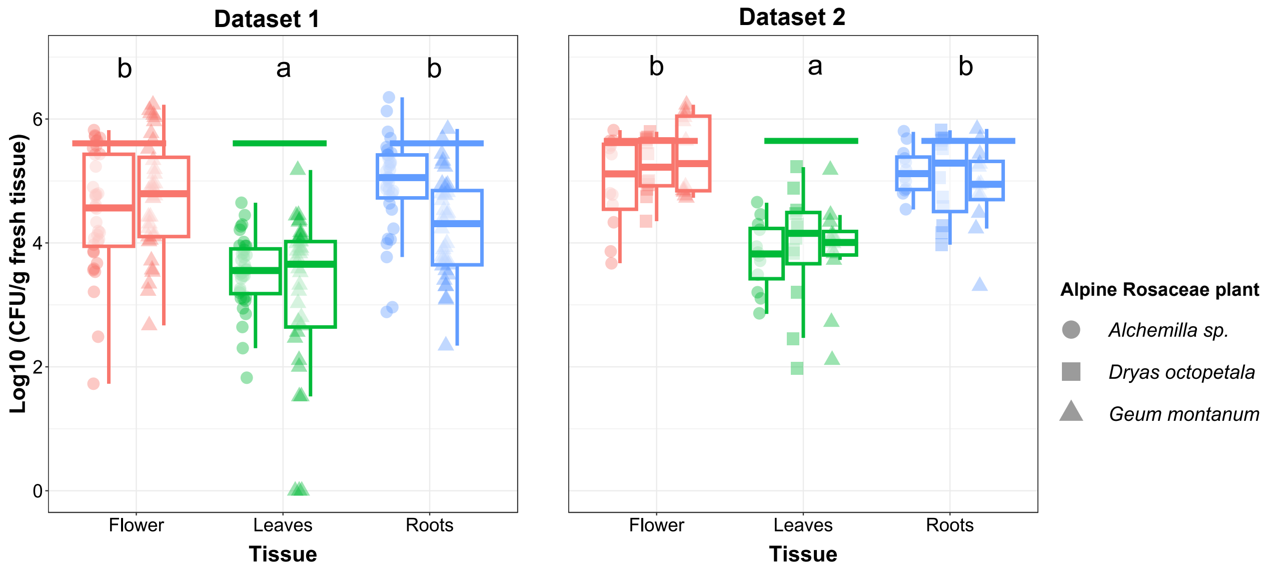

FIG S10 The abundance of culturable psychrotolerant bacterial endophytes in alpine Rosaceae plants. Box plots represent log10 colony forming unit (CFU) per gram of fresh weight of flower, leaf, and root tissues in the first dataset (*Alchemilla* sp. and *Geum montanum* from six collection sites) and the second dataset (*Alchemilla* sp., *Dryas octopetala,* and *G. montanum* from two collection sites). Values were obtained from three replicates. Different letters indicate significant differences, according to ANOVA followed by post-hoc analysis with estimated marginal mean comparisons ($P \leq 0.05$).
