# Supplementary material for: Amplicon sequencing and culture-dependent approaches reveal core bacterial endophytes aiding freezing stress tolerance in alpine Rosaceae plants
Source: mBio. 2025 Feb 25;16(4):e01418-24. doi: 10.1128/mbio.01418-24 (PMC11980557; doi:10.1128/mbio.01418-24)
Supplement: Supplemental tables — Tables S1, S2, S4 to S13, S15, and S17. [file mbio.01418-24-s0003.pdf]

**Supplementary tables**

# Amplicon sequencing and culture-dependent approaches reveal core bacterial endophytes aiding freezing stress tolerance in alpine Rosaceae plants

Malek Marian,[a]#, Livio Antonielli,[b] Ilaria Pertot,[a,c] Michele Perazzolli[a,c]

[a]Center for Agriculture Food Environment (C3A), University of Trento, San Michele all'Adige, Italy

[b]Department of Health and Environment, Bioresources Unit, AIT Austrian Institute of Technology, Tulln an der Donau, Austria

[c]Research and Innovation Centre, Fondazione Edmund Mach, San Michele all'Adige, Italy

Running Head: Endophytic Bacterial Communities of Alpine Plants

#Address correspondence to: Malek Marian, malekmarian@hotmail.com

**Table S1.** Summary of collection site and sample description of alpine Rocaseae plants.

| Sample code | Rocaseae plant | Tissue | Site | Site code | Exposure | Coordinates | Altitude (meters above sea level) | Temperature (°C) | Collection date |
|---|---|---|---|---|---|---|---|---|---|
| Alc-F-A-N-1 | *Alchemilla* sp. | Flowers | Val di Non | A | North | 46.43236880107553, 10.887484371878964 | 1950 | 14 | 20-06-21 |
| Alc-F-A-N-2 | *Alchemilla* sp. | Flowers | Val di Non | | North | 46.43236880107553, 10.887484371878964 | 1950 | 14 | 20-06-21 |
| Alc-F-A-N-3 | *Alchemilla* sp. | Flowers | Val di Non | | North | 46.43236880107553, 10.887484371878964 | 1950 | 14 | 20-06-21 |
| Alc-F-A-S-1 | *Alchemilla* sp. | Flowers | Val di Non | | South | 46.43236880107553, 10.887484371878964 | 1950 | 14 | 20-06-21 |
| Alc-F-A-S-2 | *Alchemilla* sp. | Flowers | Val di Non | | South | 46.43236880107553, 10.887484371878964 | 1950 | 14 | 20-06-21 |
| Alc-F-A-S-3 | *Alchemilla* sp. | Flowers | Val di Non | | South | 46.43236880107553, 10.887484371878964 | 1950 | 14 | 20-06-21 |
| Geu-F-A-N-1 | *Geum montanum* | Flowers | Val di Non | | North | 46.43140936845373, 10.874719416962568 | 2100 | 12 | 20-06-21 |
| Geu-F-A-N-2 | *Geum montanum* | Flowers | Val di Non | | North | 46.43140936845373, 10.874719416962568 | 2100 | 12 | 20-06-21 |
| Geu-F-A-N-3 | *Geum montanum* | Flowers | Val di Non | | North | 46.43140936845373, 10.874719416962568 | 2100 | 12 | 20-06-21 |
| Geu-F-A-S-1 | *Geum montanum* | Flowers | Val di Non | | South | 46.43140936845373, 10.874719416962568 | 2100 | 12 | 20-06-21 |
| Geu-F-A-S-2 | *Geum montanum* | Flowers | Val di Non | | South | 46.43140936845373, 10.874719416962568 | 2100 | 12 | 20-06-21 |
| Geu-F-A-S-3 | *Geum montanum* | Flowers | Val di Non | | South | 46.43140936845373, 10.874719416962568 | 2100 | 12 | 20-06-21 |
| Alc-F-B-N-1 | *Alchemilla* sp. | Flowers | Val di Sole | B | North | 46.281873339437034, 10.809519461015759 | 1800 | 14 | 27-06-21 |
| Alc-F-B-N-2 | *Alchemilla* sp. | Flowers | Val di Sole | | North | 46.281873339437034, 10.809519461015759 | 1800 | 14 | 27-06-21 |
| Alc-F-B-N-3 | *Alchemilla* sp. | Flowers | Val di Sole | | North | 46.281873339437034, 10.809519461015759 | 1800 | 14 | 27-06-21 |
| Alc-F-B-S-1 | *Alchemilla* sp. | Flowers | Val di Sole | | South | 46.281873339437034, 10.809519461015759 | 1800 | 14 | 27-06-21 |
| Alc-F-B-S-2 | *Alchemilla* sp. | Flowers | Val di Sole | | South | 46.281873339437034, 10.809519461015759 | 1800 | 14 | 27-06-21 |
| Alc-F-B-S-3 | *Alchemilla* sp. | Flowers | Val di Sole | | South | 46.281873339437034, 10.809519461015759 | 1800 | 14 | 27-06-21 |
| Geu-F-B-N-1 | *Geum montanum* | Flowers | Val di Sole | | North | 46.24360124596042, 10.653712543817255 | 2300 | 12 | 26-06-21 |
| Geu-F-B-N-2 | *Geum montanum* | Flowers | Val di Sole | | North | 46.24360124596042, 10.653712543817255 | 2300 | 12 | 26-06-21 |
| Geu-F-B-N-3 | *Geum montanum* | Flowers | Val di Sole | | North | 46.24360124596042, 10.653712543817255 | 2300 | 12 | 26-06-21 |
| Geu-F-B-S-1 | *Geum montanum* | Flowers | Val di Sole | | South | 46.24360124596042, 10.653712543817255 | 2300 | 12 | 26-06-21 |
| Geu-F-B-S-2 | *Geum montanum* | Flowers | Val di Sole | | South | 46.24360124596042, 10.653712543817255 | 2300 | 12 | 26-06-21 |
| Geu-F-B-S-3 | *Geum montanum* | Flowers | Val di Sole | | South | 46.24360124596042, 10.653712543817255 | 2300 | 12 | 26-06-21 |
| Alc-F-C-N-1 | *Alchemilla* sp. | Flowers | Val di Pejo | C | North | 46.33063108126337, 10.66518914945317 | 2200 | 14 | 03-07-21 |
| Alc-F-C-N-2 | *Alchemilla* sp. | Flowers | Val di Pejo | | North | 46.33063108126337, 10.66518914945317 | 2200 | 14 | 03-07-21 |
| Alc-F-C-N-3 | *Alchemilla* sp. | Flowers | Val di Pejo | | North | 46.33063108126337, 10.66518914945317 | 2200 | 14 | 03-07-21 |
| Alc-F-C-S-1 | *Alchemilla* sp. | Flowers | Val di Pejo | | South | 46.33063108126337, 10.66518914945317 | 2200 | 14 | 03-07-21 |
| Alc-F-C-S-2 | *Alchemilla* sp. | Flowers | Val di Pejo | | South | 46.33063108126337, 10.66518914945317 | 2200 | 14 | 03-07-21 |
| Alc-F-C-S-3 | *Alchemilla* sp. | Flowers | Val di Pejo | | South | 46.33063108126337, 10.66518914945317 | 2200 | 14 | 03-07-21 |
| Geu-F-C-N-1 | *Geum montanum* | Flowers | Val di Pejo | | North | 46.332063857223105, 10.6649275161016 | 2200 | 14 | 03-07-21 |
| Geu-F-C-N-2 | *Geum montanum* | Flowers | Val di Pejo | | North | 46.332063857223105, 10.6649275161016 | 2200 | 14 | 03-07-21 |
| Geu-F-C-N-3 | *Geum montanum* | Flowers | Val di Pejo | | North | 46.332063857223105, 10.6649275161016 | 2200 | 14 | 03-07-21 |
| Geu-F-C-S-1 | *Geum montanum* | Flowers | Val di Pejo | | South | 46.332063857223105, 10.6649275161016 | 2200 | 14 | 03-07-21 |
| Geu-F-C-S-2 | *Geum montanum* | Flowers | Val di Pejo | | South | 46.332063857223105, 10.6649275161016 | 2200 | 14 | 03-07-21 |
| Geu-F-C-S-3 | *Geum montanum* | Flowers | Val di Pejo | | South | 46.332063857223105, 10.6649275161016 | 2200 | 14 | 03-07-21 |
| Alc-F-D-N-1 | *Alchemilla* sp. | Flowers | Val Rendena | D | North | 46.223249397684846, 10.87747766791696 | 2100 | 12 | 04-07-21 |
| Alc-F-D-N-2 | *Alchemilla* sp. | Flowers | Val Rendena | | North | 46.223249397684846, 10.87747766791696 | 2100 | 12 | 04-07-21 |
| Alc-F-D-N-3 | *Alchemilla* sp. | Flowers | Val Rendena | | North | 46.223249397684846, 10.87747766791696 | 2100 | 12 | 04-07-21 |
| Alc-F-D-S-1 | *Alchemilla* sp. | Flowers | Val Rendena | | South | 46.223249397684846, 10.87747766791696 | 2100 | 12 | 04-07-21 |
| Alc-F-D-S-2 | *Alchemilla* sp. | Flowers | Val Rendena | | South | 46.223249397684846, 10.87747766791696 | 2100 | 12 | 04-07-21 |

| | | | | | | | | | |
|---|---|---|---|---|---|---|---|---|---|
| Alc-F-D-S-3 | *Alchemilla* sp. | Flowers | Val Rendena | | South | 46.223249397684846, 10.87747766791696 | 2100 | 12 | 04-07-21 |
| Geu-F-D-N-1 | *Geum montanum* | Flowers | Val Rendena | | North | 46.223249397684846, 10.87747766791696 | 2100 | 12 | 04-07-21 |
| Geu-F-D-N-2 | *Geum montanum* | Flowers | Val Rendena | | North | 46.223249397684846, 10.87747766791696 | 2100 | 12 | 04-07-21 |
| Geu-F-D-N-3 | *Geum montanum* | Flowers | Val Rendena | | North | 46.223249397684846, 10.87747766791696 | 2100 | 12 | 04-07-21 |
| Geu-F-D-S-1 | *Geum montanum* | Flowers | Val Rendena | | South | 46.223249397684846, 10.87747766791696 | 2100 | 12 | 04-07-21 |
| Geu-F-D-S-2 | *Geum montanum* | Flowers | Val Rendena | | South | 46.223249397684846, 10.87747766791696 | 2100 | 12 | 04-07-21 |
| Geu-F-D-S-3 | *Geum montanum* | Flowers | Val Rendena | | South | 46.223249397684846, 10.87747766791696 | 2100 | 12 | 04-07-21 |
| Dry-F-D-N-1 | *Dryas octopetala* | Flowers | Val Rendena | | North | 46.223249397684846, 10.87747766791696 | 2100 | 12 | 04-07-21 |
| Dry-F-D-N-2 | *Dryas octopetala* | Flowers | Val Rendena | | North | 46.223249397684846, 10.87747766791696 | 2100 | 12 | 04-07-21 |
| Dry-F-D-N-3 | *Dryas octopetala* | Flowers | Val Rendena | | North | 46.223249397684846, 10.87747766791696 | 2100 | 12 | 04-07-21 |
| Dry-F-D-S-1 | *Dryas octopetala* | Flowers | Val Rendena | | South | 46.223249397684846, 10.87747766791696 | 2100 | 12 | 04-07-21 |
| Dry-F-D-S-2 | *Dryas octopetala* | Flowers | Val Rendena | | South | 46.223249397684846, 10.87747766791696 | 2100 | 12 | 04-07-21 |
| Dry-F-D-S-3 | *Dryas octopetala* | Flowers | Val Rendena | | South | 46.223249397684846, 10.87747766791696 | 2100 | 12 | 04-07-21 |
| Dry-F-E-N-1 | *Dryas octopetala* | Flowers | South Tyrol | | North | 46.355572840650794, 11.443758796261003 | 2288 | 8 | 02-08-21 |
| Dry-F-E-N-2 | *Dryas octopetala* | Flowers | South Tyrol | | North | 46.355572840650794, 11.443758796261003 | 2288 | 8 | 02-08-21 |
| Dry-F-E-N-3 | *Dryas octopetala* | Flowers | South Tyrol | E | North | 46.355572840650794, 11.443758796261003 | 2288 | 8 | 02-08-21 |
| Dry-F-E-S-1 | *Dryas octopetala* | Flowers | South Tyrol | | South | 46.355572840650794, 11.443758796261003 | 2288 | 8 | 02-08-21 |
| Dry-F-E-S-2 | *Dryas octopetala* | Flowers | South Tyrol | | South | 46.355572840650794, 11.443758796261003 | 2288 | 8 | 02-08-21 |
| Dry-F-E-S-3 | *Dryas octopetala* | Flowers | South Tyrol | | South | 46.355572840650794, 11.443758796261003 | 2288 | 8 | 02-08-21 |
| Alc-F-F-N-1 | *Alchemilla* sp. | Flowers | Stelvio Park | | North | 46.473951654331785, 10.826623016285474 | 1050 | 16 | 12-08-21 |
| Alc-F-F-N-2 | *Alchemilla* sp. | Flowers | Stelvio Park | | North | 46.473951654331785, 10.826623016285474 | 1050 | 16 | 12-08-21 |
| Alc-F-F-N-3 | *Alchemilla* sp. | Flowers | Stelvio Park | | North | 46.473951654331785, 10.826623016285474 | 1050 | 16 | 12-08-21 |
| Alc-F-F-S-1 | *Alchemilla* sp. | Flowers | Stelvio Park | | South | 46.473951654331785, 10.826623016285474 | 1050 | 16 | 12-08-21 |
| Alc-F-F-S-2 | *Alchemilla* sp. | Flowers | Stelvio Park | | South | 46.473951654331785, 10.826623016285474 | 1050 | 16 | 12-08-21 |
| Alc-F-F-S-3 | *Alchemilla* sp. | Flowers | Stelvio Park | F | South | 46.473951654331785, 10.826623016285474 | 1050 | 16 | 12-08-21 |
| Geu-F-F-N-1 | *Geum montanum* | Flowers | Stelvio Park | | North | 46.46332969253064, 10.81141962884433 | 1050 | 14 | 12-08-21 |
| Geu-F-F-N-2 | *Geum montanum* | Flowers | Stelvio Park | | North | 46.46332969253064, 10.81141962884433 | 1050 | 14 | 12-08-21 |
| Geu-F-F-N-3 | *Geum montanum* | Flowers | Stelvio Park | | North | 46.46332969253064, 10.81141962884433 | 1050 | 14 | 12-08-21 |
| Geu-F-F-S-1 | *Geum montanum* | Flowers | Stelvio Park | | South | 46.46332969253064, 10.81141962884433 | 1050 | 14 | 12-08-21 |
| Geu-F-F-S-2 | *Geum montanum* | Flowers | Stelvio Park | | South | 46.46332969253064, 10.81141962884433 | 1050 | 14 | 12-08-21 |
| Geu-F-F-S-3 | *Geum montanum* | Flowers | Stelvio Park | | South | 46.46332969253064, 10.81141962884433 | 1050 | 14 | 12-08-21 |
| Alc-F-G-N-1 | *Alchemilla* sp. | Flowers | Val di Fassa | | North | 46.48564476989307, 11.814082677976842 | 2300 | 10 | 19-08-21 |
| Alc-F-G-N-2 | *Alchemilla* sp. | Flowers | Val di Fassa | | North | 46.48564476989307, 11.814082677976842 | 2300 | 10 | 19-08-21 |
| Alc-F-G-N-3 | *Alchemilla* sp. | Flowers | Val di Fassa | | North | 46.48564476989307, 11.814082677976842 | 2300 | 10 | 19-08-21 |
| Alc-F-G-S-1 | *Alchemilla* sp. | Flowers | Val di Fassa | | South | 46.48564476989307, 11.814082677976842 | 2300 | 10 | 19-08-21 |
| Alc-F-G-S-2 | *Alchemilla* sp. | Flowers | Val di Fassa | | South | 46.48564476989307, 11.814082677976842 | 2300 | 10 | 19-08-21 |
| Alc-F-G-S-3 | *Alchemilla* sp. | Flowers | Val di Fassa | | South | 46.48564476989307, 11.814082677976842 | 2300 | 10 | 19-08-21 |
| Geu-F-G-N-1 | *Geum montanum* | Flowers | Val di Fassa | | North | 46.488981227296485, 11.81204928225736 | 2300 | 10 | 19-08-21 |
| Geu-F-G-N-2 | *Geum montanum* | Flowers | Val di Fassa | | North | 46.488981227296485, 11.81204928225736 | 2300 | 10 | 19-08-21 |
| Geu-F-G-N-3 | *Geum montanum* | Flowers | Val di Fassa | G | North | 46.488981227296485, 11.81204928225736 | 2300 | 10 | 19-08-21 |
| Geu-F-G-S-1 | *Geum montanum* | Flowers | Val di Fassa | | South | 46.488981227296485, 11.81204928225736 | 2300 | 10 | 19-08-21 |
| Geu-F-G-S-2 | *Geum montanum* | Flowers | Val di Fassa | | South | 46.488981227296485, 11.81204928225736 | 2300 | 10 | 19-08-21 |
| Geu-F-G-S-3 | *Geum montanum* | Flowers | Val di Fassa | | South | 46.488981227296485, 11.81204928225736 | 2300 | 10 | 19-08-21 |
| Dry-F-G-N-1 | *Dryas octopetala* | Flowers | Val di Fassa | | North | 46.48574359995444, 11.814133713209195 | 2300 | 9 | 19-08-21 |
| Dry-F-G-N-2 | *Dryas octopetala* | Flowers | Val di Fassa | | North | 46.48574359995444, 11.814133713209195 | 2300 | 9 | 19-08-21 |
| Dry-F-G-N-3 | *Dryas octopetala* | Flowers | Val di Fassa | | North | 46.48574359995444, 11.814133713209195 | 2300 | 9 | 19-08-21 |

| | | | | | | | | | |
|---|---|---|---|---|---|---|---|---|---|
| Dry-F-G-S-1 | *Dryas octopetala* | Flowers | Val di Fassa | | South | 46.48574359995444, 11.814133713209195 | 2300 | 9 | 19-08-21 |
| Dry-F-G-S-2 | *Dryas octopetala* | Flowers | Val di Fassa | | South | 46.48574359995444, 11.814133713209195 | 2300 | 9 | 19-08-21 |
| Dry-F-G-S-3 | *Dryas octopetala* | Flowers | Val di Fassa | | South | 46.48574359995444, 11.814133713209195 | 2300 | 9 | 19-08-21 |
| Alc-L-A-N-1 | *Alchemilla* sp. | Leaves | Val di Non | | North | 46.43236880107553, 10.887484371878964 | 1950 | 14 | 20-06-21 |
| Alc-L-A-N-2 | *Alchemilla* sp. | Leaves | Val di Non | | North | 46.43236880107553, 10.887484371878964 | 1950 | 14 | 20-06-21 |
| Alc-L-A-N-3 | *Alchemilla* sp. | Leaves | Val di Non | | North | 46.43236880107553, 10.887484371878964 | 1950 | 14 | 20-06-21 |
| Alc-L-A-S-1 | *Alchemilla* sp. | Leaves | Val di Non | | South | 46.43236880107553, 10.887484371878964 | 1950 | 14 | 20-06-21 |
| Alc-L-A-S-2 | *Alchemilla* sp. | Leaves | Val di Non | | South | 46.43236880107553, 10.887484371878964 | 1950 | 14 | 20-06-21 |
| Alc-L-A-S-3 | *Alchemilla* sp. | Leaves | Val di Non | | South | 46.43236880107553, 10.887484371878964 | 1950 | 14 | 20-06-21 |
| Geu-L-A-N-1 | *Geum montanum* | Leaves | Val di Non | A | North | 46.43140936845373, 10.874719416962568 | 2100 | 12 | 20-06-21 |
| Geu-L-A-N-2 | *Geum montanum* | Leaves | Val di Non | | North | 46.43140936845373, 10.874719416962568 | 2100 | 12 | 20-06-21 |
| Geu-L-A-N-3 | *Geum montanum* | Leaves | Val di Non | | North | 46.43140936845373, 10.874719416962568 | 2100 | 12 | 20-06-21 |
| Geu-L-A-S-1 | *Geum montanum* | Leaves | Val di Non | | South | 46.43140936845373, 10.874719416962568 | 2100 | 12 | 20-06-21 |
| Geu-L-A-S-2 | *Geum montanum* | Leaves | Val di Non | | South | 46.43140936845373, 10.874719416962568 | 2100 | 12 | 20-06-21 |
| Geu-L-A-S-3 | *Geum montanum* | Leaves | Val di Non | | South | 46.43140936845373, 10.874719416962568 | 2100 | 12 | 20-06-21 |
| Alc-L-B-N-1 | *Alchemilla* sp. | Leaves | Val di Sole | | North | 46.281873339437034, 10.809519461015759 | 1800 | 14 | 27-06-21 |
| Alc-L-B-N-2 | *Alchemilla* sp. | Leaves | Val di Sole | | North | 46.281873339437034, 10.809519461015759 | 1800 | 14 | 27-06-21 |
| Alc-L-B-N-3 | *Alchemilla* sp. | Leaves | Val di Sole | | North | 46.281873339437034, 10.809519461015759 | 1800 | 14 | 27-06-21 |
| Alc-L-B-S-1 | *Alchemilla* sp. | Leaves | Val di Sole | | South | 46.281873339437034, 10.809519461015759 | 1800 | 14 | 27-06-21 |
| Alc-L-B-S-2 | *Alchemilla* sp. | Leaves | Val di Sole | | South | 46.281873339437034, 10.809519461015759 | 1800 | 14 | 27-06-21 |
| Alc-L-B-S-3 | *Alchemilla* sp. | Leaves | Val di Sole | | South | 46.281873339437034, 10.809519461015759 | 1800 | 14 | 27-06-21 |
| Geu-L-B-N-1 | *Geum montanum* | Leaves | Val di Sole | B | North | 46.24360124596042, 10.653712543817255 | 2300 | 12 | 26-06-21 |
| Geu-L-B-N-2 | *Geum montanum* | Leaves | Val di Sole | | North | 46.24360124596042, 10.653712543817255 | 2300 | 12 | 26-06-21 |
| Geu-L-B-N-3 | *Geum montanum* | Leaves | Val di Sole | | North | 46.24360124596042, 10.653712543817255 | 2300 | 12 | 26-06-21 |
| Geu-L-B-S-1 | *Geum montanum* | Leaves | Val di Sole | | South | 46.24360124596042, 10.653712543817255 | 2300 | 12 | 26-06-21 |
| Geu-L-B-S-2 | *Geum montanum* | Leaves | Val di Sole | | South | 46.24360124596042, 10.653712543817255 | 2300 | 12 | 26-06-21 |
| Geu-L-B-S-3 | *Geum montanum* | Leaves | Val di Sole | | South | 46.24360124596042, 10.653712543817255 | 2300 | 12 | 26-06-21 |
| Alc-L-C-N-1 | *Alchemilla* sp. | Leaves | Val di Pejo | | North | 46.33063108126337, 10.66518914945317 | 2200 | 14 | 03-07-21 |
| Alc-L-C-N-2 | *Alchemilla* sp. | Leaves | Val di Pejo | | North | 46.33063108126337, 10.66518914945317 | 2200 | 14 | 03-07-21 |
| Alc-L-C-N-3 | *Alchemilla* sp. | Leaves | Val di Pejo | | North | 46.33063108126337, 10.66518914945317 | 2200 | 14 | 03-07-21 |
| Alc-L-C-S-1 | *Alchemilla* sp. | Leaves | Val di Pejo | | South | 46.33063108126337, 10.66518914945317 | 2200 | 14 | 03-07-21 |
| Alc-L-C-S-2 | *Alchemilla* sp. | Leaves | Val di Pejo | | South | 46.33063108126337, 10.66518914945317 | 2200 | 14 | 03-07-21 |
| Alc-L-C-S-3 | *Alchemilla* sp. | Leaves | Val di Pejo | | South | 46.33063108126337, 10.66518914945317 | 2200 | 14 | 03-07-21 |
| Geu-L-C-N-1 | *Geum montanum* | Leaves | Val di Pejo | C | North | 46.332063857223105, 10.6649275161016 | 2200 | 14 | 03-07-21 |
| Geu-L-C-N-2 | *Geum montanum* | Leaves | Val di Pejo | | North | 46.332063857223105, 10.6649275161016 | 2200 | 14 | 03-07-21 |
| Geu-L-C-N-3 | *Geum montanum* | Leaves | Val di Pejo | | North | 46.332063857223105, 10.6649275161016 | 2200 | 14 | 03-07-21 |
| Geu-L-C-S-1 | *Geum montanum* | Leaves | Val di Pejo | | South | 46.332063857223105, 10.6649275161016 | 2200 | 14 | 03-07-21 |
| Geu-L-C-S-2 | *Geum montanum* | Leaves | Val di Pejo | | South | 46.332063857223105, 10.6649275161016 | 2200 | 14 | 03-07-21 |
| Geu-L-C-S-3 | *Geum montanum* | Leaves | Val di Pejo | | South | 46.332063857223105, 10.6649275161016 | 2200 | 14 | 03-07-21 |
| Alc-L-D-N-1 | *Alchemilla* sp. | Leaves | Val Rendena | | North | 46.223249397684846, 10.87747766791696 | 2100 | 12 | 04-07-21 |
| Alc-L-D-N-2 | *Alchemilla* sp. | Leaves | Val Rendena | | North | 46.223249397684846, 10.87747766791696 | 2100 | 12 | 04-07-21 |
| Alc-L-D-N-3 | *Alchemilla* sp. | Leaves | Val Rendena | | North | 46.223249397684846, 10.87747766791696 | 2100 | 12 | 04-07-21 |
| Alc-L-D-S-1 | *Alchemilla* sp. | Leaves | Val Rendena | | South | 46.223249397684846, 10.87747766791696 | 2100 | 12 | 04-07-21 |
| Alc-L-D-S-2 | *Alchemilla* sp. | Leaves | Val Rendena | | South | 46.223249397684846, 10.87747766791696 | 2100 | 12 | 04-07-21 |
| Alc-L-D-S-3 | *Alchemilla* sp. | Leaves | Val Rendena | | South | 46.223249397684846, 10.87747766791696 | 2100 | 12 | 04-07-21 |
| Geu-L-D-N-1 | *Geum montanum* | Leaves | Val Rendena | | North | 46.223249397684846, 10.87747766791696 | 2100 | 12 | 04-07-21 |

| | | | | | | | | | |
|---|---|---|---|---|---|---|---|---|---|
| Geu-L-D-N-2 | *Geum montanum* | Leaves | Val Rendena | | North | 46.223249397684846, 10.87747766791696 | 2100 | 12 | 04-07-21 |
| Geu-L-D-N-3 | *Geum montanum* | Leaves | Val Rendena | D | North | 46.223249397684846, 10.87747766791696 | 2100 | 12 | 04-07-21 |
| Geu-L-D-S-1 | *Geum montanum* | Leaves | Val Rendena | | South | 46.223249397684846, 10.87747766791696 | 2100 | 12 | 04-07-21 |
| Geu-L-D-S-2 | *Geum montanum* | Leaves | Val Rendena | | South | 46.223249397684846, 10.87747766791696 | 2100 | 12 | 04-07-21 |
| Geu-L-D-S-3 | *Geum montanum* | Leaves | Val Rendena | | South | 46.223249397684846, 10.87747766791696 | 2100 | 12 | 04-07-21 |
| Dry-L-D-N-1 | *Dryas octopetala* | Leaves | Val Rendena | | North | 46.223249397684846, 10.87747766791696 | 2100 | 12 | 04-07-21 |
| Dry-L-D-N-2 | *Dryas octopetala* | Leaves | Val Rendena | | North | 46.223249397684846, 10.87747766791696 | 2100 | 12 | 04-07-21 |
| Dry-L-D-N-3 | *Dryas octopetala* | Leaves | Val Rendena | | North | 46.223249397684846, 10.87747766791696 | 2100 | 12 | 04-07-21 |
| Dry-L-D-S-1 | *Dryas octopetala* | Leaves | Val Rendena | | South | 46.223249397684846, 10.87747766791696 | 2100 | 12 | 04-07-21 |
| Dry-L-D-S-2 | *Dryas octopetala* | Leaves | Val Rendena | | South | 46.223249397684846, 10.87747766791696 | 2100 | 12 | 04-07-21 |
| Dry-L-D-S-3 | *Dryas octopetala* | Leaves | Val Rendena | | South | 46.223249397684846, 10.87747766791696 | 2100 | 12 | 04-07-21 |
| Dry-L-E-N-1 | *Dryas octopetala* | Leaves | South Tyrol | | North | 46.355572840650794, 11.443758796261003 | 2288 | 8 | 02-08-21 |
| Dry-L-E-N-2 | *Dryas octopetala* | Leaves | South Tyrol | | North | 46.355572840650794, 11.443758796261003 | 2288 | 8 | 02-08-21 |
| Dry-L-E-N-3 | *Dryas octopetala* | Leaves | South Tyrol | E | North | 46.355572840650794, 11.443758796261003 | 2288 | 8 | 02-08-21 |
| Dry-L-E-S-1 | *Dryas octopetala* | Leaves | South Tyrol | | South | 46.355572840650794, 11.443758796261003 | 2288 | 8 | 02-08-21 |
| Dry-L-E-S-2 | *Dryas octopetala* | Leaves | South Tyrol | | South | 46.355572840650794, 11.443758796261003 | 2288 | 8 | 02-08-21 |
| Dry-L-E-S-3 | *Dryas octopetala* | Leaves | South Tyrol | | South | 46.355572840650794, 11.443758796261003 | 2288 | 8 | 02-08-21 |
| Alc-L-F-N-1 | *Alchemilla* sp. | Leaves | Stelvio Park | | North | 46.473951654331785, 10.826623016285474 | 1050 | 16 | 12-08-21 |
| Alc-L-F-N-2 | *Alchemilla* sp. | Leaves | Stelvio Park | | North | 46.473951654331785, 10.826623016285474 | 1050 | 16 | 12-08-21 |
| Alc-L-F-N-3 | *Alchemilla* sp. | Leaves | Stelvio Park | | North | 46.473951654331785, 10.826623016285474 | 1050 | 16 | 12-08-21 |
| Alc-L-F-S-1 | *Alchemilla* sp. | Leaves | Stelvio Park | | South | 46.473951654331785, 10.826623016285474 | 1050 | 16 | 12-08-21 |
| Alc-L-F-S-2 | *Alchemilla* sp. | Leaves | Stelvio Park | | South | 46.473951654331785, 10.826623016285474 | 1050 | 16 | 12-08-21 |
| Alc-L-F-S-3 | *Alchemilla* sp. | Leaves | Stelvio Park | F | South | 46.473951654331785, 10.826623016285474 | 1050 | 16 | 12-08-21 |
| Geu-L-F-N-1 | *Geum montanum* | Leaves | Stelvio Park | | North | 46.46332969253064, 10.81141962884433 | 1050 | 14 | 12-08-21 |
| Geu-L-F-N-2 | *Geum montanum* | Leaves | Stelvio Park | | North | 46.46332969253064, 10.81141962884433 | 1050 | 14 | 12-08-21 |
| Geu-L-F-N-3 | *Geum montanum* | Leaves | Stelvio Park | | North | 46.46332969253064, 10.81141962884433 | 1050 | 14 | 12-08-21 |
| Geu-L-F-S-1 | *Geum montanum* | Leaves | Stelvio Park | | South | 46.46332969253064, 10.81141962884433 | 1050 | 14 | 12-08-21 |
| Geu-L-F-S-2 | *Geum montanum* | Leaves | Stelvio Park | | South | 46.46332969253064, 10.81141962884433 | 1050 | 14 | 12-08-21 |
| Geu-L-F-S-3 | *Geum montanum* | Leaves | Stelvio Park | | South | 46.46332969253064, 10.81141962884433 | 1050 | 14 | 12-08-21 |
| Alc-L-G-N-1 | *Alchemilla* sp. | Leaves | Val di Fassa | | North | 46.48564476989307, 11.814082677976842 | 2300 | 10 | 19-08-21 |
| Alc-L-G-N-2 | *Alchemilla* sp. | Leaves | Val di Fassa | | North | 46.48564476989307, 11.814082677976842 | 2300 | 10 | 19-08-21 |
| Alc-L-G-N-3 | *Alchemilla* sp. | Leaves | Val di Fassa | | North | 46.48564476989307, 11.814082677976842 | 2300 | 10 | 19-08-21 |
| Alc-L-G-S-1 | *Alchemilla* sp. | Leaves | Val di Fassa | | South | 46.48564476989307, 11.814082677976842 | 2300 | 10 | 19-08-21 |
| Alc-L-G-S-2 | *Alchemilla* sp. | Leaves | Val di Fassa | | South | 46.48564476989307, 11.814082677976842 | 2300 | 10 | 19-08-21 |
| Alc-L-G-S-3 | *Alchemilla* sp. | Leaves | Val di Fassa | | South | 46.48564476989307, 11.814082677976842 | 2300 | 10 | 19-08-21 |
| Geu-L-G-N-1 | *Geum montanum* | Leaves | Val di Fassa | | North | 46.488981227296485, 11.81204928225736 | 2300 | 10 | 19-08-21 |
| Geu-L-G-N-2 | *Geum montanum* | Leaves | Val di Fassa | | North | 46.488981227296485, 11.81204928225736 | 2300 | 10 | 19-08-21 |
| Geu-L-G-N-3 | *Geum montanum* | Leaves | Val di Fassa | G | North | 46.488981227296485, 11.81204928225736 | 2300 | 10 | 19-08-21 |
| Geu-L-G-S-1 | *Geum montanum* | Leaves | Val di Fassa | | South | 46.488981227296485, 11.81204928225736 | 2300 | 10 | 19-08-21 |
| Geu-L-G-S-2 | *Geum montanum* | Leaves | Val di Fassa | | South | 46.488981227296485, 11.81204928225736 | 2300 | 10 | 19-08-21 |
| Geu-L-G-S-3 | *Geum montanum* | Leaves | Val di Fassa | | South | 46.488981227296485, 11.81204928225736 | 2300 | 10 | 19-08-21 |
| Dry-L-G-N-1 | *Dryas octopetala* | Leaves | Val di Fassa | | North | 46.48574359995444, 11.814133713209195 | 2300 | 9 | 19-08-21 |
| Dry-L-G-N-2 | *Dryas octopetala* | Leaves | Val di Fassa | | North | 46.48574359995444, 11.814133713209195 | 2300 | 9 | 19-08-21 |
| Dry-L-G-N-3 | *Dryas octopetala* | Leaves | Val di Fassa | | North | 46.48574359995444, 11.814133713209195 | 2300 | 9 | 19-08-21 |
| Dry-L-G-S-1 | *Dryas octopetala* | Leaves | Val di Fassa | | South | 46.48574359995444, 11.814133713209195 | 2300 | 9 | 19-08-21 |
| Dry-L-G-S-2 | *Dryas octopetala* | Leaves | Val di Fassa | | South | 46.48574359995444, 11.814133713209195 | 2300 | 9 | 19-08-21 |

| | | | | | | | | | |
|---|---|---|---|---|---|---|---|---|---|
| Dry-L-G-S-3 | *Dryas octopetala* | Leaves | Val di Fassa | | South | 46.48574359995444, 11.814133713209195 | 2300 | 9 | 19-08-21 |
| Alc-R-A-N-1 | *Alchemilla* sp. | Roots | Val di Non | | North | 46.43236880107553, 10.887484371878964 | 1950 | 14 | 20-06-21 |
| Alc-R-A-N-2 | *Alchemilla* sp. | Roots | Val di Non | | North | 46.43236880107553, 10.887484371878964 | 1950 | 14 | 20-06-21 |
| Alc-R-A-N-3 | *Alchemilla* sp. | Roots | Val di Non | | North | 46.43236880107553, 10.887484371878964 | 1950 | 14 | 20-06-21 |
| Alc-R-A-S-1 | *Alchemilla* sp. | Roots | Val di Non | | South | 46.43236880107553, 10.887484371878964 | 1950 | 14 | 20-06-21 |
| Alc-R-A-S-2 | *Alchemilla* sp. | Roots | Val di Non | | South | 46.43236880107553, 10.887484371878964 | 1950 | 14 | 20-06-21 |
| Alc-R-A-S-3 | *Alchemilla* sp. | Roots | Val di Non | A | South | 46.43236880107553, 10.887484371878964 | 1950 | 14 | 20-06-21 |
| Geu-R-A-N-1 | *Geum montanum* | Roots | Val di Non | | North | 46.43140936845373, 10.874719416962568 | 2100 | 12 | 20-06-21 |
| Geu-R-A-N-2 | *Geum montanum* | Roots | Val di Non | | North | 46.43140936845373, 10.874719416962568 | 2100 | 12 | 20-06-21 |
| Geu-R-A-N-3 | *Geum montanum* | Roots | Val di Non | | North | 46.43140936845373, 10.874719416962568 | 2100 | 12 | 20-06-21 |
| Geu-R-A-S-1 | *Geum montanum* | Roots | Val di Non | | South | 46.43140936845373, 10.874719416962568 | 2100 | 12 | 20-06-21 |
| Geu-R-A-S-2 | *Geum montanum* | Roots | Val di Non | | South | 46.43140936845373, 10.874719416962568 | 2100 | 12 | 20-06-21 |
| Geu-R-A-S-3 | *Geum montanum* | Roots | Val di Non | | South | 46.43140936845373, 10.874719416962568 | 2100 | 12 | 20-06-21 |
| Alc-R-B-N-1 | *Alchemilla* sp. | Roots | Val di Sole | | North | 46.281873339437034, 10.809519461015759 | 1800 | 14 | 27-06-21 |
| Alc-R-B-N-2 | *Alchemilla* sp. | Roots | Val di Sole | | North | 46.281873339437034, 10.809519461015759 | 1800 | 14 | 27-06-21 |
| Alc-R-B-N-3 | *Alchemilla* sp. | Roots | Val di Sole | | North | 46.281873339437034, 10.809519461015759 | 1800 | 14 | 27-06-21 |
| Alc-R-B-S-1 | *Alchemilla* sp. | Roots | Val di Sole | | South | 46.281873339437034, 10.809519461015759 | 1800 | 14 | 27-06-21 |
| Alc-R-B-S-2 | *Alchemilla* sp. | Roots | Val di Sole | | South | 46.281873339437034, 10.809519461015759 | 1800 | 14 | 27-06-21 |
| Alc-R-B-S-3 | *Alchemilla* sp. | Roots | Val di Sole | B | South | 46.281873339437034, 10.809519461015759 | 1800 | 14 | 27-06-21 |
| Geu-R-B-N-1 | *Geum montanum* | Roots | Val di Sole | | North | 46.24360124596042, 10.653712543817255 | 2300 | 12 | 26-06-21 |
| Geu-R-B-N-2 | *Geum montanum* | Roots | Val di Sole | | North | 46.24360124596042, 10.653712543817255 | 2300 | 12 | 26-06-21 |
| Geu-R-B-N-3 | *Geum montanum* | Roots | Val di Sole | | North | 46.24360124596042, 10.653712543817255 | 2300 | 12 | 26-06-21 |
| Geu-R-B-S-1 | *Geum montanum* | Roots | Val di Sole | | South | 46.24360124596042, 10.653712543817255 | 2300 | 12 | 26-06-21 |
| Geu-R-B-S-2 | *Geum montanum* | Roots | Val di Sole | | South | 46.24360124596042, 10.653712543817255 | 2300 | 12 | 26-06-21 |
| Geu-R-B-S-3 | *Geum montanum* | Roots | Val di Sole | | South | 46.24360124596042, 10.653712543817255 | 2300 | 12 | 26-06-21 |
| Alc-R-C-N-1 | *Alchemilla* sp. | Roots | Val di Pejo | | North | 46.33063108126337, 10.66518914945317 | 2200 | 14 | 03-07-21 |
| Alc-R-C-N-2 | *Alchemilla* sp. | Roots | Val di Pejo | | North | 46.33063108126337, 10.66518914945317 | 2200 | 14 | 03-07-21 |
| Alc-R-C-N-3 | *Alchemilla* sp. | Roots | Val di Pejo | | North | 46.33063108126337, 10.66518914945317 | 2200 | 14 | 03-07-21 |
| Alc-R-C-S-1 | *Alchemilla* sp. | Roots | Val di Pejo | | South | 46.33063108126337, 10.66518914945317 | 2200 | 14 | 03-07-21 |
| Alc-R-C-S-2 | *Alchemilla* sp. | Roots | Val di Pejo | | South | 46.33063108126337, 10.66518914945317 | 2200 | 14 | 03-07-21 |
| Alc-R-C-S-3 | *Alchemilla* sp. | Roots | Val di Pejo | C | South | 46.33063108126337, 10.66518914945317 | 2200 | 14 | 03-07-21 |
| Geu-R-C-N-1 | *Geum montanum* | Roots | Val di Pejo | | North | 46.332063857223105, 10.6649275161016 | 2200 | 14 | 03-07-21 |
| Geu-R-C-N-2 | *Geum montanum* | Roots | Val di Pejo | | North | 46.332063857223105, 10.6649275161016 | 2200 | 14 | 03-07-21 |
| Geu-R-C-N-3 | *Geum montanum* | Roots | Val di Pejo | | North | 46.332063857223105, 10.6649275161016 | 2200 | 14 | 03-07-21 |
| Geu-R-C-S-1 | *Geum montanum* | Roots | Val di Pejo | | South | 46.332063857223105, 10.6649275161016 | 2200 | 14 | 03-07-21 |
| Geu-R-C-S-2 | *Geum montanum* | Roots | Val di Pejo | | South | 46.332063857223105, 10.6649275161016 | 2200 | 14 | 03-07-21 |
| Geu-R-C-S-3 | *Geum montanum* | Roots | Val di Pejo | | South | 46.332063857223105, 10.6649275161016 | 2200 | 14 | 03-07-21 |
| Alc-R-D-N-1 | *Alchemilla* sp. | Roots | Val Rendena | D | North | 46.223249397684846, 10.87747766791696 | 2100 | 12 | 04-07-21 |
| Alc-R-D-N-2 | *Alchemilla* sp. | Roots | Val Rendena | | North | 46.223249397684846, 10.87747766791696 | 2100 | 12 | 04-07-21 |
| Alc-R-D-N-3 | *Alchemilla* sp. | Roots | Val Rendena | | North | 46.223249397684846, 10.87747766791696 | 2100 | 12 | 04-07-21 |
| Alc-R-D-S-1 | *Alchemilla* sp. | Roots | Val Rendena | | South | 46.223249397684846, 10.87747766791696 | 2100 | 12 | 04-07-21 |
| Alc-R-D-S-2 | *Alchemilla* sp. | Roots | Val Rendena | | South | 46.223249397684846, 10.87747766791696 | 2100 | 12 | 04-07-21 |
| Alc-R-D-S-3 | *Alchemilla* sp. | Roots | Val Rendena | | South | 46.223249397684846, 10.87747766791696 | 2100 | 12 | 04-07-21 |
| Geu-R-D-N-1 | *Geum montanum* | Roots | Val Rendena | | North | 46.223249397684846, 10.87747766791696 | 2100 | 12 | 04-07-21 |
| Geu-R-D-N-2 | *Geum montanum* | Roots | Val Rendena | | North | 46.223249397684846, 10.87747766791696 | 2100 | 12 | 04-07-21 |
| Geu-R-D-N-3 | *Geum montanum* | Roots | Val Rendena | | North | 46.223249397684846, 10.87747766791696 | 2100 | 12 | 04-07-21 |

| | | | | | | | | | |
|---|---|---|---|---|---|---|---|---|---|
| Geu-R-D-S-1 | *Geum montanum* | Roots | Val Rendena | | South | 46.223249397684846, 10.87747766791696 | 2100 | 12 | 04-07-21 |
| Geu-R-D-S-2 | *Geum montanum* | Roots | Val Rendena | | South | 46.223249397684846, 10.87747766791696 | 2100 | 12 | 04-07-21 |
| Geu-R-D-S-3 | *Geum montanum* | Roots | Val Rendena | | South | 46.223249397684846, 10.87747766791696 | 2100 | 12 | 04-07-21 |
| Dry-R-D-N-1 | *Dryas octopetala* | Roots | Val Rendena | | North | 46.223249397684846, 10.87747766791696 | 2100 | 12 | 04-07-21 |
| Dry-R-D-N-2 | *Dryas octopetala* | Roots | Val Rendena | | North | 46.223249397684846, 10.87747766791696 | 2100 | 12 | 04-07-21 |
| Dry-R-D-N-3 | *Dryas octopetala* | Roots | Val Rendena | | North | 46.223249397684846, 10.87747766791696 | 2100 | 12 | 04-07-21 |
| Dry-R-D-S-1 | *Dryas octopetala* | Roots | Val Rendena | | South | 46.223249397684846, 10.87747766791696 | 2100 | 12 | 04-07-21 |
| Dry-R-D-S-2 | *Dryas octopetala* | Roots | Val Rendena | | South | 46.223249397684846, 10.87747766791696 | 2100 | 12 | 04-07-21 |
| Dry-R-D-S-3 | *Dryas octopetala* | Roots | Val Rendena | | South | 46.223249397684846, 10.87747766791696 | 2100 | 12 | 04-07-21 |
| Dry-R-E-N-1 | *Dryas octopetala* | Roots | South Tyrol | | North | 46.355572840650794, 11.443758796261003 | 2288 | 8 | 02-08-21 |
| Dry-R-E-N-2 | *Dryas octopetala* | Roots | South Tyrol | | North | 46.355572840650794, 11.443758796261003 | 2288 | 8 | 02-08-21 |
| Dry-R-E-N-3 | *Dryas octopetala* | Roots | South Tyrol | E | North | 46.355572840650794, 11.443758796261003 | 2288 | 8 | 02-08-21 |
| Dry-R-E-S-1 | *Dryas octopetala* | Roots | South Tyrol | | South | 46.355572840650794, 11.443758796261003 | 2288 | 8 | 02-08-21 |
| Dry-R-E-S-2 | *Dryas octopetala* | Roots | South Tyrol | | South | 46.355572840650794, 11.443758796261003 | 2288 | 8 | 02-08-21 |
| Dry-R-E-S-3 | *Dryas octopetala* | Roots | South Tyrol | | South | 46.355572840650794, 11.443758796261003 | 2288 | 8 | 02-08-21 |
| Alc-R-F-N-1 | *Alchemilla* sp. | Roots | Stelvio Park | | North | 46.473951654331785, 10.826623016285474 | 1050 | 16 | 12-08-21 |
| Alc-R-F-N-2 | *Alchemilla* sp. | Roots | Stelvio Park | | North | 46.473951654331785, 10.826623016285474 | 1050 | 16 | 12-08-21 |
| Alc-R-F-N-3 | *Alchemilla* sp. | Roots | Stelvio Park | | North | 46.473951654331785, 10.826623016285474 | 1050 | 16 | 12-08-21 |
| Alc-R-F-S-1 | *Alchemilla* sp. | Roots | Stelvio Park | | South | 46.473951654331785, 10.826623016285474 | 1050 | 16 | 12-08-21 |
| Alc-R-F-S-2 | *Alchemilla* sp. | Roots | Stelvio Park | | South | 46.473951654331785, 10.826623016285474 | 1050 | 16 | 12-08-21 |
| Alc-R-F-S-3 | *Alchemilla* sp. | Roots | Stelvio Park | F | South | 46.473951654331785, 10.826623016285474 | 1050 | 16 | 12-08-21 |
| Geu-R-F-N-1 | *Geum montanum* | Roots | Stelvio Park | | North | 46.46332969253064, 10.81141962884433 | 1050 | 14 | 12-08-21 |
| Geu-R-F-N-2 | *Geum montanum* | Roots | Stelvio Park | | North | 46.46332969253064, 10.81141962884433 | 1050 | 14 | 12-08-21 |
| Geu-R-F-N-3 | *Geum montanum* | Roots | Stelvio Park | | North | 46.46332969253064, 10.81141962884433 | 1050 | 14 | 12-08-21 |
| Geu-R-F-S-1 | *Geum montanum* | Roots | Stelvio Park | | South | 46.46332969253064, 10.81141962884433 | 1050 | 14 | 12-08-21 |
| Geu-R-F-S-2 | *Geum montanum* | Roots | Stelvio Park | | South | 46.46332969253064, 10.81141962884433 | 1050 | 14 | 12-08-21 |
| Geu-R-F-S-3 | *Geum montanum* | Roots | Stelvio Park | | South | 46.46332969253064, 10.81141962884433 | 1050 | 14 | 12-08-21 |
| Alc-R-G-N-1 | *Alchemilla* sp. | Roots | Val di Fassa | | North | 46.48564476989307, 11.814082677976842 | 2300 | 10 | 19-08-21 |
| Alc-R-G-N-2 | *Alchemilla* sp. | Roots | Val di Fassa | | North | 46.48564476989307, 11.814082677976842 | 2300 | 10 | 19-08-21 |
| Alc-R-G-N-3 | *Alchemilla* sp. | Roots | Val di Fassa | | North | 46.48564476989307, 11.814082677976842 | 2300 | 10 | 19-08-21 |
| Alc-R-G-S-1 | *Alchemilla* sp. | Roots | Val di Fassa | | South | 46.48564476989307, 11.814082677976842 | 2300 | 10 | 19-08-21 |
| Alc-R-G-S-2 | *Alchemilla* sp. | Roots | Val di Fassa | | South | 46.48564476989307, 11.814082677976842 | 2300 | 10 | 19-08-21 |
| Alc-R-G-S-3 | *Alchemilla* sp. | Roots | Val di Fassa | | South | 46.48564476989307, 11.814082677976842 | 2300 | 10 | 19-08-21 |
| Geu-R-G-N-1 | *Geum montanum* | Roots | Val di Fassa | | North | 46.488981227296485, 11.81204928225736 | 2300 | 10 | 19-08-21 |
| Geu-R-G-N-2 | *Geum montanum* | Roots | Val di Fassa | | North | 46.488981227296485, 11.81204928225736 | 2300 | 10 | 19-08-21 |
| Geu-R-G-N-3 | *Geum montanum* | Roots | Val di Fassa | G | North | 46.488981227296485, 11.81204928225736 | 2300 | 10 | 19-08-21 |
| Geu-R-G-S-1 | *Geum montanum* | Roots | Val di Fassa | | South | 46.488981227296485, 11.81204928225736 | 2300 | 10 | 19-08-21 |
| Geu-R-G-S-2 | *Geum montanum* | Roots | Val di Fassa | | South | 46.488981227296485, 11.81204928225736 | 2300 | 10 | 19-08-21 |
| Geu-R-G-S-3 | *Geum montanum* | Roots | Val di Fassa | | South | 46.488981227296485, 11.81204928225736 | 2300 | 10 | 19-08-21 |
| Dry-R-G-N-1 | *Dryas octopetala* | Roots | Val di Fassa | | North | 46.48574359995444, 11.814133713209195 | 2300 | 9 | 19-08-21 |
| Dry-R-G-N-2 | *Dryas octopetala* | Roots | Val di Fassa | | North | 46.48574359995444, 11.814133713209195 | 2300 | 9 | 19-08-21 |
| Dry-R-G-N-3 | *Dryas octopetala* | Roots | Val di Fassa | | North | 46.48574359995444, 11.814133713209195 | 2300 | 9 | 19-08-21 |
| Dry-R-G-S-1 | *Dryas octopetala* | Roots | Val di Fassa | | South | 46.48574359995444, 11.814133713209195 | 2300 | 9 | 19-08-21 |
| Dry-R-G-S-2 | *Dryas octopetala* | Roots | Val di Fassa | | South | 46.48574359995444, 11.814133713209195 | 2300 | 9 | 19-08-21 |
| Dry-R-G-S-3 | *Dryas octopetala* | Roots | Val di Fassa | | South | 46.48574359995444, 11.814133713209195 | 2300 | 9 | 19-08-21 |

**Table S2.** Summary of the 16S rRNA gene amplicon sequencing analysis of endophytic bacterial communities associated with alpine Rosaceae plants. Raw reads (prior to any filtering steps), total reads (plant and bacterial/archaeal reads), bacterial/archaeal reads (post filtering-out chloroplast and mitochondria reads), observed amplicon sequence variants (richness) and Simpson's index (alpha-diversity) are reported for each replicate (named 1, 2, and 3) of samples collected form *Alchemilla* sp. (Alc), *Dryas octopetala* (Dry), and *Geum montanum* (Geu) flower (F), leaf (L), and root (R) samples from seven different collection sites (named from A to G) from North (N) or South (S) exposure.

| Sample code | Raw reads | Total reads | Bacterial/archaeal reads | Observed amplicon sequence variants | Simpson's index |
|---|---|---|---|---|---|
| Alc-F-A-N-1 | 70236 | 33086 | 32211 | 136.39 | 0.96 |
| Alc-F-A-N-2 | 73168 | 34510 | 33410 | 111.63 | 0.91 |
| Alc-F-A-N-3 | 84107 | 39683 | 38073 | 173.67 | 0.97 |
| Alc-F-A-S-1 | 98308 | 55546 | 53102 | 133.01 | 0.91 |
| Alc-F-A-S-2 | 95232 | 47431 | 45186 | 119.11 | 0.94 |
| Alc-F-A-S-3 | 92160 | 64216 | 62586 | 85.65 | 0.39 |
| Alc-F-B-N-1 | 129076 | 73642 | 70414 | 142.35 | 0.86 |
| Alc-F-B-N-2 | 103972 | 55775 | 53683 | 180.32 | 0.95 |
| Alc-F-B-N-3 | 128084 | 65463 | 63047 | 145.26 | 0.94 |
| Alc-F-B-S-1 | 117540 | 57285 | 52422 | 188.92 | 0.93 |
| Alc-F-B-S-2 | 102452 | 62260 | 60135 | 94.43 | 0.77 |
| Alc-F-B-S-3 | 104112 | 55306 | 51851 | 116.78 | 0.88 |
| Alc-F-C-N-1 | 112694 | 54867 | 53014 | 132.95 | 0.95 |
| Alc-F-C-N-2 | 112937 | 61043 | 59123 | 143.74 | 0.89 |
| Alc-F-C-N-3 | 108178 | 59356 | 56915 | 130.79 | 0.92 |
| Alc-F-C-S-1 | 102963 | 54541 | 52666 | 160.97 | 0.85 |
| Alc-F-C-S-2 | 105379 | 53174 | 50147 | 115.9 | 0.85 |
| Alc-F-C-S-3 | 97833 | 44106 | 42796 | 102.83 | 0.92 |
| Alc-F-D-N-1 | 100938 | 49009 | 46887 | 297.14 | 0.96 |
| Alc-F-D-N-2 | 119602 | 58995 | 56089 | 376.63 | 0.97 |
| Alc-F-D-N-3 | 92979 | 38544 | 36227 | 491.41 | 0.98 |
| Alc-F-D-S-1 | 125111 | 59081 | 55952 | 422.01 | 0.98 |
| Alc-F-D-S-2 | 110998 | 51978 | 49154 | 248.59 | 0.93 |
| Alc-F-D-S-3 | 103260 | 47416 | 45457 | 239.82 | 0.96 |
| Alc-F-F-N-1 | 102903 | 43436 | 41232 | 207 | 0.97 |
| Alc-F-F-N-2 | 101260 | 46193 | 43493 | 184.36 | 0.93 |
| Alc-F-F-N-3 | 122681 | 50790 | 48381 | 185.48 | 0.94 |
| Alc-F-F-S-1 | 111066 | 43481 | 42898 | 113.52 | 0.71 |
| Alc-F-F-S-2 | 123355 | 35360 | 34279 | 114.77 | 0.84 |
| Alc-F-F-S-3 | 107074 | 38565 | 37573 | 130.33 | 0.87 |
| Alc-F-G-N-1 | 95593 | 56741 | 56245 | 135.32 | 0.87 |

| | | | | | |
|---|---|---|---|---|---|
| Alc-F-G-N-2 | 98807 | 51621 | 50331 | 102.62 | 0.88 |
| Alc-F-G-N-3 | 114908 | 51257 | 49642 | 153.89 | 0.95 |
| Alc-F-G-S-1 | 119657 | 53456 | 52542 | 217.99 | 0.93 |
| Alc-F-G-S-2 | 121658 | 59949 | 58716 | 153.71 | 0.93 |
| Alc-F-G-S-3 | 120218 | 56951 | 55102 | 159.69 | 0.9 |
| Alc-L-A-N-1 | 100296 | 42016 | 40485 | 150.75 | 0.88 |
| Alc-L-A-N-2 | 135975 | 67359 | 65092 | 149.55 | 0.92 |
| Alc-L-A-N-3 | 99594 | 53545 | 49601 | 153.9 | 0.88 |
| Alc-L-A-S-1 | 91374 | 42509 | 39377 | 142.32 | 0.88 |
| Alc-L-A-S-2 | 127243 | 67038 | 63541 | 108.37 | 0.83 |
| Alc-L-A-S-3 | 765557 | 411719 | 395858 | 175.68 | 0.81 |
| Alc-L-B-N-1 | 103415 | 61280 | 53575 | 139.3 | 0.85 |
| Alc-L-B-N-2 | 91633 | 52799 | 48769 | 139.6 | 0.88 |
| Alc-L-B-N-3 | 86824 | 48821 | 45799 | 139.4 | 0.86 |
| Alc-L-B-S-1 | 90146 | 46447 | 43730 | 120.7 | 0.92 |
| Alc-L-B-S-2 | 88426 | 54028 | 47877 | 104.38 | 0.82 |
| Alc-L-B-S-3 | 94251 | 57544 | 52993 | 94.49 | 0.83 |
| Alc-L-C-N-1 | 114360 | 54255 | 49766 | 215.06 | 0.93 |
| Alc-L-C-N-2 | 87039 | 45478 | 39120 | 172.75 | 0.93 |
| Alc-L-C-N-3 | 99206 | 52625 | 48149 | 126.39 | 0.9 |
| Alc-L-C-S-1 | 80739 | 46944 | 43692 | 115.72 | 0.81 |
| Alc-L-C-S-2 | 104264 | 59926 | 56266 | 163.05 | 0.86 |
| Alc-L-C-S-3 | 97731 | 54273 | 50176 | 135.94 | 0.86 |
| Alc-L-D-N-1 | 95419 | 36185 | 34794 | 401.21 | 0.97 |
| Alc-L-D-N-2 | 89655 | 35957 | 34856 | 364.88 | 0.98 |
| Alc-L-D-N-3 | 84221 | 36459 | 34626 | 421.78 | 0.97 |
| Alc-L-D-S-1 | 103224 | 45935 | 45077 | 252.21 | 0.95 |
| Alc-L-D-S-2 | 115842 | 56950 | 54405 | 287.86 | 0.92 |
| Alc-L-D-S-3 | 120267 | 50989 | 48266 | 380.43 | 0.97 |
| Alc-L-F-N-1 | 97091 | 35418 | 33104 | 393.62 | 0.97 |
| Alc-L-F-N-2 | 108577 | 50825 | 48790 | 364.16 | 0.97 |
| Alc-L-F-N-3 | 103152 | 50436 | 48277 | 229.49 | 0.94 |
| Alc-L-F-S-1 | 112869 | 47215 | 45911 | 231.54 | 0.95 |
| Alc-L-F-S-2 | 70676 | 32869 | 31994 | 202.65 | 0.95 |
| Alc-L-F-S-3 | 94753 | 41820 | 40130 | 354.14 | 0.97 |
| Alc-L-G-N-1 | 122343 | 79195 | 78005 | 174.58 | 0.88 |
| Alc-L-G-N-2 | 100830 | 54322 | 50944 | 246.38 | 0.96 |
| Alc-L-G-N-3 | 126311 | 63688 | 60576 | 207.91 | 0.94 |
| Alc-L-G-S-1 | 113706 | 56332 | 54559 | 223.77 | 0.97 |
| Alc-L-G-S-2 | 109013 | 39056 | 36394 | 323.37 | 0.92 |
| Alc-L-G-S-3 | 104116 | 43298 | 39068 | 200.33 | 0.97 |
| Alc-R-A-N-1 | 69140 | 21833 | 21536 | 510.25 | 0.98 |
| Alc-R-A-N-2 | 63523 | 16862 | 16661 | 423.65 | 0.96 |
| Alc-R-A-N-3 | 79077 | 21308 | 21001 | 596.87 | 0.98 |
| Alc-R-A-S-1 | 70737 | 25214 | 25157 | 384.12 | 0.87 |
| Alc-R-A-S-2 | 78255 | 23696 | 23366 | 403.07 | 0.94 |

| | | | | | |
|---|---|---|---|---|---|
| Alc-R-A-S-3 | 62798 | 25870 | 25455 | 294.85 | 0.86 |
| Alc-R-B-N-1 | 78711 | 25022 | 24866 | 319.87 | 0.92 |
| Alc-R-B-N-2 | 497020 | 116622 | 114645 | 684.73 | 0.97 |
| Alc-R-B-N-3 | 75377 | 15699 | 15373 | 866.21 | 0.99 |
| Alc-R-B-S-1 | 78438 | 27960 | 27782 | 523.85 | 0.99 |
| Alc-R-B-S-2 | 93028 | 32442 | 31937 | 524.75 | 0.95 |
| Alc-R-B-S-3 | 69482 | 37352 | 37352 | 371.66 | 0.96 |
| Alc-R-C-N-1 | 75980 | 21034 | 20785 | 529.14 | 0.95 |
| Alc-R-C-N-2 | 61851 | 14803 | 14741 | 277.13 | 0.96 |
| Alc-R-C-N-3 | 101605 | 22929 | 22512 | 805.02 | 0.99 |
| Alc-R-C-S-1 | 89494 | 33761 | 33371 | 665.86 | 0.97 |
| Alc-R-C-S-2 | 90750 | 32287 | 31669 | 624.45 | 0.97 |
| Alc-R-C-S-3 | 82081 | 22532 | 21801 | 659.79 | 0.99 |
| Alc-R-D-N-1 | 78196 | 25124 | 24833 | 721.53 | 0.99 |
| Alc-R-D-N-2 | 79173 | 26300 | 25162 | 602.98 | 0.99 |
| Alc-R-D-N-3 | 85766 | 24792 | 24589 | 498.23 | 0.97 |
| Alc-R-D-S-1 | 82115 | 31837 | 31307 | 613.41 | 0.96 |
| Alc-R-D-S-2 | 72173 | 23593 | 22944 | 350.68 | 0.96 |
| Alc-R-D-S-3 | 63604 | 24681 | 24386 | 537.66 | 0.97 |
| Alc-R-F-N-1 | 67109 | 21401 | 20756 | 568.48 | 0.98 |
| Alc-R-F-N-2 | 94409 | 33069 | 32493 | 365.54 | 0.96 |
| Alc-R-F-N-3 | 94099 | 27336 | 26615 | 643 | 0.98 |
| Alc-R-F-S-1 | 76614 | 30363 | 29677 | 551.68 | 0.99 |
| Alc-R-F-S-2 | 714617 | 270014 | 264636 | 649.94 | 0.95 |
| Alc-R-F-S-3 | 85876 | 44771 | 44383 | 322.02 | 0.69 |
| Alc-R-G-N-1 | 786348 | 344621 | 344227 | 951.84 | 0.98 |
| Alc-R-G-N-2 | 587775 | 232663 | 231464 | 642.98 | 0.69 |
| Alc-R-G-N-3 | 83652 | 25326 | 24573 | 625.01 | 0.99 |
| Alc-R-G-S-1 | 147075 | 41255 | 40419 | 566.18 | 0.96 |
| Alc-R-G-S-2 | 63275 | 17422 | 17076 | 429.51 | 0.97 |
| Alc-R-G-S-3 | 73107 | 21320 | 20966 | 490.26 | 0.98 |
| Dry-F-D-N-1 | 125039 | 85346 | 84282 | 71.81 | 0.39 |
| Dry-F-D-N-2 | 107386 | 67824 | 66191 | 141.49 | 0.89 |
| Dry-F-D-N-3 | 110554 | 62705 | 61056 | 144.64 | 0.74 |
| Dry-F-D-S-1 | 115996 | 83660 | 82210 | 105.34 | 0.78 |
| Dry-F-D-S-2 | 89649 | 49077 | 45645 | 251.11 | 0.94 |
| Dry-F-D-S-3 | 94566 | 47564 | 44767 | 249.18 | 0.88 |
| Dry-F-E-N-1 | 125515 | 85890 | 84292 | 97.82 | 0.84 |
| Dry-F-E-N-2 | 94513 | 67774 | 66590 | 55.08 | 0.88 |
| Dry-F-E-N-3 | 108739 | 77334 | 75597 | 130.36 | 0.8 |
| Dry-F-E-S-1 | 122191 | 61138 | 59629 | 86.07 | 0.94 |
| Dry-F-E-S-2 | 115903 | 71909 | 69745 | 145.77 | 0.83 |
| Dry-F-E-S-3 | 108447 | 72648 | 71474 | 95.21 | 0.93 |
| Dry-F-G-N-1 | 109234 | 76269 | 75499 | 64.49 | 0.8 |
| Dry-F-G-N-2 | 110750 | 66766 | 65513 | 92.69 | 0.94 |
| Dry-F-G-N-3 | 101943 | 65871 | 64843 | 65.92 | 0.82 |

| | | | | | |
|---|---|---|---|---|---|
| Dry-F-G-S-1 | 89266 | 56614 | 55825 | 56.84 | 0.87 |
| Dry-F-G-S-2 | 96809 | 50806 | 50368 | 151.09 | 0.95 |
| Dry-F-G-S-3 | 104303 | 68972 | 68070 | 75.74 | 0.9 |
| Dry-L-D-N-1 | 110271 | 74386 | 73742 | 236.94 | 0.5 |
| Dry-L-D-N-2 | 126545 | 59754 | 59153 | 402.68 | 0.82 |
| Dry-L-D-N-3 | 108121 | 63753 | 63615 | 158.28 | 0.5 |
| Dry-L-D-S-1 | 90770 | 37476 | 36843 | 308.08 | 0.97 |
| Dry-L-D-S-2 | 83507 | 32965 | 32289 | 340.73 | 0.98 |
| Dry-L-D-S-3 | 113237 | 40539 | 39835 | 308.11 | 0.94 |
| Dry-L-E-N-1 | 80478 | 37326 | 36422 | 132.76 | 0.91 |
| Dry-L-E-N-2 | 86826 | 50566 | 49126 | 142.87 | 0.93 |
| Dry-L-E-N-3 | 91767 | 50893 | 48891 | 141.83 | 0.88 |
| Dry-L-E-S-1 | 110393 | 56916 | 55372 | 164.25 | 0.93 |
| Dry-L-E-S-2 | 117528 | 54257 | 53044 | 161.94 | 0.9 |
| Dry-L-E-S-3 | 102381 | 49746 | 48901 | 165.72 | 0.85 |
| Dry-L-G-N-1 | 99342 | 67405 | 65091 | 145.76 | 0.72 |
| Dry-L-G-N-2 | 92338 | 50271 | 47862 | 136.17 | 0.95 |
| Dry-L-G-N-3 | 109392 | 54590 | 51744 | 174.07 | 0.96 |
| Dry-L-G-S-1 | 101153 | 50899 | 47442 | 138.81 | 0.95 |
| Dry-L-G-S-2 | 112323 | 52942 | 50401 | 224.55 | 0.97 |
| Dry-L-G-S-3 | 84447 | 40157 | 38159 | 194.65 | 0.97 |
| Dry-R-D-N-1 | 71284 | 15567 | 15302 | 554.64 | 0.97 |
| Dry-R-D-N-2 | 74947 | 18541 | 18375 | 491.4 | 0.93 |
| Dry-R-D-N-3 | 83395 | 17252 | 16959 | 644.29 | 0.98 |
| Dry-R-D-S-1 | 67312 | 16515 | 16329 | 364.43 | 0.97 |
| Dry-R-D-S-2 | 69039 | 14200 | 13869 | 516 | 0.99 |
| Dry-R-D-S-3 | 71758 | 15004 | 14709 | 680.92 | 0.99 |
| Dry-R-E-N-1 | 84963 | 23544 | 23397 | 598.18 | 0.97 |
| Dry-R-E-N-2 | 82234 | 22042 | 22003 | 877.99 | 0.99 |
| Dry-R-E-N-3 | 76291 | 20848 | 20724 | 502.48 | 0.82 |
| Dry-R-E-S-1 | 69804 | 14247 | 14160 | 548.2 | 0.99 |
| Dry-R-E-S-2 | 66312 | 19781 | 19659 | 545.37 | 0.91 |
| Dry-R-E-S-3 | 82137 | 16557 | 16447 | 675.52 | 0.99 |
| Dry-R-G-N-1 | 510153 | 191721 | 190433 | 796.07 | 0.96 |
| Dry-R-G-N-2 | 88195 | 21998 | 21526 | 523.11 | 0.97 |
| Dry-R-G-N-3 | 73961 | 16674 | 16438 | 622.62 | 0.99 |
| Dry-R-G-S-1 | 82673 | 25560 | 25381 | 439.11 | 0.96 |
| Dry-R-G-S-2 | 76713 | 23248 | 22964 | 406.77 | 0.95 |
| Dry-R-G-S-3 | 80148 | 21115 | 21002 | 537.9 | 0.98 |
| Geu-F-A-N-1 | 122585 | 54654 | 51964 | 408.57 | 0.97 |
| Geu-F-A-N-2 | 112719 | 52221 | 50040 | 513.46 | 0.97 |
| Geu-F-A-N-3 | 113025 | 49850 | 47958 | 446.74 | 0.95 |
| Geu-F-A-S-1 | 104738 | 56171 | 53833 | 120.26 | 0.85 |
| Geu-F-A-S-2 | 108310 | 61986 | 59310 | 149.54 | 0.81 |
| Geu-F-A-S-3 | 124272 | 68239 | 66217 | 146.3 | 0.83 |
| Geu-F-B-N-1 | 96852 | 41085 | 40047 | 541.15 | 0.95 |

| | | | | | |
|---|---|---|---|---|---|
| Geu-F-B-N-2 | 129009 | 64544 | 62131 | 230.91 | 0.89 |
| Geu-F-B-N-3 | 55002 | 25510 | 24575 | 275.82 | 0.97 |
| Geu-F-B-S-1 | 113850 | 49884 | 48372 | 608.02 | 0.97 |
| Geu-F-B-S-2 | 115444 | 55950 | 53474 | 510.12 | 0.96 |
| Geu-F-B-S-3 | 112739 | 58709 | 57092 | 295.45 | 0.9 |
| Geu-F-C-N-1 | 87462 | 38771 | 37892 | 164.86 | 0.93 |
| Geu-F-C-N-2 | 125091 | 58476 | 56509 | 260.64 | 0.91 |
| Geu-F-C-N-3 | 118194 | 68376 | 66888 | 135.1 | 0.57 |
| Geu-F-C-S-1 | 133461 | 70124 | 66466 | 253.66 | 0.67 |
| Geu-F-C-S-2 | 116689 | 57089 | 54073 | 184.57 | 0.9 |
| Geu-F-C-S-3 | 90360 | 45475 | 44397 | 83.05 | 0.59 |
| Geu-F-D-N-1 | 121487 | 49364 | 48627 | 141.11 | 0.94 |
| Geu-F-D-N-2 | 110042 | 40959 | 40430 | 217.22 | 0.97 |
| Geu-F-D-N-3 | 123601 | 46312 | 45437 | 135.67 | 0.95 |
| Geu-F-D-S-1 | 115198 | 46769 | 46114 | 142.01 | 0.93 |
| Geu-F-D-S-2 | 112567 | 54628 | 53873 | 161.94 | 0.95 |
| Geu-F-D-S-3 | 90925 | 35543 | 34961 | 189.75 | 0.95 |
| Geu-F-F-N-1 | 120377 | 41370 | 40410 | 141.5 | 0.89 |
| Geu-F-F-N-2 | 130093 | 49638 | 48237 | 143.11 | 0.89 |
| Geu-F-F-N-3 | 107142 | 59394 | 58749 | 86.85 | 0.63 |
| Geu-F-F-S-1 | 120794 | 54402 | 53870 | 145.22 | 0.62 |
| Geu-F-F-S-2 | 105697 | 38557 | 38451 | 60.8 | 0.61 |
| Geu-F-F-S-3 | 98992 | 54009 | 53898 | 55.64 | 0.35 |
| Geu-F-G-N-1 | 108772 | 72041 | 71607 | 82.17 | 0.83 |
| Geu-F-G-N-2 | 106152 | 69053 | 65727 | 102.26 | 0.76 |
| Geu-F-G-N-3 | 105297 | 66573 | 66034 | 86.04 | 0.83 |
| Geu-F-G-S-1 | 118392 | 71064 | 70941 | 206.39 | 0.92 |
| Geu-F-G-S-2 | 91482 | 59892 | 59798 | 119.83 | 0.86 |
| Geu-F-G-S-3 | 106685 | 70879 | 70612 | 156.47 | 0.74 |
| Geu-L-A-N-1 | 102747 | 60976 | 52387 | 107.33 | 0.82 |
| Geu-L-A-N-2 | 97056 | 56029 | 53818 | 130.56 | 0.87 |
| Geu-L-A-N-3 | 89390 | 51918 | 51086 | 111.41 | 0.77 |
| Geu-L-A-S-1 | 105403 | 63777 | 62534 | 76.05 | 0.81 |
| Geu-L-A-S-2 | 105184 | 67615 | 64096 | 97.84 | 0.79 |
| Geu-L-A-S-3 | 98816 | 62092 | 57410 | 91.9 | 0.78 |
| Geu-L-B-N-1 | 82256 | 45317 | 29717 | 295.46 | 0.94 |
| Geu-L-B-N-2 | 93119 | 57348 | 40512 | 104.61 | 0.86 |
| Geu-L-B-N-3 | 108404 | 69653 | 59163 | 81.89 | 0.85 |
| Geu-L-B-S-1 | 91671 | 57271 | 49204 | 151.14 | 0.86 |
| Geu-L-B-S-2 | 87767 | 48046 | 38197 | 211.95 | 0.93 |
| Geu-L-B-S-3 | 99744 | 60941 | 54289 | 107.29 | 0.89 |
| Geu-L-C-N-1 | 102006 | 66599 | 63554 | 60.68 | 0.64 |
| Geu-L-C-N-2 | 80131 | 44224 | 36746 | 138.09 | 0.9 |
| Geu-L-C-N-3 | 96337 | 55141 | 47508 | 168.6 | 0.91 |
| Geu-L-C-S-1 | 101029 | 71183 | 69209 | 76.41 | 0.72 |
| Geu-L-C-S-2 | 97777 | 55849 | 50828 | 179.31 | 0.88 |

| | | | | | |
|---|---|---|---|---|---|
| Geu-L-C-S-3 | 91496 | 59725 | 57223 | 116.64 | 0.85 |
| Geu-L-D-N-1 | 118656 | 61046 | 58685 | 276.4 | 0.9 |
| Geu-L-D-N-2 | 91097 | 46726 | 44734 | 237.72 | 0.9 |
| Geu-L-D-N-3 | 90992 | 51262 | 48353 | 183.84 | 0.86 |
| Geu-L-D-S-1 | 98881 | 55042 | 50196 | 117.68 | 0.85 |
| Geu-L-D-S-2 | 109688 | 60947 | 53363 | 188.84 | 0.88 |
| Geu-L-D-S-3 | 84223 | 45952 | 43158 | 127.24 | 0.89 |
| Geu-L-F-N-1 | 88306 | 49323 | 46391 | 159.85 | 0.88 |
| Geu-L-F-N-2 | 96901 | 60899 | 57323 | 118.26 | 0.81 |
| Geu-L-F-N-3 | 86410 | 25427 | 22535 | 110.66 | 0.93 |
| Geu-L-F-S-1 | 110460 | 57801 | 55154 | 193.35 | 0.89 |
| Geu-L-F-S-2 | 102766 | 48409 | 45470 | 252.94 | 0.95 |
| Geu-L-F-S-3 | 108452 | 56284 | 53239 | 152.87 | 0.9 |
| Geu-L-G-N-1 | 113803 | 51922 | 50798 | 186.7 | 0.85 |
| Geu-L-G-N-2 | 109698 | 48841 | 47907 | 174.56 | 0.94 |
| Geu-L-G-N-3 | 91940 | 50911 | 49173 | 160.18 | 0.9 |
| Geu-L-G-S-1 | 99569 | 46704 | 46035 | 341.25 | 0.92 |
| Geu-L-G-S-2 | 132091 | 63909 | 62328 | 298.67 | 0.97 |
| Geu-L-G-S-3 | 105338 | 67621 | 67133 | 118.78 | 0.37 |
| Geu-R-A-N-1 | 84950 | 26859 | 26779 | 518 | 0.99 |
| Geu-R-A-N-2 | 91359 | 26698 | 26481 | 655.49 | 0.98 |
| Geu-R-A-N-3 | 74056 | 20494 | 20087 | 384.96 | 0.95 |
| Geu-R-A-S-1 | 138510 | 75340 | 75224 | 622.56 | 0.98 |
| Geu-R-A-S-2 | 94882 | 34714 | 34696 | 429.46 | 0.96 |
| Geu-R-A-S-3 | 89648 | 31448 | 30852 | 359.93 | 0.97 |
| Geu-R-B-N-1 | 85360 | 37192 | 37175 | 370.55 | 0.98 |
| Geu-R-B-N-2 | 74175 | 31456 | 31392 | 372.81 | 0.98 |
| Geu-R-B-N-3 | 80207 | 30199 | 30155 | 487.34 | 0.99 |
| Geu-R-B-S-1 | 73250 | 28280 | 28262 | 480 | 0.99 |
| Geu-R-B-S-2 | 88945 | 32620 | 32605 | 560.51 | 0.97 |
| Geu-R-B-S-3 | 71578 | 23649 | 23292 | 400.12 | 0.91 |
| Geu-R-C-N-1 | 56439 | 16228 | 16017 | 326.87 | 0.97 |
| Geu-R-C-N-2 | 72240 | 21563 | 20989 | 450.36 | 0.99 |
| Geu-R-C-N-3 | 94871 | 29036 | 28562 | 637.4 | 0.98 |
| Geu-R-C-S-1 | 85719 | 25867 | 25584 | 544.18 | 0.99 |
| Geu-R-C-S-2 | 80926 | 27003 | 26524 | 623.95 | 0.99 |
| Geu-R-C-S-3 | 95899 | 30011 | 29515 | 622.46 | 0.98 |
| Geu-R-D-N-1 | 58472 | 19338 | 19168 | 507.27 | 0.99 |
| Geu-R-D-N-2 | 75757 | 19258 | 18907 | 492.84 | 0.99 |
| Geu-R-D-N-3 | 89536 | 29761 | 29553 | 562.79 | 0.99 |
| Geu-R-D-S-1 | 81789 | 35036 | 35022 | 552.63 | 0.98 |
| Geu-R-D-S-2 | 83914 | 29186 | 28886 | 588.11 | 0.99 |
| Geu-R-D-S-3 | 93922 | 35838 | 35319 | 745.14 | 0.99 |
| Geu-R-F-N-1 | 64590 | 19740 | 19326 | 423.5 | 0.96 |
| Geu-R-F-N-2 | 82693 | 30215 | 29886 | 460.35 | 0.98 |
| Geu-R-F-N-3 | 72501 | 28199 | 28102 | 454.2 | 0.98 |

| | | | | | |
|---|---|---|---|---|---|
| Geu-R-F-S-1 | 93402 | 32923 | 32363 | 595.09 | 0.99 |
| Geu-R-F-S-2 | 76967 | 22771 | 22439 | 503.61 | 0.98 |
| Geu-R-F-S-3 | 90926 | 31636 | 31364 | 490.35 | 0.99 |
| Geu-R-G-N-1 | 62010 | 43047 | 42794 | 96.15 | 0.87 |
| Geu-R-G-N-2 | 74128 | 25782 | 25183 | 485.04 | 0.98 |
| Geu-R-G-N-3 | 72580 | 30815 | 30788 | 160.2 | 0.72 |
| Geu-R-G-S-1 | 85128 | 26312 | 25901 | 527.19 | 0.99 |
| Geu-R-G-S-2 | 64355 | 21542 | 21084 | 480.78 | 0.98 |
| Geu-R-G-S-3 | 88205 | 31402 | 31012 | 483.11 | 0.95 |

**Table S4.** Analysis of variance (ANOVA) of linear models (LMs) on bacterial richness for the first dataset (**A**; *Alchemilla* sp. and *G. montanum* from six collection sites) and second dataset (**B**; *Alchemilla* sp., *D. octopetala,* and *G. montanum* from two collection sites) of samples from alpine Rosaceae plants. Results of the model performance to select the most appropriate model (i.e., model with either fixed effect or with interactions having the lowest root mean squared error (RMSE) for the analysis are shown in the top. A post-hoc analysis with estimated marginal mean (EMM) comparisons was carried-out to better highlight differences between levels in each factor (alpine Rosaceae plant, plant tissue, site, and exposure) ($P \leq 0.05$).

## A

**Summary of model performance**

| | | | |
|---|---|---|---|
| LM with fixed effects: | RMSE | R squared | MAE |
| | 60.38665 | 0.622515 | 45.60492 |
| LM with interactions: | RMSE | R squared | MAE |
| | 55.68044 | 0.70157 | 41.07274 |

| *p* value | Significance Code |
|---|---|
| > 0.05 | not significative (NS) |
| < 0.05 | * |
| < 0.01 | ** |
| < 0.001 | *** |

**LMs on bacterial richness**

**Analysis of Variance Table**

| ANOVA of LM based on fixed effects + interactions. | **Df** | **Sum Sq** | **Mean Sq** | **F** | **Pr(>F)** | **Significance** |
|---|---|---|---|---|---|---|
| alpine_Rosaceae_plant | 1 | 7077 | 7077 | 3.45 | 0.065 | NS |
| Tissue | 2 | 1191505 | 595753 | 290.76 | < 2.2e-16 | *** |
| Collection_site | 5 | 88702 | 17740 | 8.66 | 0.000 | *** |
| Exposure | 1 | 1687 | 1687 | 0.82 | 0.366 | NS |
| alpine_Rosaceae_plant:Tissue | 2 | 17773 | 8886 | 4.34 | 0.015 | * |
| Rosaceae_plant:site | 5 | 70795 | 14159 | 6.91 | 0.000 | *** |
| Tissue:site | 10 | 101637 | 10164 | 4.96 | 0.000 | *** |
| Rosaceae_plant:Exposure | 1 | 20113 | 20113 | 9.82 | 0.002 | ** |
| Tissue:Exposure | 2 | 8532 | 4266 | 2.08 | 0.128 | NS |
| Collection_site:Exposure | 5 | 28436 | 5687 | 2.78 | 0.020 | * |
| alpine_Rosaceae_plant:Tissue:Collection_site | 10 | 94371 | 9437 | 4.61 | 0.000 | *** |
| alpine_Rosaceae_plant:Tissue:Exposure | 2 | 15810 | 7905 | 3.86 | 0.023 | * |
| alpine_Rosaceae_plant:Collection_site:Exposure | 5 | 20942 | 4188 | 2.04 | 0.08 | NS |
| Tissue:Collection_site:Exposure | 10 | 31588 | 3159 | 1.54 | 0.13 | NS |
| alpine_Rosaceae_plant:Tissue:Collection_site:Exposure | 10 | 36011 | 3601 | 1.76 | 0.07 | NS |
| Residuals | 144 | 295050 | 2049 | | | |

**Estimated marginal mean (EMM) comparisons of bacterial richness between alpine Rosaceae plants**

| **Alpine Rosaceae plant** | **emmean** | **SE** | **df** | **lower.CL** | **upper.CL** | **.group** |
|---|---|---|---|---|---|---|
| *Geum montanum* | 180 | 4.35 | 144 | 171 | 190 | a |
| *Alchemilla* sp. | 192 | 4.35 | 144 | 182 | 202 | a |

Results are averaged over the levels of: tissue, collection site, exposure
Confidence level used: 0.95
Conf-level adjustment: bonferroni method for 2 estimates
significance level used: alpha = 0.05

**Estimated marginal mean (EMM) comparisons of bacterial richness between plant tissues**

| **Tissue** | **emmean** | **SE** | **df** | **lower.CL** | **upper.CL** | **.group** |
|---|---|---|---|---|---|---|
| Leaves | 129 | 5.33 | 144 | 116 | 141 | a |
| Flowers | 139 | 5.33 | 144 | 126 | 152 | a |
| Roots | 291 | 5.33 | 144 | 278 | 304 | b |

Results are averaged over the levels of: alpine Rosaceae plant, collection_site, exposure
Confidence level used: 0.95
Conf-level adjustment: bonferroni method for 3 estimates
P value adjustment: fdr method for 3 tests
significance level used: alpha = 0.05

**Estimated marginal mean (EMM) comparisons of bacterial richness between collection sites**

| **Collection site** | **emmean** | **SE** | **df** | **lower.CL** | **upper.CL** | **.group** |
|---|---|---|---|---|---|---|
| Site A | 165 | 7.54 | 144 | 145 | 185 | a |
| Site C | 173 | 7.54 | 144 | 153 | 194 | a |
| Site B | 182 | 7.54 | 144 | 162 | 203 | a |
| Site F | 183 | 7.54 | 144 | 163 | 203 | a |
| Site G | 184 | 7.54 | 144 | 164 | 204 | a |
| Site D | 229 | 7.54 | 144 | 209 | 249 | b |

Results are averaged over the levels of: alpine Rosaceae plant, tissue, exposure
Confidence level used: 0.95
Conf-level adjustment: bonferroni method for 6 estimates
P value adjustment: fdr method for 15 tests
significance level used: alpha = 0.05

**Estimated marginal mean (EMM) comparisons of bacterial richness between exposuress**

| Exposure | emmean | SE | df | lower.CL | upper.CL | .group |
|---|---|---|---|---|---|---|
| South | 183 | 4.35 | 144 | 174 | 193 | a |
| North | 189 | 4.35 | 144 | 179 | 199 | a |

Results are averaged over the levels of: alpine Rosaceae plant, tissue, collection site
Confidence level used: 0.95
Conf-level adjustment: bonferroni method for 2 estimates
significance level used: alpha = 0.05

## B

**Significance**

| *p* value | Code |
|---|---|
| > 0.05 | not significative (NS) |
| < 0.05 | * |
| < 0.01 | ** |
| < 0.001 | *** |

**Summary of model performance**

| | RMSE | R squared | MAE |
|---|---|---|---|
| LM with fixed effects: | 51.86608 | 0.673046 | 38.71479 |
| LM with interactions: | 51.95925 | 0.691129 | 39.89636 |

**LMs on bacterial richness**

**Analysis of Variance Table**

| ANOVA of LM based on fixed effects. | Df | Sum Sq | Mean Sq | F | Pr(>F) | Significance |
|---|---|---|---|---|---|---|
| alpine_Rosaceae_plant | 2 | 54923 | 27462 | 10.00 | 0.0001 | *** |
| Tissue | 2 | 457674 | 228837 | 83.33 | < 2.2e-16 | *** |
| collection_site | 1 | 35119 | 35119 | 12.79 | 0.0005 | *** |
| Exposure | 1 | 1675 | 1675 | 0.61 | 0.4366 | NS |
| Residuals | 101 | 277375 | 2746 | | | |

**Estimated marginal mean (EMM) comparisons of bacterial richness between alpine Rosaceae plants**

| Alpine Rosaceae plant | emmean | SE | df | lower.CL | upper.CL | .group |
|---|---|---|---|---|---|---|
| *Dryas octopetala* | 170 | 8.73 | 101 | 149 | 191 | a |
| *Geum montanum* | 172 | 8.73 | 101 | 151 | 194 | a |
| *Alchemilla* sp. | 219 | 8.73 | 101 | 198 | 240 | b |

Results are averaged over the levels of: tissue, collection site, exposure
Confidence level used: 0.95
Conf-level adjustment: bonferroni method for 3 estimates
P value adjustment: fdr method for 3 tests
significance level used: alpha = 0.05

**Estimated marginal mean (EMM) comparisons of bacterial richness between plant tissues**

| Tissue | emmean | SE | df | lower.CL | upper.CL | .group |
|---|---|---|---|---|---|---|
| Flowers | 127 | 8.73 | 101 | 105 | 148 | a |
| Leaves | 157 | 8.73 | 101 | 136 | 179 | b |
| Roots | 277 | 8.73 | 101 | 256 | 299 | c |

Results are averaged over the levels of: alpine Rosaceae plant, collection_site, exposure
Confidence level used: 0.95
Conf-level adjustment: bonferroni method for 3 estimates
P value adjustment: fdr method for 3 tests
significance level used: alpha = 0.05

**Estimated marginal mean (EMM) comparisons of bacterial richness between collection sites**

| Collection site | emmean | SE | df | lower.CL | upper.CL | .group |
|---|---|---|---|---|---|---|
| Site G | 169 | 7.13 | 101 | 153 | 185 | a |
| Site D | 205 | 7.13 | 101 | 189 | 221 | b |

Results are averaged over the levels of: alpine Rosaceae plant, tissue, exposure
Confidence level used: 0.95
Conf-level adjustment: bonferroni method for 2 estimates
significance level used: alpha = 0.05

**Estimated marginal mean (EMM) comparisons of bacterial richness between exposuress**

| Exposure | emmean | SE | df | lower.CL | upper.CL | .group |
|---|---|---|---|---|---|---|
| North | 183 | 7.13 | 101 | 167 | 199 | a |
| South | 191 | 7.13 | 101 | 175 | 207 | a |

Results are averaged over the levels of: alpine Rosaceae plant, tissue, collection site
Confidence level used: 0.95
Conf-level adjustment: bonferroni method for 2 estimates
significance level used: alpha = 0.05

**Table S5.** Analysis of variance (ANOVA) of linear models (LMs) on bacterial alpha-diversity for the first dataset (**A**; *Alchemilla* sp. and *G. montanum* from six collection sites) and second dataset (**B**; *Alchemilla* sp., *D. octopetala* and *G. montanum* from two collection sites) of samples from alpine Rosaceae plants. Results of the model performance to select the most appropriate model (i.e., model with either fixed effect or with interactions having the lowest root mean squared error (RMSE)) for the analysis are shown in the top. A post-hoc analysis with estimated marginal mean (EMM) comparisons was carried-out to better highlight differences between levels in each factor (alpine Rosaceae plant, plant tissue, collection site, and exposure) ($P \leq 0.05$).

| Significance | |
|---|---|
| ***p* value** | **Code** |
| > 0.05 | not significative (NS) |
| < 0.05 | * |
| < 0.01 | ** |
| < 0.001 | *** |

## A

**Summary of model performance**

| | RMSE | R squared | MAE |
|---|---|---|---|
| LM with fixed effects: | 0.09292824 | 0.2394924 | 0.063671 |
| LM with interactions: | 0.1037122 | 0.2822584 | 0.061857 |

**LMs on bacterial alpha-diversity**

| **Analysis of Variance Table** | **Df** | **Sum Sq** | **Mean Sq** | **F** | **Pr(>F)** | **Significance** |
|---|---|---|---|---|---|---|
| ANOVA of LM based on fixed effects. | | | | | | |
| alpine_Rosaceae_plant | 1 | 0.05611 | 0.05611 | 6.18 | 0.0137 | * |
| Tissue | 2 | 0.34113 | 0.170563 | 18.79 | 0.0000 | *** |
| Collection_site | 5 | 0.14496 | 0.028993 | 3.19 | 0.0084 | ** |
| Exposure | 1 | 0.06091 | 0.06091 | 6.71 | 0.0103 | * |
| Residuals | 206 | 1.86957 | 0.009076 | | | |

**Estimated marginal mean (EMM) comparisons of bacterial richness between alpine Rosaceae plants**

| **alpine Rosaceae plant** | **emmean** | **SE** | **df** | **lower.CL** | **upper.CL** | **.group** |
|---|---|---|---|---|---|---|
| *Geum montanum* | 0.882 | 0.00917 | 206 | 0.862 | 0.903 | a |
| *Alchemilla* sp. | 0.915 | 0.00917 | 206 | 0.894 | 0.935 | b |

Results are averaged over the levels of: tissue, collection site, exposure
Confidence level used: 0.95
Conf-level adjustment: bonferroni method for 2 estimates
significance level used: alpha = 0.05

**Estimated marginal mean (EMM) comparisons of bacterial richness between plant tissues**

| **Tissue** | **emmean** | **SE** | **df** | **lower.CL** | **upper.CL** | **.group** |
|---|---|---|---|---|---|---|
| Flowers | 0.864 | 0.0112 | 206 | 0.837 | 0.892 | a |
| Leaves | 0.877 | 0.0112 | 206 | 0.85 | 0.904 | a |
| Roots | 0.954 | 0.0112 | 206 | 0.927 | 0.981 | b |

Results are averaged over the levels of: alpine Rosaceae plant, collection site, exposure
Confidence level used: 0.95
Conf-level adjustment: bonferroni method for 3 estimates
P value adjustment: fdr method for 3 tests
significance level used: alpha = 0.05

**Estimated marginal mean (EMM) comparisons of bacterial richness between collection sites**

| **Collection site** | **emmean** | **SE** | **df** | **lower.CL** | **upper.CL** | **.group** |
|---|---|---|---|---|---|---|
| Site F | 0.878 | 0.0159 | 206 | 0.836 | 0.921 | a |
| Site A | 0.881 | 0.0159 | 206 | 0.839 | 0.923 | a |
| Site C | 0.882 | 0.0159 | 206 | 0.839 | 0.924 | a |
| Site G | 0.887 | 0.0159 | 206 | 0.844 | 0.929 | a |
| Site B | 0.914 | 0.0159 | 206 | 0.871 | 0.956 | ab |
| Site D | 0.95 | 0.0159 | 206 | 0.908 | 0.992 | b |

Results are averaged over the levels of: alpine Rosaceae plant, tissue, exposure
Confidence level used: 0.95
Conf-level adjustment: bonferroni method for 6 estimates
P value adjustment: fdr method for 15 tests
significance level used: alpha = 0.05

**Estimated marginal mean (EMM) comparisons of bacterial richness between exposures**

| **Exposure** | **emmean** | **SE** | **df** | **lower.CL** | **upper.CL** | **.group** |
|---|---|---|---|---|---|---|
| South | 0.882 | 0.00917 | 206 | 0.861 | 0.902 | a |
| North | 0.915 | 0.00917 | 206 | 0.895 | 0.936 | b |

Results are averaged over the levels of: alpine Rosaceae plant, tissue, collection site
Confidence level used: 0.95
Conf-level adjustment: bonferroni method for 2 estimates
significance level used: alpha = 0.05

**B**

| Significance | |
|---|---|
| ***p* value** | **Code** |
| > 0.05 | not significative (NS) |
| < 0.05 | * |
| < 0.01 | ** |
| < 0.001 | *** |

**Summary of model performance**

| | RMSE | R squared | MAE |
|---|---|---|---|
| LM with fixed effects: | 0.104554 | 0.2318533 | 0.069627 |
| LM with interactions: | 0.1081045 | 0.3456834 | 0.065596 |

**LMs on bacterial alpha-diversity**

| **Analysis of Variance Table** | **Df** | **Sum Sq** | **Mean Sq** | **F** | **Pr(>F)** | **Significance** |
|---|---|---|---|---|---|---|
| ANOVA of LM based on fixed effects. | | | | | | |
| alpine_Rosaceae_plant | 2 | 0.08091 | 0.040455 | 3.26 | 0.042 | * |
| Tissue | 2 | 0.12494 | 0.062469 | 5.04 | 0.008 | ** |
| Collection_site | 1 | 0.00455 | 0.004549 | 0.37 | 0.546 | NS |
| Exposure | 1 | 0.05273 | 0.052732 | 4.25 | 0.042 | * |
| Residuals | 101 | 1.25269 | 0.012403 | | | |

**Estimated marginal mean (EMM) comparisons of bacterial richness between alpine Rosaceae plants**

| **alpine Rosaceae plant** | **emmean** | **SE** | **df** | **lower.Cl** | **upper.CL** | **.group** |
|---|---|---|---|---|---|---|
| *Dryas octopetala* | 0.877 | 0.0186 | 101 | 0.832 | 0.922 | a |
| *Geum montanum* | 0.894 | 0.0186 | 101 | 0.849 | 0.939 | ab |
| *Alchemilla* sp. | 0.942 | 0.0186 | 101 | 0.897 | 0.987 | b |

Results are averaged over the levels of: tissue, collection site, exposure
Confidence level used: 0.95
Conf-level adjustment: bonferroni method for 2 estimates
P value adjustment: fdr method for 3 tests
significance level used: alpha = 0.05

**Estimated marginal mean (EMM) comparisons of bacterial richness between plant tissues**

| **Tissue** | **emmean** | **SE** | **df** | **lower.Cl** | **upper.CL** | **.group** |
|---|---|---|---|---|---|---|
| Leaves | 0.879 | 0.0186 | 101 | 0.834 | 0.925 | a |
| Flowers | 0.881 | 0.0186 | 101 | 0.836 | 0.926 | a |
| Roots | 0.952 | 0.0186 | 101 | 0.907 | 0.998 | b |

Results are averaged over the levels of: alpine Rosaceae plant, collection site, exposure
Confidence level used: 0.95
Conf-level adjustment: bonferroni method for 3 estimates
P value adjustment: fdr method for 3 tests
significance level used: alpha = 0.05

**Estimated marginal mean (EMM) comparisons of bacterial richness between collection sites**

| **Collection site** | **emmean** | **SE** | **df** | **lower.Cl** | **upper.CL** | **.group** |
|---|---|---|---|---|---|---|
| Site G | 0.898 | 0.0152 | 101 | 0.863 | 0.932 | a |
| Site D | 0.911 | 0.0152 | 101 | 0.876 | 0.945 | a |

Results are averaged over the levels of: alpine Rosaceae plant, tissue, exposure
Confidence level used: 0.95
Conf-level adjustment: bonferroni method for 2 estimates
significance level used: alpha = 0.05

**Estimated marginal mean (EMM) comparisons of bacterial richness between exposures**

| **Exposure** | **emmean** | **SE** | **df** | **lower.Cl** | **upper.CL** | **.group** |
|---|---|---|---|---|---|---|
| North | 0.882 | 0.0152 | 101 | 0.848 | 0.917 | a |
| South | 0.926 | 0.0152 | 101 | 0.892 | 0.961 | b |

Results are averaged over the levels of: alpine Rosaceae plant, tissue, collection site
Confidence level used: 0.95
Conf-level adjustment: bonferroni method for 2 estimates
significance level used: alpha = 0.05

**Table S6**. Canonical analysis of principal coordinates (CAP) permutation test results for the first dataset (**A**; *Alchemilla* sp. and *G. montanum* from six collection sites) and second dataset (**B**; *Alchemilla* sp., *D. octopetala,* and *G. montanum* from two collection sites) of samples from alpine Rosaceae plants.

## A

| Analysis of Variance Table | Df | SumOfSqs | F | Pr(>F) | Significance |
|---|---|---|---|---|---|
| alpine_Rosaceae_plant | 1 | 3.802 | 15.3128 | 0.001 | *** |
| Tissue | 2 | 12.399 | 24.9697 | 0.001 | *** |
| Collection_site | 5 | 5.412 | 4.3597 | 0.001 | *** |
| Exposure | 1 | 0.453 | 1.8237 | 0.007 | ** |
| alpine_Rosaceae_plant:Tissue | 2 | 3.568 | 7.1862 | 0.001 | *** |
| alpine_Rosaceae_plant:Collection_site | 5 | 3.627 | 2.922 | 0.001 | *** |
| Tissue:Collection_site | 10 | 6.333 | 2.5506 | 0.001 | *** |
| alpine_Rosaceae_plant:Exposure | 1 | 0.356 | 1.4346 | 0.035 | * |
| Tissue:Exposure | 2 | 0.714 | 1.4388 | 0.012 | * |
| Collection_site:Exposure | 5 | 2.035 | 1.639 | 0.001 | *** |
| alpine_Rosaceae_plant:Tissue:Collection_site | 10 | 5.099 | 2.0538 | 0.001 | *** |
| alpine_Rosaceae_plant:Tissue:Exposure | 2 | 0.648 | 1.3057 | 0.043 | * |
| alpine_Rosaceae_plant:Collection_site:Exposure | 5 | 1.906 | 1.5356 | 0.001 | *** |
| Tissue:Collection_site:Exposure | 10 | 3.122 | 1.2574 | 0.002 | ** |
| alpine_Rosaceae_plant:Tissue:Collection_site:Exposure | 10 | 3.059 | 1.2323 | 0.006 | ** |
| Residual | 144 | 35.753 | | | |

Number of permutations: 999

| Significance | |
|---|---|
| ***p* value** | **Code** |
| > 0.05 | not significative (NS) |
| < 0.05 | * |
| < 0.01 | ** |
| < 0.001 | *** |

## B

| Analysis of Variance Table | Df | SumOfSqs | F | Pr(>F) | Significance |
|---|---|---|---|---|---|
| alpine_Rosaceae_plant | 2 | 3.1935 | 8.0062 | 0.001 | *** |
| Tissue | 2 | 7.053 | 17.6821 | 0.001 | *** |
| Collection_site | 1 | 1.8338 | 9.1947 | 0.001 | *** |
| Exposure | 1 | 0.4003 | 2.007 | 0.005 | ** |
| alpine_Rosaceae_plant:Tissue | 4 | 3.4905 | 4.3755 | 0.001 | *** |
| alpine_Rosaceae_plant:Collection_site | 2 | 1.6164 | 4.0524 | 0.001 | *** |
| Tissue:Collection_site | 2 | 1.8879 | 4.7332 | 0.001 | *** |
| alpine_Rosaceae_plant:Exposure | 2 | 0.7584 | 1.9013 | 0.002 | ** |
| Tissue:Exposure | 2 | 0.5746 | 1.4406 | 0.025 | * |
| collection_Collection_site:Exposure | 1 | 0.4202 | 2.1069 | 0.003 | ** |
| alpine_Rosaceae_plant:Tissue:Collection_site | 4 | 2.3812 | 2.9849 | 0.001 | *** |
| alpine_Rosaceae_plant:Tissue:Expositi | 4 | 1.1802 | 1.4794 | 0.003 | ** |
| alpine_Rosaceae_plant:Collection_site:Exposure | 2 | 0.7866 | 1.9721 | 0.001 | *** |
| Tissue:Collection_site:Exposure | 2 | 0.5444 | 1.3648 | 0.036 | * |
| alpine_Rosaceae_plant:Tissue:Collection_site:Exposure | 4 | 1.1592 | 1.4531 | 0.001 | *** |
| Residual | 72 | 14.3596 | | | |

Number of permutations: 999

| Significance | |
|---|---|
| ***p* value** | **Code** |
| > 0.05 | not significative (NS) |
| < 0.05 | * |
| < 0.01 | ** |
| < 0.001 | *** |

**Table S7.** Factors affecting the beta-diversity of endophytic bacterial communities of alpine Rosaceae plants. Results of a permutational multivariate analysis of variances (PERMANOVA) on bacterial Bray–Curtis dissimilarities for the first dataset (**A**; *Alchemilla* sp. and *G. montanum* from six collection sites) and second dataset (**B**; *Alchemilla* sp., *D. octopetala,* and *G. montanum* from two collection sites) of samples from alpine Rosaceae plants. The percentage of explained variance (R2 = contribution coefficient) of each factor to the community beta-diversity is reported. Intra-factor pairwise comparisons between levels in each factor (alpine Rosaceae plant, plant tissue, collection site, and exposure) was carried-out using the pairwise.perm.manova function from the RVAideMemoire R package with P-value adjustment using Benjamini-Hochberg method ($P \leq 0.05$). Multivariate homogeneity of group dispersions was performed using the betadisper function from the vegan package, followed by ANOVA. Intra-factor pairwise comparisons between factor levels was carried-out using permutational test (999 iterations) with the permutest function from the vegan R package.

## A

**PERMANOVA**

| | Df | SumOfSqs | R2 | Explained variance (%) | F | Pr(>F) | Significance |
|---|---|---|---|---|---|---|---|
| alpine_Rosaceae_plant | 1 | 2.96 | 0.04 | 3.67 | 15.49 | 0.001 | *** |
| Tissue | 2 | 16.98 | 0.21 | 21.06 | 44.45 | 0.001 | *** |
| Collection_site | 5 | 5.98 | 0.07 | 7.41 | 6.26 | 0.001 | *** |
| Exposure | 1 | 0.46 | 0.01 | 0.57 | 2.40 | 0.005 | ** |
| alpine_Rosaceae_plant:Tissue | 2 | 3.16 | 0.04 | 3.93 | 8.28 | 0.001 | *** |
| alpine_Rosaceae_plant:Collection_site | 5 | 3.22 | 0.04 | 3.99 | 3.37 | 0.001 | *** |
| Tissue:Collection_site | 10 | 6.53 | 0.08 | 8.10 | 3.42 | 0.001 | *** |
| alpine_Rosaceae_plant:Exposure | 1 | 0.26 | 0.00 | 0.33 | 1.38 | 0.117 | NS |
| Tissue:Exposure | 2 | 0.64 | 0.01 | 0.79 | 1.67 | 0.010 | ** |
| Collection_site:Exposure | 5 | 1.70 | 0.02 | 2.11 | 1.78 | 0.001 | *** |
| alpine_Rosaceae_plant:Tissue:Collection_site | 10 | 4.26 | 0.05 | 5.28 | 2.23 | 0.001 | *** |
| alpine_Rosaceae_plant:Tissue:Exposure | 2 | 0.54 | 0.01 | 0.67 | 1.41 | 0.047 | * |
| alpine_Rosaceae_plant:Collection_site:Exposure | 5 | 1.61 | 0.02 | 2.00 | 1.69 | 0.001 | *** |
| Tissue:Collection_site:Exposure | 10 | 2.35 | 0.03 | 2.92 | 1.23 | 0.030 | * |
| alpine_Rosaceae_plant:Tissue:Collection_site:Exposure | 10 | 2.47 | 0.03 | 3.06 | 1.29 | 0.008 | ** |
| Residual | 144 | 27.51 | 0.34 | 34.12 | NA | NA | |
| Total | 215 | 80.61 | 1.00 | 100.00 | NA | NA | |

| Significance | |
|---|---|
| ***p* value** | **Code** |
| > 0.05 | not significative (NS) |
| < 0.05 | * |
| < 0.01 | ** |
| < 0.001 | *** |

**Pairwise comparisons using permutation MANOVAs on a distance matrix**

| **Tissue** | Roots | Flowers |
|---|---|---|
| Flowers | 0.001 | - |
| Leaves | 0.001 | 0.001 |

| **alpine Rosaceae plant** | ALCHEMILLA |
|---|---|
| GEUM | 0.001 |

| **Collection site** | Site A | Site B | Site C | Site D | Site F |
|---|---|---|---|---|---|
| Site B | 0.36 | - | - | - | - |
| Site C | 0.1768 | 0.1327 | - | - | - |
| Site D | 0.0043 | 0.0025 | 0.012 | - | - |
| Site F | 0.0338 | 0.01 | 0.0338 | 0.0075 | - |
| Site G | 0.0025 | 0.0025 | 0.0025 | 0.0025 | 0.0025 |

| **Exposure** | North |
|---|---|
| South | 0.2 |

**Dispersion**

**Tissue**

| Analysis of Variance Table | Df | Sum Sq | Mean Sq | F value | Pr(>F) | Significance |
|---|---|---|---|---|---|---|
| Groups | 2 | 1.481 | 0.7405 | 48.802 | <2.20 ×1016 | *** |
| Residuals | 213 | 3.232 | 0.01517 | | | |

---

Pairwise comparisons:

| (Observed p-value below diagonal, permuted p-value above diagonal) | Roots | Flowers | Leaves |
|---|---|---|---|
| Roots | - | 0.88100 | 0.00100 |

| | | | |
|---|---|---|---|
| Flowers | 0.88474 | - | 0.00100 |
| Leaves | 0.00000 | 0.00000 | - |

**Alpine Rosaceae plant**

| Analysis of Variance Table | **Df** | **Sum Sq** | **Mean Sq** | **F value** | **Pr(>F)** |
|---|---|---|---|---|---|
| Groups | 1 | 0.00961 | 0.00961 | 1.4956 | 0.2227 |
| Residuals | 214 | 1.37454 | 0.00642 | | |

**Collection site**

| Analysis of Variance Table | **Df** | **Sum Sq** | **Mean Sq** | **F value** | **Pr(>F)** | **Significance** |
|---|---|---|---|---|---|---|
| Groups | 5 | 0.10024 | 0.02005 | 2.2881 | 0.04721 | * |
| Residuals | 210 | 1.83997 | 0.00876 | | | |

---
Pairwise comparisons:
(Observed p-value below diagonal, permuted p-value above diagonal)

| | Site A | Site B | Site C | Site D | Site F | Site G |
|---|---|---|---|---|---|---|
| Site A | - | 0.69600 | 0.79800 | 0.10700 | 0.17500 | 0.79200 |
| Site B | 0.68238 | - | 0.40700 | 0.13500 | 0.04000 | 0.88200 |
| Site C | 0.77780 | 0.41158 | - | 0.01600 | 0.21300 | 0.51000 |
| Site D | 0.09486 | 0.13970 | 0.01238 | - | 0.00100 | 0.10800 |
| Site F | 0.18596 | 0.03663 | 0.19701 | 0.00004 | - | 0.06900 |
| Site G | 0.78163 | 0.88565 | 0.51438 | 0.11092 | 0.06059 | - |

**Exposure**

| Analysis of Variance Table | **Df** | **Sum Sq** | **Mean Sq** | **F value** | **Pr(>F)** |
|---|---|---|---|---|---|
| Groups | 1 | 0.00212 | 0.00212 | 0.3382 | 0.5615 |
| Residuals | 214 | 1.33835 | 0.00625 | | |

---

## B

**PERMANOVA**

| | **Df** | **SumOfSqs** | **R2** | **Explained variance (%)** | **F** | **Pr(>F)** | **Significance** |
|---|---|---|---|---|---|---|---|
| alpine_Rosaceae_plant | 2 | 2.51 | 0.06 | 6.38 | 7.52 | 0.001 | *** |
| Tissue | 2 | 7.11 | 0.18 | 18.06 | 21.28 | 0.001 | *** |
| Collection_site | 1 | 2.70 | 0.07 | 6.87 | 16.18 | 0.001 | *** |
| Exposure | 1 | 0.38 | 0.01 | 0.97 | 2.28 | 0.004 | ** |
| alpine_Rosaceae_plant:Tissue | 4 | 3.20 | 0.08 | 8.13 | 4.79 | 0.001 | *** |
| alpine_Rosaceae_plant:Collection_site | 2 | 1.83 | 0.05 | 4.66 | 5.49 | 0.001 | *** |
| Tissue:Collection_site | 2 | 2.30 | 0.06 | 5.85 | 6.89 | 0.001 | *** |
| alpine_Rosaceae_plant:Exposure | 2 | 0.77 | 0.02 | 1.96 | 2.31 | 0.001 | *** |
| Tissue:Exposure | 2 | 0.48 | 0.01 | 1.22 | 1.44 | 0.037 | * |
| Collection_site:Exposure | 1 | 0.40 | 0.01 | 1.02 | 2.40 | 0.001 | *** |
| alpine_Rosaceae_plant:Tissue:Collection_site | 4 | 2.14 | 0.05 | 5.43 | 3.20 | 0.001 | *** |
| alpine_Rosaceae_plant:Tissue:Exposure | 4 | 1.22 | 0.03 | 3.10 | 1.83 | 0.001 | *** |
| alpine_Rosaceae_plant:Collection_site:Exposure | 2 | 0.75 | 0.02 | 1.91 | 2.25 | 0.001 | *** |
| Tissue:Collection_site:Exposure | 2 | 0.44 | 0.01 | 1.12 | 1.32 | 0.103 | NS |
| alpine_Rosaceae_plant:Tissue:Collection_site:Exposure | 4 | 1.09 | 0.03 | 2.77 | 1.63 | 0.003 | *** |
| Residual | 72 | 12.04 | 0.31 | 30.56 | NA | NA | |
| Total | 107 | 39.39 | 1.00 | 100.00 | NA | NA | |

| **Significance** | |
|---|---|
| ***p* value** | **Code** |
| > 0.05 | not significative (NS) |
| < 0.05 | * |
| < 0.01 | ** |
| < 0.001 | *** |

**Pairwise comparisons using permutation MANOVAs on a distance matrix**

| **Tissue** | Roots | Flowers |
|---|---|---|
| Flowers | 0.001 | - |
| Leaves | 0.001 | 0.001 |

| **alpine Rosaceae plant** | ALCHEM | GEUM |
|---|---|---|
| GEUM | 0.001 | - |
| DRYAS | 0.001 | 0.001 |

| **Collection site** | Site D |
|---|---|
| Site G | 0.001 |

| **Exposure** | North |
|---|---|
| South | 0.36 |

**Dispersion**

**Tissue**

| Analysis of Variance Table | **Df** | **Sum Sq** | **Mean Sq** | **F value** | **Pr(>F)** | **Significance** |
|---|---|---|---|---|---|---|
| Groups | 2 | 0.08639 | 0.04319 | 5.1188 | 0.007564 | ** |
| Residuals | 105 | 0.886 | 0.00844 | | | |

---

Pairwise comparisons:

| (Observed p-value below diagonal, permuted p-value ab | Roots | Flowers | Leaves |
|---|---|---|---|
| Roots | - | 0.002 | 0.014 |
| Flowers | 0.0004 | - | 0.826 |
| Leaves | 0.0129 | 0.8361536 | - |

**Alpine Rosaceae plant**

| Analysis of Variance Table | **Df** | **Sum Sq** | **Mean Sq** | **F value** | **Pr(>F)** |
|---|---|---|---|---|---|
| Groups | 2 | 0.01943 | 0.00972 | 1.7653 | 0.1762 |
| Residuals | 105 | 0.57786 | 0.0055 | | |

---

Pairwise comparisons:

| (Observed p-value below diagonal, permuted p-value ab | ALCHEM | GEUM | DRYAS |
|---|---|---|---|
| ALCHEMILLA | - | 0.092 | 0.129 |
| GEUM | 0.085 | - | 0.743 |
| DRYAS | 0.1221 | 0.739 | - |

**Collection site**

| Analysis of Variance Table | **Df** | **Sum Sq** | **Mean Sq** | **F value** | **Pr(>F)** |
|---|---|---|---|---|---|
| Groups | 1 | 0.01519 | 0.01519 | 1.7888 | 0.1839 |
| Residuals | 106 | 0.90015 | 0.00849 | | |

**Exposure**

| Analysis of Variance Table | **Df** | **Sum Sq** | **Mean Sq** | **F value** | **Pr(>F)** |
|---|---|---|---|---|---|
| Groups | 1 | 0.01396 | 0.01396 | 3.3746 | 0.06901 |
| Residuals | 106 | 0.43852 | 0.00414 | | |

**Table S8.** Factors affecting the beta-diversity of endophytic bacterial communities of alpine Rosaceae plants identified through multivariate generalized linear models (mGLMs). Analysis was performed for the first dataset (**A**; *Alchemilla* sp. and *G. montanum* from six collection sites) and second dataset (**B**; *Alchemilla* sp., *D. octopetala,* and *G. montanum* from two collection sites) of samples from alpine Rosaceae plants. Rarefied bacterial count data of amplicon sequence variants (ASVs) with 0.25 occupancy were used in this analysis. An analysis of deviance was calculated with a likelihood-ratio test using a permutational test (999 iterations, Monte Carlo resampling).

## A

| Analysis of Deviance Table | Res.Df | Df.diff | Dev | Pr(>Dev) | Significance |
|---|---|---|---|---|---|
| (Intercept) | 215 | NA | NA | NA | |
| Tissue | 213 | 2 | 4193.2234 | 0.001 | *** |
| alpine_Rosaceae_plant | 212 | 1 | 2156.20024 | 0.001 | *** |
| Collection_site | 207 | 5 | 2898.53549 | 0.001 | *** |
| Exposure | 206 | 1 | 308.506982 | 0.001 | *** |
| Tissue:alpine_Rosaceae_plant | 204 | 2 | 989.042854 | 0.001 | *** |
| Tissue:Collection_site | 194 | 10 | 4724.22074 | 0.001 | *** |
| alpine_Rosaceae_plant:Collection_site | 189 | 5 | 2145.40819 | 0.001 | *** |
| Tissue:Exposure | 187 | 2 | 632.611463 | 0.001 | *** |
| alpine_Rosaceae_plant:Exposure | 186 | 1 | 2742.01611 | 0.001 | *** |
| Collection_site:Exposure | 181 | 5 | 6564.20305 | 0.001 | *** |
| Tissue:alpine_Rosaceae_plant:Collection_site | 171 | 10 | 3237.36558 | 0.001 | *** |
| Tissue:alpine_Rosaceae_plant:Exposure | 169 | 2 | 14939.6809 | 0.001 | *** |
| Tissue:Collection_site:Exposure | 159 | 10 | 2591.03456 | 0.001 | *** |
| alpine_Rosaceae_plant:Collection_site:Exposure | 154 | 5 | 17493.8143 | 0.001 | *** |
| Tissue:alpine_Rosaceae_plant:Collection_site:Exposure | 144 | 10 | 119650.87 | 1 | |

---
Model: mva ~ metadata$Tissue * metadata$Rosaceae_plant * metadata$Collection_site * metadata$Exposure
P-value calculated using 1000 iterations via parametric resampling.

| *p* value | Significance Code |
|---|---|
| > 0.05 | not significative (NS) |
| < 0.05 | * |
| < 0.01 | ** |
| < 0.001 | *** |

## B

| Analysis of Deviance Table | Res.Df | Df.diff | Dev | Pr(>Dev) | Significance |
|---|---|---|---|---|---|
| (Intercept) | 107 | NA | NA | NA | |
| Tissue | 105 | 2 | 2864.44591 | 0.000999 | *** |
| alpine_Rosaceae_plant | 103 | 2 | 1935.42575 | 0.000999 | *** |
| Collection_site | 102 | 1 | 1419.73039 | 0.000999 | *** |
| Exposure | 101 | 1 | 279.499418 | 0.000999 | *** |
| Tissue:alpine_Rosaceae_plant | 97 | 4 | 2106.77445 | 0.000999 | *** |
| Tissue:Collection_site | 95 | 2 | 1369.75322 | 0.000999 | *** |
| alpine_Rosaceae_plant:Collection_site | 93 | 2 | 1299.86159 | 0.000999 | *** |
| Tissue:Exposure | 91 | 2 | 566.469828 | 0.000999 | *** |
| alpine_Rosaceae_plant:Exposure | 89 | 2 | 865.360818 | 0.000999 | *** |
| Collection_site:Exposure | 88 | 1 | 588.505219 | 0.000999 | *** |
| Tissue:alpine_Rosaceae_plant:Collection_site | 84 | 4 | 147112.161 | 0.000999 | *** |
| Tissue:alpine_Rosaceae_plant:Exposure | 80 | 4 | 8445.6261 | 0.000999 | *** |
| Tissue:Collection_site:Exposure | 78 | 2 | 1002.92604 | 0.000999 | *** |
| alpine_Rosaceae_plant:Collection_site:Exposure | 76 | 2 | 1093.32339 | 0.000999 | *** |
| Tissue:alpine_Rosaceae_plant:Collection_site:Exposure | 72 | 4 | 446.41476 | 0.001998 | ** |

Model: mva_s2 ~ metadata$Tissue * metadata$Rosaceae_plant * metadata$Collection_site * metadata$Exposure
P-value calculated using 1000 iterations via parametric resampling.

| *p* value | Significance Code |
|---|---|
| > 0.05 | not significative (NS) |
| < 0.05 | * |
| < 0.01 | ** |
| < 0.001 | *** |

**Table S9.** Factors affecting the beta-diversity of endophytic bacterial communities in flowers, leaves and roots of alpine Rosaceae plants. Results of a permutational multivariate analysis of variances (PERMANOVA) partitioning test by tissues on bacterial Bray–Curtis dissimilarities for the first dataset (**A**; *Alchemilla* sp. and *G. montanum* from six collection sites) and second dataset (**B**; *Alchemilla* sp., *D. octopetala* and *G. montanum* from two collection sites) of samples from alpine Rosaceae plants. Pairwise comparisons between levels within each factor was performed using permutation MANOVAs. The percentage of explained variance (R2 = contribution coefficient) of each factor to the community beta-diversity as well as the host-environment effects index (HEEI = relative contribution of alpine Rosaceae plant/relative contribution of collection site) are reported.

## A

**Flowers**

| Factor | Df | SumOfSqs | R2 | Explained variance (%) | F | Pr(>F) | HEEI |
|---|---|---|---|---|---|---|---|
| alpine_Rosaceae_plant | 1 | 1.263663258 | 0.050741 | 5.07 | 6.13799 | 0.000999 | 0.1952 |
| Collection_site | 5 | 6.473088867 | 0.259922 | 25.99 | 6.288346 | 0.000999 | |
| Exposure | 1 | 0.596887772 | 0.023968 | 2.40 | 2.899262 | 0.000999 | |
| alpine_Rosaceae_plant:Collection_site | 5 | 3.017632251 | 0.121171 | 12.12 | 2.931508 | 0.000999 | |
| alpine_Rosaceae_plant:Exposure | 1 | 0.294776947 | 0.011837 | 1.18 | 1.43182 | 0.098901 | |
| Collection_site:Exposure | 5 | 1.618100283 | 0.064974 | 6.50 | 1.571919 | 0.005994 | |
| alpine_Rosaceae_plant:Collection_site:Exposure | 5 | 1.757771989 | 0.070582 | 7.06 | 1.707605 | 0.000999 | |
| Residual | 48 | 9.882035107 | 0.396806 | 39.68 | NA | NA | |
| Total | 71 | 24.90395647 | 1 | 100.00 | NA | NA | |

**Leaves**

| Factor | Df | SumOfSqs | R2 | Explained variance (%) | F | Pr(>F) | HEEI |
|---|---|---|---|---|---|---|---|
| alpine_Rosaceae_plant | 1 | 1.126919252 | 0.080853 | 8.09 | 8.517931 | 0.000999 | 0.3703 |
| Collection_site | 5 | 3.042974947 | 0.218324 | 21.83 | 4.600125 | 0.000999 | |
| Exposure | 1 | 0.180482701 | 0.012949 | 1.29 | 1.364196 | 0.143856 | |
| alpine_Rosaceae_plant:Collection_site | 5 | 1.475097465 | 0.105834 | 10.58 | 2.229934 | 0.000999 | |
| alpine_Rosaceae_plant:Exposure | 1 | 0.197842544 | 0.014195 | 1.42 | 1.495412 | 0.097902 | |
| Collection_site:Exposure | 5 | 0.856635934 | 0.061461 | 6.15 | 1.294993 | 0.07992 | |
| alpine_Rosaceae_plant:Collection_site:Exposure | 5 | 0.707544502 | 0.050764 | 5.08 | 1.069609 | 0.323676 | |
| Residual | 48 | 6.350383277 | 0.45562 | 45.56 | NA | NA | |
| Total | 71 | 13.93788062 | 1 | 100.00 | NA | NA | |

**Roots**

| Factor | Df | SumOfSqs | R2 | Explained variance (%) | F | Pr(>F) | HEEI |
|---|---|---|---|---|---|---|---|
| alpine_Rosaceae_plant | 1 | 3.73243442 | 0.150539 | 15.05 | 15.89153 | 9.99E-04 | 1.2474 |
| Collection_site | 5 | 2.992206557 | 0.120684 | 12.07 | 2.547975 | 9.99E-04 | |
| Exposure | 1 | 0.318732066 | 0.012855 | 1.29 | 1.357061 | 0.112887 | |
| alpine_Rosaceae_plant:Collection_site | 5 | 2.981283297 | 0.120243 | 12.02 | 2.538673 | 0.000999 | |
| alpine_Rosaceae_plant:Exposure | 1 | 0.308826507 | 0.012456 | 1.25 | 1.314886 | 0.106893 | |
| Collection_site:Exposure | 5 | 1.575087853 | 0.063528 | 6.35 | 1.341246 | 0.010989 | |
| alpine_Rosaceae_plant:Collection_site:Exposure | 5 | 1.611487714 | 0.064996 | 6.50 | 1.372242 | 0.006993 | |
| Residual | 48 | 11.27373133 | 0.4547 | 45.47 | NA | NA | |
| Total | 71 | 24.79378974 | 1 | 100.00 | NA | NA | |

## B

**Flowers**

| Factor | Df | SumOfSqs | R2 | Explained variance (%) | F | Pr(>F) | HEEI |
|---|---|---|---|---|---|---|---|
| alpine_Rosaceae_plant | 2 | 1.58436776 | 0.160994 | 16.10 | 6.807856 | 0.000999 | 0.5308 |
| Collection_site | 1 | 2.984953296 | 0.303313 | 30.33 | 25.65204 | 0.000999 | |
| Exposure | 1 | 0.134285751 | 0.013645 | 1.36 | 1.154023 | 0.28971 | |
| alpine_Rosaceae_plant:Collection_site | 2 | 1.266487213 | 0.128693 | 12.87 | 5.441958 | 0.000999 | |
| alpine_Rosaceae_plant:Exposure | 2 | 0.485756102 | 0.04936 | 4.94 | 2.087241 | 0.01998 | |
| Collection_site:Exposure | 1 | 0.150938689 | 0.015337 | 1.53 | 1.297134 | 0.200799 | |
| alpine_Rosaceae_plant:Collection_site:Exposure | 2 | 0.441651853 | 0.044878 | 4.49 | 1.89773 | 0.043956 | |
| Residual | 24 | 2.792716634 | 0.283779 | 28.38 | NA | NA | |
| Total | 35 | 9.841157299 | 1 | 100.00 | NA | NA | |

**Leaves**

| Factor | Df | SumOfSqs | R2 | Explained variance (%) | F | Pr(>F) | HEEI |
|---|---|---|---|---|---|---|---|
| alpine_Rosaceae_plant | 2 | 1.739450691 | 0.169397 | 16.94 | 5.483332 | 0.000999 | 1.4838 |
| Collection_site | 1 | 1.172305053 | 0.114166 | 11.42 | 7.390997 | 0.000999 | |
| Exposure | 1 | 0.340610324 | 0.033171 | 3.32 | 2.147436 | 0.008991 | |

| | | | | | | | |
|---|---|---|---|---|---|---|---|
| alpine_Rosaceae_plant:Collection_site | 2 | 1.38458614 | 0.134839 | 13.48 | 4.36468 | 0.000999 | |
| alpine_Rosaceae_plant:Exposure | 2 | 0.766165522 | 0.074613 | 7.46 | 2.415211 | 0.000999 | |
| Collection_site:Exposure | 1 | 0.358808197 | 0.034943 | 3.49 | 2.262168 | 0.008991 | |
| alpine_Rosaceae_plant:Collection_site:Exposure | 2 | 0.69984245 | 0.068155 | 6.82 | 2.206138 | 0.002997 | |
| Residual | 24 | 3.806701559 | 0.370718 | 37.07 | NA | NA | |
| Total | 35 | 10.26846994 | 1 | 100.00 | NA | NA | |

**Roots**

| **Factor** | **Df** | **SumOfSqs** | **R2** | **Explained variance (%)** | **F** | **Pr(>F)** | **HEEI** |
|---|---|---|---|---|---|---|---|
| alpine_Rosaceae_plant | 2 | 2.394838899 | 0.196786 | 19.68 | 5.285618 | 0.000999 | 2.8144 |
| Collection_site | 1 | 0.850917857 | 0.069921 | 6.99 | 3.7561 | 0.000999 | |
| Exposure | 1 | 0.387662768 | 0.031855 | 3.19 | 1.711211 | 0.012987 | |
| alpine_Rosaceae_plant:Collection_site | 2 | 1.323497383 | 0.108753 | 10.88 | 2.921074 | 0.000999 | |
| alpine_Rosaceae_plant:Exposure | 2 | 0.740039996 | 0.06081 | 6.08 | 1.633333 | 0.001998 | |
| Collection_site:Exposure | 1 | 0.331996986 | 0.027281 | 2.73 | 1.465493 | 0.058941 | |
| alpine_Rosaceae_plant:Collection_site:Exposure | 2 | 0.703753653 | 0.057828 | 5.78 | 1.553246 | 0.008991 | |
| Residual | 24 | 5.437030631 | 0.446766 | 44.68 | NA | NA | |
| Total | 35 | 12.16973817 | 1 | 100.00 | NA | NA | |

**Table S10.** Main ecological processes governing the assembly of flower-, leaf-, and root-associated communities of alpine Rosaceae plants analyzed through Infer Community Assembly Mechanisms by Phylogenetic-bin-based null model (iCAMP). (**A**) Relative abundances of bins and their top amplicon sequence variants (ASVs). (**B**) Relative importance of each bin to the different processes governing community assembly. Abbreviations; Drift and others (DR), homogenizing dispersal (HD), dispersal limitation (DL), homogeneous selection (HoS), heterogeneous selection (HeS), are reported. The dominant process and its relative importance are shown for each bin.

**A**

| Bin | Bin relative abundance | TopTaxon ID | TopTaxon relative |
|---|---|---|---|
| Bin1 | 0.001883058 | ASV_308 | 0.194918651 |
| Bin2 | 0.001471939 | ASV_812 | 0.064385601 |
| Bin3 | 0.036954736 | ASV_3 | 0.963636537 |
| Bin4 | 0.002808554 | ASV_227 | 0.155323157 |
| Bin5 | 0.004862705 | ASV_71 | 0.467007966 |
| Bin6 | 0.011021974 | ASV_54 | 0.365698369 |
| Bin7 | 0.000657055 | ASV_688 | 0.167194651 |
| Bin8 | 0.011239677 | ASV_27 | 0.494668537 |
| Bin9 | 0.005988476 | ASV_28 | 0.928344429 |
| Bin10 | 0.025611644 | ASV_13 | 0.389167742 |
| Bin11 | 0.015715382 | ASV_16 | 0.447130495 |
| Bin12 | 0.017182605 | ASV_35 | 0.312483872 |
| Bin13 | 0.006758633 | ASV_47 | 0.578042423 |
| Bin14 | 0.007579158 | ASV_34 | 0.660795049 |
| Bin15 | 0.00908344 | ASV_110 | 0.180183333 |
| Bin16 | 0.005808651 | ASV_147 | 0.209127212 |
| Bin17 | 0.00899403 | ASV_37 | 0.650441803 |
| Bin18 | 0.00237193 | ASV_352 | 0.216967534 |
| Bin19 | 0.013539289 | ASV_76 | 0.183233353 |
| Bin20 | 0.007126387 | ASV_39 | 0.623153633 |
| Bin21 | 0.004927231 | ASV_65 | 0.570572267 |
| Bin22 | 0.038345439 | ASV_4 | 0.866652931 |
| Bin23 | 0.000612056 | ASV_1152 | 0.137056724 |
| Bin24 | 0.000780253 | ASV_424 | 0.274340661 |
| Bin25 | 0.009072588 | ASV_145 | 0.125267406 |
| Bin26 | 0.018772656 | ASV_64 | 0.14526069 |
| Bin27 | 0.001492078 | ASV_759 | 0.131592951 |
| Bin28 | 0.002130559 | ASV_319 | 0.270864878 |
| Bin29 | 0.021603434 | ASV_14 | 0.414887159 |
| Bin30 | 0.018975223 | ASV_17 | 0.462841629 |
| Bin31 | 0.109704048 | ASV_1 | 0.614799503 |
| Bin32 | 0.007549234 | ASV_82 | 0.402149961 |
| Bin33 | 0.003827979 | ASV_228 | 0.384021685 |
| Bin34 | 0.000659574 | ASV_1163 | 0.172431705 |
| Bin35 | 0.003655697 | ASV_149 | 0.569611694 |
| Bin36 | 0.004653452 | ASV_191 | 0.231097629 |
| Bin37 | 0.005940112 | ASV_153 | 0.193917477 |
| Bin38 | 0.011546998 | ASV_26 | 0.551367401 |

| | | | |
|---|---|---|---|
| Bin39 | 0.002385122 | ASV_531 | 0.174036251 |
| Bin40 | 0.00323391 | ASV_260 | 0.154109736 |
| Bin41 | 0.000680004 | ASV_876 | 0.193409398 |
| Bin42 | 0.063342828 | ASV_5 | 0.47751695 |
| Bin43 | 0.078497874 | ASV_6 | 0.330685534 |
| Bin44 | 0.044290079 | ASV_11 | 0.273000894 |
| Bin45 | 0.069553109 | ASV_2 | 0.496537983 |
| Bin46 | 0.001049412 | ASV_1140 | 0.072195289 |
| Bin47 | 0.016022662 | ASV_15 | 0.4376715 |
| Bin48 | 0.000743965 | ASV_343 | 0.536028463 |
| Bin49 | 0.000879539 | ASV_733 | 0.176684621 |
| Bin50 | 0.00531276 | ASV_68 | 0.354987898 |
| Bin51 | 0.001868626 | ASV_517 | 0.145108548 |
| Bin52 | 0.041690386 | ASV_9 | 0.380779713 |
| Bin53 | 0.000849897 | ASV_900 | 0.305319453 |
| Bin54 | 0.007812662 | ASV_208 | 0.147439664 |
| Bin55 | 0.005916739 | ASV_189 | 0.175616283 |
| Bin56 | 0.001427686 | ASV_627 | 0.152064926 |
| Bin57 | 0.007859012 | ASV_51 | 0.647946438 |
| Bin58 | 0.003052285 | ASV_184 | 0.400472567 |
| Bin59 | 0.003319528 | ASV_275 | 0.269371328 |
| Bin60 | 0.002806548 | ASV_585 | 0.101443262 |
| Bin61 | 0.007576065 | ASV_342 | 0.088964318 |
| Bin62 | 0.009360816 | ASV_88 | 0.241515052 |
| Bin63 | 0.001116104 | ASV_508 | 0.179076488 |
| Bin64 | 0.005691968 | ASV_109 | 0.470710891 |
| Bin65 | 0.013907956 | ASV_19 | 0.602586589 |
| Bin66 | 0.001275081 | ASV_280 | 0.543527647 |
| Bin67 | 0.001842451 | ASV_473 | 0.229177859 |
| Bin68 | 0.001302122 | ASV_734 | 0.159831163 |
| Bin69 | 0.002204886 | ASV_957 | 0.062721069 |
| Bin70 | 0.001588111 | ASV_504 | 0.203635876 |
| Bin71 | 0.001168095 | ASV_521 | 0.188355055 |
| Bin72 | 0.001458063 | ASV_919 | 0.080529362 |
| Bin73 | 0.001166838 | ASV_1108 | 0.078321049 |
| Bin74 | 0.008698084 | ASV_77 | 0.450534192 |
| Bin75 | 0.010633173 | ASV_176 | 0.114267632 |
| Bin76 | 0.008250875 | ASV_45 | 0.460023905 |
| Bin77 | 0.004169758 | ASV_173 | 0.3169328 |
| Bin78 | 0.002005756 | ASV_555 | 0.191764204 |
| Bin79 | 0.001022077 | ASV_696 | 0.202599745 |
| Bin80 | 0.013504761 | ASV_66 | 0.342386067 |
| Bin81 | 0.002080519 | ASV_262 | 0.340229224 |
| Bin82 | 0.004048548 | ASV_211 | 0.280093408 |
| Bin83 | 0.000768064 | ASV_815 | 0.121556749 |
| Bin84 | 0.001159503 | ASV_239 | 0.530912548 |
| Bin85 | 0.002977671 | ASV_301 | 0.15147752 |
| Bin86 | 0.001424978 | ASV_684 | 0.15330809 |

| | | | |
|---|---|---|---|
| Bin87 | 0.003180415 | ASV_154 | 0.474460266 |
| Bin88 | 0.014183571 | ASV_69 | 0.288187353 |
| Bin89 | 0.005297151 | ASV_160 | 0.274187199 |
| Bin90 | 0.00571871 | ASV_111 | 0.278575927 |
| Bin91 | 0.002076249 | ASV_402 | 0.196525582 |
| Bin92 | 0.003945225 | ASV_152 | 0.300595592 |
| Bin93 | 0.001789399 | ASV_361 | 0.243554068 |
| Bin94 | 0.004424898 | ASV_224 | 0.139504468 |
| Bin95 | 0.001730714 | ASV_419 | 0.203948186 |
| Bin96 | 0.000635881 | ASV_764 | 0.152424556 |
| Bin97 | 0.001520296 | ASV_467 | 0.209334798 |
| Bin98 | 0.000875066 | ASV_516 | 0.161090592 |
| Bin99 | 0.001249508 | ASV_390 | 0.254299313 |
| Bin100 | 0.001520008 | ASV_282 | 0.446594401 |
| Bin101 | 0.014375765 | ASV_93 | 0.157537832 |
| Bin102 | 0.00196697 | ASV_215 | 0.193315858 |
| Bin103 | 0.000878451 | ASV_804 | 0.19287361 |
| Bin104 | 0.000726833 | ASV_436 | 0.284354978 |

**B**

| Group | Index | bin22 | bin23 | bin24 | bin25 | bin26 | bin27 | bin28 | bin29 | bin30 | bin31 | bin32 | bin33 | bin34 | bin35 | bin36 | bin37 |
|---|---|---|---|---|---|---|---|---|---|---|---|---|---|---|---|---|---|
| Flowers | HeS | 0 | 0 | 0 | 0.01188 | 0.001416 | 0 | 0 | 0.04597 | 0 | 0 | 0 | 0 | 0 | 0 | 0 | 0 |
| Flowers | HoS | 0.43024049 | 0.001179812 | 0.08621 | 0.1531 | 0.136744 | 0.14381 | 0.00069 | 0.18206 | 0.01133 | 0.12784 | 0.09033 | 0.03483 | 0.03371 | 0.016924 | 0.006616 | 0.479697 |
| Flowers | DL | 0 | 0.998820188 | 0.91379 | 0.26284 | 0 | 0.85619 | 0.99931 | 0 | 0 | 0.00704 | 0.13924 | 0.96517 | 0.96629 | 0.983076 | 0.993384 | 0.518862 |
| Flowers | HD | 0 | 0 | 0 | 0 | 0 | 0 | 0 | 0 | 0 | 0 | 0 | 0 | 0 | 0 | 0 | 0 |
| Flowers | DR | 0.56975951 | 0 | 0 | 0.57218 | 0.86184 | 0 | 0 | 0.77196 | 0.98867 | 0.86512 | 0.77043 | 0 | 0 | 0 | 0 | 0.00144 |
| Flowers | DominantProcess | DR | DL | DL | DR | DR | DL | DL | DR | DR | DR | DR | DL | DL | DL | DL | DL |
| Flowers | DominantProcessImportance | 0.56975951 | 0.998820188 | 0.91379 | 0.57218 | 0.86184 | 0.85619 | 0.99931 | 0.77196 | 0.98867 | 0.86512 | 0.77043 | 0.96517 | 0.96629 | 0.983076 | 0.993384 | 0.518862 |
| Flowers | DominantProcessPvalue | 0.117 | 0 | 0 | 0.062 | 0 | 0 | 0 | 0 | 0 | 0 | 0.003 | 0 | 0.015 | 0 | 0.002 | 0.486 |
| Leaves | HeS | 0 | 0 | 0 | 0.03612 | 0 | 0.00311 | 0 | 0 | 0.00237 | 0 | 0 | 0 | 0 | 0 | 0 | 0 |
| Leaves | HoS | 0.1501989 | 0.007185963 | 0.14152 | 0.12438 | 0.343964 | 0.31642 | 0.01696 | 0.16697 | 0.09407 | 0.8994 | 0.39718 | 0.53824 | 0.16098 | 0.71507 | 0.069844 | 0.53965 |
| Leaves | DL | 0 | 0.992814037 | 0.85848 | 0.48275 | 0 | 0.68047 | 0.98304 | 0 | 0 | 0 | 0.20226 | 0.46176 | 0.83902 | 0.28493 | 0.930156 | 0.460309 |
| Leaves | HD | 0 | 0 | 0 | 0 | 0 | 0 | 0 | 0 | 0 | 0 | 0 | 0 | 0 | 0 | 0 | 0 |
| Leaves | DR | 0.8498011 | 0 | 0 | 0.35675 | 0.656036 | 0 | 0 | 0.83303 | 0.90356 | 0.1006 | 0.40057 | 0 | 0 | 0 | 0 | 4.05E-05 |
| Leaves | DominantProcess | DR | DL | DL | DL | DR | DL | DL | DR | DR | HoS | DR | HoS | DL | HoS | DL | HoS |
| Leaves | DominantProcessImportance | 0.8498011 | 0.992814037 | 0.85848 | 0.48275 | 0.656036 | 0.68047 | 0.98304 | 0.83303 | 0.90356 | 0.8994 | 0.40057 | 0.53824 | 0.83902 | 0.71507 | 0.930156 | 0.53965 |
| Leaves | DominantProcessPvalue | 0 | 0 | 0 | 0.276 | 0 | 0.139 | 0 | 0 | 0 | 0 | 0.443 | 0.514 | 0 | 0.282 | 0.015 | 0.412 |
| Roots | HeS | 0.00027317 | 0 | 0 | 0 | 0 | 0 | 0.00042 | 0.00307 | 0 | 0 | 0.03112 | 0 | 0 | 0 | 0 | 0 |
| Roots | HoS | 0.22894107 | 0.122413042 | 0.00372 | 0.02391 | 0.298105 | 0.10759 | 0.25824 | 0.11003 | 0.01799 | 0.57013 | 0.26568 | 0.08742 | 0.32155 | 0.095874 | 0.289976 | 0.143103 |
| Roots | DL | 0 | 0.877586958 | 0.99628 | 0.16695 | 0 | 0.89241 | 0.74134 | 0.00475 | 0 | 0 | 0.41173 | 0.91258 | 0.67845 | 0.904087 | 0.709986 | 0.856707 |
| Roots | HD | 0 | 0 | 0 | 0 | 0 | 0 | 0 | 0 | 0 | 0 | 0 | 0 | 0 | 0 | 0 | 0 |
| Roots | DR | 0.77078576 | 0 | 0 | 0.80913 | 0.701895 | 0 | 0 | 0.88216 | 0.98201 | 0.42987 | 0.29147 | 0 | 0 | 3.93E-05 | 3.81E-05 | 0.000191 |
| Roots | DominantProcess | DR | DL | DL | DR | DR | DL | DL | DR | DR | HoS | DL | DL | DL | DL | DL | DL |
| Roots | DominantProcessImportance | 0.77078576 | 0.877586958 | 0.99628 | 0.80913 | 0.701895 | 0.89241 | 0.74134 | 0.88216 | 0.98201 | 0.57013 | 0.41173 | 0.91258 | 0.67845 | 0.904087 | 0.709986 | 0.856707 |
| Roots | DominantProcessPvalue | 0 | 0 | 0 | 0 | 0.001 | 0 | 0 | 0 | 0 | 0.186 | 0.241 | 0 | 0.04 | 0 | 0.03 | 0 |
| **Group** | **Index** | **bin38** | **bin39** | **bin40** | **bin41** | **bin42** | **bin43** | **bin44** | **bin45** | **bin46** | **bin47** | **bin48** | **bin49** | **bin50** | **bin51** | **bin52** | **bin53** |
| Flowers | HeS | 0 | 0 | 0 | 0 | 0 | 0.05416 | 0 | 0 | 0.01535 | 0.00063 | 0 | 0 | 0 | 0.020355 | 0 | 0 |

| | | | | | | | | | | | | | | | | | |
|---|---|---|---|---|---|---|---|---|---|---|---|---|---|---|---|---|---|
| Flowers | HoS | 0.05013753 | 0 | 0.12854 | 0.02173 | 0.072391 | 0.12539 | 0.85565 | 0.59799 | 0.08505 | 0.40537 | 0.00442 | 0.00966 | 0.0836 | 0.060551 | 0.136014 | 0.010622 |
| Flowers | DL | 0.51940226 | 1 | 0.87146 | 0.97827 | 0 | 0.00196 | 0 | 0 | 0.8996 | 0.3109 | 0.99558 | 0.99034 | 0.9164 | 0.919094 | 0.071906 | 0.989378 |
| Flowers | HD | 0 | 0 | 0 | 0 | 0 | 0 | 0 | 0 | 0 | 0 | 0 | 0 | 0 | 0 | 0 | 0 |
| Flowers | DR | 0.43046021 | 0 | 0 | 0 | 0.927609 | 0.81849 | 0.14435 | 0.40201 | 0 | 0.2831 | 0 | 0 | 0 | 0 | 0.792079 | 0 |
| Flowers | DominantProcess | DL | DL | DL | DL | DR | DR | HoS | HoS | DL | HoS | DL | DL | DL | DL | DR | DL |
| Flowers | DominantProcessImportance | 0.51940226 | 1 | 0.87146 | 0.97827 | 0.927609 | 0.81849 | 0.85565 | 0.59799 | 0.8996 | 0.40537 | 0.99558 | 0.99034 | 0.9164 | 0.919094 | 0.792079 | 0.989378 |
| Flowers | DominantProcessPvalue | 0.432 | 0.014 | 0 | 0 | 0 | 0 | 0 | 0.031 | 0 | 0.458 | 0.001 | 0.003 | 0 | 0 | 0 | 0.045 |
| Leaves | HeS | 0 | 0 | 0 | 0 | 0 | 0.04363 | 0 | 0 | 0.00057 | 0 | 0 | 0 | 0 | 0 | 0 | 0 |
| Leaves | HoS | 0.72094266 | 0 | 0.67053 | 0.56148 | 0.046154 | 0.22494 | 0.76658 | 0.29449 | 0.01773 | 0.33174 | 0.30187 | 0.28116 | 0.74522 | 0.287146 | 0.030298 | 0.021633 |
| Leaves | DL | 0.17211971 | 1 | 0.32947 | 0.43852 | 0 | 0 | 0 | 0.00293 | 0.9817 | 0.62303 | 0.69813 | 0.71884 | 0.25478 | 0.712854 | 0 | 0.978367 |
| Leaves | HD | 0 | 0 | 0 | 0 | 0 | 0 | 0 | 0 | 0 | 0 | 0 | 0 | 0 | 0 | 0 | 0 |
| Leaves | DR | 0.10693764 | 0 | 0 | 0 | 0.953846 | 0.73143 | 0.23342 | 0.70258 | 0 | 0.04523 | 0 | 0 | 0 | 0 | 0.969702 | 0 |
| Leaves | DominantProcess | HoS | DL | HoS | HoS | DR | DR | HoS | DR | DL | DL | DL | DL | HoS | DL | DR | DL |
| Leaves | DominantProcessImportance | 0.72094266 | 1 | 0.67053 | 0.56148 | 0.953846 | 0.73143 | 0.76658 | 0.70258 | 0.9817 | 0.62303 | 0.69813 | 0.71884 | 0.74522 | 0.712854 | 0.969702 | 0.978367 |
| Leaves | DominantProcessPvalue | 0.036 | 0.017 | 0.168 | 0.414 | 0 | 0 | 0 | 0 | 0 | 0.06 | 0.01 | 0 | 0.048 | 0.106 | 0 | 0.012 |
| Roots | HeS | 0 | 0 | 0 | 0.00566 | 0 | 0.01089 | 0.00673 | 0.00031 | 0.0273 | 0.01454 | 0.01048 | 0 | 0 | 0 | 0 | 0 |
| Roots | HoS | 0.17110051 | 0.038566041 | 0.38155 | 0.10397 | 0.151018 | 0.15231 | 0.16804 | 0.14173 | 0.18454 | 0.15016 | 0.37958 | 0.03532 | 0.03305 | 0.077742 | 0.143242 | 0.504972 |
| Roots | DL | 0.5535621 | 0.961433959 | 0.61845 | 0.89037 | 0 | 0 | 0 | 0 | 0.78816 | 0.02816 | 0.60994 | 0.96468 | 0.96695 | 0.922258 | 0.002676 | 0.495028 |
| Roots | HD | 0 | 0 | 0 | 0 | 0 | 0 | 0 | 0 | 0 | 0 | 0 | 0 | 0 | 0 | 0 | 0 |
| Roots | DR | 0.2753374 | 0 | 0 | 0 | 0.848982 | 0.8368 | 0.82523 | 0.85796 | 0 | 0.80714 | 0 | 0 | 0 | 0 | 0.854082 | 0 |
| Roots | DominantProcess | DL | DL | DL | DL | DR | DR | DR | DR | DL | DR | DL | DL | DL | DL | DR | HoS |
| Roots | DominantProcessImportance | 0.5535621 | 0.961433959 | 0.61845 | 0.89037 | 0.848982 | 0.8368 | 0.82523 | 0.85796 | 0.78816 | 0.80714 | 0.60994 | 0.96468 | 0.96695 | 0.922258 | 0.854082 | 0.504972 |
| Roots | DominantProcessPvalue | 0.087 | 0 | 0.05 | 0 | 0 | 0 | 0 | 0 | 0 | 0 | 0.188 | 0 | 0.002 | 0 | 0 | 0.544 |

| Group | Index | bin54 | bin55 | bin56 | bin57 | bin58 | bin59 | bin60 | bin61 | bin62 | bin63 | bin64 | bin65 | bin66 | bin67 | bin68 | bin69 |
|---|---|---|---|---|---|---|---|---|---|---|---|---|---|---|---|---|---|
| Flowers | HeS | 0 | 0 | 0 | 0 | 0 | 0 | 0.0012 | 0 | 0 | 0 | 0 | 0 | 0 | 0 | 0 | 0 |
| Flowers | HoS | 0.0039303 | 0.022029747 | 0.46159 | 0.16386 | 0.275745 | 0.04094 | 0.28217 | 0.11939 | 0.02737 | 0.02355 | 0.02253 | 0.48019 | 0.10387 | 0.027597 | 0.670141 | 0.268272 |
| Flowers | DL | 0 | 0.112549838 | 0.53841 | 0 | 0.622 | 0.85405 | 0.71663 | 0.31367 | 0 | 0.97645 | 0.301 | 0 | 0.89613 | 0.972403 | 0.329859 | 0.731728 |
| Flowers | HD | 0 | 0 | 0 | 0 | 0 | 0 | 0 | 0 | 0 | 0 | 0 | 0 | 0 | 0 | 0 | 0 |
| Flowers | DR | 0.9960697 | 0.865420415 | 0 | 0.83614 | 0.102254 | 0.10501 | 0 | 0.56694 | 0.97263 | 0 | 0.67647 | 0.51981 | 0 | 0 | 0 | 0 |
| Flowers | DominantProcess | DR | DR | DL | DR | DL | DL | DL | DR | DR | DL | DR | DR | DL | DL | HoS | DL |
| Flowers | DominantProcessImportance | 0.9960697 | 0.865420415 | 0.53841 | 0.83614 | 0.622 | 0.85405 | 0.71663 | 0.56694 | 0.97263 | 0.97645 | 0.67647 | 0.51981 | 0.89613 | 0.972403 | 0.670141 | 0.731728 |
| Flowers | DominantProcessPvalue | 0 | 0 | 0.394 | 0 | 0.07 | 0 | 0.08 | 0.048 | 0 | 0 | 0 | 0.407 | 0 | 0 | 0.358 | 0.049 |
| Leaves | HeS | 0 | 0 | 0 | 0 | 0 | 0 | 0 | 0 | 0 | 0 | 0 | 0 | 0 | 0.001472 | 0 | 0.00831 |
| Leaves | HoS | 0.00348815 | 0.012278907 | 0.01393 | 0.02411 | 0.334665 | 0.00546 | 0.12481 | 0.39079 | 0.0402 | 0.01588 | 0.00547 | 0.69243 | 0.02009 | 0.05467 | 0.884401 | 0.073944 |
| Leaves | DL | 0.00395161 | 0.169511387 | 0.98607 | 0 | 0.525188 | 0.97871 | 0.87515 | 0.20516 | 0.12518 | 0.98412 | 0.44881 | 0 | 0.97991 | 0.943858 | 0.115599 | 0.917746 |
| Leaves | HD | 0 | 0 | 0 | 0 | 0 | 0 | 0 | 0 | 0 | 0 | 0 | 0 | 0 | 0 | 0 | 0 |
| Leaves | DR | 0.99256024 | 0.818209706 | 0 | 0.97589 | 0.140147 | 0.01584 | 3.94E-05 | 0.40405 | 0.83462 | 0 | 0.54572 | 0.30757 | 0 | 0 | 0 | 0 |
| Leaves | DominantProcess | DR | DR | DL | DR | DL | DL | DL | DR | DR | DL | DR | HoS | DL | DL | HoS | DL |
| Leaves | DominantProcessImportance | 0.99256024 | 0.818209706 | 0.98607 | 0.97589 | 0.525188 | 0.97871 | 0.87515 | 0.40405 | 0.83462 | 0.98412 | 0.54572 | 0.69243 | 0.97991 | 0.943858 | 0.884401 | 0.917746 |
| Leaves | DominantProcessPvalue | 0 | 0 | 0 | 0 | 0.235 | 0 | 0 | 0.459 | 0 | 0 | 0.376 | 0.008 | 0 | 0 | 0.131 | 0 |
| Roots | HeS | 0 | 0 | 0 | 0 | 0 | 0.02654 | 0 | 0 | 0 | 0.00606 | 0.00547 | 0 | 0.00275 | 0.000708 | 0 | 0 |
| Roots | HoS | 0.00641919 | 0.236800888 | 0.18019 | 0.00776 | 0.388638 | 0.05353 | 0.1947 | 0.48834 | 0.03876 | 0.07205 | 0.23786 | 0.01479 | 0.28515 | 0.135557 | 0.135346 | 0.090361 |
| Roots | DL | 0.06048105 | 0.537924572 | 0.81981 | 0.30197 | 0.581791 | 0.91857 | 0.8053 | 0.34707 | 0.55644 | 0.92189 | 0.67179 | 0 | 0.7121 | 0.863735 | 0.864654 | 0.909639 |
| Roots | HD | 0 | 0 | 0 | 0 | 0 | 0 | 0 | 0 | 0 | 0 | 0 | 0 | 0 | 0 | 0 | 0 |
| Roots | DR | 0.93309977 | 0.22527454 | 0 | 0.69027 | 0.029571 | 0.00135 | 0 | 0.16459 | 0.40479 | 0 | 0.08488 | 0.98521 | 0 | 0 | 0 | 0 |
| Roots | DominantProcess | DR | DL | DL | DR | DL | DL | DL | HoS | DL | DL | DL | DR | DL | DL | DL | DL |
| Roots | DominantProcessImportance | 0.93309977 | 0.537924572 | 0.81981 | 0.69027 | 0.581791 | 0.91857 | 0.8053 | 0.48834 | 0.55644 | 0.92189 | 0.67179 | 0.98521 | 0.7121 | 0.863735 | 0.864654 | 0.909639 |
| Roots | DominantProcessPvalue | 0 | 0.003 | 0 | 0.108 | 0.072 | 0 | 0 | 0.093 | 0.198 | 0 | 0 | 0 | 0.003 | 0 | 0 | 0 |

| Group | Index | bin70 | bin71 | bin72 | bin73 | bin74 | bin75 | bin76 | bin77 | bin78 | bin79 | bin80 | bin81 | bin82 | bin83 | bin84 | bin85 |
|---|---|---|---|---|---|---|---|---|---|---|---|---|---|---|---|---|---|
| Flowers | HeS | 0.01689942 | 0.029166548 | 0 | 0.01546 | 0 | 0.00175 | 0.01758 | 0.00546 | 0 | 0 | 0 | 0.0037 | 0 | 0 | 0 | 0 |
| Flowers | HoS | 0.00643287 | 0.129921295 | 0.00778 | 0.06214 | 0.457911 | 0.27996 | 0.13403 | 0.06176 | 0.009 | 0.01042 | 0.48203 | 0.64879 | 0.11701 | 0.009113 | 0.065493 | 0.296631 |
| Flowers | DL | 0.97666771 | 0.840912157 | 0.99222 | 0.9224 | 0 | 0.01741 | 0.43411 | 0.92842 | 0.991 | 0.98958 | 0 | 0.34751 | 0.84697 | 0.990887 | 0.934507 | 0.700325 |
| Flowers | HD | 0 | 0 | 0 | 0 | 0 | 0 | 0 | 0 | 0 | 0 | 0 | 0 | 0 | 0 | 0 | 0 |
| Flowers | DR | 0 | 0 | 0 | 0 | 0.542089 | 0.70088 | 0.41428 | 0.00436 | 0 | 0 | 0.51797 | 0 | 0.03602 | 0 | 0 | 0.003044 |
| Flowers | DominantProcess | DL | DL | DL | DL | DR | DR | DL | DL | DL | DL | DR | HoS | DL | DL | DL | DL |
| Flowers | DominantProcessImportance | 0.97666771 | 0.840912157 | 0.99222 | 0.9224 | 0.542089 | 0.70088 | 0.43411 | 0.92842 | 0.991 | 0.98958 | 0.51797 | 0.64879 | 0.84697 | 0.990887 | 0.934507 | 0.700325 |
| Flowers | DominantProcessPvalue | 0 | 0.021 | 0 | 0 | 0.376 | 0.013 | 0.49 | 0 | 0 | 0 | 0.407 | 0.283 | 0.001 | 0 | 0.002 | 0.009 |
| Leaves | HeS | 0 | 0.004935653 | 0 | 0.0031 | 0 | 0 | 0.0077 | 0.00433 | 0 | 0 | 0 | 0 | 0 | 0 | 0 | 0 |
| Leaves | HoS | 0.0056269 | 0.238396096 | 0.04368 | 0.00456 | 0.358486 | 0.09471 | 0.14426 | 0.0766 | 0.01602 | 0.00308 | 0.48689 | 0 | 0.05287 | 0.32532 | 0.113614 | 0.064595 |
| Leaves | DL | 0.9943731 | 0.756668251 | 0.95632 | 0.99233 | 0 | 0 | 0.79445 | 0.85532 | 0.98398 | 0.99692 | 0 | 1 | 0.85027 | 0.67468 | 0.886386 | 0.934027 |
| Leaves | HD | 0 | 0 | 0 | 0 | 0 | 0 | 0 | 0 | 0 | 0 | 0 | 0 | 0 | 0 | 0 | 0 |
| Leaves | DR | 0 | 0 | 0 | 0 | 0.641514 | 0.90529 | 0.05359 | 0.06375 | 0 | 0 | 0.51311 | 0 | 0.09686 | 0 | 0 | 0.001378 |
| Leaves | DominantProcess | DL | DL | DL | DL | DR | DR | DL | DL | DL | DL | DR | DL | DL | DL | DL | DL |
| Leaves | DominantProcessImportance | 0.9943731 | 0.756668251 | 0.95632 | 0.99233 | 0.641514 | 0.90529 | 0.79445 | 0.85532 | 0.98398 | 0.99692 | 0.51311 | 1 | 0.85027 | 0.67468 | 0.886386 | 0.934027 |
| Leaves | DominantProcessPvalue | 0 | 0.006 | 0 | 0 | 0.285 | 0 | 0 | 0 | 0 | 0 | 0.418 | 0.004 | 0 | 0.018 | 0 | 0 |
| Roots | HeS | 0.01116728 | 0.124049124 | 0.00375 | 0.01164 | 0 | 0 | 0 | 0 | 0 | 0.00024 | 0 | 0 | 0 | 0.002997 | 0 | 0.002885 |
| Roots | HoS | 0.01117749 | 0.016189975 | 0.36727 | 0.00928 | 0.857926 | 0.15448 | 0.22764 | 0.63229 | 0.46225 | 0.04726 | 0.34485 | 0.03939 | 0.32661 | 0.247505 | 0.088266 | 0.453475 |
| Roots | DL | 0.97765523 | 0.859760901 | 0.62898 | 0.97908 | 0.018039 | 0.02859 | 0.35087 | 0.36331 | 0.53775 | 0.9525 | 0 | 0.96061 | 0.66921 | 0.749497 | 0.911734 | 0.543619 |
| Roots | HD | 0 | 0 | 0 | 0 | 0 | 0 | 0 | 0 | 0 | 0 | 0 | 0 | 0 | 0 | 0 | 0 |
| Roots | DR | 0 | 0 | 0 | 0 | 0.124035 | 0.81693 | 0.42149 | 0.0044 | 0 | 0 | 0.65515 | 0 | 0.00418 | 0 | 0 | 2.12E-05 |
| Roots | DominantProcess | DL | DL | DL | DL | HoS | DR | DR | HoS | DL | DL | DR | DL | DL | DL | DL | DL |
| Roots | DominantProcessImportance | 0.97765523 | 0.859760901 | 0.62898 | 0.97908 | 0.857926 | 0.81693 | 0.42149 | 0.63229 | 0.53775 | 0.9525 | 0.65515 | 0.96061 | 0.66921 | 0.749497 | 0.911734 | 0.543619 |
| Roots | DominantProcessPvalue | 0 | 0 | 0.034 | 0 | 0 | 0 | 0.243 | 0.022 | 0.223 | 0 | 0.016 | 0 | 0.001 | 0.015 | 0 | 0.304 |

| Group | Index | bin86 | bin87 | bin88 | bin89 | bin90 | bin91 | bin92 | bin93 | bin94 | bin95 | bin96 | bin97 | bin98 | bin99 | bin100 | bin101 |
|---|---|---|---|---|---|---|---|---|---|---|---|---|---|---|---|---|---|
| Flowers | HeS | 0 | 0 | 0.00108 | 0 | 0 | 0.01736 | 0 | 0 | 0 | 0 | 0.00364 | 0 | 0 | 0 | 0 | 0 |
| Flowers | HoS | 0.01665066 | 0.009486819 | 0.18104 | 0.00169 | 0.005731 | 0.03518 | 0.27429 | 0.00367 | 0.20219 | 0.06918 | 0.03712 | 0.45737 | 0.0003 | 0.007039 | 0.02026 | 0.024829 |
| Flowers | DL | 0.98334935 | 0.985344999 | 0 | 0.95337 | 0.573836 | 0.94746 | 0.62835 | 0.99633 | 0.78417 | 0.93082 | 0.95924 | 0.54263 | 0.9997 | 0.992961 | 0.97974 | 0.007287 |
| Flowers | HD | 0 | 0 | 0 | 0 | 0 | 0 | 0 | 0 | 0 | 0 | 0 | 0 | 0 | 0 | 0 | 0 |
| Flowers | DR | 0 | 0.005168183 | 0.81788 | 0.04494 | 0.420432 | 0 | 0.09735 | 0 | 0.01364 | 0 | 0 | 0 | 0 | 0 | 0 | 0.967885 |
| Flowers | DominantProcess | DL | DL | DR | DL | DL | DL | DL | DL | DL | DL | DL | DL | DL | DL | DL | DR |
| Flowers | DominantProcessImportance | 0.98334935 | 0.985344999 | 0.81788 | 0.95337 | 0.573836 | 0.94746 | 0.62835 | 0.99633 | 0.78417 | 0.93082 | 0.95924 | 0.54263 | 0.9997 | 0.992961 | 0.97974 | 0.967885 |
| Flowers | DominantProcessPvalue | 0 | 0 | 0 | 0 | 0.309 | 0.001 | 0.2 | 0 | 0.002 | 0.003 | 0 | 0.388 | 0 | 0 | 0 | 0 |
| Leaves | HeS | 0 | 0 | 0 | 0.00818 | 0 | 0 | 0 | 0 | 0 | 0.06752 | 0 | 0 | 0 | 0 | 0 | 0 |
| Leaves | HoS | 0.00486366 | 0.007121608 | 0.3604 | 0.00458 | 0.011236 | 0.00266 | 0.14898 | 0.016 | 0.27343 | 0.00691 | 0.00223 | 0.02078 | 0.1081 | 0.033969 | 0.016733 | 0.002517 |
| Leaves | DL | 0.99513634 | 0.99130339 | 0.10794 | 0.79433 | 0.966022 | 0.99734 | 0.71027 | 0.984 | 0.72154 | 0.92558 | 0.99777 | 0.97922 | 0.8919 | 0.966031 | 0.983267 | 0.006798 |
| Leaves | HD | 0 | 0 | 0 | 0 | 0 | 0 | 0 | 0 | 0 | 0 | 0 | 0 | 0 | 0 | 0 | 0 |
| Leaves | DR | 0 | 0.001575002 | 0.53166 | 0.19291 | 0.022742 | 0 | 0.14075 | 0 | 0.00503 | 0 | 0 | 0 | 0 | 0 | 0 | 0.990685 |
| Leaves | DominantProcess | DL | DL | DR | DL | DL | DL | DL | DL | DL | DL | DL | DL | DL | DL | DL | DR |
| Leaves | DominantProcessImportance | 0.99513634 | 0.99130339 | 0.53166 | 0.79433 | 0.966022 | 0.99734 | 0.71027 | 0.984 | 0.72154 | 0.92558 | 0.99777 | 0.97922 | 0.8919 | 0.966031 | 0.983267 | 0.990685 |
| Leaves | DominantProcessPvalue | 0 | 0 | 0.125 | 0.001 | 0.015 | 0 | 0.001 | 0 | 0.001 | 0 | 0 | 0 | 0 | 0 | 0 | 0 |
| Roots | HeS | 0.0030129 | 0.013402407 | 0 | 0.02202 | 0 | 0 | 0.00521 | 0 | 0 | 0 | 0.04698 | 0 | 0 | 0 | 0 | 0 |
| Roots | HoS | 0.01409356 | 0.03737116 | 0.23528 | 0.01003 | 0.101728 | 0.00126 | 0.25083 | 0.19834 | 0.27894 | 0.11301 | 0.0303 | 0.58762 | 0.0869 | 0.142494 | 0.177206 | 0.082002 |
| Roots | DL | 0.98289354 | 0.948088848 | 0 | 0.94968 | 0.896953 | 0.99874 | 0.64345 | 0.80166 | 0.71611 | 0.88699 | 0.92273 | 0.41238 | 0.9131 | 0.857506 | 0.822794 | 0.218958 |
| Roots | HD | 0 | 0 | 0 | 0 | 0 | 0 | 0 | 0 | 0 | 0 | 0 | 0 | 0 | 0 | 0 | 0 |
| Roots | DR | 0 | 0.001137585 | 0.76472 | 0.01827 | 0.001319 | 0 | 0.10051 | 0 | 0.00495 | 0 | 0 | 0 | 0 | 0 | 0 | 0.699039 |
| Roots | DominantProcess | DL | DL | DR | DL | DL | DL | DL | DL | DL | DL | DL | HoS | DL | DL | DL | DR |

| | | | | | | | | | | | | | | | | | |
|---|---|---|---|---|---|---|---|---|---|---|---|---|---|---|---|---|---|
| Roots | DominantProcessImportance | 0.98289354 | 0.948088848 | 0.76472 | 0.94968 | 0.896953 | 0.99874 | 0.64345 | 0.80166 | 0.71611 | 0.88699 | 0.92273 | 0.58762 | 0.9131 | 0.857506 | 0.822794 | 0.699039 |
| Roots | DominantProcessPvalue | 0 | 0 | 0 | 0 | 0 | 0 | 0 | 0 | 0.002 | 0 | 0 | 0.096 | 0 | 0 | 0 | 0.007 |

| **Group** | **Index** | **bin102** | **bin103** | **bin104** |
|---|---|---|---|---|
| Flowers | HeS | 0.0020822 | 0 | 0 |
| Flowers | HoS | 0.26617296 | 0.005216587 | 0.03655 |
| Flowers | DL | 0.73174483 | 0.994783413 | 0.96345 |
| Flowers | HD | 0 | 0 | 0 |
| Flowers | DR | 0 | 0 | 0 |
| Flowers | DominantProcess | DL | DL | DL |
| Flowers | DominantProcessImportance | 0.73174483 | 0.994783413 | 0.96345 |
| Flowers | DominantProcessPvalue | 0.055 | 0 | 0 |
| Leaves | HeS | 0 | 0 | 0 |
| Leaves | HoS | 0.01483595 | 0.001035511 | 0.00573 |
| Leaves | DL | 0.98516405 | 0.998964489 | 0.99427 |
| Leaves | HD | 0 | 0 | 0 |
| Leaves | DR | 0 | 0 | 0 |
| Leaves | DominantProcess | DL | DL | DL |
| Leaves | DominantProcessImportance | 0.98516405 | 0.998964489 | 0.99427 |
| Leaves | DominantProcessPvalue | 0 | 0 | 0 |
| Roots | HeS | 0 | 0 | 0.02743 |
| Roots | HoS | 0.23551936 | 0.145075819 | 0.07208 |
| Roots | DL | 0.76448064 | 0.854924181 | 0.90049 |
| Roots | HD | 0 | 0 | 0 |
| Roots | DR | 0 | 0 | 0 |
| Roots | DominantProcess | DL | DL | DL |
| Roots | DominantProcessImportance | 0.76448064 | 0.854924181 | 0.90049 |
| Roots | DominantProcessPvalue | 0 | 0 | 0 |

**Table S11.** Indicator taxon analysis with random forest (RF) machine learning identified bacterial amplicon sequence variants (ASV) based on all the four factors (alpine Rosaceae plant, plant tissue, collection site, and exposure). Bacterial ASVs based on individual factor with significant mean decrease accuracy (MDA) from the first dataset (**A**; *Alchemilla* sp. and *G. montanum* from six collection sites) and second dataset (**B**; *Alchemilla* sp., *D. octopetala,* and *G. montanum* from two collection sites) of samples from alpine Rosaceae plants are reported. P-values for each level within the factor are shown. Taxonomy indicates kingdom (k), phylum (p), class (c), order (o), family (f), genus (g), and species (s) of selected ASVs.

## A

| RF based on tissue | | | | | RF based on alpine Rosaceae plant | | | | RF based on collection site | | | | | | | | RF based on exposure | | | |
|---|---|---|---|---|---|---|---|---|---|---|---|---|---|---|---|---|---|---|---|---|
| **ASV** | **Flowers** | **Leaves** | **Roots** | **MDA** | **ASV** | **Alchemilla** | **Geum** | **MDA** | **ASV** | **Stelvio_Park** | **Val_di_Fassa** | **Val_di_Non** | **Val_di_Pejo** | **Val_di_Sole** | **Val_Rendena** | **MDA** | **ASV** | **North** | **South** | **MDA** |
| ASV_1 | 0.0099 | 0.0099 | 0.0099 | 0.0099 | ASV_103 | 0.0099 | 0.0099 | 0.0099 | ASV_1 | 0.019802 | 0.0099 | 0.0099 | 0.14851 | 0.019802 | 0.0891 | 0.0099 | ASV_1019 | 0.0099 | 0.23762 | 0.0099 |
| ASV_10 | 0.0099 | 0.0099 | 0.0099 | 0.0099 | ASV_109 | 0.0099 | 0.0099 | 0.0099 | ASV_11 | 0.009901 | 0.0099 | 0.0297 | 0.0099 | 0.1287129 | 0.0891 | 0.0099 | ASV_107 | 0.0198 | 0.0099 | 0.0099 |
| ASV_103 | 0.0099 | 0.09901 | 0.0198 | 0.0099 | ASV_110 | 0.24752 | 0.0198 | 0.0099 | ASV_113 | 0.970297 | 0.50495 | 0.0099 | 0.34653 | 0.039604 | 0.0099 | 0.0099 | ASV_113 | 0.05941 | 0.0099 | 0.0099 |
| ASV_109 | 0.0099 | 0.0099 | 0.0099 | 0.0099 | ASV_123 | 0.0099 | 0.0099 | 0.0099 | ASV_118 | 0.2574257 | 0.0099 | 0.0099 | 0.06931 | 0.029703 | 0.396 | 0.0099 | ASV_1140 | 0.0099 | 0.0198 | 0.0099 |
| ASV_115 | 0.0099 | 0.66337 | 0.0099 | 0.0099 | ASV_145 | 0.0099 | 0.0099 | 0.0099 | ASV_12 | 0.1287129 | 0.0198 | 0.0198 | 0.15842 | 0.3960396 | 0.0891 | 0.0099 | ASV_1157 | 0.0099 | 0.0099 | 0.0099 |
| ASV_116 | 0.0099 | 0.0099 | 0.0099 | 0.0099 | ASV_1511 | 0.0495 | 0.0099 | 0.0099 | ASV_122 | 0.009901 | 0.0099 | 0.0099 | 0.0099 | 0.019802 | 0.0099 | 0.0099 | ASV_148 | 0.05941 | 0.0099 | 0.0099 |
| ASV_119 | 0.0297 | 0.11881 | 0.0198 | 0.0099 | ASV_154 | 0.0099 | 0.0099 | 0.0099 | ASV_124 | 0.0594059 | 0.0099 | 0.0099 | 0.51485 | 0.009901 | 0.0099 | 0.0099 | ASV_16 | 0.0198 | 0.11881 | 0.0099 |
| ASV_120 | 0.0099 | 0.0297 | 0.16832 | 0.0099 | ASV_158 | 0.0198 | 0.0198 | 0.0099 | ASV_136 | 0.009901 | 0.84158 | 0.9901 | 0.0495 | 0.009901 | 0.0099 | 0.0099 | ASV_160 | 0.0495 | 0.09901 | 0.0099 |
| ASV_126 | 0.0099 | 0.85149 | 0.62376 | 0.0099 | ASV_176 | 0.0099 | 0.0099 | 0.0099 | ASV_138 | 0.019802 | 0.25743 | 0.0099 | 0.0099 | 0.009901 | 0.0099 | 0.0099 | ASV_225 | 0.35644 | 0.0198 | 0.0099 |
| ASV_137 | 0.0297 | 0.07921 | 0.09901 | 0.0099 | ASV_177 | 0.0099 | 0.0099 | 0.0099 | ASV_14 | 0.039604 | 0.0396 | 0.0099 | 0.32673 | 0.049505 | 0.2277 | 0.0099 | ASV_34 | 0.0099 | 0.0198 | 0.0099 |
| ASV_14 | 0.0099 | 0.0099 | 0.0099 | 0.0099 | ASV_200 | 0.0099 | 0.0099 | 0.0099 | ASV_141 | 0.1584158 | 0.35644 | 0.0198 | 0.77228 | 0.049505 | 0.0099 | 0.0099 | ASV_42 | 0.0495 | 0.0198 | 0.0099 |
| ASV_1635 | 0.0099 | 0.0396 | 0.0099 | 0.0099 | ASV_206 | 0.0099 | 0.0099 | 0.0099 | ASV_143 | 0.009901 | 0.0099 | 0.43564 | 0.0099 | 0.009901 | 0.0099 | 0.0099 | ASV_682 | 0.0099 | 0.0099 | 0.0099 |
| ASV_167 | 0.0099 | 0.28713 | 0.0099 | 0.0099 | ASV_212 | 0.0099 | 0.0099 | 0.0099 | ASV_150 | 0.4455446 | 0.0099 | 0.07921 | 0.07921 | 0.1089109 | 0.1683 | 0.0099 | ASV_733 | 0.0099 | 0.65347 | 0.0099 |
| ASV_17 | 0.63366 | 0.0099 | 0.28713 | 0.0099 | ASV_232 | 0.0099 | 0.12871 | 0.0099 | ASV_157 | 0.009901 | 0.9703 | 0.0099 | 0.0099 | 0.009901 | 0.0099 | 0.0099 | ASV_755 | 0.0099 | 0.0099 | 0.0099 |
| ASV_170 | 0.0099 | 0.05941 | 0.0495 | 0.0099 | ASV_235 | 0.0099 | 0.30693 | 0.0099 | ASV_17 | 0.009901 | 0.12871 | 0.30693 | 0.0099 | 0.2673267 | 0.0099 | 0.0099 | ASV_776 | 0.0198 | 0.0099 | 0.0099 |
| ASV_174 | 0.0099 | 0.0198 | 0.0099 | 0.0099 | ASV_244 | 0.0099 | 0.0099 | 0.0099 | ASV_172 | 0.019802 | 0.65347 | 0.0198 | 0.0099 | 0.6039604 | 0.2475 | 0.0099 | ASV_940 | 0.0198 | 0.0099 | 0.0099 |
| ASV_18 | 0.0099 | 0.0099 | 0.0099 | 0.0099 | ASV_251 | 0.0099 | 0.12871 | 0.0099 | ASV_173 | 0.2970297 | 0.06931 | 0.13861 | 0.32673 | 0.009901 | 0.2871 | 0.0099 | ASV_1193 | 0.0198 | 0.0297 | 0.0198 |
| ASV_182 | 0.0099 | 0.0099 | 0.0297 | 0.0099 | ASV_255 | 0.0099 | 0.0099 | 0.0099 | ASV_183 | 0.019802 | 0.42574 | 0.0396 | 0.0099 | 0.039604 | 0.0099 | 0.0099 | ASV_141 | 0.0495 | 0.23762 | 0.0198 |
| ASV_184 | 0.0099 | 0.0099 | 0.0099 | 0.0099 | ASV_264 | 0.06931 | 0.0099 | 0.0099 | ASV_186 | 0.009901 | 0.0099 | 0.0198 | 0.0099 | 0.009901 | 0.0099 | 0.0099 | ASV_414 | 0.0099 | 0.42574 | 0.0198 |
| ASV_189 | 0.0099 | 0.0495 | 0.0198 | 0.0099 | ASV_270 | 0.0099 | 0.0099 | 0.0099 | ASV_188 | 0.049505 | 0.0099 | 0.0099 | 1 | 0.009901 | 0.0099 | 0.0099 | ASV_62 | 0.9802 | 0.0099 | 0.0198 |
| ASV_199 | 0.0099 | 0.0099 | 0.0099 | 0.0099 | ASV_272 | 0.0099 | 0.0099 | 0.0099 | ASV_19 | 0.0792079 | 0.06931 | 0.06931 | 0.11881 | 0.029703 | 0.0099 | 0.0099 | ASV_120 | 0.15842 | 0.0396 | 0.0297 |
| ASV_200 | 0.0198 | 0.33663 | 0.0099 | 0.0099 | ASV_275 | 0.0396 | 0.0198 | 0.0099 | ASV_196 | 0.2277228 | 0.12871 | 0.0495 | 0.25743 | 0.009901 | 0.8119 | 0.0099 | ASV_157 | 0.0099 | 0.05941 | 0.0297 |
| ASV_206 | 0.0099 | 0.25743 | 0.16832 | 0.0099 | ASV_280 | 0.0099 | 0.0099 | 0.0099 | ASV_2 | 0.019802 | 0.0099 | 0.0099 | 0.0198 | 0.009901 | 0.0099 | 0.0099 | ASV_212 | 0.0495 | 0.08911 | 0.0297 |
| ASV_208 | 0.0099 | 0.0198 | 0.0099 | 0.0099 | ASV_287 | 0.0099 | 0.0495 | 0.0099 | ASV_20 | 0.019802 | 0.0099 | 0.0099 | 0.0297 | 0.5742574 | 0.0396 | 0.0099 | ASV_287 | 0.90099 | 0.0099 | 0.0297 |
| ASV_21 | 0.0297 | 0.35644 | 0.16832 | 0.0099 | ASV_290 | 0.0198 | 0.0099 | 0.0099 | ASV_205 | 0.0594059 | 0.9901 | 0.0297 | 0.19802 | 0.0990099 | 0.0099 | 0.0099 | ASV_30 | 0.0495 | 0.0198 | 0.0297 |
| ASV_210 | 0.0099 | 0.0099 | 0.0099 | 0.0099 | ASV_297 | 0.0099 | 0.0099 | 0.0099 | ASV_209 | 0.9306931 | 0.82178 | 0.0099 | 0.11881 | 0.029703 | 0.0099 | 0.0099 | ASV_366 | 0.0198 | 0.22772 | 0.0297 |
| ASV_211 | 0.0099 | 0.0495 | 0.0099 | 0.0099 | ASV_32 | 0.0099 | 0.0099 | 0.0099 | ASV_21 | 0.4059406 | 0.12871 | 0.0297 | 0.05941 | 0.4653465 | 0.0198 | 0.0099 | ASV_46 | 0.75248 | 0.0099 | 0.0297 |
| ASV_218 | 0.0198 | 0.0099 | 0.0099 | 0.0099 | ASV_339 | 0.0099 | 0.0099 | 0.0099 | ASV_22 | 0.029703 | 0.0297 | 0.0495 | 0.36634 | 0.1188119 | 0.0099 | 0.0099 | ASV_488 | 0.12871 | 0.0495 | 0.0297 |
| ASV_226 | 0.0099 | 0.0198 | 0.0099 | 0.0099 | ASV_35 | 0.17822 | 0.0099 | 0.0099 | ASV_226 | 0.1782178 | 0.67327 | 0.0099 | 0.0297 | 0.0792079 | 0.703 | 0.0099 | ASV_573 | 0.76238 | 0.0099 | 0.0297 |
| ASV_229 | 0.0099 | 0.0099 | 0.0099 | 0.0099 | ASV_359 | 0.0099 | 0.0099 | 0.0099 | ASV_242 | 0.019802 | 0.0099 | 0.0198 | 0.0099 | 1 | 0.0099 | 0.0099 | ASV_58 | 0.13861 | 0.0297 | 0.0297 |
| ASV_234 | 0.0099 | 0.0495 | 0.53465 | 0.0099 | ASV_363 | 0.0099 | 0.0099 | 0.0099 | ASV_244 | 0.039604 | 0.0099 | 0.13861 | 0.0099 | 0.019802 | 0.0198 | 0.0099 | ASV_88 | 0.0297 | 0.0396 | 0.0297 |
| ASV_238 | 0.0099 | 0.0099 | 0.0099 | 0.0099 | ASV_366 | 0.13861 | 0.0099 | 0.0099 | ASV_250 | 0.019802 | 0.0297 | 0.15842 | 0.07921 | 0.019802 | 0.2871 | 0.0099 | ASV_1233 | 0.25743 | 0.0297 | 0.0396 |
| ASV_250 | 0.0099 | 0.0099 | 0.0198 | 0.0099 | ASV_385 | 0.0099 | 0.0099 | 0.0099 | ASV_263 | 0.049505 | 0.0099 | 0.0396 | 0.06931 | 0.009901 | 0.0099 | 0.0099 | ASV_177 | 0.29703 | 0.0297 | 0.0396 |
| ASV_265 | 0.0099 | 0.49505 | 0.0396 | 0.0099 | ASV_39 | 0.0099 | 0.0297 | 0.0099 | ASV_267 | 0.7029703 | 0.77228 | 0.0198 | 0.9703 | 0.019802 | 0.0297 | 0.0099 | ASV_284 | 0.0198 | 0.07921 | 0.0396 |

| | | | | | | | | | | | | | | | | | | | | |
|---|---|---|---|---|---|---|---|---|---|---|---|---|---|---|---|---|---|---|---|---|
| ASV_27 | 0.0099 | 0.0099 | 0.0099 | 0.0099 | ASV_421 | 0.0099 | 0.07921 | 0.0099 | ASV_272 | 0.009901 | 0.32673 | 0.11881 | 0.94059 | 0.039604 | 0.0198 | 0.0099 | ASV_439 | 0.88119 | 0.0198 | 0.0396 |
| ASV_274 | 0.0099 | 0.14851 | 0.0297 | 0.0099 | ASV_445 | 0.0099 | 0.07921 | 0.0099 | ASV_29 | 0.0990099 | 0.0099 | 0.36634 | 0.07921 | 0.1287129 | 0.297 | 0.0099 | ASV_455 | 0.0495 | 0.10891 | 0.0396 |
| ASV_275 | 0.0099 | 0.17822 | 0.0099 | 0.0099 | ASV_451 | 0.0198 | 0.0099 | 0.0099 | ASV_295 | 0.049505 | 0.0099 | 0.33663 | 0.46535 | 0.019802 | 0.8812 | 0.0099 | ASV_582 | 0.0297 | 0.15842 | 0.0396 |
| ASV_28 | 0.07921 | 0.0099 | 0.0099 | 0.0099 | ASV_495 | 0.0099 | 0.0099 | 0.0099 | ASV_297 | 0.009901 | 0.0099 | 0.0099 | 0.0099 | 0.009901 | 0.0396 | 0.0099 | ASV_712 | 0.20792 | 0.0198 | 0.0396 |
| ASV_284 | 0.0396 | 0.0297 | 0.0495 | 0.0099 | ASV_504 | 0.0099 | 0.0396 | 0.0099 | ASV_298 | 0.0990099 | 0.18812 | 0.0198 | 0.0099 | 0.019802 | 0.3267 | 0.0099 | ASV_140 | 0.0495 | 0.34653 | 0.0495 |
| ASV_290 | 0.0099 | 0.0297 | 0.0099 | 0.0099 | ASV_51 | 0.0297 | 0.0099 | 0.0099 | ASV_3 | 0.009901 | 0.05941 | 0.0297 | 0.60396 | 0.009901 | 0.1485 | 0.0099 | ASV_209 | 0.67327 | 0.0495 | 0.0495 |
| ASV_293 | 0.0099 | 0.0297 | 0.0198 | 0.0099 | ASV_537 | 0.0099 | 0.0297 | 0.0099 | ASV_311 | 0.039604 | 0.09901 | 0.0297 | 0.0198 | 0.0990099 | 0.0099 | 0.0099 | ASV_247 | 0.21782 | 0.0297 | 0.0495 |
| ASV_299 | 0.0099 | 0.10891 | 0.0396 | 0.0099 | ASV_56 | 0.0099 | 0.0099 | 0.0099 | ASV_347 | 0.2376238 | 0.0198 | 0.67327 | 0.28713 | 0.2970297 | 0.0099 | 0.0099 | ASV_254 | 0.0297 | 0.27723 | 0.0495 |
| ASV_3 | 0.0099 | 0.0099 | 0.0099 | 0.0099 | ASV_62 | 0.0297 | 0.0099 | 0.0099 | ASV_348 | 0.009901 | 0.90099 | 0.0198 | 0.0099 | 0.009901 | 0.9505 | 0.0099 | ASV_26 | 0.10891 | 0.0297 | 0.0495 |
| ASV_305 | 0.0099 | 0.05941 | 0.0198 | 0.0099 | ASV_64 | 0.0099 | 0.0099 | 0.0099 | ASV_35 | 0.4356436 | 0.0396 | 0.0495 | 0.40594 | 0.009901 | 0.3267 | 0.0099 | ASV_286 | 0.28713 | 0.0297 | 0.0495 |
| ASV_309 | 0.0099 | 0.51485 | 0.45545 | 0.0099 | ASV_665 | 0.29703 | 0.0099 | 0.0099 | ASV_36 | 0.2673267 | 0.0099 | 0.24752 | 0.0198 | 0.009901 | 0.0891 | 0.0099 | ASV_47 | 0.05941 | 0.13861 | 0.0495 |
| ASV_313 | 0.0099 | 0.0198 | 0.0099 | 0.0099 | ASV_689 | 0.0198 | 0.0495 | 0.0099 | ASV_385 | 0.009901 | 0.0099 | 0.0099 | 0.0099 | 0.009901 | 0.0099 | 0.0099 | ASV_61 | 0.22772 | 0.05941 | 0.0495 |
| ASV_315 | 0.0099 | 0.0396 | 0.22772 | 0.0099 | ASV_693 | 0.0198 | 0.0099 | 0.0099 | ASV_39 | 0.009901 | 0.24752 | 0.07921 | 0.19802 | 0.1485149 | 0.2376 | 0.0099 | ASV_79 | 0.0495 | 0.18812 | 0.0495 |
| ASV_316 | 0.0099 | 0.0099 | 0.0099 | 0.0099 | ASV_78 | 0.0099 | 0.0099 | 0.0099 | ASV_394 | 0.009901 | 0.20792 | 0.0099 | 0.0099 | 0.1089109 | 0.0693 | 0.0099 | | | | |
| ASV_34 | 0.42574 | 0.0099 | 0.0099 | 0.0099 | ASV_87 | 0.0099 | 0.0099 | 0.0099 | ASV_4 | 0.009901 | 0.0099 | 0.0099 | 0.14851 | 0.009901 | 0.099 | 0.0099 | | | | |
| ASV_352 | 0.0099 | 0.0198 | 0.0099 | 0.0099 | ASV_98 | 0.0099 | 0.0099 | 0.0099 | ASV_42 | 0.970297 | 0.0099 | 0.11881 | 0.0297 | 0.049505 | 0.0396 | 0.0099 | | | | |
| ASV_359 | 0.0099 | 0.24752 | 0.0099 | 0.0099 | ASV_1134 | 0.0099 | 0.0495 | 0.0198 | ASV_423 | 0.009901 | 0.0099 | 0.0297 | 0.0099 | 0.009901 | 0.0099 | 0.0099 | | | | |
| ASV_361 | 0.0099 | 0.0198 | 0.0198 | 0.0099 | ASV_115 | 0.10891 | 0.08911 | 0.0198 | ASV_44 | 0.019802 | 0.0099 | 0.28713 | 0.57426 | 0.1089109 | 0.0396 | 0.0099 | | | | |
| ASV_363 | 0.0099 | 0.30693 | 0.0099 | 0.0099 | ASV_116 | 0.0198 | 0.05941 | 0.0198 | ASV_449 | 0.009901 | 0.12871 | 0.0198 | 0.94059 | 0.9306931 | 0.2178 | 0.0099 | | | | |
| ASV_367 | 0.0099 | 0.0099 | 0.0099 | 0.0099 | ASV_120 | 0.06931 | 0.0099 | 0.0198 | ASV_49 | 0.009901 | 0.0099 | 0.0198 | 0.08911 | 0.0594059 | 0.0198 | 0.0099 | | | | |
| ASV_37 | 0.41584 | 0.0099 | 0.44554 | 0.0099 | ASV_174 | 0.0099 | 0.0396 | 0.0198 | ASV_523 | 0.009901 | 0.0099 | 0.64356 | 0.0099 | 0.009901 | 0.0099 | 0.0099 | | | | |
| ASV_370 | 0.0198 | 0.51485 | 0.0198 | 0.0099 | ASV_178 | 0.06931 | 0.0099 | 0.0198 | ASV_536 | 0.9108911 | 0.0297 | 0.0297 | 0.08911 | 0.1089109 | 0.0099 | 0.0099 | | | | |
| ASV_371 | 0.0099 | 0.0099 | 0.0099 | 0.0099 | ASV_220 | 0.20792 | 0.0099 | 0.0198 | ASV_55 | 0.8217822 | 0.27723 | 0.0099 | 0.67327 | 0.0693069 | 0.0099 | 0.0099 | | | | |
| ASV_38 | 0.0099 | 0.0099 | 0.0099 | 0.0099 | ASV_265 | 0.08911 | 0.0396 | 0.0198 | ASV_58 | 0.009901 | 0.0099 | 0.0099 | 0.0297 | 0.009901 | 0.0099 | 0.0099 | | | | |
| ASV_4 | 0.0099 | 0.0099 | 0.0099 | 0.0099 | ASV_278 | 0.19802 | 0.0099 | 0.0198 | ASV_59 | 0.029703 | 0.0099 | 0.0396 | 0.06931 | 0.1485149 | 0.4752 | 0.0099 | | | | |
| ASV_419 | 0.0099 | 0.0099 | 0.0099 | 0.0099 | ASV_284 | 0.33663 | 0.0099 | 0.0198 | ASV_6 | 0.0693069 | 0.36634 | 0.0099 | 0.0297 | 0.029703 | 0.0099 | 0.0099 | | | | |
| ASV_424 | 0.0099 | 0.10891 | 0.0495 | 0.0099 | ASV_335 | 0.27723 | 0.0099 | 0.0198 | ASV_60 | 0.1782178 | 0.9604 | 0.0396 | 0.0198 | 0.009901 | 0.0099 | 0.0099 | | | | |
| ASV_439 | 0.0099 | 0.20792 | 0.22772 | 0.0099 | ASV_336 | 0.17822 | 0.0198 | 0.0198 | ASV_648 | 0.2673267 | 0.0099 | 0.07921 | 0.0297 | 0.0891089 | 0.0198 | 0.0099 | | | | |
| ASV_472 | 0.0099 | 0.36634 | 0.53465 | 0.0099 | ASV_37 | 0.0198 | 0.0396 | 0.0198 | ASV_71 | 0.009901 | 0.79208 | 0.0099 | 0.0396 | 0.009901 | 0.099 | 0.0099 | | | | |
| ASV_478 | 0.0099 | 0.12871 | 0.0099 | 0.0099 | ASV_370 | 0.06931 | 0.0099 | 0.0198 | ASV_73 | 0.1584158 | 0.29703 | 0.0099 | 0.0297 | 0.0792079 | 0.2871 | 0.0099 | | | | |
| ASV_491 | 0.0099 | 0.88119 | 0.42574 | 0.0099 | ASV_375 | 0.0099 | 0.28713 | 0.0198 | ASV_74 | 0.7227723 | 0.0099 | 0.0297 | 0.92079 | 0.1089109 | 0.3267 | 0.0099 | | | | |
| ASV_560 | 0.0297 | 0.0198 | 0.0198 | 0.0099 | ASV_403 | 0.0198 | 0.71287 | 0.0198 | ASV_755 | 0.009901 | 0.0099 | 0.93069 | 0.0396 | 0.2574257 | 0.4059 | 0.0099 | | | | |
| ASV_585 | 0.0099 | 0.0396 | 0.0396 | 0.0099 | ASV_407 | 0.0099 | 0.06931 | 0.0198 | ASV_76 | 0.009901 | 0.05941 | 0.0099 | 0.70297 | 0.0792079 | 0.8317 | 0.0099 | | | | |
| ASV_609 | 0.0099 | 0.0099 | 0.0198 | 0.0099 | ASV_440 | 0.0396 | 0.0099 | 0.0198 | ASV_79 | 0.2772277 | 0.0495 | 0.59406 | 0.22772 | 0.019802 | 0.0099 | 0.0099 | | | | |
| ASV_616 | 0.0099 | 0.0198 | 0.0396 | 0.0099 | ASV_472 | 0.0495 | 0.0198 | 0.0198 | ASV_8 | 0.7425743 | 0.36634 | 0.07921 | 0.45545 | 0.0792079 | 0.0099 | 0.0099 | | | | |
| ASV_64 | 0.0198 | 0.06931 | 0.0099 | 0.0099 | ASV_474 | 0.0099 | 0.44554 | 0.0198 | ASV_81 | 0.009901 | 0.58416 | 0.0495 | 0.0099 | 0.2574257 | 0.6139 | 0.0099 | | | | |
| ASV_66 | 0.0099 | 0.0099 | 0.0099 | 0.0099 | ASV_491 | 0.0198 | 0.0297 | 0.0198 | ASV_85 | 0.019802 | 0.0198 | 0.0099 | 0.0099 | 0.029703 | 0.0198 | 0.0099 | | | | |
| ASV_69 | 0.0099 | 0.0099 | 0.0099 | 0.0099 | ASV_492 | 0.10891 | 0.0198 | 0.0198 | ASV_90 | 0.009901 | 0.0099 | 0.0099 | 0.74257 | 0.019802 | 0.0297 | 0.0099 | | | | |
| ASV_733 | 0.0297 | 0.05941 | 0.09901 | 0.0099 | ASV_544 | 0.50495 | 0.0198 | 0.0198 | ASV_957 | 0.2871287 | 0.12871 | 0.0099 | 0.0495 | 0.0990099 | 0.0693 | 0.0099 | | | | |
| ASV_747 | 0.0099 | 0.0099 | 0.0198 | 0.0099 | ASV_575 | 0.0396 | 0.0297 | 0.0198 | ASV_97 | 0.039604 | 0.07921 | 0.0099 | 0.0099 | 0.7722772 | 0.0099 | 0.0099 | | | | |
| ASV_75 | 0.0099 | 0.0099 | 0.13861 | 0.0099 | ASV_662 | 0.0099 | 0.62376 | 0.0198 | ASV_98 | 0.009901 | 0.0099 | 0.54455 | 0.0198 | 0.960396 | 0.8614 | 0.0099 | | | | |
| ASV_77 | 0.0099 | 0.0198 | 0.0099 | 0.0099 | ASV_83 | 0.32673 | 0.0099 | 0.0198 | ASV_99 | 0.0693069 | 0.0396 | 0.0099 | 0.0495 | 0.009901 | 0.0693 | 0.0099 | | | | |
| ASV_82 | 0.0099 | 0.0099 | 0.0099 | 0.0099 | ASV_876 | 0.0198 | 0.20792 | 0.0198 | ASV_10 | 0.049505 | 0.48515 | 0.0099 | 0.57426 | 0.2376238 | 0.4158 | 0.0198 | | | | |
| ASV_84 | 0.0099 | 0.0099 | 0.0099 | 0.0099 | ASV_274 | 0.40594 | 0.0396 | 0.0297 | ASV_106 | 0.2970297 | 0.71287 | 0.15842 | 0.28713 | 0.0990099 | 0.0198 | 0.0198 | | | | |
| ASV_87 | 0.0198 | 0.13861 | 0.0297 | 0.0099 | ASV_285 | 0.0396 | 0.05941 | 0.0297 | ASV_1157 | 0.019802 | 0.87129 | 0.24752 | 0.05941 | 0.7920792 | 0.0099 | 0.0198 | | | | |
| ASV_876 | 0.19802 | 0.0099 | 0.08911 | 0.0099 | ASV_295 | 0.31683 | 0.0198 | 0.0297 | ASV_16 | 0.5742574 | 0.0297 | 0.07921 | 0.0396 | 0.7227723 | 0.1287 | 0.0198 | | | | |

| | | | | | | | | | | | | | | | | |
|---|---|---|---|---|---|---|---|---|---|---|---|---|---|---|---|---|
| ASV_88 | 0.0099 | 0.07921 | 0.0198 | 0.0099 | ASV_299 | 0.17822 | 0.0396 | 0.0297 | ASV_177 | 0.039604 | 0.0099 | 0.08911 | 0.0099 | 0.009901 | 0.0198 | 0.0198 |
| ASV_144 | 0.0198 | 0.50495 | 0.14851 | 0.0198 | ASV_455 | 0.0495 | 0.0198 | 0.0297 | ASV_18 | 0.2277228 | 0.0297 | 0.0198 | 0.77228 | 0.3762376 | 0.2079 | 0.0198 |
| ASV_154 | 0.0198 | 0.0198 | 0.0198 | 0.0198 | ASV_488 | 0.0099 | 0.24752 | 0.0297 | ASV_189 | 0.039604 | 0.23762 | 0.0099 | 0.0099 | 0.1782178 | 0.3069 | 0.0198 |
| ASV_235 | 0.08911 | 0.0099 | 0.56436 | 0.0198 | ASV_634 | 0.22772 | 0.0099 | 0.0297 | ASV_23 | 0.960396 | 0.0099 | 0.51485 | 0.55446 | 0.009901 | 0.604 | 0.0198 |
| ASV_301 | 0.0297 | 0.10891 | 0.0297 | 0.0198 | ASV_91 | 0.07921 | 0.0297 | 0.0297 | ASV_239 | 0.1188119 | 0.0297 | 0.0198 | 0.0099 | 0.019802 | 0.0297 | 0.0198 |
| ASV_308 | 0.43564 | 0.10891 | 0.0099 | 0.0198 | ASV_93 | 0.25743 | 0.0297 | 0.0297 | ASV_252 | 0.3168317 | 0.0396 | 0.68317 | 0.0495 | 0.029703 | 0.0198 | 0.0198 |
| ASV_314 | 0.0099 | 0.45545 | 0.11881 | 0.0198 | ASV_95 | 0.0495 | 0.0198 | 0.0297 | ASV_262 | 0.6336634 | 0.32673 | 0.0396 | 0.60396 | 0.3465347 | 0.0297 | 0.0198 |
| ASV_327 | 0.0099 | 0.53465 | 0.12871 | 0.0198 | ASV_134 | 0.25743 | 0.0297 | 0.0396 | ASV_278 | 0.019802 | 0.0099 | 0.78218 | 0.49505 | 0.5346535 | 0.4059 | 0.0198 |
| ASV_347 | 0.23762 | 0.0297 | 0.19802 | 0.0198 | ASV_194 | 0.10891 | 0.0495 | 0.0396 | ASV_28 | 0.1881188 | 0.17822 | 0.9703 | 0.0396 | 0.029703 | 0.4554 | 0.0198 |
| ASV_387 | 0.06931 | 0.15842 | 0.05941 | 0.0198 | ASV_198 | 0.20792 | 0.09901 | 0.0396 | ASV_30 | 0.5247525 | 0.0495 | 0.78218 | 0.0099 | 0.0990099 | 0.3465 | 0.0198 |
| ASV_41 | 0.0297 | 0.20792 | 0.0495 | 0.0198 | ASV_2 | 0.22772 | 0.06931 | 0.0396 | ASV_31 | 0.4257426 | 0.37624 | 0.18812 | 0.16832 | 0.039604 | 0.0099 | 0.0198 |
| ASV_413 | 0.0099 | 0.52475 | 0.32673 | 0.0198 | ASV_208 | 0.0396 | 0.42574 | 0.0396 | ASV_315 | 0.029703 | 0.0198 | 0.0099 | 1 | 0.6039604 | 0.0297 | 0.0198 |
| ASV_440 | 0.0396 | 0.32673 | 0.11881 | 0.0198 | ASV_252 | 0.08911 | 0.09901 | 0.0396 | ASV_320 | 0.2178218 | 0.10891 | 0.58416 | 0.0297 | 0.029703 | 0.495 | 0.0198 |
| ASV_447 | 0.0099 | 0.05941 | 0.29703 | 0.0198 | ASV_404 | 0.0396 | 0.09901 | 0.0396 | ASV_324 | 0.0891089 | 0.0099 | 0.0396 | 0.65347 | 0.5247525 | 0.0495 | 0.0198 |
| ASV_504 | 0.0099 | 0.07921 | 0.0099 | 0.0198 | ASV_506 | 0.11881 | 0.07921 | 0.0396 | ASV_325 | 0.019802 | 0.0099 | 0.06931 | 0.07921 | 0.6930693 | 0.1782 | 0.0198 |
| ASV_518 | 0.0099 | 0.43564 | 0.30693 | 0.0198 | ASV_600 | 0.0495 | 0.25743 | 0.0396 | ASV_34 | 0.0693069 | 0.38614 | 0.13861 | 0.0396 | 0.0792079 | 0.1782 | 0.0198 |
| ASV_607 | 0.0099 | 0.07921 | 0.06931 | 0.0198 | ASV_733 | 0.10891 | 0.0396 | 0.0396 | ASV_361 | 0.039604 | 0.70297 | 0.0396 | 0.0099 | 0.049505 | 0.0594 | 0.0198 |
| ASV_629 | 0.05941 | 0.25743 | 0.0099 | 0.0198 | ASV_736 | 0.18812 | 0.0198 | 0.0396 | ASV_413 | 0.009901 | 0.53465 | 0.05941 | 0.71287 | 0.2772277 | 0.2871 | 0.0198 |
| ASV_640 | 0.0495 | 0.10891 | 0.16832 | 0.0198 | ASV_88 | 0.21782 | 0.0495 | 0.0396 | ASV_435 | 0.029703 | 0.74257 | 0.57426 | 0.09901 | 0.0990099 | 0.1188 | 0.0198 |
| ASV_65 | 0.06931 | 0.27723 | 0.11881 | 0.0198 | ASV_920 | 0.0297 | 0.36634 | 0.0396 | ASV_442 | 0.009901 | 0.23762 | 0.16832 | 0.07921 | 0.0792079 | 0.3663 | 0.0198 |
| ASV_686 | 0.0198 | 0.0198 | 0.23762 | 0.0198 | ASV_118 | 0.36634 | 0.0198 | 0.0495 | ASV_477 | 0.0594059 | 0.0099 | 0.0297 | 1 | 0.5643564 | 0.6436 | 0.0198 |
| ASV_696 | 0.0198 | 0.27723 | 0.0099 | 0.0198 | ASV_24 | 0.09901 | 0.09901 | 0.0495 | ASV_487 | 0.0594059 | 0.29703 | 0.08911 | 0.10891 | 0.3861386 | 0.0396 | 0.0198 |
| ASV_699 | 0.0099 | 0.42574 | 0.11881 | 0.0198 | ASV_242 | 0.37624 | 0.08911 | 0.0495 | ASV_5 | 0.039604 | 0.0396 | 0.11881 | 0.05941 | 0.960396 | 0.0198 | 0.0198 |
| ASV_71 | 0.29703 | 0.0297 | 0.11881 | 0.0198 | ASV_257 | 0.06931 | 0.0198 | 0.0495 | ASV_508 | 0.2574257 | 0.26733 | 0.14851 | 0.0396 | 0.0693069 | 0.0792 | 0.0198 |
| ASV_8 | 0.0396 | 0.0396 | 0.42574 | 0.0198 | ASV_36 | 0.22772 | 0.09901 | 0.0495 | ASV_549 | 0.9207921 | 0.88119 | 0.0495 | 0.05941 | 0.039604 | 0.0099 | 0.0198 |
| ASV_820 | 0.0099 | 0.9505 | 0.40594 | 0.0198 | ASV_560 | 0.0396 | 0.34653 | 0.0495 | ASV_554 | 0.2871287 | 0.09901 | 0.0495 | 0.0198 | 0.0792079 | 0.099 | 0.0198 |
| ASV_98 | 0.06931 | 0.0297 | 0.0198 | 0.0198 | ASV_59 | 0.09901 | 0.08911 | 0.0495 | ASV_609 | 0.7425743 | 0.65347 | 0.57426 | 0.0198 | 0.5148515 | 0.0099 | 0.0198 |
| ASV_107 | 0.22772 | 0.36634 | 0.0099 | 0.0297 | ASV_639 | 0.21782 | 0.0297 | 0.0495 | ASV_61 | 0.0693069 | 0.0198 | 0.0297 | 0.77228 | 0.009901 | 0.8218 | 0.0198 |
| ASV_130 | 0.0198 | 0.20792 | 0.47525 | 0.0297 | | | | | ASV_63 | 0.5148515 | 0.0099 | 0.55446 | 0.05941 | 0.2772277 | 0.3762 | 0.0198 |
| ASV_160 | 0.07921 | 0.47525 | 0.0198 | 0.0297 | | | | | ASV_641 | 0.039604 | 0.46535 | 0.30693 | 0.0297 | 0.0792079 | 0.0396 | 0.0198 |
| ASV_19 | 0.0495 | 0.17822 | 0.37624 | 0.0297 | | | | | ASV_65 | 0.009901 | 0.0099 | 0.90099 | 0.0099 | 0.019802 | 0.1188 | 0.0198 |
| ASV_255 | 0.0198 | 0.16832 | 0.0099 | 0.0297 | | | | | ASV_879 | 0.049505 | 0.41584 | 0.32673 | 0.36634 | 0.0792079 | 0.0297 | 0.0198 |
| ASV_266 | 0.0099 | 0.49505 | 0.48515 | 0.0297 | | | | | ASV_91 | 0.4554455 | 0.0198 | 0.0297 | 0.14851 | 0.5346535 | 0.1485 | 0.0198 |
| ASV_285 | 0.0198 | 0.33663 | 0.27723 | 0.0297 | | | | | ASV_96 | 0.2673267 | 0.0198 | 0.24752 | 0.12871 | 0.0891089 | 0.1683 | 0.0198 |
| ASV_375 | 0.86139 | 0.0198 | 0.70297 | 0.0297 | | | | | ASV_103 | 0.1485149 | 0.34653 | 0.44554 | 0.50495 | 0.049505 | 0.0198 | 0.0297 |
| ASV_394 | 0.0495 | 0.0198 | 0.17822 | 0.0297 | | | | | ASV_104 | 0.0693069 | 0.21782 | 0.0396 | 0.20792 | 0.9108911 | 0.099 | 0.0297 |
| ASV_398 | 0.0495 | 0.41584 | 0.0198 | 0.0297 | | | | | ASV_111 | 0.0891089 | 0.0198 | 0.27723 | 0.0396 | 0.049505 | 0.3861 | 0.0297 |
| ASV_399 | 0.0297 | 0.33663 | 0.41584 | 0.0297 | | | | | ASV_120 | 0.2277228 | 0.0297 | 0.0396 | 0.17822 | 0.0891089 | 0.4059 | 0.0297 |
| ASV_451 | 0.0198 | 0.36634 | 0.0198 | 0.0297 | | | | | ASV_135 | 0.5643564 | 0.05941 | 0.05941 | 0.51485 | 0.0594059 | 0.1485 | 0.0297 |
| ASV_47 | 0.32673 | 0.48515 | 0.0099 | 0.0297 | | | | | ASV_1376 | 0.1782178 | 0.68317 | 0.15842 | 0.24752 | 0.4356436 | 0.0891 | 0.0297 |
| ASV_514 | 0.10891 | 0.41584 | 0.08911 | 0.0297 | | | | | ASV_140 | 0.049505 | 0.0198 | 0.09901 | 0.09901 | 0.0891089 | 0.8119 | 0.0297 |
| ASV_537 | 0.13861 | 0.10891 | 0.0396 | 0.0297 | | | | | ASV_142 | 0.7524752 | 0.47525 | 0.28713 | 0.0495 | 0.0693069 | 0.0099 | 0.0297 |
| ASV_61 | 0.0297 | 0.28713 | 0.13861 | 0.0297 | | | | | ASV_1430 | 0.5643564 | 0.61386 | 0.31683 | 0.0099 | 0.0792079 | 0.6238 | 0.0297 |
| ASV_627 | 0.34653 | 0.0198 | 0.35644 | 0.0297 | | | | | ASV_145 | 0.0594059 | 0.0297 | 0.64356 | 0.12871 | 0.2871287 | 0.2277 | 0.0297 |
| ASV_684 | 0.10891 | 0.55446 | 0.0297 | 0.0297 | | | | | ASV_1545 | 0.1188119 | 0.0099 | 0.13861 | 0.15842 | 0.6336634 | 0.6139 | 0.0297 |
| ASV_954 | 0.63366 | 0.0099 | 0.41584 | 0.0297 | | | | | ASV_170 | 0.049505 | 0.07921 | 0.0099 | 0.23762 | 0.2178218 | 0.7327 | 0.0297 |

| | | | | |
|---|---|---|---|---|
| ASV_999 | 0.0297 | 0.09901 | 0.30693 | 0.0297 |
| ASV_117 | 0.0297 | 0.20792 | 0.14851 | 0.0396 |
| ASV_128 | 0.47525 | 0.0198 | 0.11881 | 0.0396 |
| ASV_13 | 0.42574 | 0.43564 | 0.0099 | 0.0396 |
| ASV_198 | 0.0396 | 0.50495 | 0.21782 | 0.0396 |
| ASV_262 | 0.13861 | 0.38614 | 0.05941 | 0.0396 |
| ASV_32 | 0.41584 | 0.0297 | 0.27723 | 0.0396 |
| ASV_337 | 0.0297 | 0.33663 | 0.26733 | 0.0396 |
| ASV_470 | 0.0099 | 0.29703 | 0.69307 | 0.0396 |
| ASV_622 | 0.0297 | 0.12871 | 0.15842 | 0.0396 |
| ASV_654 | 0.15842 | 0.22772 | 0.22772 | 0.0396 |
| ASV_73 | 0.08911 | 0.0297 | 0.06931 | 0.0396 |
| ASV_74 | 0.0198 | 0.80198 | 0.09901 | 0.0396 |
| ASV_920 | 0.0198 | 0.48515 | 0.46535 | 0.0396 |
| ASV_97 | 0.23762 | 0.38614 | 0.0099 | 0.0396 |
| ASV_111 | 0.22772 | 0.0396 | 0.12871 | 0.0495 |
| ASV_176 | 0.32673 | 0.37624 | 0.07921 | 0.0495 |
| ASV_233 | 0.0198 | 0.58416 | 0.15842 | 0.0495 |
| ASV_312 | 0.05941 | 0.0396 | 0.24752 | 0.0495 |
| ASV_404 | 0.10891 | 0.0198 | 0.50495 | 0.0495 |
| ASV_446 | 0.39604 | 0.0297 | 0.21782 | 0.0495 |
| ASV_670 | 0.0198 | 0.41584 | 0.52475 | 0.0495 |
| ASV_739 | 0.0297 | 0.45545 | 0.39604 | 0.0495 |
| ASV_826 | 0.0198 | 0.65347 | 0.0297 | 0.0495 |
| ASV_828 | 0.0099 | 0.55446 | 0.23762 | 0.0495 |

| | | | | | | | |
|---|---|---|---|---|---|---|---|
| ASV_24 | 0.3267327 | 0.0198 | 0.32673 | 0.0198 | 0.0792079 | 0.4851 | 0.0297 |
| ASV_257 | 0.0594059 | 0.0099 | 0.0198 | 0.41584 | 0.3168317 | 0.8812 | 0.0297 |
| ASV_299 | 0.5049505 | 0.61386 | 0.32673 | 0.53465 | 0.3069307 | 0.0099 | 0.0297 |
| ASV_343 | 0.1782178 | 0.15842 | 0.26733 | 0.42574 | 0.029703 | 0.2178 | 0.0297 |
| ASV_358 | 0.3267327 | 0.29703 | 0.0396 | 0.36634 | 0.1089109 | 0.8317 | 0.0297 |
| ASV_623 | 0.1089109 | 0.36634 | 0.42574 | 0.17822 | 0.009901 | 0.5941 | 0.0297 |
| ASV_92 | 0.980198 | 0.31683 | 0.10891 | 0.50495 | 0.009901 | 0.0396 | 0.0297 |
| ASV_999 | 0.0990099 | 0.47525 | 0.82178 | 0.07921 | 0.5049505 | 0.0099 | 0.0297 |
| ASV_115 | 0.4653465 | 0.15842 | 0.27723 | 0.19802 | 0.0693069 | 0.0792 | 0.0396 |
| ASV_126 | 0.049505 | 0.0198 | 0.08911 | 0.11881 | 0.8316832 | 0.0495 | 0.0396 |
| ASV_139 | 0.5346535 | 0.0099 | 0.31683 | 0.19802 | 0.0792079 | 0.8218 | 0.0396 |
| ASV_147 | 0.4554455 | 0.15842 | 0.05941 | 0.0198 | 0.0990099 | 0.7822 | 0.0396 |
| ASV_171 | 0.1188119 | 0.15842 | 0.44554 | 0.47525 | 0.1188119 | 0.1188 | 0.0396 |
| ASV_212 | 0.019802 | 0.28713 | 0.37624 | 0.0198 | 0.029703 | 0.0693 | 0.0396 |
| ASV_234 | 0.0792079 | 0.43564 | 0.0297 | 0.0495 | 0.1287129 | 0.1881 | 0.0396 |
| ASV_251 | 0.7029703 | 0.0495 | 0.05941 | 0.18812 | 0.1089109 | 0.2277 | 0.0396 |
| ASV_27 | 0.039604 | 0.24752 | 0.43564 | 0.19802 | 0.019802 | 0.7525 | 0.0396 |
| ASV_339 | 0.8217822 | 0.05941 | 0.31683 | 0.79208 | 0.2277228 | 0.0198 | 0.0396 |
| ASV_341 | 0.1881188 | 0.0099 | 0.10891 | 0.10891 | 0.2970297 | 0.9901 | 0.0396 |
| ASV_356 | 0.5445545 | 0.19802 | 0.32673 | 0.19802 | 0.3663366 | 0.0495 | 0.0396 |
| ASV_47 | 0.0594059 | 0.38614 | 0.0495 | 0.49505 | 0.039604 | 0.4752 | 0.0396 |
| ASV_553 | 0.0891089 | 0.76238 | 0.07921 | 0.0099 | 0.029703 | 0.8317 | 0.0396 |
| ASV_56 | 0.049505 | 0.0198 | 0.56436 | 0.05941 | 0.3267327 | 0.1386 | 0.0396 |
| ASV_692 | 0.0693069 | 0.18812 | 0.0396 | 0.39604 | 0.2277228 | 0.2574 | 0.0396 |
| ASV_80 | 0.7920792 | 0.28713 | 0.0495 | 0.13861 | 0.2178218 | 0.0297 | 0.0396 |
| ASV_820 | 0.0891089 | 0.0297 | 0.62376 | 0.26733 | 0.5445545 | 0.3861 | 0.0396 |
| ASV_828 | 0.8514851 | 0.93069 | 0.06931 | 0.61386 | 0.009901 | 0.0792 | 0.0396 |
| ASV_83 | 0.4752475 | 0.75248 | 0.11881 | 0.0396 | 0.1188119 | 0.0099 | 0.0396 |
| ASV_93 | 0.1485149 | 0.48515 | 0.15842 | 0.20792 | 0.019802 | 0.1089 | 0.0396 |
| ASV_100 | 0.019802 | 0.87129 | 0.42574 | 0.0495 | 0.1584158 | 0.0297 | 0.0495 |
| ASV_102 | 0.3366337 | 0.0099 | 0.16832 | 0.42574 | 0.3366337 | 0.4257 | 0.0495 |
| ASV_132 | 0.7425743 | 0.0396 | 0.35644 | 0.18812 | 0.2475248 | 0.0396 | 0.0495 |
| ASV_154 | 0.2871287 | 0.06931 | 0.61386 | 0.38614 | 0.0792079 | 0.1089 | 0.0495 |
| ASV_2529 | 0.4851485 | 0.0297 | 0.65347 | 0.07921 | 0.2673267 | 0.1881 | 0.0495 |
| ASV_264 | 0.5841584 | 0.0198 | 0.18812 | 0.32673 | 0.1089109 | 0.099 | 0.0495 |
| ASV_286 | 0.3069307 | 0.16832 | 0.74257 | 0.16832 | 0.0792079 | 0.0198 | 0.0495 |
| ASV_371 | 0.5841584 | 0.61386 | 0.40594 | 0.0099 | 0.2376238 | 0.2772 | 0.0495 |
| ASV_399 | 0.029703 | 0.86139 | 0.15842 | 0.89109 | 0.1683168 | 0.099 | 0.0495 |
| ASV_428 | 0.5544554 | 0.15842 | 0.77228 | 0.58416 | 0.019802 | 0.0396 | 0.0495 |
| ASV_470 | 0.029703 | 0.06931 | 0.76238 | 0.11881 | 0.3168317 | 0.8416 | 0.0495 |
| ASV_479 | 0.1881188 | 0.15842 | 0.16832 | 0.07921 | 0.1584158 | 0.1386 | 0.0495 |
| ASV_52 | 0.9009901 | 0.0396 | 0.06931 | 0.0297 | 0.7425743 | 0.0099 | 0.0495 |
| ASV_616 | 0.029703 | 0.63366 | 0.29703 | 0.10891 | 0.1089109 | 0.1485 | 0.0495 |
| ASV_747 | 0.1089109 | 0.05941 | 0.18812 | 0.13861 | 0.019802 | 0.9802 | 0.0495 |
| ASV_758 | 0.1881188 | 0.50495 | 0.0099 | 0.75248 | 0.1980198 | 0.0198 | 0.0495 |
| ASV_920 | 0.0594059 | 0.56436 | 0.07921 | 0.0198 | 0.3960396 | 0.2376 | 0.0495 |

| Selected ASVs based on all factors | |
|---|---|
| ASV | Taxonomy |
| ASV_103 | k__Bacteria; p__Proteobacteria; c__Gammaproteobacteria; o__Burkholderiales; f__Alcaligenaceae; g__Ampullimonas |
| ASV_109 | k__Bacteria; p__Proteobacteria; c__Alphaproteobacteria; o__Sphingomonadales; f__Sphingomonadaceae |
| ASV_110 | k__Bacteria; p__Proteobacteria; c__Gammaproteobacteria; o__Burkholderiales; f__Oxalobacteraceae |
| ASV_1134 | k__Bacteria; p__Proteobacteria; c__Gammaproteobacteria; o__Burkholderiales |
| ASV_115 | k__Bacteria; p__Proteobacteria; c__Gammaproteobacteria; o__Burkholderiales; f__Comamonadaceae; g__Variovorax |
| ASV_116 | k__Bacteria; p__Actinobacteriota; c__Actinobacteria; o__Micrococcales; f__Microbacteriaceae; g__Lysinimonas |
| ASV_118 | k__Bacteria; p__Proteobacteria; c__Gammaproteobacteria; o__Pseudomonadales; f__Pseudomonadaceae; g__Pseudomonas |
| ASV_120 | k__Bacteria; p__Proteobacteria; c__Gammaproteobacteria; o__Burkholderiales; f__Comamonadaceae |
| ASV_123 | k__Bacteria; p__Bacteroidota; c__Bacteroidia; o__Flavobacteriales; f__Flavobacteriaceae; g__Flavobacterium |
| ASV_134 | k__Bacteria; p__Proteobacteria; c__Gammaproteobacteria; o__Burkholderiales; f__Oxalobacteraceae |
| ASV_145 | k__Bacteria; p__Proteobacteria; c__Alphaproteobacteria |
| ASV_1511 | k__Bacteria; p__Proteobacteria; c__Alphaproteobacteria; o__Caulobacterales; f__Caulobacteraceae |
| ASV_154 | k__Bacteria; p__Actinobacteriota; c__Actinobacteria; o__Propionibacteriales; f__Nocardioidaceae; g__Aeromicrobium |
| ASV_158 | k__Bacteria; p__Proteobacteria; c__Gammaproteobacteria; o__Burkholderiales; f__Oxalobacteraceae; g__Duganella |
| ASV_174 | k__Bacteria; p__Proteobacteria; c__Gammaproteobacteria; o__Burkholderiales; f__Methylophilaceae |
| ASV_176 | k__Bacteria; p__Actinobacteriota; c__Actinobacteria; o__Micrococcales; f__Microbacteriaceae |
| ASV_177 | k__Bacteria; p__Proteobacteria; c__Gammaproteobacteria; o__Pseudomonadales |
| ASV_178 | k__Bacteria; p__Proteobacteria; c__Alphaproteobacteria; o__Sphingomonadales; f__Sphingomonadaceae; g__Sphingobium |
| ASV_194 | k__Bacteria; p__Proteobacteria; c__Gammaproteobacteria; o__Pseudomonadales; f__Pseudomonadaceae; g__Pseudomonas |
| ASV_198 | k__Bacteria; p__Actinobacteriota; c__Actinobacteria; o__Frankiales; f__Cryptosporangiaceae; g__Cryptosporangium |
| ASV_2 | k__Bacteria; p__Proteobacteria; c__Gammaproteobacteria; o__Pseudomonadales; f__Pseudomonadaceae; g__Pseudomonas |
| ASV_200 | k__Bacteria; p__Actinobacteriota; c__Actinobacteria; o__Micrococcales; f__Microbacteriaceae; g__Subtercola |
| ASV_206 | k__Bacteria; p__Actinobacteriota; c__Actinobacteria; o__Micrococcales; f__Microbacteriaceae |
| ASV_208 | k__Bacteria; p__Proteobacteria; c__Alphaproteobacteria; o__Rhizobiales; f__Xanthobacteraceae; g__Tardiphaga |
| ASV_212 | k__Bacteria; p__Proteobacteria; c__Gammaproteobacteria; o__Pseudomonadales |
| ASV_220 | k__Bacteria; p__Proteobacteria; c__Gammaproteobacteria; o__Burkholderiales; f__Oxalobacteraceae |
| ASV_232 | k__Bacteria; p__Actinobacteriota; c__Actinobacteria; o__Micrococcales; f__Microbacteriaceae; g__Subtercola; s__Subtercola boreus |
| ASV_235 | k__Bacteria; p__Proteobacteria; c__Alphaproteobacteria; o__Rhizobiales; f__Rhizobiaceae; g__Allorhizobium-Neorhizobium-Pararhizobium-Rhizobium |
| ASV_24 | k__Bacteria; p__Proteobacteria; c__Gammaproteobacteria; o__Burkholderiales; f__Oxalobacteraceae; g__Duganella |
| ASV_242 | k__Bacteria; p__Proteobacteria; c__Gammaproteobacteria; o__Burkholderiales; f__Oxalobacteraceae |
| ASV_244 | k__Bacteria; p__Proteobacteria; c__Gammaproteobacteria; o__Pseudomonadales |
| ASV_251 | k__Bacteria; p__Proteobacteria; c__Gammaproteobacteria; o__Burkholderiales; f__Burkholderiaceae; g__Burkholderia-Caballeronia-Paraburkholderia |
| ASV_252 | k__Bacteria; p__Proteobacteria; c__Gammaproteobacteria; o__Burkholderiales; f__Oxalobacteraceae; g__Duganella |
| ASV_255 | k__Bacteria; p__Proteobacteria; c__Alphaproteobacteria; o__Sphingomonadales; f__Sphingomonadaceae |
| ASV_257 | k__Bacteria; p__Actinobacteriota; c__Actinobacteria; o__Pseudonocardiales; f__Pseudonocardiaceae; g__Pseudonocardia; s__Pseudonocardia xinjiangensis |
| ASV_264 | k__Bacteria; p__Proteobacteria; c__Alphaproteobacteria |
| ASV_265 | k__Bacteria; p__Proteobacteria; c__Alphaproteobacteria; o__Sphingomonadales; f__Sphingomonadaceae; g__Novosphingobium |
| ASV_270 | k__Bacteria; p__Proteobacteria; c__Alphaproteobacteria; o__Rhizobiales; f__Pleomorphomonadaceae; g__Hartmannibacter |
| ASV_272 | k__Bacteria; p__Proteobacteria; c__Alphaproteobacteria; o__Rhizobiales |
| ASV_274 | k__Bacteria; p__Actinobacteriota; c__Actinobacteria; o__Micrococcales; f__Microbacteriaceae; g__Lysinimonas |
| ASV_275 | k__Bacteria; p__Proteobacteria; c__Alphaproteobacteria; o__Rhizobiales; f__Rhizobiaceae |
| ASV_278 | k__Bacteria; p__Proteobacteria; c__Gammaproteobacteria; o__Burkholderiales; f__Comamonadaceae; g__Xylophilus |

| | |
|---|---|
| ASV_280 | k__Bacteria; p__Proteobacteria; c__Alphaproteobacteria; o__Sphingomonadales; f__Sphingomonadaceae; g__Sphingomonas |
| ASV_284 | k__Bacteria; p__Actinobacteriota; c__Actinobacteria; o__Micrococcales; f__Microbacteriaceae; g__Herbiconiux; s__Herbiconiux solani |
| ASV_285 | k__Bacteria; p__Actinobacteriota; c__Actinobacteria; o__Micrococcales; f__Microbacteriaceae |
| ASV_287 | k__Bacteria; p__Proteobacteria; c__Alphaproteobacteria; o__Rhizobiales; f__Rhizobiaceae; g__Allorhizobium-Neorhizobium-Pararhizobium-Rhizobium |
| ASV_290 | k__Bacteria; p__Proteobacteria; c__Alphaproteobacteria; o__Rhizobiales; f__Rhizobiaceae |
| ASV_295 | k__Bacteria; p__Bacteroidota; c__Bacteroidia; o__Sphingobacteriales; f__Sphingobacteriaceae; g__Pedobacter |
| ASV_297 | k__Bacteria; p__Proteobacteria |
| ASV_299 | k__Bacteria; p__Proteobacteria; c__Alphaproteobacteria; o__Rhizobiales; f__Rhizobiaceae |
| ASV_32 | k__Bacteria; p__Proteobacteria; c__Alphaproteobacteria; o__Rhizobiales; f__Rhizobiaceae; g__Allorhizobium-Neorhizobium-Pararhizobium-Rhizobium |
| ASV_335 | k__Bacteria; p__Proteobacteria; c__Alphaproteobacteria |
| ASV_336 | k__Bacteria; p__Proteobacteria; c__Alphaproteobacteria; o__Rhizobiales; f__Rhizobiaceae; g__Candidatus Liberibacter |
| ASV_339 | k__Bacteria; p__Proteobacteria; c__Alphaproteobacteria |
| ASV_35 | k__Bacteria; p__Proteobacteria; c__Alphaproteobacteria; o__Rhizobiales; f__Rhizobiaceae; g__Candidatus Liberibacter |
| ASV_359 | k__Bacteria |
| ASV_36 | k__Bacteria; p__Proteobacteria; c__Gammaproteobacteria; o__Pseudomonadales; f__Pseudomonadaceae; g__Pseudomonas; s__Pseudomonas syringae |
| ASV_363 | k__Bacteria; p__Proteobacteria; c__Alphaproteobacteria; o__Caulobacterales; f__Caulobacteraceae; g__Asticcacaulis; s__Asticcacaulis benevestitus |
| ASV_366 | k__Bacteria; p__Proteobacteria; c__Gammaproteobacteria; o__Burkholderiales; f__Oxalobacteraceae; g__Duganella |
| ASV_37 | k__Bacteria; p__Proteobacteria; c__Gammaproteobacteria; o__Burkholderiales; f__Comamonadaceae |
| ASV_370 | k__Bacteria; p__Proteobacteria; c__Alphaproteobacteria; o__Caulobacterales; f__Caulobacteraceae; g__Caulobacter; s__Caulobacter henricii |
| ASV_375 | k__Bacteria; p__Actinobacteriota; c__Actinobacteria; o__Micrococcales; f__Microbacteriaceae |
| ASV_385 | k__Bacteria; p__Proteobacteria; c__Gammaproteobacteria; o__Pseudomonadales |
| ASV_39 | k__Bacteria; p__Proteobacteria; c__Gammaproteobacteria; o__Burkholderiales; f__Burkholderiaceae |
| ASV_403 | k__Bacteria; p__Actinobacteriota; c__Actinobacteria; o__Kineosporiales; f__Kineosporiaceae |
| ASV_404 | k__Bacteria; p__Proteobacteria; c__Alphaproteobacteria; o__Rhizobiales; f__Rhizobiaceae; g__Allorhizobium-Neorhizobium-Pararhizobium-Rhizobium |
| ASV_407 | k__Bacteria; p__Proteobacteria; c__Gammaproteobacteria; o__Burkholderiales; f__Comamonadaceae; g__Rhizobacter |
| ASV_421 | k__Bacteria; p__Actinobacteriota; c__Actinobacteria; o__Micrococcales; f__Microbacteriaceae; g__Parafrigoribacterium |
| ASV_440 | k__Bacteria; p__Actinobacteriota; c__Actinobacteria; o__Corynebacteriales; f__Nocardiaceae; g__Rhodococcus |
| ASV_445 | k__Bacteria; p__Proteobacteria; c__Alphaproteobacteria; o__Rhizobiales |
| ASV_451 | k__Bacteria; p__Actinobacteriota; c__Actinobacteria |
| ASV_455 | k__Bacteria; p__Proteobacteria; c__Gammaproteobacteria; o__Burkholderiales; f__Oxalobacteraceae |
| ASV_472 | k__Bacteria; p__Proteobacteria; c__Alphaproteobacteria |
| ASV_474 | k__Bacteria; p__Actinobacteriota; c__Actinobacteria; o__Micrococcales; f__Microbacteriaceae |
| ASV_488 | k__Bacteria; p__Proteobacteria; c__Gammaproteobacteria; o__Xanthomonadales; f__Rhodanobacteraceae |
| ASV_491 | k__Bacteria; p__Proteobacteria; c__Alphaproteobacteria; o__Rhizobiales; f__Rhizobiaceae |
| ASV_492 | k__Bacteria; p__Proteobacteria; c__Alphaproteobacteria; o__Rhizobiales; f__Rhizobiaceae; g__Allorhizobium-Neorhizobium-Pararhizobium-Rhizobium |
| ASV_495 | k__Bacteria; p__Actinobacteriota; c__Actinobacteria |
| ASV_504 | k__Bacteria; p__Actinobacteriota; c__Thermoleophilia; o__Solirubrobacterales; f__67-14 |
| ASV_506 | k__Bacteria; p__Proteobacteria; c__Gammaproteobacteria; o__Burkholderiales; f__Oxalobacteraceae |
| ASV_51 | k__Bacteria; p__Proteobacteria; c__Alphaproteobacteria; o__Rhizobiales; f__Rhizobiaceae; g__Allorhizobium-Neorhizobium-Pararhizobium-Rhizobium |
| ASV_537 | k__Bacteria; p__Proteobacteria; c__Alphaproteobacteria |
| ASV_544 | k__Bacteria; p__Proteobacteria; c__Alphaproteobacteria; o__Rhizobiales; f__Rhizobiaceae; g__Allorhizobium-Neorhizobium-Pararhizobium-Rhizobium |
| ASV_56 | k__Bacteria; p__Proteobacteria; c__Gammaproteobacteria; o__Enterobacterales |
| ASV_560 | k__Bacteria; p__Proteobacteria; c__Alphaproteobacteria; o__Sphingomonadales; f__Sphingomonadaceae; g__Novosphingobium |
| ASV_575 | k__Bacteria; p__Proteobacteria; c__Gammaproteobacteria; o__Burkholderiales; f__Comamonadaceae |
| ASV_59 | k__Bacteria; p__Proteobacteria; c__Gammaproteobacteria; o__Pseudomonadales; f__Pseudomonadaceae; g__Pseudomonas |
| ASV_600 | k__Bacteria; p__Actinobacteriota; c__Actinobacteria; o__Corynebacteriales; f__Nocardiaceae |

| | |
|---|---|
| ASV_62 | k__Bacteria; p__Proteobacteria; c__Gammaproteobacteria; o__Burkholderiales; f__Oxalobacteraceae; g__Duganella; s__Duganella zoogloeoides |
| ASV_634 | k__Bacteria; p__Proteobacteria; c__Alphaproteobacteria |
| ASV_639 | k__Bacteria; p__Actinobacteriota; c__Actinobacteria; o__Frankiales; f__Nakamurellaceae; g__Nakamurella |
| ASV_64 | k__Bacteria; p__Proteobacteria; c__Gammaproteobacteria; o__Enterobacterales |
| ASV_662 | k__Bacteria; p__Proteobacteria; c__Alphaproteobacteria; o__Acetobacterales; f__Acetobacteraceae; g__Acidisoma; s__Acidisoma tundrae |
| ASV_665 | k__Bacteria; p__Proteobacteria; c__Alphaproteobacteria; o__Rhizobiales; f__Rhizobiaceae; g__Allorhizobium-Neorhizobium-Pararhizobium-Rhizobium |
| ASV_689 | k__Bacteria; p__Actinobacteriota; c__Actinobacteria; o__Micrococcales; f__Microbacteriaceae |
| ASV_693 | k__Bacteria; p__Proteobacteria; c__Alphaproteobacteria |
| ASV_733 | k__Bacteria; p__Proteobacteria; c__Gammaproteobacteria; o__Steroidobacterales; f__Steroidobacteraceae |
| ASV_736 | k__Bacteria; p__Actinobacteriota; c__Actinobacteria; o__Micrococcales; f__Microbacteriaceae |
| ASV_78 | k__Bacteria; p__Proteobacteria; c__Gammaproteobacteria; o__Enterobacterales |
| ASV_83 | k__Bacteria; p__Proteobacteria; c__Gammaproteobacteria; o__Burkholderiales; f__Oxalobacteraceae |
| ASV_87 | k__Bacteria; p__Proteobacteria; c__Gammaproteobacteria; o__Enterobacterales |
| ASV_876 | k__Bacteria; p__Acidobacteriota; c__Acidobacteriae; o__Acidobacteriales; f__Acidobacteriaceae (Subgroup 1); g__Terriglobus |
| ASV_88 | k__Bacteria; p__Proteobacteria; c__Alphaproteobacteria; o__Sphingomonadales; f__Sphingomonadaceae; g__Novosphingobium |
| ASV_91 | k__Bacteria; p__Proteobacteria; c__Gammaproteobacteria; o__Enterobacterales; f__Morganellaceae; g__Arsenophonus |
| ASV_920 | k__Bacteria; p__Proteobacteria; c__Alphaproteobacteria; o__Sphingomonadales; f__Sphingomonadaceae; g__Novosphingobium |
| ASV_93 | k__Bacteria; p__Bacteroidota; c__Bacteroidia; o__Flavobacteriales; f__Flavobacteriaceae; g__Flavobacterium |
| ASV_95 | k__Bacteria; p__Proteobacteria; c__Gammaproteobacteria; o__Burkholderiales; f__Oxalobacteraceae |
| ASV_98 | k__Bacteria; p__Proteobacteria; c__Gammaproteobacteria; o__Burkholderiales; f__Alcaligenaceae; g__Ampullimonas |
| ASV_1 | k__Bacteria; p__Proteobacteria; c__Gammaproteobacteria; o__Enterobacterales; f__Enterobacteriaceae; g__Escherichia-Shigella |
| ASV_10 | k__Bacteria; p__Proteobacteria; c__Gammaproteobacteria; o__Enterobacterales; f__Enterobacteriaceae; g__Escherichia-Shigella |
| ASV_107 | k__Bacteria; p__Proteobacteria; c__Gammaproteobacteria; o__Pseudomonadales; f__Pseudomonadaceae; g__Pseudomonas |
| ASV_111 | k__Bacteria; p__Actinobacteriota; c__Actinobacteria; o__Streptomycetales; f__Streptomycetaceae; g__Streptomyces |
| ASV_117 | k__Bacteria; p__Actinobacteriota; c__Actinobacteria; o__Micrococcales; f__Microbacteriaceae; g__Galbitalea |
| ASV_119 | k__Bacteria; p__Actinobacteriota; c__Actinobacteria; o__Kineosporiales; f__Kineosporiaceae; g__Kineosporia; s__Kineosporia aurantiaca |
| ASV_126 | k__Bacteria; p__Bacteroidota; c__Bacteroidia; o__Sphingobacteriales; f__Sphingobacteriaceae; g__Pedobacter |
| ASV_128 | k__Bacteria; p__Proteobacteria; c__Alphaproteobacteria; o__Rhizobiales; f__Rhizobiaceae; g__Allorhizobium-Neorhizobium-Pararhizobium-Rhizobium |
| ASV_13 | k__Bacteria; p__Proteobacteria; c__Gammaproteobacteria; o__Burkholderiales; f__Oxalobacteraceae; g__Duganella |
| ASV_130 | k__Bacteria; p__Actinobacteriota; c__Actinobacteria; o__Frankiales; f__Cryptosporangiaceae; g__Cryptosporangium; s__Cryptosporangium aurantiacum |
| ASV_137 | k__Bacteria; p__Proteobacteria; c__Gammaproteobacteria; o__Burkholderiales; f__Oxalobacteraceae |
| ASV_14 | k__Bacteria; p__Proteobacteria; c__Gammaproteobacteria; o__Enterobacterales; f__Enterobacteriaceae |
| ASV_144 | k__Bacteria; p__Proteobacteria; c__Gammaproteobacteria; o__Xanthomonadales; f__Xanthomonadaceae; g__Pseudoxanthomonas |
| ASV_160 | k__Bacteria; p__Actinobacteriota; c__Actinobacteria; o__Micrococcales; f__Microbacteriaceae |
| ASV_1635 | k__Bacteria; p__Proteobacteria; c__Alphaproteobacteria; o__Sphingomonadales; f__Sphingomonadaceae; g__Sphingomonas |
| ASV_167 | k__Bacteria; p__Proteobacteria; c__Gammaproteobacteria; o__Burkholderiales; f__Comamonadaceae; g__Polaromonas |
| ASV_17 | k__Bacteria; p__Proteobacteria; c__Gammaproteobacteria; o__Enterobacterales; f__Erwiniaceae; g__Erwinia |
| ASV_170 | k__Bacteria; p__Proteobacteria; c__Alphaproteobacteria; o__Sphingomonadales; f__Sphingomonadaceae; g__Rhizorhapis |
| ASV_18 | k__Bacteria; p__Proteobacteria; c__Gammaproteobacteria; o__Enterobacterales; f__Enterobacteriaceae; g__Escherichia-Shigella |
| ASV_182 | k__Bacteria; p__Actinobacteriota; c__Actinobacteria; o__Micrococcales; f__Microbacteriaceae; g__Lysinimonas; s__Lysinimonas soli |
| ASV_184 | k__Bacteria; p__Proteobacteria; c__Alphaproteobacteria; o__Rhizobiales; f__Xanthobacteraceae; g__Bradyrhizobium |
| ASV_189 | k__Bacteria; p__Proteobacteria; c__Alphaproteobacteria; o__Caulobacterales; f__Caulobacteraceae; g__Caulobacter |
| ASV_19 | k__Bacteria; p__Proteobacteria; c__Alphaproteobacteria; o__Sphingomonadales; f__Sphingomonadaceae; g__Sphingomonas; s__Sphingomonas faeni |
| ASV_199 | k__Bacteria; p__Proteobacteria; c__Alphaproteobacteria; o__Rhizobiales; f__Xanthobacteraceae; g__Rhodopseudomonas |
| ASV_21 | k__Bacteria; p__Proteobacteria; c__Gammaproteobacteria; o__Pseudomonadales; f__Pseudomonadaceae; g__Pseudomonas |
| ASV_210 | k__Bacteria; p__Proteobacteria; c__Alphaproteobacteria; o__Rhizobiales; f__Xanthobacteraceae; g__Tardiphaga |

| | |
|---|---|
| ASV_211 | k__Bacteria; p__Actinobacteriota; c__Actinobacteria; o__Micromonosporales; f__Micromonosporaceae; g__Actinoplanes |
| ASV_218 | k__Bacteria; p__Proteobacteria; c__Alphaproteobacteria; o__Rhizobiales; f__Xanthobacteraceae |
| ASV_226 | k__Bacteria; p__Actinobacteriota; c__Actinobacteria; o__Streptomycetales; f__Streptomycetaceae; g__Streptomyces |
| ASV_229 | k__Bacteria; p__Proteobacteria; c__Alphaproteobacteria; o__Rhizobiales; f__Xanthobacteraceae; g__Tardiphaga; s__Tardiphaga robiniae |
| ASV_233 | k__Bacteria; p__Proteobacteria; c__Gammaproteobacteria; o__Burkholderiales; f__Comamonadaceae |
| ASV_234 | k__Bacteria; p__Proteobacteria; c__Alphaproteobacteria; o__Sphingomonadales; f__Sphingomonadaceae; g__Novosphingobium; s__Novosphingobium barchaimii |
| ASV_238 | k__Bacteria; p__Proteobacteria; c__Alphaproteobacteria; o__Sphingomonadales; f__Sphingomonadaceae; g__Sphingomonas |
| ASV_250 | k__Bacteria; p__Proteobacteria; c__Gammaproteobacteria; o__Burkholderiales; f__Burkholderiaceae; g__Ralstonia |
| ASV_262 | k__Bacteria; p__Actinobacteriota; c__Actinobacteria; o__Frankiales; f__Acidothermaceae; g__Acidothermus |
| ASV_266 | k__Bacteria; p__Proteobacteria; c__Gammaproteobacteria; o__Burkholderiales; f__Comamonadaceae; g__Limnohabitans |
| ASV_27 | k__Bacteria; p__Proteobacteria; c__Gammaproteobacteria; o__Burkholderiales; f__Oxalobacteraceae; g__Janthinobacterium; s__Janthinobacterium lividum |
| ASV_28 | k__Bacteria; p__Proteobacteria; c__Gammaproteobacteria; o__Burkholderiales; f__Oxalobacteraceae; g__Janthinobacterium; s__Janthinobacterium lividum |
| ASV_293 | k__Bacteria; p__Proteobacteria; c__Gammaproteobacteria; o__Pseudomonadales; f__Moraxellaceae |
| ASV_3 | k__Bacteria; p__Proteobacteria; c__Gammaproteobacteria; o__Burkholderiales; f__Oxalobacteraceae; g__Janthinobacterium; s__Janthinobacterium lividum |
| ASV_301 | k__Bacteria; p__Actinobacteriota; c__Actinobacteria; o__Frankiales; f__Nakamurellaceae; g__Nakamurella |
| ASV_305 | k__Bacteria; p__Proteobacteria; c__Gammaproteobacteria; o__Burkholderiales; f__Burkholderiaceae; g__Burkholderia-Caballeronia-Paraburkholderia |
| ASV_308 | k__Bacteria; p__Firmicutes; c__Bacilli; o__Bacillales; f__Bacillaceae |
| ASV_309 | k__Bacteria; p__Proteobacteria; c__Gammaproteobacteria; o__Burkholderiales; f__Comamonadaceae; g__Variovorax; s__Variovorax paradoxus |
| ASV_312 | k__Bacteria; p__Actinobacteriota; c__Actinobacteria; o__Propionibacteriales; f__Propionibacteriaceae; g__Cutibacterium; s__Cutibacterium acnes |
| ASV_313 | k__Bacteria; p__Proteobacteria; c__Alphaproteobacteria; o__Rhizobiales; f__Rhizobiaceae; g__Allorhizobium-Neorhizobium-Pararhizobium-Rhizobium |
| ASV_314 | k__Bacteria; p__Bacteroidota; c__Bacteroidia; o__Sphingobacteriales; f__Sphingobacteriaceae; g__Mucilaginibacter; s__Mucilaginibacter pocheonensis |
| ASV_315 | k__Bacteria; p__Bacteroidota; c__Bacteroidia; o__Sphingobacteriales; f__Sphingobacteriaceae; g__Pedobacter |
| ASV_316 | k__Bacteria; p__Proteobacteria; c__Alphaproteobacteria; o__Sphingomonadales; f__Sphingomonadaceae |
| ASV_327 | k__Bacteria; p__Bacteroidota; c__Bacteroidia; o__Flavobacteriales; f__Flavobacteriaceae; g__Flavobacterium |
| ASV_337 | k__Bacteria; p__Actinobacteriota; c__Actinobacteria; o__Propionibacteriales; f__Nocardioidaceae; g__Aeromicrobium; s__Aeromicrobium ginsengisoli |
| ASV_34 | k__Bacteria; p__Proteobacteria; c__Gammaproteobacteria; o__Burkholderiales; f__Oxalobacteraceae; g__Massilia |
| ASV_347 | k__Bacteria; p__Proteobacteria; c__Gammaproteobacteria; o__Pseudomonadales; f__Pseudomonadaceae; g__Pseudomonas |
| ASV_352 | k__Bacteria; p__Proteobacteria; c__Gammaproteobacteria; o__Burkholderiales; f__Comamonadaceae; g__Rhizobacter |
| ASV_361 | k__Bacteria; p__Bacteroidota; c__Bacteroidia; o__Sphingobacteriales; f__Sphingobacteriaceae; g__Mucilaginibacter |
| ASV_367 | k__Bacteria; p__Proteobacteria; c__Alphaproteobacteria; o__Rhizobiales; f__Rhizobiaceae; g__Allorhizobium-Neorhizobium-Pararhizobium-Rhizobium |
| ASV_371 | k__Bacteria; p__Proteobacteria; c__Gammaproteobacteria; o__Burkholderiales; f__Methylophilaceae |
| ASV_38 | k__Bacteria; p__Proteobacteria; c__Gammaproteobacteria; o__Enterobacterales; f__Enterobacteriaceae; g__Escherichia-Shigella |
| ASV_387 | k__Bacteria; p__Bacteroidota; c__Bacteroidia; o__Chitinophagales; f__Chitinophagaceae; g__Puia |
| ASV_394 | k__Bacteria; p__Proteobacteria; c__Alphaproteobacteria; o__Rhizobiales; f__Rhizobiaceae; g__Aureimonas |
| ASV_398 | k__Bacteria; p__Firmicutes; c__Bacilli; o__Bacillales; f__Bacillaceae; g__Bacillus |
| ASV_399 | k__Bacteria; p__Proteobacteria; c__Alphaproteobacteria; o__Caulobacterales; f__Caulobacteraceae; g__Caulobacter |
| ASV_4 | k__Bacteria; p__Proteobacteria; c__Gammaproteobacteria; o__Burkholderiales; f__Burkholderiaceae; g__Ralstonia |
| ASV_41 | k__Bacteria; p__Proteobacteria; c__Gammaproteobacteria; o__Pseudomonadales; f__Pseudomonadaceae; g__Pseudomonas |
| ASV_413 | k__Bacteria; p__Firmicutes; c__Bacilli; o__Entomoplasmatales; f__Spiroplasmataceae; g__Spiroplasma |
| ASV_419 | k__Bacteria; p__Bacteroidota; c__Bacteroidia; o__Cytophagales; f__Microscillaceae |
| ASV_424 | k__Bacteria; p__Proteobacteria; c__Gammaproteobacteria; o__Burkholderiales; f__Neisseriaceae |
| ASV_439 | k__Bacteria; p__Proteobacteria; c__Alphaproteobacteria; o__Sphingomonadales; f__Sphingomonadaceae; g__Polymorphobacter |
| ASV_446 | k__Bacteria; p__Actinobacteriota; c__Actinobacteria; o__Micrococcales; f__Microbacteriaceae; g__Conyzicola; s__Conyzicola nivalis |
| ASV_447 | k__Bacteria; p__Proteobacteria; c__Alphaproteobacteria; o__Sphingomonadales; f__Sphingomonadaceae; g__Sphingomonas |
| ASV_47 | k__Bacteria; p__Proteobacteria; c__Gammaproteobacteria; o__Burkholderiales; f__Oxalobacteraceae |
| ASV_470 | k__Bacteria; p__Proteobacteria; c__Alphaproteobacteria; o__Caulobacterales; f__Caulobacteraceae; g__Brevundimonas; s__Brevundimonas staleyi |

| | |
|---|---|
| ASV_478 | k__Bacteria; p__Bacteroidota; c__Bacteroidia; o__Sphingobacteriales; f__Sphingobacteriaceae; g__Mucilaginibacter; s__Mucilaginibacter ginsenosidivorax |
| ASV_514 | k__Bacteria; p__Proteobacteria; c__Alphaproteobacteria; o__Sphingomonadales; f__Sphingomonadaceae; g__Sphingomonas |
| ASV_518 | k__Bacteria; p__Proteobacteria; c__Gammaproteobacteria; o__Burkholderiales; f__Comamonadaceae; g__Variovorax |
| ASV_585 | k__Bacteria; p__Proteobacteria; c__Alphaproteobacteria; o__Rhizobiales; f__Xanthobacteraceae; g__Ancylobacter |
| ASV_607 | k__Bacteria; p__Proteobacteria; c__Gammaproteobacteria; o__Burkholderiales; f__Burkholderiaceae; g__Cupriavidus; s__Cupriavidus basilensis |
| ASV_609 | k__Bacteria; p__Proteobacteria; c__Alphaproteobacteria; o__Caulobacterales; f__Caulobacteraceae; g__Caulobacter |
| ASV_61 | k__Bacteria; p__Proteobacteria; c__Gammaproteobacteria; o__Pseudomonadales; f__Pseudomonadaceae; g__Pseudomonas |
| ASV_616 | k__Bacteria; p__Proteobacteria; c__Gammaproteobacteria; o__Burkholderiales; f__Comamonadaceae; g__Xylophilus |
| ASV_622 | k__Bacteria; p__Proteobacteria; c__Alphaproteobacteria; o__Rhizobiales; f__Beijerinckiaceae; g__Bosea |
| ASV_627 | k__Bacteria; p__Proteobacteria; c__Alphaproteobacteria; o__Rhizobiales |
| ASV_629 | k__Bacteria; p__Firmicutes; c__Bacilli; o__Bacillales; f__Bacillaceae |
| ASV_640 | k__Bacteria; p__Actinobacteriota; c__Thermoleophilia; o__Solirubrobacterales; f__67-14 |
| ASV_65 | k__Bacteria; p__Proteobacteria; c__Gammaproteobacteria; o__Burkholderiales; f__Burkholderiaceae; g__Burkholderia-Caballeronia-Paraburkholderia |
| ASV_654 | k__Bacteria; p__Proteobacteria; c__Gammaproteobacteria; o__Xanthomonadales; f__Rhodanobacteraceae; g__Dokdonella; s__Dokdonella soli |
| ASV_66 | k__Bacteria; p__Actinobacteriota; c__Actinobacteria; o__Kineosporiales; f__Kineosporiaceae; g__Kineosporia |
| ASV_670 | k__Bacteria; p__Proteobacteria; c__Alphaproteobacteria; o__Sphingomonadales; f__Sphingomonadaceae; g__Sphingomonas |
| ASV_684 | k__Bacteria; p__Actinobacteriota; c__Actinobacteria |
| ASV_686 | k__Bacteria; p__Proteobacteria; c__Alphaproteobacteria; o__Rhizobiales; f__Rhizobiaceae |
| ASV_69 | k__Bacteria; p__Actinobacteriota; c__Actinobacteria; o__Micrococcales; f__Microbacteriaceae; g__Galbitalea |
| ASV_696 | k__Bacteria; p__Actinobacteriota; c__Actinobacteria; o__Frankiales |
| ASV_699 | k__Bacteria; p__Proteobacteria; c__Alphaproteobacteria; o__Sphingomonadales; f__Sphingomonadaceae; g__Novosphingobium |
| ASV_71 | k__Bacteria; p__Proteobacteria; c__Gammaproteobacteria; o__Burkholderiales; f__Oxalobacteraceae |
| ASV_73 | k__Bacteria; p__Proteobacteria; c__Gammaproteobacteria; o__Pseudomonadales; f__Pseudomonadaceae; g__Pseudomonas; s__Pseudomonas graminis |
| ASV_739 | k__Bacteria; p__Actinobacteriota; c__Actinobacteria; o__Kineosporiales; f__Kineosporiaceae; g__Kineosporia |
| ASV_74 | k__Bacteria; p__Proteobacteria; c__Gammaproteobacteria; o__Burkholderiales; f__Oxalobacteraceae; g__Duganella |
| ASV_747 | k__Bacteria; p__Proteobacteria; c__Gammaproteobacteria; o__Burkholderiales; f__Burkholderiaceae; g__Burkholderia-Caballeronia-Paraburkholderia |
| ASV_75 | k__Bacteria; p__Proteobacteria; c__Gammaproteobacteria; o__Enterobacterales |
| ASV_77 | k__Bacteria; p__Actinobacteriota; c__Actinobacteria; o__Frankiales; f__Cryptosporangiaceae; g__Cryptosporangium; s__Cryptosporangium mongoliense |
| ASV_8 | k__Bacteria; p__Proteobacteria; c__Gammaproteobacteria; o__Pseudomonadales; f__Pseudomonadaceae; g__Pseudomonas |
| ASV_82 | k__Bacteria; p__Chloroflexi; c__Chloroflexia; o__Chloroflexales; f__Roseiflexaceae |
| ASV_820 | k__Bacteria; p__Actinobacteriota; c__Actinobacteria; o__Frankiales; f__Frankiaceae; g__Jatrophihabitans |
| ASV_826 | k__Bacteria; p__Acidobacteriota; c__Acidobacteriae; o__Subgroup 2 |
| ASV_828 | k__Bacteria; p__Bacteroidota; c__Bacteroidia; o__Sphingobacteriales; f__Sphingobacteriaceae; g__Mucilaginibacter |
| ASV_84 | k__Bacteria; p__Actinobacteriota; c__Actinobacteria; o__Kineosporiales; f__Kineosporiaceae; g__Kineosporia |
| ASV_954 | k__Bacteria; p__Proteobacteria; c__Alphaproteobacteria; o__Rhizobiales; f__Kaistiaceae; g__Kaistia |
| ASV_97 | k__Bacteria; p__Proteobacteria; c__Gammaproteobacteria; o__Burkholderiales; f__Oxalobacteraceae; g__Massilia |
| ASV_999 | k__Bacteria; p__Proteobacteria; c__Alphaproteobacteria; o__Rhizobiales; f__Rhizobiaceae; g__Allorhizobium-Neorhizobium-Pararhizobium-Rhizobium |
| ASV_100 | k__Bacteria; p__Bacteroidota; c__Bacteroidia; o__Flavobacteriales; f__Flavobacteriaceae; g__Flavobacterium |
| ASV_102 | k__Bacteria; p__Proteobacteria; c__Gammaproteobacteria; o__Pseudomonadales; f__Pseudomonadaceae; g__Pseudomonas |
| ASV_104 | k__Bacteria; p__Proteobacteria; c__Gammaproteobacteria; o__Pseudomonadales; f__Pseudomonadaceae; g__Pseudomonas |
| ASV_106 | k__Bacteria; p__Proteobacteria; c__Gammaproteobacteria; o__Pseudomonadales; f__Pseudomonadaceae; g__Pseudomonas |
| ASV_11 | k__Bacteria; p__Proteobacteria; c__Gammaproteobacteria; o__Pseudomonadales; f__Pseudomonadaceae; g__Pseudomonas |
| ASV_113 | k__Bacteria; p__Proteobacteria; c__Gammaproteobacteria; o__Burkholderiales; f__Oxalobacteraceae |
| ASV_1157 | k__Bacteria; p__Proteobacteria; c__Gammaproteobacteria; o__Enterobacterales |
| ASV_12 | k__Bacteria; p__Proteobacteria; c__Alphaproteobacteria; o__Rhizobiales; f__Rhizobiaceae; g__Allorhizobium-Neorhizobium-Pararhizobium-Rhizobium |
| ASV_122 | k__Bacteria; p__Proteobacteria; c__Gammaproteobacteria; o__Burkholderiales; f__Comamonadaceae |

| | |
|---|---|
| ASV_124 | k__Bacteria; p__Proteobacteria; c__Gammaproteobacteria |
| ASV_132 | k__Bacteria; p__Proteobacteria; c__Gammaproteobacteria; o__Burkholderiales; f__Oxalobacteraceae; g__Duganella |
| ASV_135 | k__Bacteria; p__Actinobacteriota; c__Actinobacteria; o__Micrococcales; f__Microbacteriaceae; g__Frondihabitans |
| ASV_136 | k__Bacteria; p__Proteobacteria; c__Gammaproteobacteria; o__Enterobacterales; f__Erwiniaceae |
| ASV_1376 | k__Bacteria; p__Proteobacteria; c__Gammaproteobacteria; o__Burkholderiales; f__Oxalobacteraceae |
| ASV_138 | k__Bacteria; p__Proteobacteria; c__Gammaproteobacteria; o__Pseudomonadales; f__Pseudomonadaceae; g__Pseudomonas |
| ASV_139 | k__Bacteria; p__Proteobacteria; c__Gammaproteobacteria; o__Pseudomonadales; f__Pseudomonadaceae; g__Pseudomonas |
| ASV_140 | k__Bacteria; p__Proteobacteria; c__Gammaproteobacteria; o__Burkholderiales; f__Oxalobacteraceae; g__Herbaspirillum |
| ASV_141 | k__Bacteria; p__Proteobacteria; c__Gammaproteobacteria; o__Burkholderiales; f__Oxalobacteraceae |
| ASV_142 | k__Bacteria; p__Proteobacteria; c__Gammaproteobacteria; o__Enterobacterales; f__Erwiniaceae; g__Erwinia |
| ASV_143 | k__Bacteria; p__Proteobacteria; c__Gammaproteobacteria; o__Enterobacterales; f__Yersiniaceae; g__Rahnella1 |
| ASV_1430 | k__Bacteria; p__Actinobacteriota; c__Thermoleophilia; o__Solirubrobacterales; f__Solirubrobacteraceae; g__Conexibacter |
| ASV_147 | k__Bacteria; p__Proteobacteria; c__Gammaproteobacteria; o__Burkholderiales; f__Oxalobacteraceae; g__Duganella |
| ASV_150 | k__Bacteria; p__Proteobacteria; c__Gammaproteobacteria |
| ASV_1545 | k__Bacteria; p__Actinobacteriota; c__Actinobacteria; o__Micrococcales; f__Intrasporangiaceae; g__Lapillicoccus |
| ASV_157 | k__Bacteria; p__Proteobacteria; c__Gammaproteobacteria; o__Enterobacterales; f__Yersiniaceae; g__Rahnella1 |
| ASV_16 | k__Bacteria; p__Proteobacteria; c__Gammaproteobacteria; o__Burkholderiales; f__Oxalobacteraceae; g__Duganella |
| ASV_171 | k__Bacteria; p__Proteobacteria; c__Alphaproteobacteria; o__Sphingomonadales; f__Sphingomonadaceae; g__Sphingomonas; s__Sphingomonas faeni |
| ASV_172 | k__Bacteria; p__Proteobacteria; c__Gammaproteobacteria; o__Burkholderiales; f__Oxalobacteraceae |
| ASV_173 | k__Bacteria; p__Actinobacteriota; c__Actinobacteria; o__Pseudonocardiales; f__Pseudonocardiaceae; g__Pseudonocardia |
| ASV_183 | k__Bacteria; p__Proteobacteria; c__Gammaproteobacteria; o__Pseudomonadales; f__Pseudomonadaceae; g__Pseudomonas |
| ASV_186 | k__Bacteria; p__Proteobacteria; c__Gammaproteobacteria; o__Enterobacterales; f__Erwiniaceae; g__Pantoea |
| ASV_188 | k__Bacteria; p__Proteobacteria; c__Gammaproteobacteria; o__Pseudomonadales; f__Pseudomonadaceae; g__Pseudomonas; s__Pseudomonas abietaniphila |
| ASV_196 | k__Bacteria; p__Proteobacteria; c__Gammaproteobacteria; o__Pseudomonadales; f__Pseudomonadaceae; g__Pseudomonas |
| ASV_20 | k__Bacteria; p__Proteobacteria; c__Gammaproteobacteria; o__Pseudomonadales; f__Pseudomonadaceae; g__Pseudomonas; s__Pseudomonas syringae |
| ASV_205 | k__Bacteria; p__Proteobacteria; c__Gammaproteobacteria; o__Pseudomonadales; f__Pseudomonadaceae; g__Pseudomonas |
| ASV_209 | k__Bacteria; p__Proteobacteria; c__Gammaproteobacteria; o__Pseudomonadales; f__Pseudomonadaceae; g__Pseudomonas; s__Pseudomonas graminis |
| ASV_22 | k__Bacteria; p__Proteobacteria; c__Gammaproteobacteria; o__Pseudomonadales; f__Pseudomonadaceae; g__Pseudomonas |
| ASV_23 | k__Bacteria; p__Proteobacteria; c__Gammaproteobacteria; o__Pseudomonadales; f__Pseudomonadaceae; g__Pseudomonas |
| ASV_239 | k__Bacteria; p__Actinobacteriota; c__Actinobacteria; o__Micrococcales; f__Cellulomonadaceae; g__Cellulomonas |
| ASV_2529 | k__Bacteria; p__Proteobacteria; c__Gammaproteobacteria |
| ASV_263 | k__Bacteria; p__Bacteroidota; c__Bacteroidia; o__Sphingobacteriales; f__Sphingobacteriaceae; g__Pedobacter; s__Pedobacter westerhofensis |
| ASV_267 | k__Bacteria; p__Proteobacteria; c__Gammaproteobacteria; o__Enterobacterales |
| ASV_286 | k__Bacteria; p__Proteobacteria; c__Gammaproteobacteria; o__Enterobacterales |
| ASV_29 | k__Bacteria; p__Proteobacteria; c__Gammaproteobacteria; o__Pseudomonadales; f__Pseudomonadaceae; g__Pseudomonas |
| ASV_298 | k__Bacteria; p__Proteobacteria; c__Gammaproteobacteria; o__Pseudomonadales; f__Pseudomonadaceae; g__Pseudomonas |
| ASV_30 | k__Bacteria; p__Proteobacteria; c__Gammaproteobacteria; o__Burkholderiales; f__Oxalobacteraceae; g__Duganella |
| ASV_31 | k__Bacteria; p__Proteobacteria; c__Gammaproteobacteria; o__Pseudomonadales; f__Pseudomonadaceae; g__Pseudomonas |
| ASV_311 | k__Bacteria; p__Proteobacteria; c__Gammaproteobacteria; o__Burkholderiales; f__Oxalobacteraceae |
| ASV_320 | k__Bacteria; p__Proteobacteria; c__Alphaproteobacteria; o__Rhizobiales; f__Rhizobiaceae; g__Aureimonas |
| ASV_324 | k__Bacteria; p__Bacteroidota; c__Bacteroidia; o__Sphingobacteriales; f__Sphingobacteriaceae; g__Pedobacter |
| ASV_325 | k__Bacteria; p__Proteobacteria; c__Alphaproteobacteria; o__Sphingomonadales; f__Sphingomonadaceae |
| ASV_341 | k__Bacteria; p__Actinobacteriota; c__Actinobacteria; o__Kineosporiales; f__Kineosporiaceae; g__Kineosporia |
| ASV_343 | k__Bacteria; p__Proteobacteria; c__Gammaproteobacteria; o__Enterobacterales |
| ASV_348 | k__Bacteria; p__Proteobacteria; c__Gammaproteobacteria; o__Pseudomonadales; f__Pseudomonadaceae; g__Pseudomonas |
| ASV_356 | k__Bacteria; p__Proteobacteria; c__Alphaproteobacteria; o__Rhizobiales; f__Rhizobiaceae |

| | |
|---|---|
| ASV_358 | k__Bacteria; p__Actinobacteriota; c__Actinobacteria; o__Micrococcales; f__Microbacteriaceae; g__Galbitalea |
| ASV_42 | k__Bacteria; p__Proteobacteria; c__Gammaproteobacteria; o__Pseudomonadales; f__Pseudomonadaceae; g__Pseudomonas |
| ASV_423 | k__Bacteria; p__Proteobacteria; c__Gammaproteobacteria; o__Pseudomonadales; f__Pseudomonadaceae; g__Pseudomonas |
| ASV_428 | k__Bacteria; p__Proteobacteria; c__Gammaproteobacteria |
| ASV_435 | k__Bacteria; p__Actinobacteriota; c__Actinobacteria; o__Kineosporiales; f__Kineosporiaceae; g__Kineosporia |
| ASV_44 | k__Bacteria; p__Proteobacteria; c__Gammaproteobacteria; o__Pseudomonadales; f__Pseudomonadaceae; g__Pseudomonas |
| ASV_442 | k__Bacteria; p__Proteobacteria; c__Gammaproteobacteria; o__Burkholderiales; f__Comamonadaceae; g__Xylophilus |
| ASV_449 | k__Bacteria; p__Actinobacteriota; c__Actinobacteria; o__Corynebacteriales; f__Mycobacteriaceae; g__Mycobacterium |
| ASV_477 | k__Bacteria; p__Actinobacteriota; c__Actinobacteria; o__Micrococcales; f__Microbacteriaceae; g__Microbacterium |
| ASV_479 | k__Bacteria; p__Proteobacteria; c__Gammaproteobacteria |
| ASV_487 | k__Bacteria; p__Proteobacteria; c__Gammaproteobacteria; o__Burkholderiales; f__Oxalobacteraceae |
| ASV_49 | k__Bacteria; p__Proteobacteria; c__Gammaproteobacteria; o__Pseudomonadales; f__Pseudomonadaceae; g__Pseudomonas |
| ASV_5 | k__Bacteria; p__Proteobacteria; c__Gammaproteobacteria; o__Pseudomonadales; f__Pseudomonadaceae; g__Pseudomonas |
| ASV_508 | k__Bacteria; p__Proteobacteria; c__Alphaproteobacteria; o__Sphingomonadales; f__Sphingomonadaceae; g__Parablastomonas |
| ASV_52 | k__Bacteria; p__Proteobacteria; c__Gammaproteobacteria; o__Pseudomonadales; f__Pseudomonadaceae; g__Pseudomonas |
| ASV_523 | k__Bacteria; p__Proteobacteria; c__Gammaproteobacteria; o__Enterobacterales; f__Erwiniaceae; g__Pantoea |
| ASV_536 | k__Bacteria; p__Actinobacteriota; c__Actinobacteria; o__Micrococcales; f__Microbacteriaceae; g__Curtobacterium |
| ASV_549 | k__Bacteria; p__Proteobacteria; c__Gammaproteobacteria; o__Burkholderiales; f__Oxalobacteraceae; g__Massilia |
| ASV_55 | k__Bacteria; p__Proteobacteria; c__Gammaproteobacteria; o__Enterobacterales |
| ASV_553 | k__Bacteria; p__Proteobacteria; c__Gammaproteobacteria; o__Pseudomonadales; f__Pseudomonadaceae; g__Pseudomonas |
| ASV_554 | k__Bacteria; p__Proteobacteria; c__Gammaproteobacteria; o__Xanthomonadales; f__Xanthomonadaceae; g__Xanthomonas |
| ASV_58 | k__Bacteria; p__Proteobacteria; c__Gammaproteobacteria; o__Pseudomonadales; f__Pseudomonadaceae; g__Pseudomonas |
| ASV_6 | k__Bacteria; p__Proteobacteria; c__Gammaproteobacteria; o__Pseudomonadales; f__Pseudomonadaceae; g__Pseudomonas |
| ASV_60 | k__Bacteria; p__Proteobacteria; c__Gammaproteobacteria; o__Enterobacterales; f__Erwiniaceae; g__Erwinia |
| ASV_623 | k__Bacteria; p__Proteobacteria; c__Gammaproteobacteria; o__Pseudomonadales; f__Pseudomonadaceae; g__Pseudomonas |
| ASV_63 | k__Bacteria; p__Proteobacteria; c__Gammaproteobacteria; o__Pseudomonadales; f__Pseudomonadaceae; g__Pseudomonas |
| ASV_641 | k__Bacteria; p__Proteobacteria; c__Alphaproteobacteria; o__Rhizobiales; f__Rhizobiaceae; g__Candidatus Liberibacter |
| ASV_648 | k__Bacteria; p__Actinobacteriota; c__Actinobacteria; o__Micrococcales; f__Microbacteriaceae; g__Microbacterium |
| ASV_692 | k__Bacteria; p__Proteobacteria; c__Alphaproteobacteria; o__Sphingomonadales; f__Sphingomonadaceae |
| ASV_755 | k__Bacteria; p__Proteobacteria; c__Alphaproteobacteria; o__Rhizobiales; f__Rhizobiaceae; g__Allorhizobium-Neorhizobium-Pararhizobium-Rhizobium |
| ASV_758 | k__Bacteria; p__Proteobacteria; c__Alphaproteobacteria; o__Reyranellales; f__Reyranellaceae; g__Reyranella |
| ASV_76 | k__Bacteria; p__Proteobacteria; c__Gammaproteobacteria; o__Burkholderiales; f__Comamonadaceae; g__Limnohabitans |
| ASV_79 | k__Bacteria; p__Proteobacteria; c__Gammaproteobacteria; o__Burkholderiales; f__Oxalobacteraceae; g__Janthinobacterium |
| ASV_80 | k__Bacteria; p__Proteobacteria; c__Gammaproteobacteria; o__Pseudomonadales; f__Pseudomonadaceae; g__Pseudomonas |
| ASV_81 | k__Bacteria; p__Patescibacteria; c__Saccharimonadia; o__Saccharimonadales |
| ASV_85 | k__Bacteria; p__Proteobacteria; c__Gammaproteobacteria; o__Burkholderiales; f__Oxalobacteraceae |
| ASV_879 | k__Bacteria; p__Actinobacteriota; c__Actinobacteria; o__Micrococcales; f__Micrococcaceae; g__Arthrobacter |
| ASV_90 | k__Bacteria; p__Proteobacteria; c__Gammaproteobacteria; o__Pseudomonadales; f__Pseudomonadaceae; g__Pseudomonas |
| ASV_92 | k__Bacteria; p__Proteobacteria; c__Gammaproteobacteria; o__Enterobacterales |
| ASV_957 | k__Bacteria; p__Myxococcota; c__Myxococcia; o__Myxococcales; f__Myxococcaceae; g__P3OB-42 |
| ASV_96 | k__Bacteria; p__Proteobacteria; c__Gammaproteobacteria; o__Pseudomonadales; f__Pseudomonadaceae; g__Pseudomonas |
| ASV_99 | k__Bacteria; p__Proteobacteria; c__Gammaproteobacteria; o__Enterobacterales; f__Yersiniaceae; g__Rahnella1 |
| ASV_1019 | k__Bacteria; p__Proteobacteria; c__Alphaproteobacteria; o__Caulobacterales; f__Caulobacteraceae |
| ASV_1140 | k__Bacteria; p__Proteobacteria |
| ASV_1193 | k__Bacteria; p__Proteobacteria; c__Gammaproteobacteria; o__Burkholderiales; f__Methylophilaceae; g__Methylotenera |
| ASV_1233 | k__Bacteria; p__Proteobacteria; c__Gammaproteobacteria; o__Burkholderiales; f__Comamonadaceae; g__Variovorax; s__Variovorax paradoxus |

| | |
|---|---|
| ASV_148 | k__Bacteria; p__Proteobacteria; c__Gammaproteobacteria; o__Pseudomonadales; f__Pseudomonadaceae; g__Pseudomonas |
| ASV_225 | k__Bacteria; p__Proteobacteria; c__Gammaproteobacteria; o__Pseudomonadales; f__Pseudomonadaceae; g__Pseudomonas |
| ASV_247 | k__Bacteria; p__Patescibacteria; c__Saccharimonadia; o__Saccharimonadales |
| ASV_254 | k__Bacteria; p__Bacteroidota; c__Bacteroidia; o__Flavobacteriales; f__Flavobacteriaceae; g__Flavobacterium |
| ASV_26 | k__Bacteria; p__Patescibacteria; c__Saccharimonadia; o__Saccharimonadales |
| ASV_414 | k__Bacteria; p__Chloroflexi; c__Chloroflexia; o__Chloroflexales; f__Roseiflexaceae |
| ASV_46 | k__Bacteria; p__Proteobacteria; c__Gammaproteobacteria; o__Burkholderiales; f__Oxalobacteraceae; g__Duganella |
| ASV_573 | k__Bacteria; p__Actinobacteriota; c__Actinobacteria; o__Frankiales; f__Nakamurellaceae; g__Nakamurella; s__Nakamurella panacisegetis |
| ASV_582 | k__Bacteria; p__Bacteroidota; c__Bacteroidia; o__Chitinophagales; f__Chitinophagaceae; g__Chitinophaga |
| ASV_682 | k__Bacteria; p__Proteobacteria; c__Gammaproteobacteria; o__Pseudomonadales; f__Pseudomonadaceae; g__Pseudomonas |
| ASV_712 | k__Bacteria; p__Actinobacteriota; c__Actinobacteria; o__Corynebacteriales; f__Mycobacteriaceae; g__Mycobacterium |
| ASV_776 | k__Bacteria; p__Proteobacteria; c__Alphaproteobacteria; o__Azospirillales; f__Inquilinaceae; g__Inquilinus |
| ASV_940 | k__Bacteria; p__Proteobacteria; c__Gammaproteobacteria; o__Burkholderiales; f__Oxalobacteraceae; g__Massilia |

## B

| | RF based on tissue | | | | | RF based on alpine Rosaceae plant | | | | | RF based on collection site | | | | RF based on exposure | | |
|---|---|---|---|---|---|---|---|---|---|---|---|---|---|---|---|---|---|
| **ASV** | **Flowers** | **Leaves** | **Roots** | **MDA** | **ASV** | **Alchemilla** | **Dryas** | **Geum** | **MDA** | **ASV** | **Val_di_Fassa** | **Val_Rendena** | **MDA** | **ASV** | **North** | **South** | **MDA** |
| ASV_1 | 0.0099 | 0.0099 | 0.0099 | 0.0099 | ASV_103 | 0.0099 | 0.0099 | 0.0099 | 0.009901 | ASV_1025 | 0.0099 | 0.0099 | 0.0099 | ASV_113 | 0.0099 | 0.0198 | 0.009901 |
| ASV_10 | 0.0099 | 0.0099 | 0.0099 | 0.0099 | ASV_109 | 0.0099 | 0.0099 | 0.0099 | 0.009901 | ASV_1048 | 0.1188 | 0.0099 | 0.0099 | ASV_212 | 0.0198 | 0.0099 | 0.009901 |
| ASV_102 | 0.0099 | 0.0099 | 0.0198 | 0.0099 | ASV_1123 | 0.0099 | 0.0099 | 0.0396 | 0.009901 | ASV_11 | 0.0099 | 0.0099 | 0.0099 | ASV_238 | 0.0099 | 0.4158 | 0.009901 |
| ASV_109 | 0.0099 | 0.0396 | 0.0099 | 0.0099 | ASV_113 | 0.0099 | 0.0099 | 0.0693 | 0.009901 | ASV_118 | 0.0594 | 0.0099 | 0.0099 | ASV_306 | 0.0198 | 0.0099 | 0.009901 |
| ASV_1138 | 0.0297 | 0.4059 | 0.0693 | 0.0099 | ASV_1157 | 0.6733 | 0.0099 | 0.198 | 0.009901 | ASV_12 | 0.0099 | 0.0099 | 0.0099 | ASV_363 | 0.0099 | 0.4158 | 0.009901 |
| ASV_115 | 0.0099 | 0.396 | 0.0198 | 0.0099 | ASV_120 | 0.0099 | 0.0099 | 0.0099 | 0.009901 | ASV_152 | 0.0099 | 0.0099 | 0.0099 | ASV_438 | 0.0099 | 0.0099 | 0.009901 |
| ASV_116 | 0.0099 | 0.0099 | 0.0099 | 0.0099 | ASV_145 | 0.0099 | 0.0099 | 0.0099 | 0.009901 | ASV_186 | 0.0099 | 0.0198 | 0.0099 | ASV_5 | 0.0297 | 0.0396 | 0.009901 |
| ASV_130 | 0.0099 | 0.0099 | 0.0099 | 0.0099 | ASV_149 | 0.0099 | 0.0099 | 0.0099 | 0.009901 | ASV_196 | 0.0099 | 0.0396 | 0.0099 | ASV_565 | 0.0198 | 0.0099 | 0.009901 |
| ASV_14 | 0.0099 | 0.0099 | 0.0099 | 0.0099 | ASV_154 | 0.0099 | 0.0099 | 0.0099 | 0.009901 | ASV_197 | 0.0297 | 0.0099 | 0.0099 | ASV_617 | 0.0099 | 0.0198 | 0.009901 |
| ASV_1635 | 0.0099 | 0.0297 | 0.0297 | 0.0099 | ASV_16 | 0.0099 | 0.0495 | 0.0693 | 0.009901 | ASV_2 | 0.0099 | 0.0099 | 0.0099 | ASV_74 | 0.0891 | 0.0891 | 0.009901 |
| ASV_174 | 0.0099 | 0.6436 | 0.0693 | 0.0099 | ASV_163 | 0.0099 | 0.0099 | 0.0099 | 0.009901 | ASV_20 | 0.0099 | 0.0099 | 0.0099 | ASV_841 | 0.0198 | 0.0099 | 0.009901 |
| ASV_18 | 0.0099 | 0.0099 | 0.0099 | 0.0099 | ASV_177 | 0.0099 | 0.0099 | 0.0099 | 0.009901 | ASV_2109 | 0.0099 | 0.0099 | 0.0099 | ASV_1276 | 0.0396 | 0.0198 | 0.019802 |
| ASV_184 | 0.0099 | 0.0099 | 0.0099 | 0.0099 | ASV_179 | 0.0099 | 0.0099 | 0.0099 | 0.009901 | ASV_2169 | 0.0198 | 0.0198 | 0.0099 | ASV_255 | 0.0297 | 0.0396 | 0.019802 |
| ASV_189 | 0.0198 | 0.0099 | 0.0099 | 0.0099 | ASV_1814 | 0.0297 | 0.0396 | 0.0396 | 0.009901 | ASV_23 | 0.0396 | 0.0099 | 0.0099 | ASV_329 | 0.0198 | 0.3168 | 0.019802 |
| ASV_19 | 0.0099 | 0.0198 | 0.2574 | 0.0099 | ASV_19 | 0.0099 | 0.0099 | 0.1782 | 0.009901 | ASV_24 | 0.0396 | 0.0099 | 0.0099 | ASV_414 | 0.0099 | 0.0792 | 0.019802 |
| ASV_195 | 0.0099 | 0.0297 | 0.0396 | 0.0099 | ASV_193 | 0.0396 | 0.0198 | 0.5842 | 0.009901 | ASV_293 | 0.0099 | 0.0099 | 0.0099 | ASV_466 | 0.099 | 0.0099 | 0.019802 |
| ASV_196 | 0.0099 | 0.0198 | 0.0396 | 0.0099 | ASV_212 | 0.0099 | 0.0099 | 0.0099 | 0.009901 | ASV_347 | 0.0297 | 0.0198 | 0.0099 | ASV_476 | 0.0198 | 0.198 | 0.019802 |
| ASV_198 | 0.0198 | 0.0297 | 0.0099 | 0.0099 | ASV_220 | 0.0099 | 0.0099 | 0.0099 | 0.009901 | ASV_36 | 0.0099 | 0.0099 | 0.0099 | ASV_1113 | 0.0297 | 0.2178 | 0.029703 |
| ASV_199 | 0.0099 | 0.0099 | 0.0099 | 0.0099 | ASV_235 | 0.1287 | 0.0099 | 0.0198 | 0.009901 | ASV_376 | 0.0198 | 0.0099 | 0.0099 | ASV_1153 | 0.0297 | 0.0693 | 0.029703 |
| ASV_21 | 0.0099 | 0.3366 | 0.3069 | 0.0099 | ASV_236 | 0.0198 | 0.0099 | 0.0099 | 0.009901 | ASV_42 | 0.0099 | 0.0099 | 0.0099 | ASV_1284 | 0.0198 | 0.1287 | 0.029703 |
| ASV_211 | 0.0099 | 0.0099 | 0.0099 | 0.0099 | ASV_242 | 0.0198 | 0.0099 | 0.4059 | 0.009901 | ASV_423 | 0.0099 | 0.0099 | 0.0099 | ASV_1717 | 0.0297 | 0.1188 | 0.029703 |
| ASV_218 | 0.0099 | 0.0198 | 0.0099 | 0.0099 | ASV_244 | 0.0099 | 0.0099 | 0.0099 | 0.009901 | ASV_440 | 0.0099 | 0.0099 | 0.0099 | ASV_21 | 0.0792 | 0.1188 | 0.029703 |
| ASV_229 | 0.0099 | 0.0099 | 0.0099 | 0.0099 | ASV_252 | 0.0099 | 0.0495 | 0.1089 | 0.009901 | ASV_479 | 0.0198 | 0.0099 | 0.0099 | ASV_369 | 0.1584 | 0.0198 | 0.029703 |
| ASV_238 | 0.0099 | 0.0099 | 0.0099 | 0.0099 | ASV_263 | 0.0099 | 0.0099 | 0.0099 | 0.009901 | ASV_523 | 0.0495 | 0.0396 | 0.0099 | ASV_395 | 0.0099 | 0.0594 | 0.029703 |
| ASV_257 | 0.0099 | 0.0099 | 0.0099 | 0.0099 | ASV_267 | 0.2277 | 0.0099 | 0.0495 | 0.009901 | ASV_557 | 0.0198 | 0.0297 | 0.0099 | ASV_432 | 0.0594 | 0.0297 | 0.029703 |
| ASV_26 | 0.0099 | 0.0594 | 0.0099 | 0.0099 | ASV_272 | 0.0099 | 0.0099 | 0.0099 | 0.009901 | ASV_561 | 0.0099 | 0.0099 | 0.0099 | ASV_54 | 0.0792 | 0.0198 | 0.029703 |

| | | | | | | | | | | | | | | | | | |
|---|---|---|---|---|---|---|---|---|---|---|---|---|---|---|---|---|---|
| ASV_27 | 0.0099 | 0.0099 | 0.0099 | 0.0099 | ASV_284 | 0.0297 | 0.0099 | 0.0099 | 0.009901 | ASV_58 | 0.0099 | 0.0099 | 0.0099 | ASV_882 | 0.0396 | 0.0792 | 0.029703 |
| ASV_275 | 0.0099 | 0.0198 | 0.0198 | 0.0099 | ASV_297 | 0.0099 | 0.0099 | 0.0099 | 0.009901 | ASV_59 | 0.0099 | 0.0099 | 0.0099 | ASV_1025 | 0.0693 | 0.1881 | 0.039604 |
| ASV_28 | 0.0198 | 0.0099 | 0.099 | 0.0099 | ASV_300 | 0.0198 | 0.0099 | 0.0099 | 0.009901 | ASV_6 | 0.0792 | 0.0099 | 0.0099 | ASV_1123 | 0.8317 | 0.0099 | 0.039604 |
| ASV_284 | 0.0198 | 0.0099 | 0.0099 | 0.0099 | ASV_315 | 0.1485 | 0.0099 | 0.0891 | 0.009901 | ASV_63 | 0.0198 | 0.0099 | 0.0099 | ASV_1784 | 0.0297 | 0.0396 | 0.039604 |
| ASV_3 | 0.0099 | 0.0099 | 0.0099 | 0.0099 | ASV_316 | 0.0198 | 0.0099 | 0.0099 | 0.009901 | ASV_8 | 0.0099 | 0.0198 | 0.0099 | ASV_22 | 0.0396 | 0.0891 | 0.039604 |
| ASV_312 | 0.0099 | 0.0297 | 0.3168 | 0.0099 | ASV_317 | 0.0099 | 0.0099 | 0.0099 | 0.009901 | ASV_90 | 0.0099 | 0.0099 | 0.0099 | ASV_235 | 0.0198 | 0.0891 | 0.039604 |
| ASV_320 | 0.0099 | 0.0099 | 0.1089 | 0.0099 | ASV_321 | 0.8614 | 0.0099 | 0.0198 | 0.009901 | ASV_96 | 0.0099 | 0.0099 | 0.0099 | ASV_269 | 0.3168 | 0.0198 | 0.039604 |
| ASV_331 | 0.0495 | 0.0099 | 0.0495 | 0.0099 | ASV_339 | 0.0099 | 0.0099 | 0.0099 | 0.009901 | ASV_107 | 0.0297 | 0.0198 | 0.0198 | ASV_271 | 0.1386 | 0.0495 | 0.039604 |
| ASV_34 | 0.297 | 0.0099 | 0.0099 | 0.0099 | ASV_341 | 0.0198 | 0.0198 | 0.0297 | 0.009901 | ASV_1153 | 0.0396 | 0.0297 | 0.0198 | ASV_367 | 0.7426 | 0.0297 | 0.039604 |
| ASV_363 | 0.0099 | 0.0297 | 0.0099 | 0.0099 | ASV_364 | 0.0198 | 0.0099 | 0.0099 | 0.009901 | ASV_119 | 0.0099 | 0.0297 | 0.0198 | ASV_666 | 0.0495 | 0.0495 | 0.039604 |
| ASV_367 | 0.0099 | 0.0099 | 0.0099 | 0.0099 | ASV_371 | 0.7129 | 0.0099 | 0.0198 | 0.009901 | ASV_130 | 0.0198 | 0.0594 | 0.0198 | ASV_713 | 0.0396 | 0.1089 | 0.039604 |
| ASV_38 | 0.0099 | 0.0099 | 0.0099 | 0.0099 | ASV_385 | 0.0099 | 0.0099 | 0.0099 | 0.009901 | ASV_132 | 0.0396 | 0.2079 | 0.0198 | ASV_77 | 0.4455 | 0.0198 | 0.039604 |
| ASV_394 | 0.0099 | 0.0495 | 0.0693 | 0.0099 | ASV_39 | 0.9703 | 0.0099 | 0.0198 | 0.009901 | ASV_150 | 0.0396 | 0.0099 | 0.0198 | ASV_79 | 0.2079 | 0.0198 | 0.039604 |
| ASV_4 | 0.0099 | 0.0099 | 0.0099 | 0.0099 | ASV_406 | 0.1089 | 0.0198 | 0.0891 | 0.009901 | ASV_16 | 0.0198 | 0.0198 | 0.0198 | ASV_88 | 0.0495 | 0.0891 | 0.039604 |
| ASV_404 | 0.0198 | 0.0396 | 0.0099 | 0.0099 | ASV_425 | 0.0099 | 0.0396 | 0.0198 | 0.009901 | ASV_1783 | 0.0594 | 0.0099 | 0.0198 | ASV_274 | 0.0297 | 0.3267 | 0.049505 |
| ASV_419 | 0.0099 | 0.0099 | 0.0099 | 0.0099 | ASV_451 | 0.0099 | 0.0198 | 0.0099 | 0.009901 | ASV_229 | 0.2079 | 0.0198 | 0.0198 | ASV_29 | 0.0297 | 0.3168 | 0.049505 |
| ASV_424 | 0.0099 | 0.0198 | 0.0891 | 0.0099 | ASV_47 | 0.0198 | 0.2574 | 0.0198 | 0.009901 | ASV_315 | 0.0198 | 0.0495 | 0.0198 | ASV_712 | 0.0891 | 0.0495 | 0.049505 |
| ASV_47 | 0.198 | 0.099 | 0.0099 | 0.0099 | ASV_472 | 0.0099 | 0.0099 | 0.0099 | 0.009901 | ASV_325 | 0.2574 | 0.0198 | 0.0198 | ASV_93 | 0.0792 | 0.2376 | 0.049505 |
| ASV_470 | 0.0099 | 0.0099 | 0.0099 | 0.0099 | ASV_51 | 0.0792 | 0.0198 | 0.0099 | 0.009901 | ASV_341 | 0.0198 | 0.0396 | 0.0198 | | | | |
| ASV_52 | 0.0099 | 0.0099 | 0.2277 | 0.0099 | ASV_537 | 0.0297 | 0.0099 | 0.0099 | 0.009901 | ASV_407 | 0.0594 | 0.0693 | 0.0198 | | | | |
| ASV_585 | 0.0099 | 0.0099 | 0.0099 | 0.0099 | ASV_55 | 0.1881 | 0.0099 | 0.0198 | 0.009901 | ASV_495 | 0.0396 | 0.0198 | 0.0198 | | | | |
| ASV_6 | 0.0099 | 0.0396 | 0.2871 | 0.0099 | ASV_56 | 0.0099 | 0.0099 | 0.0099 | 0.009901 | ASV_61 | 0.2277 | 0.0099 | 0.0198 | | | | |
| ASV_609 | 0.0099 | 0.0099 | 0.0099 | 0.0099 | ASV_561 | 0.0198 | 0.0495 | 0.1386 | 0.009901 | ASV_626 | 0.0099 | 0.0495 | 0.0198 | | | | |
| ASV_63 | 0.0099 | 0.0297 | 0.2871 | 0.0099 | ASV_60 | 0.1287 | 0.0099 | 0.0099 | 0.009901 | ASV_74 | 0.0396 | 0.0198 | 0.0198 | | | | |
| ASV_65 | 0.0099 | 0.0099 | 0.0099 | 0.0099 | ASV_612 | 0.0792 | 0.0198 | 0.0297 | 0.009901 | ASV_84 | 0.0099 | 0.2673 | 0.0198 | | | | |
| ASV_66 | 0.0099 | 0.0099 | 0.0099 | 0.0099 | ASV_64 | 0.0198 | 0.0297 | 0.0099 | 0.009901 | ASV_868 | 0.0198 | 0.0198 | 0.0198 | | | | |
| ASV_69 | 0.0099 | 0.0693 | 0.0099 | 0.0099 | ASV_78 | 0.0099 | 0.0099 | 0.0099 | 0.009901 | ASV_1 | 0.099 | 0.0198 | 0.0297 | | | | |
| ASV_7 | 0.0099 | 0.0495 | 0.4554 | 0.0099 | ASV_83 | 0.0099 | 0.0198 | 0.0099 | 0.009901 | ASV_1087 | 0.0297 | 0.099 | 0.0297 | | | | |
| ASV_71 | 0.0099 | 0.0099 | 0.0099 | 0.0099 | ASV_898 | 0.0099 | 0.0693 | 0.0198 | 0.009901 | ASV_136 | 0.0198 | 0.0297 | 0.0297 | | | | |
| ASV_75 | 0.0099 | 0.0099 | 0.0594 | 0.0099 | ASV_92 | 0.0891 | 0.0099 | 0.0099 | 0.009901 | ASV_137 | 0.0495 | 0.0891 | 0.0297 | | | | |
| ASV_815 | 0.0099 | 0.0099 | 0.1188 | 0.0099 | ASV_941 | 0.0198 | 0.0099 | 0.0198 | 0.009901 | ASV_1407 | 0.0099 | 0.2178 | 0.0297 | | | | |
| ASV_82 | 0.0099 | 0.0099 | 0.0099 | 0.0099 | ASV_95 | 0.0099 | 0.0792 | 0.0198 | 0.009901 | ASV_143 | 0.0693 | 0.0693 | 0.0297 | | | | |
| ASV_820 | 0.0099 | 0.0099 | 0.0099 | 0.0099 | ASV_98 | 0.0099 | 0.0099 | 0.0099 | 0.009901 | ASV_188 | 0.0396 | 0.099 | 0.0297 | | | | |
| ASV_84 | 0.0099 | 0.0297 | 0.0099 | 0.0099 | ASV_1110 | 0.0396 | 0.0099 | 0.1188 | 0.019802 | ASV_252 | 0.2475 | 0.0099 | 0.0297 | | | | |
| ASV_999 | 0.099 | 0.0198 | 0.1386 | 0.0099 | ASV_176 | 0.0495 | 0.0297 | 0.0099 | 0.019802 | ASV_29 | 0.6535 | 0.0099 | 0.0297 | | | | |
| ASV_103 | 0.0198 | 0.099 | 0.2673 | 0.0198 | ASV_181 | 0.2475 | 0.0198 | 0.0396 | 0.019802 | ASV_393 | 0.0297 | 0.0891 | 0.0297 | | | | |
| ASV_12 | 0.0099 | 0.1287 | 0.6139 | 0.0198 | ASV_2073 | 0.0891 | 0.0099 | 0.0198 | 0.019802 | ASV_55 | 0.1584 | 0.0495 | 0.0297 | | | | |
| ASV_1277 | 0.0297 | 0.0297 | 0.0495 | 0.0198 | ASV_224 | 0.7228 | 0.0099 | 0.0693 | 0.019802 | ASV_66 | 0.1188 | 0.0396 | 0.0297 | | | | |
| ASV_181 | 0.1089 | 0.0198 | 0.1782 | 0.0198 | ASV_232 | 0.1683 | 0.3069 | 0.0099 | 0.019802 | ASV_67 | 0.0396 | 0.1287 | 0.0297 | | | | |
| ASV_182 | 0.0198 | 0.0297 | 0.0099 | 0.0198 | ASV_24 | 0.0099 | 0.0495 | 0.4257 | 0.019802 | ASV_771 | 0.0198 | 0.0594 | 0.0297 | | | | |
| ASV_226 | 0.0396 | 0.0198 | 0.0198 | 0.0198 | ASV_258 | 0.0297 | 0.0198 | 0.5347 | 0.019802 | ASV_1071 | 0.0297 | 0.0396 | 0.0396 | | | | |
| ASV_274 | 0.0198 | 0.3762 | 0.0198 | 0.0198 | ASV_275 | 0.1683 | 0.0297 | 0.0594 | 0.019802 | ASV_139 | 0.0792 | 0.0495 | 0.0396 | | | | |
| ASV_290 | 0.0099 | 0.0693 | 0.0495 | 0.0198 | ASV_295 | 0.0396 | 0.0297 | 0.0198 | 0.019802 | ASV_1500 | 0.0396 | 0.1287 | 0.0396 | | | | |
| ASV_299 | 0.0099 | 0.0495 | 0.0297 | 0.0198 | ASV_30 | 0.0297 | 0.0396 | 0.3267 | 0.019802 | ASV_1545 | 0.0297 | 0.0396 | 0.0396 | | | | |
| ASV_305 | 0.0693 | 0.1584 | 0.0099 | 0.0198 | ASV_338 | 0.6931 | 0.0396 | 0.0198 | 0.019802 | ASV_1633 | 0.0396 | 0.099 | 0.0396 | | | | |
| ASV_316 | 0.0099 | 0.2376 | 0.0198 | 0.0198 | ASV_418 | 0.7426 | 0.0099 | 0.0396 | 0.019802 | ASV_199 | 0.0693 | 0.0396 | 0.0396 | | | | |

| | | | | | | | | | | | | | |
|---|---|---|---|---|---|---|---|---|---|---|---|---|---|
| ASV_41 | 0.0099 | 0.2277 | 0.0891 | 0.0198 | ASV_44 | 0.0396 | 0.0099 | 0.1287 | 0.019802 | ASV_2073 | 0.3366 | 0.0396 | 0.0396 |
| ASV_1384 | 0.0297 | 0.0198 | 0.5743 | 0.0297 | ASV_455 | 0.0099 | 0.0891 | 0.0396 | 0.019802 | ASV_286 | 0.0396 | 0.0495 | 0.0396 |
| ASV_139 | 0.1782 | 0.2871 | 0.0198 | 0.0297 | ASV_46 | 0.0891 | 0.0396 | 0.0693 | 0.019802 | ASV_346 | 0.0396 | 0.1386 | 0.0396 |
| ASV_140 | 0.0891 | 0.0396 | 0.0891 | 0.0297 | ASV_504 | 0.0693 | 0.0792 | 0.0099 | 0.019802 | ASV_477 | 0.0396 | 0.0297 | 0.0396 |
| ASV_148 | 0.0198 | 0.3465 | 0.1386 | 0.0297 | ASV_536 | 0.4455 | 0.0099 | 0.604 | 0.019802 | ASV_525 | 0.0396 | 0.0297 | 0.0396 |
| ASV_149 | 0.0198 | 0.4158 | 0.1188 | 0.0297 | ASV_67 | 0.2277 | 0.0297 | 0.0396 | 0.019802 | ASV_536 | 0.0396 | 0.0396 | 0.0396 |
| ASV_170 | 0.0099 | 0.2475 | 0.2673 | 0.0297 | ASV_771 | 0.0297 | 0.4752 | 0.0198 | 0.019802 | ASV_636 | 0.0594 | 0.1683 | 0.0396 |
| ASV_183 | 0.0198 | 0.198 | 0.0396 | 0.0297 | ASV_79 | 0.0594 | 0.0099 | 0.7822 | 0.019802 | ASV_723 | 0.0594 | 0.1683 | 0.0396 |
| ASV_208 | 0.0297 | 0.198 | 0.0396 | 0.0297 | ASV_87 | 0.1188 | 0.0396 | 0.0297 | 0.019802 | ASV_75 | 0.0198 | 0.0693 | 0.0396 |
| ASV_234 | 0.0198 | 0.2871 | 0.1485 | 0.0297 | ASV_123 | 0.0594 | 0.0396 | 0.0495 | 0.029703 | ASV_819 | 0.099 | 0.0396 | 0.0396 |
| ASV_265 | 0.0198 | 0.4554 | 0.099 | 0.0297 | ASV_13 | 0.0396 | 0.0297 | 0.8713 | 0.029703 | ASV_1120 | 0.0396 | 0.1287 | 0.0495 |
| ASV_313 | 0.0198 | 0.0495 | 0.1584 | 0.0297 | ASV_1407 | 0.0297 | 0.6238 | 0.2772 | 0.029703 | ASV_123 | 0.0693 | 0.1584 | 0.0495 |
| ASV_321 | 0.0099 | 0.0693 | 0.099 | 0.0297 | ASV_206 | 0.0792 | 0.099 | 0.0099 | 0.029703 | ASV_1574 | 0.0198 | 0.1188 | 0.0495 |
| ASV_337 | 0.0297 | 0.2871 | 0.1881 | 0.0297 | ASV_342 | 0.2871 | 0.0297 | 0.0198 | 0.029703 | ASV_301 | 0.0297 | 0.1881 | 0.0495 |
| ASV_352 | 0.0396 | 0.1485 | 0.0792 | 0.0297 | ASV_440 | 0.0198 | 0.0594 | 0.0792 | 0.029703 | ASV_32 | 0.0594 | 0.1584 | 0.0495 |
| ASV_383 | 0.2574 | 0.0099 | 0.0792 | 0.0297 | ASV_461 | 0.3663 | 0.0099 | 0.5347 | 0.029703 | ASV_360 | 0.0198 | 0.3861 | 0.0495 |
| ASV_418 | 0.0495 | 0.3168 | 0.0396 | 0.0297 | ASV_622 | 0.3069 | 0.0693 | 0.0099 | 0.029703 | ASV_60 | 0.0594 | 0.0792 | 0.0495 |
| ASV_491 | 0.0099 | 0.8812 | 0.2178 | 0.0297 | ASV_957 | 0.2772 | 0.0099 | 0.1584 | 0.029703 | ASV_627 | 0.0396 | 0.1881 | 0.0495 |
| ASV_504 | 0.5842 | 0.0297 | 0.0297 | 0.0297 | ASV_1209 | 0.0891 | 0.0792 | 0.0396 | 0.039604 | ASV_648 | 0.0297 | 0.7723 | 0.0495 |
| ASV_61 | 0.0198 | 0.3663 | 0.4158 | 0.0297 | ASV_142 | 0.396 | 0.0693 | 0.099 | 0.039604 | ASV_790 | 0.3168 | 0.0198 | 0.0495 |
| ASV_612 | 0.0198 | 0.2574 | 0.2277 | 0.0297 | ASV_168 | 0.1188 | 0.0198 | 0.8515 | 0.039604 | ASV_812 | 0.0495 | 0.0594 | 0.0495 |
| ASV_622 | 0.0198 | 0.0396 | 0.0297 | 0.0297 | ASV_1783 | 0.4653 | 0.0198 | 0.0297 | 0.039604 | ASV_95 | 0.198 | 0.0099 | 0.0495 |
| ASV_648 | 0.1584 | 0.0297 | 0.5941 | 0.0297 | ASV_200 | 0.2079 | 0.0792 | 0.0099 | 0.039604 | | | | |
| ASV_682 | 0.1287 | 0.4752 | 0.0099 | 0.0297 | ASV_301 | 0.2376 | 0.0198 | 0.5743 | 0.039604 | | | | |
| ASV_107 | 0.099 | 0.4059 | 0.0099 | 0.0396 | ASV_320 | 0.2475 | 0.0792 | 0.1188 | 0.039604 | | | | |
| ASV_135 | 0.0891 | 0.1188 | 0.1287 | 0.0396 | ASV_324 | 0.1782 | 0.2277 | 0.0396 | 0.039604 | | | | |
| ASV_144 | 0.0099 | 0.6535 | 0.3762 | 0.0396 | ASV_356 | 0.4554 | 0.0396 | 0.0396 | 0.039604 | | | | |
| ASV_213 | 0.0693 | 0.1881 | 0.1485 | 0.0396 | ASV_405 | 0.8119 | 0.1188 | 0.0297 | 0.039604 | | | | |
| ASV_23 | 0.0297 | 0.2475 | 0.2871 | 0.0396 | ASV_454 | 0.495 | 0.0198 | 0.1287 | 0.039604 | | | | |
| ASV_255 | 0.1782 | 0.0792 | 0.0891 | 0.0396 | ASV_495 | 0.0099 | 0.1386 | 0.0594 | 0.039604 | | | | |
| ASV_285 | 0.0198 | 0.1485 | 0.5842 | 0.0396 | ASV_699 | 0.0099 | 0.7129 | 0.0099 | 0.039604 | | | | |
| ASV_390 | 0.0396 | 0.2079 | 0.1881 | 0.0396 | ASV_308 | 0.1089 | 0.0891 | 0.2574 | 0.049505 | | | | |
| ASV_412 | 0.099 | 0.3366 | 0.0198 | 0.0396 | ASV_330 | 0.2871 | 0.0495 | 0.0297 | 0.049505 | | | | |
| ASV_50 | 0.0297 | 0.0396 | 0.198 | 0.0396 | ASV_35 | 0.3465 | 0.0198 | 0.5545 | 0.049505 | | | | |
| ASV_627 | 0.0099 | 0.6931 | 0.3564 | 0.0396 | ASV_49 | 0.1881 | 0.0792 | 0.1782 | 0.049505 | | | | |
| ASV_657 | 0.0792 | 0.0693 | 0.2475 | 0.0396 | ASV_630 | 0.2673 | 0.4554 | 0.0198 | 0.049505 | | | | |
| ASV_110 | 0.0198 | 0.505 | 0.2376 | 0.0495 | ASV_736 | 0.1584 | 0.1683 | 0.2079 | 0.049505 | | | | |
| ASV_126 | 0.0396 | 0.2574 | 0.5743 | 0.0495 | ASV_99 | 0.1683 | 0.0099 | 0.6931 | 0.049505 | | | | |
| ASV_1500 | 0.5149 | 0.0198 | 0.4455 | 0.0495 | | | | | | | | | |
| ASV_309 | 0.0297 | 0.4455 | 0.505 | 0.0495 | | | | | | | | | |
| ASV_31 | 0.0297 | 0.3366 | 0.2079 | 0.0495 | | | | | | | | | |
| ASV_347 | 0.1188 | 0.0396 | 0.0891 | 0.0495 | | | | | | | | | |
| ASV_371 | 0.0495 | 0.495 | 0.1089 | 0.0495 | | | | | | | | | |
| ASV_520 | 0.0594 | 0.1881 | 0.099 | 0.0495 | | | | | | | | | |
| ASV_56 | 0.0396 | 0.0891 | 0.5644 | 0.0495 | | | | | | | | | |
| ASV_59 | 0.0297 | 0.4653 | 0.2871 | 0.0495 | | | | | | | | | |

| | | | | |
|---|---|---|---|---|
| ASV_616 | 0.0297 | 0.1584 | 0.1386 | 0.0495 |
| ASV_74 | 0.0099 | 0.3069 | 0.1881 | 0.0495 |
| ASV_98 | 0.0297 | 0.1485 | 0.2376 | 0.0495 |

| | Selected ASVs based on all factors |
|---|---|
| **ASV** | **Taxonomy** |
| ASV_103 | k__Bacteria; p__Proteobacteria; c__Gammaproteobacteria; o__Burkholderiales; f__Alcaligenaceae; g__Ampullimonas |
| ASV_109 | k__Bacteria; p__Proteobacteria; c__Alphaproteobacteria; o__Sphingomonadales; f__Sphingomonadaceae |
| ASV_1110 | k__Bacteria; p__Proteobacteria; c__Alphaproteobacteria; o__Caulobacterales; f__Caulobacteraceae; g__Caulobacter |
| ASV_1123 | k__Bacteria; p__Proteobacteria; c__Gammaproteobacteria; o__Burkholderiales; f__Oxalobacteraceae |
| ASV_113 | k__Bacteria; p__Proteobacteria; c__Gammaproteobacteria; o__Burkholderiales; f__Oxalobacteraceae |
| ASV_1157 | k__Bacteria; p__Proteobacteria; c__Gammaproteobacteria; o__Enterobacterales |
| ASV_120 | k__Bacteria; p__Proteobacteria; c__Gammaproteobacteria; o__Burkholderiales; f__Comamonadaceae |
| ASV_1209 | k__Bacteria; p__Proteobacteria; c__Alphaproteobacteria; o__Rhizobiales; f__Rhizobiaceae; g__Aureimonas |
| ASV_123 | k__Bacteria; p__Bacteroidota; c__Bacteroidia; o__Flavobacteriales; f__Flavobacteriaceae; g__Flavobacterium |
| ASV_13 | k__Bacteria; p__Proteobacteria; c__Gammaproteobacteria; o__Burkholderiales; f__Oxalobacteraceae; g__Duganella |
| ASV_1407 | k__Bacteria; p__Actinobacteriota; c__Acidimicrobiia; o__Microtrichales; f__Ilumatobacteraceae; g__Ilumatobacter |
| ASV_142 | k__Bacteria; p__Proteobacteria; c__Gammaproteobacteria; o__Enterobacterales; f__Erwiniaceae; g__Erwinia |
| ASV_145 | k__Bacteria; p__Proteobacteria; c__Alphaproteobacteria |
| ASV_149 | k__Bacteria; p__Patescibacteria; c__Saccharimonadia; o__Saccharimonadales |
| ASV_154 | k__Bacteria; p__Actinobacteriota; c__Actinobacteria; o__Propionibacteriales; f__Nocardioidaceae; g__Aeromicrobium |
| ASV_16 | k__Bacteria; p__Proteobacteria; c__Gammaproteobacteria; o__Burkholderiales; f__Oxalobacteraceae; g__Duganella |
| ASV_163 | k__Bacteria; p__Proteobacteria |
| ASV_168 | k__Bacteria; p__Proteobacteria; c__Gammaproteobacteria; o__Burkholderiales; f__Oxalobacteraceae |
| ASV_176 | k__Bacteria; p__Actinobacteriota; c__Actinobacteria; o__Micrococcales; f__Microbacteriaceae |
| ASV_177 | k__Bacteria; p__Proteobacteria; c__Gammaproteobacteria; o__Pseudomonadales |
| ASV_1783 | k__Bacteria; p__Proteobacteria; c__Alphaproteobacteria; o__Sphingomonadales; f__Sphingomonadaceae |
| ASV_179 | k__Bacteria; p__Proteobacteria |
| ASV_181 | k__Bacteria; p__Actinobacteriota; c__Actinobacteria; o__Micrococcales; f__Microbacteriaceae |
| ASV_1814 | k__Bacteria; p__Proteobacteria; c__Gammaproteobacteria; o__Burkholderiales |
| ASV_19 | k__Bacteria; p__Proteobacteria; c__Alphaproteobacteria; o__Sphingomonadales; f__Sphingomonadaceae; g__Sphingomonas; s__Sphingomonas faeni |
| ASV_193 | k__Bacteria; p__Proteobacteria; c__Gammaproteobacteria; o__Burkholderiales; f__Oxalobacteraceae |
| ASV_200 | k__Bacteria; p__Actinobacteriota; c__Actinobacteria; o__Micrococcales; f__Microbacteriaceae; g__Subtercola |
| ASV_206 | k__Bacteria; p__Actinobacteriota; c__Actinobacteria; o__Micrococcales; f__Microbacteriaceae |
| ASV_2073 | k__Bacteria; p__Proteobacteria; c__Alphaproteobacteria; o__Caulobacterales; f__Caulobacteraceae; g__Caulobacter |
| ASV_212 | k__Bacteria; p__Proteobacteria; c__Gammaproteobacteria; o__Pseudomonadales |
| ASV_220 | k__Bacteria; p__Proteobacteria; c__Gammaproteobacteria; o__Burkholderiales; f__Oxalobacteraceae |
| ASV_224 | k__Bacteria; p__Bacteroidota; c__Bacteroidia; o__Sphingobacteriales; f__Sphingobacteriaceae; g__Pedobacter |
| ASV_232 | k__Bacteria; p__Actinobacteriota; c__Actinobacteria; o__Micrococcales; f__Microbacteriaceae; g__Subtercola; s__Subtercola boreus |
| ASV_235 | k__Bacteria; p__Proteobacteria; c__Alphaproteobacteria; o__Rhizobiales; f__Rhizobiaceae; g__Allorhizobium-Neorhizobium-Pararhizobium-Rhizobium |
| ASV_236 | k__Bacteria; p__Proteobacteria |
| ASV_24 | k__Bacteria; p__Proteobacteria; c__Gammaproteobacteria; o__Burkholderiales; f__Oxalobacteraceae; g__Duganella |
| ASV_242 | k__Bacteria; p__Proteobacteria; c__Gammaproteobacteria; o__Burkholderiales; f__Oxalobacteraceae |
| ASV_244 | k__Bacteria; p__Proteobacteria; c__Gammaproteobacteria; o__Pseudomonadales |
| ASV_252 | k__Bacteria; p__Proteobacteria; c__Gammaproteobacteria; o__Burkholderiales; f__Oxalobacteraceae; g__Duganella |
| ASV_258 | k__Bacteria; p__Bacteroidota; c__Bacteroidia; o__Sphingobacteriales; f__Sphingobacteriaceae; g__Mucilaginibacter; s__Mucilaginibacter polytrichastri |

| | |
|---|---|
| ASV_263 | k__Bacteria; p__Bacteroidota; c__Bacteroidia; o__Sphingobacteriales; f__Sphingobacteriaceae; g__Pedobacter; s__Pedobacter westerhofensis |
| ASV_267 | k__Bacteria; p__Proteobacteria; c__Gammaproteobacteria; o__Enterobacterales |
| ASV_272 | k__Bacteria; p__Proteobacteria; c__Alphaproteobacteria; o__Rhizobiales |
| ASV_275 | k__Bacteria; p__Proteobacteria; c__Alphaproteobacteria; o__Rhizobiales; f__Rhizobiaceae |
| ASV_284 | k__Bacteria; p__Actinobacteriota; c__Actinobacteria; o__Micrococcales; f__Microbacteriaceae; g__Herbiconiux; s__Herbiconiux solani |
| ASV_295 | k__Bacteria; p__Bacteroidota; c__Bacteroidia; o__Sphingobacteriales; f__Sphingobacteriaceae; g__Pedobacter |
| ASV_297 | k__Bacteria; p__Proteobacteria |
| ASV_30 | k__Bacteria; p__Proteobacteria; c__Gammaproteobacteria; o__Burkholderiales; f__Oxalobacteraceae; g__Duganella |
| ASV_300 | k__Bacteria; p__Patescibacteria; c__Saccharimonadia; o__Saccharimonadales |
| ASV_301 | k__Bacteria; p__Actinobacteriota; c__Actinobacteria; o__Frankiales; f__Nakamurellaceae; g__Nakamurella |
| ASV_308 | k__Bacteria; p__Firmicutes; c__Bacilli; o__Bacillales; f__Bacillaceae |
| ASV_315 | k__Bacteria; p__Bacteroidota; c__Bacteroidia; o__Sphingobacteriales; f__Sphingobacteriaceae; g__Pedobacter |
| ASV_316 | k__Bacteria; p__Proteobacteria; c__Alphaproteobacteria; o__Sphingomonadales; f__Sphingomonadaceae |
| ASV_317 | k__Bacteria; p__Proteobacteria |
| ASV_320 | k__Bacteria; p__Proteobacteria; c__Alphaproteobacteria; o__Rhizobiales; f__Rhizobiaceae; g__Aureimonas |
| ASV_321 | k__Bacteria; p__Proteobacteria; c__Gammaproteobacteria; o__Burkholderiales; f__Comamonadaceae; g__Leptothrix |
| ASV_324 | k__Bacteria; p__Bacteroidota; c__Bacteroidia; o__Sphingobacteriales; f__Sphingobacteriaceae; g__Pedobacter |
| ASV_330 | k__Bacteria; p__Proteobacteria; c__Alphaproteobacteria; o__Rhizobiales; f__Rhizobiaceae; g__Allorhizobium-Neorhizobium-Pararhizobium-Rhizobium |
| ASV_338 | k__Bacteria; p__Proteobacteria; c__Gammaproteobacteria |
| ASV_339 | k__Bacteria; p__Proteobacteria; c__Alphaproteobacteria |
| ASV_341 | k__Bacteria; p__Actinobacteriota; c__Actinobacteria; o__Kineosporiales; f__Kineosporiaceae; g__Kineosporia |
| ASV_342 | k__Bacteria; p__Proteobacteria; c__Alphaproteobacteria; o__Rhizobiales; f__Rhizobiaceae; g__Neorhizobium |
| ASV_35 | k__Bacteria; p__Proteobacteria; c__Alphaproteobacteria; o__Rhizobiales; f__Rhizobiaceae; g__Candidatus Liberibacter |
| ASV_356 | k__Bacteria; p__Proteobacteria; c__Alphaproteobacteria; o__Rhizobiales; f__Rhizobiaceae |
| ASV_364 | k__Bacteria; p__Proteobacteria; c__Gammaproteobacteria |
| ASV_371 | k__Bacteria; p__Proteobacteria; c__Gammaproteobacteria; o__Burkholderiales; f__Methylophilaceae |
| ASV_385 | k__Bacteria; p__Proteobacteria; c__Gammaproteobacteria; o__Pseudomonadales |
| ASV_39 | k__Bacteria; p__Proteobacteria; c__Gammaproteobacteria; o__Burkholderiales; f__Burkholderiaceae |
| ASV_405 | k__Bacteria; p__Proteobacteria; c__Alphaproteobacteria |
| ASV_406 | k__Bacteria; p__Proteobacteria; c__Alphaproteobacteria; o__Caulobacterales; f__Caulobacteraceae; g__Brevundimonas; s__Brevundimonas staleyi |
| ASV_418 | k__Bacteria; p__Proteobacteria; c__Alphaproteobacteria; o__Sphingomonadales; f__Sphingomonadaceae; g__Novosphingobium; s__Novosphingobium soli |
| ASV_425 | k__Bacteria; p__Proteobacteria; c__Gammaproteobacteria; o__Burkholderiales; f__Oxalobacteraceae |
| ASV_44 | k__Bacteria; p__Proteobacteria; c__Gammaproteobacteria; o__Pseudomonadales; f__Pseudomonadaceae; g__Pseudomonas |
| ASV_440 | k__Bacteria; p__Actinobacteriota; c__Actinobacteria; o__Corynebacteriales; f__Nocardiaceae; g__Rhodococcus |
| ASV_451 | k__Bacteria; p__Actinobacteriota; c__Actinobacteria |
| ASV_454 | k__Bacteria; p__Bacteroidota; c__Bacteroidia; o__Sphingobacteriales; f__Sphingobacteriaceae; g__Mucilaginibacter; s__Mucilaginibacter angelicae |
| ASV_455 | k__Bacteria; p__Proteobacteria; c__Gammaproteobacteria; o__Burkholderiales; f__Oxalobacteraceae |
| ASV_46 | k__Bacteria; p__Proteobacteria; c__Gammaproteobacteria; o__Burkholderiales; f__Oxalobacteraceae; g__Duganella |
| ASV_461 | k__Bacteria; p__Actinobacteriota; c__Actinobacteria; o__Frankiales; f__Nakamurellaceae; g__Nakamurella |
| ASV_47 | k__Bacteria; p__Proteobacteria; c__Gammaproteobacteria; o__Burkholderiales; f__Oxalobacteraceae |
| ASV_472 | k__Bacteria; p__Proteobacteria; c__Alphaproteobacteria |
| ASV_49 | k__Bacteria; p__Proteobacteria; c__Gammaproteobacteria; o__Pseudomonadales; f__Pseudomonadaceae; g__Pseudomonas |
| ASV_495 | k__Bacteria; p__Actinobacteriota; c__Actinobacteria |
| ASV_504 | k__Bacteria; p__Actinobacteriota; c__Thermoleophilia; o__Solirubrobacterales; f__67-14 |
| ASV_51 | k__Bacteria; p__Proteobacteria; c__Alphaproteobacteria; o__Rhizobiales; f__Rhizobiaceae; g__Allorhizobium-Neorhizobium-Pararhizobium-Rhizobium |
| ASV_536 | k__Bacteria; p__Actinobacteriota; c__Actinobacteria; o__Micrococcales; f__Microbacteriaceae; g__Curtobacterium |

| | |
|---|---|
| ASV_537 | k__Bacteria; p__Proteobacteria; c__Alphaproteobacteria |
| ASV_55 | k__Bacteria; p__Proteobacteria; c__Gammaproteobacteria; o__Enterobacterales |
| ASV_56 | k__Bacteria; p__Proteobacteria; c__Gammaproteobacteria; o__Enterobacterales |
| ASV_561 | k__Bacteria; p__Actinobacteriota; c__Actinobacteria; o__Micrococcales; f__Cellulomonadaceae; g__Cellulomonas; s__Cellulomonas denverensis |
| ASV_60 | k__Bacteria; p__Proteobacteria; c__Gammaproteobacteria; o__Enterobacterales; f__Erwiniaceae; g__Erwinia |
| ASV_612 | k__Bacteria; p__Proteobacteria; c__Alphaproteobacteria; o__Rhizobiales; f__Rhizobiaceae; g__Allorhizobium-Neorhizobium-Pararhizobium-Rhizobium |
| ASV_622 | k__Bacteria; p__Proteobacteria; c__Alphaproteobacteria; o__Rhizobiales; f__Beijerinckiaceae; g__Bosea |
| ASV_630 | k__Bacteria; p__Bacteroidota; c__Bacteroidia; o__Sphingobacteriales; f__Sphingobacteriaceae; g__Mucilaginibacter |
| ASV_64 | k__Bacteria; p__Proteobacteria; c__Gammaproteobacteria; o__Enterobacterales |
| ASV_67 | k__Bacteria; p__Proteobacteria; c__Gammaproteobacteria; o__Xanthomonadales; f__Rhodanobacteraceae; g__Luteibacter |
| ASV_699 | k__Bacteria; p__Proteobacteria; c__Alphaproteobacteria; o__Sphingomonadales; f__Sphingomonadaceae; g__Novosphingobium |
| ASV_736 | k__Bacteria; p__Actinobacteriota; c__Actinobacteria; o__Micrococcales; f__Microbacteriaceae |
| ASV_771 | k__Bacteria; p__Actinobacteriota; c__Actinobacteria; o__Kineosporiales; f__Kineosporiaceae; g__Kineosporia |
| ASV_78 | k__Bacteria; p__Proteobacteria; c__Gammaproteobacteria; o__Enterobacterales |
| ASV_79 | k__Bacteria; p__Proteobacteria; c__Gammaproteobacteria; o__Burkholderiales; f__Oxalobacteraceae; g__Janthinobacterium |
| ASV_83 | k__Bacteria; p__Proteobacteria; c__Gammaproteobacteria; o__Burkholderiales; f__Oxalobacteraceae |
| ASV_87 | k__Bacteria; p__Proteobacteria; c__Gammaproteobacteria; o__Enterobacterales |
| ASV_898 | k__Bacteria; p__Actinobacteriota; c__Actinobacteria; o__Micrococcales; f__Microbacteriaceae; g__Galbitalea |
| ASV_92 | k__Bacteria; p__Proteobacteria; c__Gammaproteobacteria; o__Enterobacterales |
| ASV_941 | k__Bacteria; p__Actinobacteriota; c__Thermoleophilia; o__Solirubrobacterales; f__Solirubrobacteraceae |
| ASV_95 | k__Bacteria; p__Proteobacteria; c__Gammaproteobacteria; o__Burkholderiales; f__Oxalobacteraceae |
| ASV_957 | k__Bacteria; p__Myxococcota; c__Myxococcia; o__Myxococcales; f__Myxococcaceae; g__P3OB-42 |
| ASV_98 | k__Bacteria; p__Proteobacteria; c__Gammaproteobacteria; o__Burkholderiales; f__Alcaligenaceae; g__Ampullimonas |
| ASV_99 | k__Bacteria; p__Proteobacteria; c__Gammaproteobacteria; o__Enterobacterales; f__Yersiniaceae; g__Rahnella1 |
| ASV_1 | k__Bacteria; p__Proteobacteria; c__Gammaproteobacteria; o__Enterobacterales; f__Enterobacteriaceae; g__Escherichia-Shigella |
| ASV_10 | k__Bacteria; p__Proteobacteria; c__Gammaproteobacteria; o__Enterobacterales; f__Enterobacteriaceae; g__Escherichia-Shigella |
| ASV_102 | k__Bacteria; p__Proteobacteria; c__Gammaproteobacteria; o__Pseudomonadales; f__Pseudomonadaceae; g__Pseudomonas |
| ASV_107 | k__Bacteria; p__Proteobacteria; c__Gammaproteobacteria; o__Pseudomonadales; f__Pseudomonadaceae; g__Pseudomonas |
| ASV_110 | k__Bacteria; p__Proteobacteria; c__Gammaproteobacteria; o__Burkholderiales; f__Oxalobacteraceae |
| ASV_1138 | k__Bacteria; p__Proteobacteria; c__Alphaproteobacteria; o__Sphingomonadales; f__Sphingomonadaceae; g__Sphingomonas |
| ASV_115 | k__Bacteria; p__Proteobacteria; c__Gammaproteobacteria; o__Burkholderiales; f__Comamonadaceae; g__Variovorax |
| ASV_116 | k__Bacteria; p__Actinobacteriota; c__Actinobacteria; o__Micrococcales; f__Microbacteriaceae; g__Lysinimonas |
| ASV_12 | k__Bacteria; p__Proteobacteria; c__Alphaproteobacteria; o__Rhizobiales; f__Rhizobiaceae; g__Allorhizobium-Neorhizobium-Pararhizobium-Rhizobium |
| ASV_126 | k__Bacteria; p__Bacteroidota; c__Bacteroidia; o__Sphingobacteriales; f__Sphingobacteriaceae; g__Pedobacter |
| ASV_1277 | k__Bacteria; p__Proteobacteria; c__Alphaproteobacteria; o__Rhizobiales; f__Beijerinckiaceae; g__Methylobacterium-Methylorubrum |
| ASV_130 | k__Bacteria; p__Actinobacteriota; c__Actinobacteria; o__Frankiales; f__Cryptosporangiaceae; g__Cryptosporangium; s__Cryptosporangium aurantiacum |
| ASV_135 | k__Bacteria; p__Actinobacteriota; c__Actinobacteria; o__Micrococcales; f__Microbacteriaceae; g__Frondihabitans |
| ASV_1384 | k__Bacteria; p__Proteobacteria; c__Alphaproteobacteria; o__Rhizobiales; f__Rhizobiaceae |
| ASV_139 | k__Bacteria; p__Proteobacteria; c__Gammaproteobacteria; o__Pseudomonadales; f__Pseudomonadaceae; g__Pseudomonas |
| ASV_14 | k__Bacteria; p__Proteobacteria; c__Gammaproteobacteria; o__Enterobacterales; f__Enterobacteriaceae |
| ASV_140 | k__Bacteria; p__Proteobacteria; c__Gammaproteobacteria; o__Burkholderiales; f__Oxalobacteraceae; g__Herbaspirillum |
| ASV_144 | k__Bacteria; p__Proteobacteria; c__Gammaproteobacteria; o__Xanthomonadales; f__Xanthomonadaceae; g__Pseudoxanthomonas |
| ASV_148 | k__Bacteria; p__Proteobacteria; c__Gammaproteobacteria; o__Pseudomonadales; f__Pseudomonadaceae; g__Pseudomonas |
| ASV_1500 | k__Bacteria; p__Actinobacteriota; c__Acidimicrobiia; o__Microtrichales; f__Ilumatobacteraceae; g__CL500-29 marine group |
| ASV_1635 | k__Bacteria; p__Proteobacteria; c__Alphaproteobacteria; o__Sphingomonadales; f__Sphingomonadaceae; g__Sphingomonas |
| ASV_170 | k__Bacteria; p__Proteobacteria; c__Alphaproteobacteria; o__Sphingomonadales; f__Sphingomonadaceae; g__Rhizorhapis |

| | |
|---|---|
| ASV_174 | k__Bacteria; p__Proteobacteria; c__Gammaproteobacteria; o__Burkholderiales; f__Methylophilaceae |
| ASV_18 | k__Bacteria; p__Proteobacteria; c__Gammaproteobacteria; o__Enterobacterales; f__Enterobacteriaceae; g__Escherichia-Shigella |
| ASV_182 | k__Bacteria; p__Actinobacteriota; c__Actinobacteria; o__Micrococcales; f__Microbacteriaceae; g__Lysinimonas; s__Lysinimonas soli |
| ASV_183 | k__Bacteria; p__Proteobacteria; c__Gammaproteobacteria; o__Pseudomonadales; f__Pseudomonadaceae; g__Pseudomonas |
| ASV_184 | k__Bacteria; p__Proteobacteria; c__Alphaproteobacteria; o__Rhizobiales; f__Xanthobacteraceae; g__Bradyrhizobium |
| ASV_189 | k__Bacteria; p__Proteobacteria; c__Alphaproteobacteria; o__Caulobacterales; f__Caulobacteraceae; g__Caulobacter |
| ASV_195 | k__Bacteria; p__Proteobacteria; c__Gammaproteobacteria; o__Pseudomonadales; f__Pseudomonadaceae; g__Pseudomonas |
| ASV_196 | k__Bacteria; p__Proteobacteria; c__Gammaproteobacteria; o__Pseudomonadales; f__Pseudomonadaceae; g__Pseudomonas |
| ASV_198 | k__Bacteria; p__Actinobacteriota; c__Actinobacteria; o__Frankiales; f__Cryptosporangiaceae; g__Cryptosporangium |
| ASV_199 | k__Bacteria; p__Proteobacteria; c__Alphaproteobacteria; o__Rhizobiales; f__Xanthobacteraceae; g__Rhodopseudomonas |
| ASV_208 | k__Bacteria; p__Proteobacteria; c__Alphaproteobacteria; o__Rhizobiales; f__Xanthobacteraceae; g__Tardiphaga |
| ASV_21 | k__Bacteria; p__Proteobacteria; c__Gammaproteobacteria; o__Pseudomonadales; f__Pseudomonadaceae; g__Pseudomonas |
| ASV_211 | k__Bacteria; p__Actinobacteriota; c__Actinobacteria; o__Micromonosporales; f__Micromonosporaceae; g__Actinoplanes |
| ASV_213 | k__Bacteria; p__Proteobacteria; c__Gammaproteobacteria; o__Pseudomonadales; f__Pseudomonadaceae; g__Pseudomonas |
| ASV_218 | k__Bacteria; p__Proteobacteria; c__Alphaproteobacteria; o__Rhizobiales; f__Xanthobacteraceae |
| ASV_226 | k__Bacteria; p__Actinobacteriota; c__Actinobacteria; o__Streptomycetales; f__Streptomycetaceae; g__Streptomyces |
| ASV_229 | k__Bacteria; p__Proteobacteria; c__Alphaproteobacteria; o__Rhizobiales; f__Xanthobacteraceae; g__Tardiphaga; s__Tardiphaga robiniae |
| ASV_23 | k__Bacteria; p__Proteobacteria; c__Gammaproteobacteria; o__Pseudomonadales; f__Pseudomonadaceae; g__Pseudomonas |
| ASV_234 | k__Bacteria; p__Proteobacteria; c__Alphaproteobacteria; o__Sphingomonadales; f__Sphingomonadaceae; g__Novosphingobium; s__Novosphingobium barchaimii |
| ASV_238 | k__Bacteria; p__Proteobacteria; c__Alphaproteobacteria; o__Sphingomonadales; f__Sphingomonadaceae; g__Sphingomonas |
| ASV_255 | k__Bacteria; p__Proteobacteria; c__Alphaproteobacteria; o__Sphingomonadales; f__Sphingomonadaceae |
| ASV_257 | k__Bacteria; p__Actinobacteriota; c__Actinobacteria; o__Pseudonocardiales; f__Pseudonocardiaceae; g__Pseudonocardia; s__Pseudonocardia xinjiangensis |
| ASV_26 | k__Bacteria; p__Patescibacteria; c__Saccharimonadia; o__Saccharimonadales |
| ASV_265 | k__Bacteria; p__Proteobacteria; c__Alphaproteobacteria; o__Sphingomonadales; f__Sphingomonadaceae; g__Novosphingobium |
| ASV_27 | k__Bacteria; p__Proteobacteria; c__Gammaproteobacteria; o__Burkholderiales; f__Oxalobacteraceae; g__Janthinobacterium; s__Janthinobacterium lividum |
| ASV_274 | k__Bacteria; p__Actinobacteriota; c__Actinobacteria; o__Micrococcales; f__Microbacteriaceae; g__Lysinimonas |
| ASV_28 | k__Bacteria; p__Proteobacteria; c__Gammaproteobacteria; o__Burkholderiales; f__Oxalobacteraceae; g__Janthinobacterium; s__Janthinobacterium lividum |
| ASV_285 | k__Bacteria; p__Actinobacteriota; c__Actinobacteria; o__Micrococcales; f__Microbacteriaceae |
| ASV_290 | k__Bacteria; p__Proteobacteria; c__Alphaproteobacteria; o__Rhizobiales; f__Rhizobiaceae |
| ASV_299 | k__Bacteria; p__Proteobacteria; c__Alphaproteobacteria; o__Rhizobiales; f__Rhizobiaceae |
| ASV_3 | k__Bacteria; p__Proteobacteria; c__Gammaproteobacteria; o__Burkholderiales; f__Oxalobacteraceae; g__Janthinobacterium; s__Janthinobacterium lividum |
| ASV_305 | k__Bacteria; p__Proteobacteria; c__Gammaproteobacteria; o__Burkholderiales; f__Burkholderiaceae; g__Burkholderia-Caballeronia-Paraburkholderia |
| ASV_309 | k__Bacteria; p__Proteobacteria; c__Gammaproteobacteria; o__Burkholderiales; f__Comamonadaceae; g__Variovorax; s__Variovorax paradoxus |
| ASV_31 | k__Bacteria; p__Proteobacteria; c__Gammaproteobacteria; o__Pseudomonadales; f__Pseudomonadaceae; g__Pseudomonas |
| ASV_312 | k__Bacteria; p__Actinobacteriota; c__Actinobacteria; o__Propionibacteriales; f__Propionibacteriaceae; g__Cutibacterium; s__Cutibacterium acnes |
| ASV_313 | k__Bacteria; p__Proteobacteria; c__Alphaproteobacteria; o__Rhizobiales; f__Rhizobiaceae; g__Allorhizobium-Neorhizobium-Pararhizobium-Rhizobium |
| ASV_331 | k__Bacteria; p__Actinobacteriota; c__Actinobacteria; o__Corynebacteriales; f__Mycobacteriaceae; g__Mycobacterium |
| ASV_337 | k__Bacteria; p__Actinobacteriota; c__Actinobacteria; o__Propionibacteriales; f__Nocardioidaceae; g__Aeromicrobium; s__Aeromicrobium ginsengisoli |
| ASV_34 | k__Bacteria; p__Proteobacteria; c__Gammaproteobacteria; o__Burkholderiales; f__Oxalobacteraceae; g__Massilia |
| ASV_347 | k__Bacteria; p__Proteobacteria; c__Gammaproteobacteria; o__Pseudomonadales; f__Pseudomonadaceae; g__Pseudomonas |
| ASV_352 | k__Bacteria; p__Proteobacteria; c__Gammaproteobacteria; o__Burkholderiales; f__Comamonadaceae; g__Rhizobacter |
| ASV_363 | k__Bacteria; p__Proteobacteria; c__Alphaproteobacteria; o__Caulobacterales; f__Caulobacteraceae; g__Asticcacaulis; s__Asticcacaulis benevestitus |
| ASV_367 | k__Bacteria; p__Proteobacteria; c__Alphaproteobacteria; o__Rhizobiales; f__Rhizobiaceae; g__Allorhizobium-Neorhizobium-Pararhizobium-Rhizobium |
| ASV_38 | k__Bacteria; p__Proteobacteria; c__Gammaproteobacteria; o__Enterobacterales; f__Enterobacteriaceae; g__Escherichia-Shigella |
| ASV_383 | k__Bacteria; p__Proteobacteria; c__Alphaproteobacteria; o__Caulobacterales; f__Caulobacteraceae; g__Caulobacter |
| ASV_390 | k__Bacteria; p__Bacteroidota; c__Bacteroidia; o__Chitinophagales; f__Chitinophagaceae; g__Chitinophaga |

| | |
|---|---|
| ASV_394 | k__Bacteria; p__Proteobacteria; c__Alphaproteobacteria; o__Rhizobiales; f__Rhizobiaceae; g__Aureimonas |
| ASV_4 | k__Bacteria; p__Proteobacteria; c__Gammaproteobacteria; o__Burkholderiales; f__Burkholderiaceae; g__Ralstonia |
| ASV_404 | k__Bacteria; p__Proteobacteria; c__Alphaproteobacteria; o__Rhizobiales; f__Rhizobiaceae; g__Allorhizobium-Neorhizobium-Pararhizobium-Rhizobium |
| ASV_41 | k__Bacteria; p__Proteobacteria; c__Gammaproteobacteria; o__Pseudomonadales; f__Pseudomonadaceae; g__Pseudomonas |
| ASV_412 | k__Bacteria; p__Proteobacteria; c__Gammaproteobacteria; o__Burkholderiales; f__Burkholderiaceae; g__Burkholderia-Caballeronia-Paraburkholderia |
| ASV_419 | k__Bacteria; p__Bacteroidota; c__Bacteroidia; o__Cytophagales; f__Microscillaceae |
| ASV_424 | k__Bacteria; p__Proteobacteria; c__Gammaproteobacteria; o__Burkholderiales; f__Neisseriaceae |
| ASV_470 | k__Bacteria; p__Proteobacteria; c__Alphaproteobacteria; o__Caulobacterales; f__Caulobacteraceae; g__Brevundimonas; s__Brevundimonas staleyi |
| ASV_491 | k__Bacteria; p__Proteobacteria; c__Alphaproteobacteria; o__Rhizobiales; f__Rhizobiaceae |
| ASV_50 | k__Bacteria; p__Proteobacteria; c__Gammaproteobacteria; o__Pseudomonadales; f__Pseudomonadaceae; g__Pseudomonas |
| ASV_52 | k__Bacteria; p__Proteobacteria; c__Gammaproteobacteria; o__Pseudomonadales; f__Pseudomonadaceae; g__Pseudomonas |
| ASV_520 | k__Bacteria; p__Actinobacteriota; c__Actinobacteria; o__Frankiales; f__Frankiaceae; g__Frankia |
| ASV_585 | k__Bacteria; p__Proteobacteria; c__Alphaproteobacteria; o__Rhizobiales; f__Xanthobacteraceae; g__Ancylobacter |
| ASV_59 | k__Bacteria; p__Proteobacteria; c__Gammaproteobacteria; o__Pseudomonadales; f__Pseudomonadaceae; g__Pseudomonas |
| ASV_6 | k__Bacteria; p__Proteobacteria; c__Gammaproteobacteria; o__Pseudomonadales; f__Pseudomonadaceae; g__Pseudomonas |
| ASV_609 | k__Bacteria; p__Proteobacteria; c__Alphaproteobacteria; o__Caulobacterales; f__Caulobacteraceae; g__Caulobacter |
| ASV_61 | k__Bacteria; p__Proteobacteria; c__Gammaproteobacteria; o__Pseudomonadales; f__Pseudomonadaceae; g__Pseudomonas |
| ASV_616 | k__Bacteria; p__Proteobacteria; c__Gammaproteobacteria; o__Burkholderiales; f__Comamonadaceae; g__Xylophilus |
| ASV_627 | k__Bacteria; p__Proteobacteria; c__Alphaproteobacteria; o__Rhizobiales |
| ASV_63 | k__Bacteria; p__Proteobacteria; c__Gammaproteobacteria; o__Pseudomonadales; f__Pseudomonadaceae; g__Pseudomonas |
| ASV_648 | k__Bacteria; p__Actinobacteriota; c__Actinobacteria; o__Micrococcales; f__Microbacteriaceae; g__Microbacterium |
| ASV_65 | k__Bacteria; p__Proteobacteria; c__Gammaproteobacteria; o__Burkholderiales; f__Burkholderiaceae; g__Burkholderia-Caballeronia-Paraburkholderia |
| ASV_657 | k__Bacteria; p__Bacteroidota; c__Bacteroidia; o__Sphingobacteriales; f__Sphingobacteriaceae; g__Mucilaginibacter |
| ASV_66 | k__Bacteria; p__Actinobacteriota; c__Actinobacteria; o__Kineosporiales; f__Kineosporiaceae; g__Kineosporia |
| ASV_682 | k__Bacteria; p__Proteobacteria; c__Gammaproteobacteria; o__Pseudomonadales; f__Pseudomonadaceae; g__Pseudomonas |
| ASV_69 | k__Bacteria; p__Actinobacteriota; c__Actinobacteria; o__Micrococcales; f__Microbacteriaceae; g__Galbitalea |
| ASV_7 | k__Bacteria; p__Proteobacteria; c__Gammaproteobacteria; o__Pseudomonadales; f__Pseudomonadaceae; g__Pseudomonas |
| ASV_71 | k__Bacteria; p__Proteobacteria; c__Gammaproteobacteria; o__Burkholderiales; f__Oxalobacteraceae |
| ASV_74 | k__Bacteria; p__Proteobacteria; c__Gammaproteobacteria; o__Burkholderiales; f__Oxalobacteraceae; g__Duganella |
| ASV_75 | k__Bacteria; p__Proteobacteria; c__Gammaproteobacteria; o__Enterobacterales |
| ASV_815 | k__Bacteria; p__Actinobacteriota; c__Actinobacteria; o__Kineosporiales; f__Kineosporiaceae; g__Kineococcus; s__Kineococcus radiotolerans |
| ASV_82 | k__Bacteria; p__Chloroflexi; c__Chloroflexia; o__Chloroflexales; f__Roseiflexaceae |
| ASV_820 | k__Bacteria; p__Actinobacteriota; c__Actinobacteria; o__Frankiales; f__Frankiaceae; g__Jatrophihabitans |
| ASV_84 | k__Bacteria; p__Actinobacteriota; c__Actinobacteria; o__Kineosporiales; f__Kineosporiaceae; g__Kineosporia |
| ASV_999 | k__Bacteria; p__Proteobacteria; c__Alphaproteobacteria; o__Rhizobiales; f__Rhizobiaceae; g__Allorhizobium-Neorhizobium-Pararhizobium-Rhizobium |
| ASV_1025 | k__Bacteria; p__Actinobacteriota; c__Actinobacteria; o__Micrococcales; f__Microbacteriaceae; g__Cryobacterium |
| ASV_1048 | k__Bacteria; p__Proteobacteria; c__Gammaproteobacteria |
| ASV_1071 | k__Bacteria; p__Proteobacteria; c__Gammaproteobacteria; o__Burkholderiales; f__Methylophilaceae |
| ASV_1087 | k__Bacteria; p__Chloroflexi; c__TK10 |
| ASV_11 | k__Bacteria; p__Proteobacteria; c__Gammaproteobacteria; o__Pseudomonadales; f__Pseudomonadaceae; g__Pseudomonas |
| ASV_1120 | k__Bacteria |
| ASV_1153 | k__Bacteria; p__Bacteroidota; c__Bacteroidia; o__Sphingobacteriales; f__Sphingobacteriaceae; g__Mucilaginibacter |
| ASV_118 | k__Bacteria; p__Proteobacteria; c__Gammaproteobacteria; o__Pseudomonadales; f__Pseudomonadaceae; g__Pseudomonas |
| ASV_119 | k__Bacteria; p__Actinobacteriota; c__Actinobacteria; o__Kineosporiales; f__Kineosporiaceae; g__Kineosporia; s__Kineosporia aurantiaca |
| ASV_132 | k__Bacteria; p__Proteobacteria; c__Gammaproteobacteria; o__Burkholderiales; f__Oxalobacteraceae; g__Duganella |
| ASV_136 | k__Bacteria; p__Proteobacteria; c__Gammaproteobacteria; o__Enterobacterales; f__Erwiniaceae |

| | |
|---|---|
| ASV_137 | k__Bacteria; p__Proteobacteria; c__Gammaproteobacteria; o__Burkholderiales; f__Oxalobacteraceae |
| ASV_143 | k__Bacteria; p__Proteobacteria; c__Gammaproteobacteria; o__Enterobacterales; f__Yersiniaceae; g__Rahnella1 |
| ASV_150 | k__Bacteria; p__Proteobacteria; c__Gammaproteobacteria |
| ASV_152 | k__Bacteria; p__Bacteroidota; c__Bacteroidia; o__Sphingobacteriales; f__Sphingobacteriaceae; g__Mucilaginibacter; s__Mucilaginibacter jinjuensis |
| ASV_1545 | k__Bacteria; p__Actinobacteriota; c__Actinobacteria; o__Micrococcales; f__Intrasporangiaceae; g__Lapillicoccus |
| ASV_1574 | k__Bacteria; p__Actinobacteriota; c__Thermoleophilia; o__Solirubrobacterales; f__67-14 |
| ASV_1633 | k__Bacteria; p__Proteobacteria; c__Gammaproteobacteria |
| ASV_186 | k__Bacteria; p__Proteobacteria; c__Gammaproteobacteria; o__Enterobacterales; f__Erwiniaceae; g__Pantoea |
| ASV_188 | k__Bacteria; p__Proteobacteria; c__Gammaproteobacteria; o__Pseudomonadales; f__Pseudomonadaceae; g__Pseudomonas; s__Pseudomonas abietaniphila |
| ASV_197 | k__Bacteria; p__Proteobacteria; c__Gammaproteobacteria; o__Pseudomonadales; f__Pseudomonadaceae; g__Pseudomonas |
| ASV_2 | k__Bacteria; p__Proteobacteria; c__Gammaproteobacteria; o__Pseudomonadales; f__Pseudomonadaceae; g__Pseudomonas |
| ASV_20 | k__Bacteria; p__Proteobacteria; c__Gammaproteobacteria; o__Pseudomonadales; f__Pseudomonadaceae; g__Pseudomonas; s__Pseudomonas syringae |
| ASV_2109 | k__Bacteria; p__Actinobacteriota; c__Actinobacteria; o__Frankiales; f__Cryptosporangiaceae; g__Cryptosporangium |
| ASV_2169 | k__Bacteria; p__Actinobacteriota; c__Actinobacteria; o__Micrococcales; f__Microbacteriaceae; g__Galbitalea |
| ASV_286 | k__Bacteria; p__Proteobacteria; c__Gammaproteobacteria; o__Enterobacterales |
| ASV_29 | k__Bacteria; p__Proteobacteria; c__Gammaproteobacteria; o__Pseudomonadales; f__Pseudomonadaceae; g__Pseudomonas |
| ASV_293 | k__Bacteria; p__Proteobacteria; c__Gammaproteobacteria; o__Pseudomonadales; f__Moraxellaceae |
| ASV_32 | k__Bacteria; p__Proteobacteria; c__Alphaproteobacteria; o__Rhizobiales; f__Rhizobiaceae; g__Allorhizobium-Neorhizobium-Pararhizobium-Rhizobium |
| ASV_325 | k__Bacteria; p__Proteobacteria; c__Alphaproteobacteria; o__Sphingomonadales; f__Sphingomonadaceae |
| ASV_346 | k__Bacteria; p__Proteobacteria; c__Gammaproteobacteria; o__Burkholderiales; f__Oxalobacteraceae |
| ASV_36 | k__Bacteria; p__Proteobacteria; c__Gammaproteobacteria; o__Pseudomonadales; f__Pseudomonadaceae; g__Pseudomonas; s__Pseudomonas syringae |
| ASV_360 | k__Bacteria; p__Proteobacteria; c__Alphaproteobacteria; o__Rhizobiales; f__Rhizobiaceae; g__Allorhizobium-Neorhizobium-Pararhizobium-Rhizobium |
| ASV_376 | k__Bacteria; p__Proteobacteria; c__Gammaproteobacteria; o__Pseudomonadales; f__Pseudomonadaceae; g__Pseudomonas |
| ASV_393 | k__Bacteria; p__Proteobacteria; c__Alphaproteobacteria; o__Rhizobiales; f__Rhizobiaceae; g__Allorhizobium-Neorhizobium-Pararhizobium-Rhizobium |
| ASV_407 | k__Bacteria; p__Proteobacteria; c__Gammaproteobacteria; o__Burkholderiales; f__Comamonadaceae; g__Rhizobacter |
| ASV_42 | k__Bacteria; p__Proteobacteria; c__Gammaproteobacteria; o__Pseudomonadales; f__Pseudomonadaceae; g__Pseudomonas |
| ASV_423 | k__Bacteria; p__Proteobacteria; c__Gammaproteobacteria; o__Pseudomonadales; f__Pseudomonadaceae; g__Pseudomonas |
| ASV_477 | k__Bacteria; p__Actinobacteriota; c__Actinobacteria; o__Micrococcales; f__Microbacteriaceae; g__Microbacterium |
| ASV_479 | k__Bacteria; p__Proteobacteria; c__Gammaproteobacteria |
| ASV_523 | k__Bacteria; p__Proteobacteria; c__Gammaproteobacteria; o__Enterobacterales; f__Erwiniaceae; g__Pantoea |
| ASV_525 | k__Bacteria; p__Actinobacteriota; c__Actinobacteria; o__Pseudonocardiales; f__Pseudonocardiaceae; g__Pseudonocardia |
| ASV_557 | k__Bacteria; p__Proteobacteria; c__Gammaproteobacteria; o__Burkholderiales; f__Oxalobacteraceae; g__Massilia; s__Massilia eurypsychrophila |
| ASV_58 | k__Bacteria; p__Proteobacteria; c__Gammaproteobacteria; o__Pseudomonadales; f__Pseudomonadaceae; g__Pseudomonas |
| ASV_626 | k__Bacteria; p__Actinobacteriota; c__Actinobacteria; o__Micrococcales; f__Microbacteriaceae; g__Galbitalea |
| ASV_636 | k__Bacteria; p__Proteobacteria; c__Gammaproteobacteria; o__Burkholderiales; f__Oxalobacteraceae |
| ASV_723 | k__Bacteria; p__Proteobacteria; c__Gammaproteobacteria; o__Burkholderiales; f__Alcaligenaceae; g__Verticiella |
| ASV_790 | k__Bacteria; p__Proteobacteria; c__Gammaproteobacteria; o__Pseudomonadales; f__Pseudomonadaceae; g__Pseudomonas |
| ASV_8 | k__Bacteria; p__Proteobacteria; c__Gammaproteobacteria; o__Pseudomonadales; f__Pseudomonadaceae; g__Pseudomonas |
| ASV_812 | k__Bacteria; p__Firmicutes; c__Bacilli; o__Erysipelotrichales; f__Erysipelotrichaceae; g__Turicibacter |
| ASV_819 | k__Bacteria; p__Bacteroidota; c__Bacteroidia; o__Flavobacteriales; f__Flavobacteriaceae; g__Flavobacterium; s__Flavobacterium subsaxonicum |
| ASV_868 | k__Bacteria; p__Proteobacteria; c__Alphaproteobacteria; o__Rhizobiales; f__Rhizobiaceae; g__Allorhizobium-Neorhizobium-Pararhizobium-Rhizobium |
| ASV_90 | k__Bacteria; p__Proteobacteria; c__Gammaproteobacteria; o__Pseudomonadales; f__Pseudomonadaceae; g__Pseudomonas |
| ASV_96 | k__Bacteria; p__Proteobacteria; c__Gammaproteobacteria; o__Pseudomonadales; f__Pseudomonadaceae; g__Pseudomonas |
| ASV_1113 | k__Bacteria; p__Chloroflexi; c__Chloroflexia; o__Chloroflexales; f__Roseiflexaceae |
| ASV_1276 | k__Bacteria; p__Proteobacteria; c__Gammaproteobacteria; o__Burkholderiales; f__Oxalobacteraceae |
| ASV_1284 | k__Bacteria; p__Proteobacteria; c__Gammaproteobacteria; o__Burkholderiales; f__Oxalobacteraceae |

| | |
|---|---|
| ASV_1717 | k__Bacteria; p__Proteobacteria; c__Alphaproteobacteria; o__Rhizobiales; f__Rhizobiaceae; g__Allorhizobium-Neorhizobium-Pararhizobium-Rhizobium |
| ASV_1784 | k__Bacteria; p__Proteobacteria; c__Gammaproteobacteria; o__Pseudomonadales; f__Moraxellaceae; g__Acinetobacter |
| ASV_22 | k__Bacteria; p__Proteobacteria; c__Gammaproteobacteria; o__Pseudomonadales; f__Pseudomonadaceae; g__Pseudomonas |
| ASV_269 | k__Bacteria; p__Proteobacteria; c__Gammaproteobacteria; o__Burkholderiales; f__Oxalobacteraceae |
| ASV_271 | k__Bacteria; p__Proteobacteria; c__Alphaproteobacteria; o__Sphingomonadales; f__Sphingomonadaceae; g__Rhizorhapis |
| ASV_306 | k__Bacteria; p__Proteobacteria; c__Gammaproteobacteria; o__Burkholderiales; f__Oxalobacteraceae; g__Janthinobacterium; s__Janthinobacterium lividum |
| ASV_329 | k__Bacteria; p__Proteobacteria; c__Gammaproteobacteria; o__Burkholderiales; f__Oxalobacteraceae |
| ASV_369 | k__Bacteria; p__Chloroflexi; c__Chloroflexia; o__Chloroflexales; f__Chloroflexaceae; g__FFCH7168 |
| ASV_395 | k__Bacteria; p__Proteobacteria; c__Gammaproteobacteria; o__Burkholderiales; f__Oxalobacteraceae; g__Janthinobacterium |
| ASV_414 | k__Bacteria; p__Chloroflexi; c__Chloroflexia; o__Chloroflexales; f__Roseiflexaceae |
| ASV_432 | k__Bacteria; p__Proteobacteria; c__Gammaproteobacteria; o__Burkholderiales; f__Oxalobacteraceae |
| ASV_438 | k__Bacteria; p__Actinobacteriota; c__Actinobacteria; o__Kineosporiales; f__Kineosporiaceae; g__Kineosporia |
| ASV_466 | k__Bacteria; p__Proteobacteria; c__Alphaproteobacteria; o__Sphingomonadales; f__Sphingomonadaceae |
| ASV_476 | k__Bacteria; p__Proteobacteria; c__Gammaproteobacteria; o__Burkholderiales; f__Comamonadaceae; g__Xylophilus |
| ASV_5 | k__Bacteria; p__Proteobacteria; c__Gammaproteobacteria; o__Pseudomonadales; f__Pseudomonadaceae; g__Pseudomonas |
| ASV_54 | k__Bacteria; p__Proteobacteria; c__Gammaproteobacteria; o__Burkholderiales; f__Oxalobacteraceae |
| ASV_565 | k__Bacteria; p__Proteobacteria; c__Alphaproteobacteria; o__Rhizobiales; f__Rhizobiaceae; g__Allorhizobium-Neorhizobium-Pararhizobium-Rhizobium |
| ASV_617 | k__Bacteria; p__Bacteroidota; c__Bacteroidia; o__Sphingobacteriales; f__Sphingobacteriaceae; g__Mucilaginibacter |
| ASV_666 | k__Bacteria; p__Bacteroidota; c__Bacteroidia; o__Flavobacteriales; f__Flavobacteriaceae; g__Flavobacterium |
| ASV_712 | k__Bacteria; p__Actinobacteriota; c__Actinobacteria; o__Corynebacteriales; f__Mycobacteriaceae; g__Mycobacterium |
| ASV_713 | k__Bacteria; p__Proteobacteria; c__Alphaproteobacteria; o__Sphingomonadales; f__Sphingomonadaceae; g__Polymorphobacter |
| ASV_77 | k__Bacteria; p__Actinobacteriota; c__Actinobacteria; o__Frankiales; f__Cryptosporangiaceae; g__Cryptosporangium; s__Cryptosporangium mongoliense |
| ASV_841 | k__Bacteria; p__Actinobacteriota; c__Actinobacteria; o__Micrococcales; f__Microbacteriaceae; g__Amnibacterium |
| ASV_88 | k__Bacteria; p__Proteobacteria; c__Alphaproteobacteria; o__Sphingomonadales; f__Sphingomonadaceae; g__Novosphingobium |
| ASV_882 | k__Bacteria; p__Bacteroidota; c__Bacteroidia; o__Flavobacteriales; f__Flavobacteriaceae; g__Flavobacterium |
| ASV_93 | k__Bacteria; p__Bacteroidota; c__Bacteroidia; o__Flavobacteriales; f__Flavobacteriaceae; g__Flavobacterium |

**Table S12.** Significantly differentially abundant amplicon sequence variants (ASV) from the first dataset (**A**; *Alchemilla* sp. and *G. montanum* from six collection sites) and second dataset (**B**; *Alchemilla* sp., *D. octopetala,* and *G. montanum* from two collection sites) of samples from alpine Rosaceae plants. Log fold change (lfc), standard errors (se) of lfc, W test statistics (W = lfc/se), p-values (obtained from two-sided Z-test using the W test statistic), q values (adjusted p-values obtained by applying Benjamini-Hochberg method) are reported for each ASV in the flower (F) and leaf (L) tissues compared with the root (R) tissue. Columns starts with Tissue_diff_abund represent significant ASVs in the flower or leaf tissues compared with the root tissue. Abbreviations; Intercept (INT).

## A

| ASV | lfc_ INT | lfc_ L | lfc_ F | se_ INT | se_ L | se_ F | W_ INT | W_ L | W_ F | p_ INT | p_ L | p_ F | q_ INT | q_ L | q_ F | Tissue_ diff_abund | Family | Genus |
|---|---|---|---|---|---|---|---|---|---|---|---|---|---|---|---|---|---|---|
| ASV_1 | -0.86 | 3.98 | 0.44 | 0.59 | 0.24 | 0.26 | -1.47 | 16.56 | 1.71 | 0.14 | 0.00 | 0.09 | 0.53 | 0.00 | 0.14 | Leaves only | Enterobacteriaceae | Escherichia-Shigella |
| ASV_10 | -1.14 | 2.64 | 0.11 | 0.63 | 0.20 | 0.19 | -1.80 | 13.50 | 0.58 | 0.07 | 0.00 | 0.57 | 0.37 | 0.00 | 0.67 | Leaves only | Enterobacteriaceae | Escherichia-Shigella |
| ASV_100 | 0.11 | -0.46 | -1.21 | 0.75 | 0.21 | 0.21 | 0.15 | -2.23 | -5.81 | 0.88 | 0.03 | 0.00 | 1.00 | 0.05 | 0.00 | Flowers only | Flavobacteriaceae | Flavobacterium |
| ASV_102 | -1.21 | 0.07 | 2.13 | 0.67 | 0.23 | 0.25 | -1.80 | 0.30 | 8.60 | 0.07 | 0.77 | 0.00 | 0.37 | 0.89 | 0.00 | Flowers only | Pseudomonadaceae | Pseudomonas |
| ASV_1029 | -0.62 | 0.65 | 0.91 | 0.77 | 0.19 | 0.20 | -0.81 | 3.34 | 4.57 | 0.44 | 0.01 | 0.00 | 0.91 | 0.01 | 0.00 | Leaves and Flowers | Xanthomonadaceae | Stenotrophomonas |
| ASV_103 | NA | NA | NA | NA | NA | NA | NA | NA | NA | 1.00 | 1.00 | 1.00 | 1.00 | 1.00 | 1.00 | Not different | Alcaligenaceae | Ampullimonas |
| ASV_104 | -1.13 | 0.22 | 1.41 | 0.72 | 0.22 | 0.23 | -1.56 | 0.99 | 6.16 | 0.12 | 0.33 | 0.00 | 0.48 | 0.43 | 0.00 | Flowers only | Pseudomonadaceae | Pseudomonas |
| ASV_106 | -0.62 | 0.44 | 1.42 | 0.68 | 0.22 | 0.23 | -0.92 | 1.98 | 6.06 | 0.36 | 0.05 | 0.00 | 0.84 | 0.08 | 0.00 | Flowers only | Pseudomonadaceae | Pseudomonas |
| ASV_11 | -1.20 | -0.10 | 1.64 | 0.61 | 0.21 | 0.24 | -1.97 | -0.46 | 6.86 | 0.05 | 0.65 | 0.00 | 0.33 | 0.79 | 0.00 | Flowers only | Pseudomonadaceae | Pseudomonas |
| ASV_110 | 0.11 | 0.78 | 0.86 | 0.73 | 0.20 | 0.21 | 0.15 | 3.94 | 4.19 | 0.88 | 0.00 | 0.00 | 1.00 | 0.00 | 0.00 | Leaves and Flowers | Oxalobacteraceae | Unclassified |
| ASV_1110 | -0.07 | -0.61 | 0.58 | 0.76 | 0.19 | 0.20 | -0.10 | -3.18 | 2.98 | 0.92 | 0.00 | 0.01 | 1.00 | 0.01 | 0.01 | Leaves and Flowers | Caulobacteraceae | Caulobacter |
| ASV_113 | -1.14 | 0.59 | 1.15 | 0.71 | 0.19 | 0.21 | -1.60 | 3.05 | 5.62 | 0.12 | 0.00 | 0.00 | 0.47 | 0.01 | 0.00 | Leaves and Flowers | Oxalobacteraceae | Unclassified |
| ASV_114 | -0.69 | -0.30 | 1.51 | 0.70 | 0.19 | 0.23 | -0.99 | -1.53 | 6.53 | 0.33 | 0.13 | 0.00 | 0.80 | 0.20 | 0.00 | Flowers only | Pseudomonadaceae | Pseudomonas |
| ASV_1140 | -0.23 | -0.11 | -0.05 | 0.72 | 0.20 | 0.22 | -0.32 | -0.56 | -0.24 | 0.75 | 0.58 | 0.81 | 1.00 | 0.71 | 0.90 | Not different | Unclassified | Unclassified |
| ASV_115 | 0.30 | -0.54 | -0.45 | 0.71 | 0.22 | 0.21 | 0.42 | -2.45 | -2.12 | 0.68 | 0.02 | 0.04 | 1.00 | 0.03 | 0.06 | Leaves only | Comamonadaceae | Variovorax |
| ASV_1157 | -0.80 | 1.06 | 0.94 | 0.76 | 0.19 | 0.21 | -1.06 | 5.46 | 4.52 | 0.30 | 0.00 | 0.00 | 0.78 | 0.00 | 0.00 | Leaves and Flowers | Unclassified | Unclassified |
| ASV_117 | 0.48 | -1.01 | -0.56 | 0.74 | 0.19 | 0.20 | 0.65 | -5.24 | -2.80 | 0.52 | 0.00 | 0.01 | 0.94 | 0.00 | 0.01 | Leaves and Flowers | Microbacteriaceae | Galbitalea |
| ASV_118 | -2.95 | -0.01 | 1.78 | 0.75 | 0.20 | 0.21 | -3.95 | -0.06 | 8.29 | 0.00 | 0.95 | 0.00 | 0.02 | 1.00 | 0.00 | Flowers only | Pseudomonadaceae | Pseudomonas |
| ASV_12 | -0.47 | -0.11 | 0.73 | 0.71 | 0.24 | 0.26 | -0.67 | -0.45 | 2.88 | 0.51 | 0.66 | 0.00 | 0.93 | 0.80 | 0.01 | Flowers only | Rhizobiaceae | Allorhizobium. |
| ASV_120 | -0.76 | 0.41 | 0.95 | 0.73 | 0.20 | 0.20 | -1.03 | 2.07 | 4.65 | 0.31 | 0.04 | 0.00 | 0.78 | 0.07 | 0.00 | Flowers only | Comamonadaceae | Unclassified |
| ASV_121 | -0.36 | 0.14 | 0.16 | 0.72 | 0.23 | 0.24 | -0.50 | 0.60 | 0.68 | 0.62 | 0.55 | 0.50 | 1.00 | 0.69 | 0.60 | Not different | Comamonadaceae | Variovorax |
| ASV_122 | -2.72 | 3.35 | 2.64 | 0.77 | 0.20 | 0.21 | -3.52 | 16.90 | 12.65 | 0.00 | 0.00 | 0.00 | 0.08 | 0.00 | 0.00 | Leaves and Flowers | Comamonadaceae | Unclassified |
| ASV_123 | 0.04 | 0.84 | 0.14 | 0.74 | 0.20 | 0.20 | 0.06 | 4.20 | 0.71 | 0.96 | 0.00 | 0.48 | 1.00 | 0.00 | 0.59 | Leaves only | Flavobacteriaceae | Flavobacterium |
| ASV_124 | -1.07 | -0.17 | 1.25 | 0.67 | 0.21 | 0.21 | -1.59 | -0.84 | 6.05 | 0.11 | 0.40 | 0.00 | 0.47 | 0.53 | 0.00 | Flowers only | Unclassified | Unclassified |
| ASV_125 | 0.90 | -0.08 | -0.03 | 0.71 | 0.20 | 0.21 | 1.27 | -0.41 | -0.15 | 0.21 | 0.69 | 0.88 | 0.63 | 0.82 | 0.97 | Not different | Oxalobacteraceae | Janthinobacterium |
| ASV_1284 | -0.64 | 1.31 | 0.28 | 0.76 | 0.20 | 0.21 | -0.84 | 6.52 | 1.34 | 0.41 | 0.00 | 0.19 | 0.90 | 0.00 | 0.26 | Leaves only | Oxalobacteraceae | Unclassified |
| ASV_13 | -0.99 | 1.56 | 2.59 | 0.64 | 0.23 | 0.25 | -1.55 | 6.92 | 10.51 | 0.12 | 0.00 | 0.00 | 0.48 | 0.00 | 0.00 | Leaves and Flowers | Oxalobacteraceae | Duganella |
| ASV_132 | -0.02 | 0.04 | -0.50 | 0.75 | 0.20 | 0.21 | -0.03 | 0.17 | -2.37 | 0.97 | 0.86 | 0.02 | 1.00 | 0.98 | 0.04 | Flowers only | Oxalobacteraceae | Duganella |
| ASV_134 | -0.46 | -0.21 | 1.83 | 0.76 | 0.20 | 0.21 | -0.60 | -1.02 | 8.82 | 0.56 | 0.32 | 0.00 | 0.95 | 0.43 | 0.00 | Flowers only | Oxalobacteraceae | Unclassified |
| ASV_135 | -0.08 | 1.05 | 0.18 | 0.68 | 0.20 | 0.20 | -0.12 | 5.28 | 0.89 | 0.91 | 0.00 | 0.38 | 1.00 | 0.00 | 0.48 | Leaves only | Microbacteriaceae | Frondihabitans |
| ASV_136 | -0.10 | 0.28 | 0.77 | 0.75 | 0.19 | 0.21 | -0.14 | 1.47 | 3.65 | 0.89 | 0.15 | 0.00 | 1.00 | 0.22 | 0.00 | Flowers only | Erwiniaceae | Unclassified |
| ASV_137 | -0.19 | -0.08 | -0.76 | 0.71 | 0.20 | 0.19 | -0.27 | -0.41 | -4.00 | 0.79 | 0.68 | 0.00 | 1.00 | 0.82 | 0.00 | Flowers only | Oxalobacteraceae | Unclassified |
| ASV_1376 | 1.14 | -1.63 | -1.48 | 0.76 | 0.20 | 0.20 | 1.50 | -8.26 | -7.26 | 0.15 | 0.00 | 0.00 | 0.53 | 0.00 | 0.00 | Leaves and Flowers | Oxalobacteraceae | Unclassified |
| ASV_138 | 0.63 | -1.61 | 0.20 | 0.76 | 0.20 | 0.21 | 0.83 | -8.00 | 0.97 | 0.41 | 0.00 | 0.34 | 0.90 | 0.00 | 0.44 | Leaves only | Pseudomonadaceae | Pseudomonas |
| ASV_139 | -1.53 | -0.32 | 0.42 | 0.74 | 0.20 | 0.21 | -2.07 | -1.59 | 2.01 | 0.04 | 0.12 | 0.05 | 0.30 | 0.18 | 0.08 | Not different | Pseudomonadaceae | Pseudomonas |
| ASV_14 | -1.47 | 3.28 | 0.65 | 0.60 | 0.20 | 0.21 | -2.43 | 16.29 | 3.05 | 0.02 | 0.00 | 0.00 | 0.21 | 0.00 | 0.01 | Leaves and Flowers | Enterobacteriaceae | Unclassified |
| ASV_141 | -0.68 | 0.70 | 1.08 | 0.74 | 0.20 | 0.20 | -0.92 | 3.57 | 5.38 | 0.36 | 0.00 | 0.00 | 0.84 | 0.00 | 0.00 | Leaves and Flowers | Oxalobacteraceae | Unclassified |
| ASV_1416 | -1.71 | -0.76 | 0.30 | 0.76 | 0.20 | 0.20 | -2.26 | -3.87 | 1.46 | 0.03 | 0.00 | 0.15 | 0.28 | 0.00 | 0.22 | Leaves only | Unclassified | Unclassified |
| ASV_142 | -0.54 | -1.18 | -1.11 | 0.75 | 0.19 | 0.20 | -0.73 | -6.13 | -5.55 | 0.47 | 0.00 | 0.00 | 0.93 | 0.00 | 0.00 | Leaves and Flowers | Erwiniaceae | Erwinia |

| | | | | | | | | | | | | | | | | | | |
|---|---|---|---|---|---|---|---|---|---|---|---|---|---|---|---|---|---|---|
| ASV_143 | 0.42 | -0.75 | 0.32 | 0.77 | 0.20 | 0.21 | 0.55 | -3.73 | 1.48 | 0.58 | 0.00 | 0.15 | 0.98 | 0.00 | 0.21 | Leaves only | Yersiniaceae | Rahnella1 |
| ASV_144 | 1.41 | -1.27 | -1.19 | 0.74 | 0.22 | 0.22 | 1.90 | -5.89 | -5.35 | 0.06 | 0.00 | 0.00 | 0.36 | 0.00 | 0.00 | Leaves and Flowers | Xanthomonadaceae | Pseudoxanthomonas |
| ASV_145 | NA | NA | NA | NA | NA | NA | NA | NA | NA | 1.00 | 1.00 | 1.00 | 1.00 | 1.00 | 1.00 | Not different | Unclassified | Unclassified |
| ASV_147 | -0.14 | 0.62 | 0.66 | 0.73 | 0.20 | 0.21 | -0.19 | 3.17 | 3.08 | 0.85 | 0.00 | 0.00 | 1.00 | 0.01 | 0.01 | Leaves and Flowers | Oxalobacteraceae | Duganella |
| ASV_148 | -1.34 | 0.56 | 0.93 | 0.72 | 0.19 | 0.21 | -1.86 | 2.93 | 4.45 | 0.07 | 0.01 | 0.00 | 0.37 | 0.01 | 0.00 | Leaves and Flowers | Pseudomonadaceae | Pseudomonas |
| ASV_150 | -1.65 | 0.91 | 1.18 | 0.72 | 0.20 | 0.21 | -2.28 | 4.66 | 5.62 | 0.03 | 0.00 | 0.00 | 0.25 | 0.00 | 0.00 | Leaves and Flowers | Unclassified | Unclassified |
| ASV_152 | -0.45 | 0.27 | 0.35 | 0.72 | 0.20 | 0.21 | -0.63 | 1.35 | 1.63 | 0.53 | 0.18 | 0.11 | 0.95 | 0.26 | 0.16 | Not different | Sphingobacteriaceae | Mucilaginibacter |
| ASV_157 | -1.06 | 0.93 | 1.56 | 0.75 | 0.20 | 0.21 | -1.41 | 4.56 | 7.50 | 0.17 | 0.00 | 0.00 | 0.56 | 0.00 | 0.00 | Leaves and Flowers | Yersiniaceae | Rahnella1 |
| ASV_16 | -1.55 | 1.44 | 2.81 | 0.68 | 0.23 | 0.23 | -2.27 | 6.26 | 12.32 | 0.03 | 0.00 | 0.00 | 0.25 | 0.00 | 0.00 | Leaves and Flowers | Oxalobacteraceae | Duganella |
| ASV_162 | 0.81 | -0.86 | 0.06 | 0.75 | 0.20 | 0.20 | 1.08 | -4.21 | 0.29 | 0.29 | 0.00 | 0.77 | 0.78 | 0.00 | 0.86 | Leaves only | Oxalobacteraceae | Unclassified |
| ASV_168 | -2.53 | 2.94 | 2.38 | 0.75 | 0.20 | 0.21 | -3.38 | 14.54 | 11.19 | 0.00 | 0.00 | 0.00 | 0.06 | 0.00 | 0.00 | Leaves and Flowers | Oxalobacteraceae | Unclassified |
| ASV_169 | 0.59 | -0.70 | -0.17 | 0.74 | 0.20 | 0.21 | 0.80 | -3.50 | -0.83 | 0.43 | 0.00 | 0.41 | 0.91 | 0.00 | 0.52 | Leaves only | Flavobacteriaceae | Flavobacterium |
| ASV_17 | -1.02 | 1.52 | 1.10 | 0.53 | 0.23 | 0.27 | -1.91 | 6.48 | 4.12 | 0.06 | 0.00 | 0.00 | 0.36 | 0.00 | 0.00 | Leaves and Flowers | Erwiniaceae | Erwinia |
| ASV_170 | -0.13 | -1.42 | -0.23 | 0.72 | 0.21 | 0.21 | -0.19 | -6.93 | -1.07 | 0.85 | 0.00 | 0.29 | 1.00 | 0.00 | 0.38 | Leaves only | Sphingomonadaceae | Rhizorhapis |
| ASV_172 | -0.39 | 0.76 | 0.38 | 0.69 | 0.18 | 0.18 | -0.56 | 4.12 | 2.05 | 0.58 | 0.00 | 0.04 | 0.97 | 0.00 | 0.07 | Leaves only | Oxalobacteraceae | Unclassified |
| ASV_173 | -0.61 | -0.67 | -0.91 | 0.76 | 0.20 | 0.21 | -0.81 | -3.34 | -4.36 | 0.42 | 0.00 | 0.00 | 0.91 | 0.01 | 0.00 | Leaves and Flowers | Pseudonocardiaceae | Pseudonocardia |
| ASV_174 | 0.15 | -1.46 | -1.66 | 0.73 | 0.21 | 0.21 | 0.21 | -7.10 | -8.02 | 0.84 | 0.00 | 0.00 | 1.00 | 0.00 | 0.00 | Leaves and Flowers | Methylophilaceae | Unclassified |
| ASV_176 | -2.18 | -1.16 | -0.69 | 0.75 | 0.20 | 0.20 | -2.92 | -5.91 | -3.39 | 0.01 | 0.00 | 0.00 | 0.13 | 0.00 | 0.00 | Leaves and Flowers | Microbacteriaceae | Unclassified |
| ASV_177 | NA | NA | NA | NA | NA | NA | NA | NA | NA | 1.00 | 1.00 | 1.00 | 1.00 | 1.00 | 1.00 | Not different | Unclassified | Unclassified |
| ASV_178 | 0.18 | -1.56 | -0.86 | 0.76 | 0.21 | 0.21 | 0.24 | -7.50 | -4.07 | 0.81 | 0.00 | 0.00 | 1.00 | 0.00 | 0.00 | Leaves and Flowers | Sphingomonadaceae | Sphingobium |
| ASV_183 | -0.38 | -0.61 | 0.11 | 0.76 | 0.21 | 0.23 | -0.50 | -2.88 | 0.49 | 0.62 | 0.01 | 0.63 | 1.00 | 0.01 | 0.72 | Leaves only | Pseudomonadaceae | Pseudomonas |
| ASV_184 | -0.10 | -0.69 | -0.30 | 0.70 | 0.20 | 0.21 | -0.14 | -3.49 | -1.44 | 0.89 | 0.00 | 0.15 | 1.00 | 0.00 | 0.22 | Leaves only | Xanthobacteraceae | Bradyrhizobium |
| ASV_186 | -0.38 | 0.13 | 0.84 | 0.73 | 0.19 | 0.20 | -0.51 | 0.71 | 4.17 | 0.61 | 0.48 | 0.00 | 1.00 | 0.60 | 0.00 | Flowers only | Erwiniaceae | Pantoea |
| ASV_188 | -0.48 | 1.44 | 0.98 | 0.73 | 0.19 | 0.21 | -0.65 | 7.59 | 4.68 | 0.52 | 0.00 | 0.00 | 0.94 | 0.00 | 0.00 | Leaves and Flowers | Pseudomonadaceae | Pseudomonas |
| ASV_189 | -0.64 | -1.18 | -1.06 | 0.72 | 0.19 | 0.20 | -0.90 | -6.07 | -5.29 | 0.37 | 0.00 | 0.00 | 0.85 | 0.00 | 0.00 | Leaves and Flowers | Caulobacteraceae | Caulobacter |
| ASV_19 | -0.35 | 1.20 | 0.22 | 0.57 | 0.24 | 0.23 | -0.61 | 4.93 | 0.96 | 0.54 | 0.00 | 0.34 | 0.95 | 0.00 | 0.44 | Leaves only | Sphingomonadaceae | Sphingomonas |
| ASV_193 | -1.41 | -1.49 | 0.37 | 0.76 | 0.20 | 0.21 | -1.86 | -7.54 | 1.73 | 0.08 | 0.00 | 0.10 | 0.37 | 0.00 | 0.15 | Leaves only | Oxalobacteraceae | Unclassified |
| ASV_195 | -0.33 | -0.01 | 0.28 | 0.75 | 0.19 | 0.22 | -0.45 | -0.07 | 1.26 | 0.66 | 0.94 | 0.22 | 1.00 | 1.00 | 0.30 | Not different | Pseudomonadaceae | Pseudomonas |
| ASV_196 | -0.95 | -0.28 | 1.01 | 0.77 | 0.20 | 0.20 | -1.24 | -1.44 | 5.02 | 0.23 | 0.17 | 0.00 | 0.65 | 0.24 | 0.00 | Flowers only | Pseudomonadaceae | Pseudomonas |
| ASV_2 | -2.33 | 1.13 | 2.96 | 0.65 | 0.22 | 0.25 | -3.58 | 5.18 | 12.05 | 0.00 | 0.00 | 0.00 | 0.02 | 0.00 | 0.00 | Leaves and Flowers | Pseudomonadaceae | Pseudomonas |
| ASV_20 | -2.48 | 0.44 | 2.41 | 0.69 | 0.21 | 0.22 | -3.59 | 2.14 | 10.73 | 0.00 | 0.04 | 0.00 | 0.02 | 0.06 | 0.00 | Flowers only | Pseudomonadaceae | Pseudomonas |
| ASV_200 | NA | NA | NA | NA | NA | NA | NA | NA | NA | 1.00 | 1.00 | 1.00 | 1.00 | 1.00 | 1.00 | Not different | Microbacteriaceae | Subtercola |
| ASV_203 | -1.10 | 0.86 | 2.21 | 0.77 | 0.20 | 0.21 | -1.43 | 4.21 | 10.46 | 0.17 | 0.00 | 0.00 | 0.56 | 0.00 | 0.00 | Leaves and Flowers | Oxalobacteraceae | Duganella |
| ASV_204 | 1.95 | -0.36 | -0.79 | 0.75 | 0.21 | 0.21 | 2.59 | -1.74 | -3.75 | 0.01 | 0.09 | 0.00 | 0.19 | 0.14 | 0.00 | Flowers only | Flavobacteriaceae | Flavobacterium |
| ASV_205 | -3.24 | 1.56 | 2.50 | 0.74 | 0.20 | 0.22 | -4.39 | 7.64 | 11.31 | 0.00 | 0.00 | 0.00 | 0.01 | 0.00 | 0.00 | Leaves and Flowers | Pseudomonadaceae | Pseudomonas |
| ASV_209 | -0.88 | -0.18 | 0.48 | 0.69 | 0.19 | 0.23 | -1.27 | -0.94 | 2.08 | 0.21 | 0.35 | 0.04 | 0.63 | 0.46 | 0.07 | Not different | Pseudomonadaceae | Pseudomonas |
| ASV_21 | -1.28 | -0.04 | 2.22 | 0.61 | 0.23 | 0.23 | -2.10 | -0.20 | 9.48 | 0.04 | 0.84 | 0.00 | 0.29 | 0.96 | 0.00 | Flowers only | Pseudomonadaceae | Pseudomonas |
| ASV_210 | 0.04 | -1.68 | -0.50 | 0.70 | 0.19 | 0.20 | 0.05 | -8.64 | -2.42 | 0.96 | 0.00 | 0.02 | 1.00 | 0.00 | 0.03 | Leaves and Flowers | Xanthobacteraceae | Tardiphaga |
| ASV_212 | NA | NA | NA | NA | NA | NA | NA | NA | NA | 1.00 | 1.00 | 1.00 | 1.00 | 1.00 | 1.00 | Not different | Unclassified | Unclassified |
| ASV_22 | -0.63 | -0.64 | 0.83 | 0.64 | 0.28 | 0.28 | -0.98 | -2.28 | 2.99 | 0.33 | 0.02 | 0.00 | 0.80 | 0.04 | 0.01 | Leaves and Flowers | Pseudomonadaceae | Pseudomonas |
| ASV_220 | -0.55 | 0.67 | 0.98 | 0.73 | 0.21 | 0.22 | -0.75 | 3.27 | 4.46 | 0.46 | 0.00 | 0.00 | 0.93 | 0.00 | 0.00 | Leaves and Flowers | Oxalobacteraceae | Unclassified |
| ASV_224 | -0.31 | -0.16 | 0.48 | 0.73 | 0.20 | 0.21 | -0.43 | -0.81 | 2.30 | 0.67 | 0.42 | 0.03 | 1.00 | 0.55 | 0.04 | Flowers only | Sphingobacteriaceae | Pedobacter |
| ASV_227 | 0.56 | 0.58 | 0.41 | 0.77 | 0.20 | 0.21 | 0.73 | 2.86 | 1.98 | 0.48 | 0.01 | 0.07 | 0.93 | 0.02 | 0.10 | Leaves only | Oxalobacteraceae | Unclassified |
| ASV_23 | -0.92 | -0.02 | 1.45 | 0.65 | 0.20 | 0.24 | -1.40 | -0.09 | 6.13 | 0.16 | 0.93 | 0.00 | 0.56 | 1.00 | 0.00 | Flowers only | Pseudomonadaceae | Pseudomonas |
| ASV_232 | 0.19 | -1.19 | -1.06 | 0.75 | 0.19 | 0.20 | 0.26 | -6.15 | -5.34 | 0.80 | 0.00 | 0.00 | 1.00 | 0.00 | 0.00 | Leaves and Flowers | Microbacteriaceae | Subtercola |
| ASV_235 | -1.29 | -0.63 | 0.46 | 0.74 | 0.20 | 0.21 | -1.74 | -3.20 | 2.16 | 0.09 | 0.00 | 0.04 | 0.41 | 0.01 | 0.06 | Leaves only | Rhizobiaceae | Allorhizobium. |
| ASV_239 | 0.78 | -0.08 | 0.23 | 0.72 | 0.20 | 0.21 | 1.08 | -0.40 | 1.14 | 0.29 | 0.69 | 0.26 | 0.78 | 0.82 | 0.35 | Not different | Cellulomonadaceae | Cellulomonas |
| ASV_24 | -1.49 | 1.44 | 3.12 | 0.70 | 0.26 | 0.26 | -2.11 | 5.61 | 12.04 | 0.04 | 0.00 | 0.00 | 0.29 | 0.00 | 0.00 | Leaves and Flowers | Oxalobacteraceae | Duganella |
| ASV_242 | 0.43 | 0.18 | 1.13 | 0.76 | 0.20 | 0.21 | 0.57 | 0.91 | 5.48 | 0.58 | 0.37 | 0.00 | 0.97 | 0.49 | 0.00 | Flowers only | Oxalobacteraceae | Unclassified |
| ASV_244 | NA | NA | NA | NA | NA | NA | NA | NA | NA | 1.00 | 1.00 | 1.00 | 1.00 | 1.00 | 1.00 | Not different | Unclassified | Unclassified |
| ASV_246 | -0.74 | 1.07 | 0.34 | 0.76 | 0.22 | 0.22 | -0.97 | 4.87 | 1.54 | 0.34 | 0.00 | 0.13 | 0.82 | 0.00 | 0.19 | Leaves only | Flavobacteriaceae | Flavobacterium |
| ASV_251 | -1.42 | -0.29 | 0.34 | 0.74 | 0.19 | 0.20 | -1.91 | -1.47 | 1.66 | 0.06 | 0.15 | 0.11 | 0.36 | 0.22 | 0.16 | Not different | Burkholderiaceae | Burkholderia. |

| | | | | | | | | | | | | | | | | | | |
|---|---|---|---|---|---|---|---|---|---|---|---|---|---|---|---|---|---|---|
| ASV_252 | -1.84 | 1.30 | 1.93 | 0.75 | 0.20 | 0.20 | -2.45 | 6.53 | 9.84 | 0.02 | 0.00 | 0.00 | 0.22 | 0.00 | 0.00 | Leaves and Flowers | Oxalobacteraceae | Duganella |
| ASV_2529 | -1.44 | 0.74 | -0.13 | 0.77 | 0.20 | 0.20 | -1.88 | 3.75 | -0.62 | 0.08 | 0.00 | 0.54 | 0.37 | 0.00 | 0.64 | Leaves only | Unclassified | Unclassified |
| ASV_254 | -0.71 | -0.33 | -0.35 | 0.75 | 0.20 | 0.21 | -0.94 | -1.60 | -1.66 | 0.35 | 0.12 | 0.11 | 0.84 | 0.18 | 0.16 | Not different | Flavobacteriaceae | Flavobacterium |
| ASV_256 | -0.42 | -0.70 | 0.70 | 0.71 | 0.20 | 0.21 | -0.59 | -3.47 | 3.31 | 0.56 | 0.00 | 0.00 | 0.95 | 0.00 | 0.00 | Leaves and Flowers | Unclassified | Unclassified |
| ASV_258 | 0.15 | -0.59 | -0.16 | 0.73 | 0.22 | 0.23 | 0.20 | -2.63 | -0.70 | 0.84 | 0.01 | 0.49 | 1.00 | 0.02 | 0.59 | Leaves only | Sphingobacteriaceae | Mucilaginibacter |
| ASV_26 | -0.35 | 0.92 | -0.39 | 0.76 | 0.25 | 0.24 | -0.46 | 3.65 | -1.62 | 0.65 | 0.00 | 0.11 | 1.00 | 0.00 | 0.17 | Leaves only | Unclassified | Unclassified |
| ASV_263 | 0.19 | -0.15 | -0.14 | 0.76 | 0.20 | 0.21 | 0.25 | -0.75 | -0.66 | 0.80 | 0.46 | 0.51 | 1.00 | 0.58 | 0.61 | Not different | Sphingobacteriaceae | Pedobacter |
| ASV_264 | NA | NA | NA | NA | NA | NA | NA | NA | NA | 1.00 | 1.00 | 1.00 | 1.00 | 1.00 | 1.00 | Not different | Unclassified | Unclassified |
| ASV_265 | 0.26 | -1.17 | -1.53 | 0.74 | 0.19 | 0.20 | 0.35 | -6.02 | -7.70 | 0.73 | 0.00 | 0.00 | 1.00 | 0.00 | 0.00 | Leaves and Flowers | Sphingomonadaceae | Novosphingobium |
| ASV_267 | -0.86 | -0.48 | 0.97 | 0.71 | 0.21 | 0.22 | -1.22 | -2.29 | 4.35 | 0.23 | 0.03 | 0.00 | 0.65 | 0.04 | 0.00 | Leaves and Flowers | Unclassified | Unclassified |
| ASV_27 | -1.76 | 3.62 | 1.00 | 0.64 | 0.21 | 0.26 | -2.75 | 16.94 | 3.89 | 0.01 | 0.00 | 0.00 | 0.13 | 0.00 | 0.00 | Leaves and Flowers | Oxalobacteraceae | Janthinobacterium |
| ASV_271 | -0.13 | -1.38 | -0.98 | 0.74 | 0.19 | 0.20 | -0.18 | -7.20 | -4.88 | 0.86 | 0.00 | 0.00 | 1.00 | 0.00 | 0.00 | Leaves and Flowers | Sphingomonadaceae | Rhizorhapis |
| ASV_272 | NA | NA | NA | NA | NA | NA | NA | NA | NA | 1.00 | 1.00 | 1.00 | 1.00 | 1.00 | 1.00 | Not different | Unclassified | Unclassified |
| ASV_277 | -0.76 | -0.07 | 0.44 | 0.76 | 0.21 | 0.22 | -1.01 | -0.34 | 2.04 | 0.32 | 0.74 | 0.05 | 0.80 | 0.86 | 0.08 | Not different | Rhodanobacteraceae | Dyella |
| ASV_28 | -1.29 | 2.01 | 0.63 | 0.63 | 0.19 | 0.21 | -2.03 | 10.50 | 3.04 | 0.04 | 0.00 | 0.00 | 0.30 | 0.00 | 0.01 | Leaves and Flowers | Oxalobacteraceae | Janthinobacterium |
| ASV_280 | 0.52 | -1.29 | -1.25 | 0.74 | 0.20 | 0.20 | 0.70 | -6.53 | -6.14 | 0.49 | 0.00 | 0.00 | 0.93 | 0.00 | 0.00 | Leaves and Flowers | Sphingomonadaceae | Sphingomonas |
| ASV_283 | -0.07 | -0.29 | 1.00 | 0.74 | 0.19 | 0.22 | -0.10 | -1.47 | 4.58 | 0.92 | 0.15 | 0.00 | 1.00 | 0.22 | 0.00 | Flowers only | Pseudomonadaceae | Pseudomonas |
| ASV_284 | 0.54 | -0.74 | -0.11 | 0.74 | 0.20 | 0.20 | 0.73 | -3.72 | -0.55 | 0.47 | 0.00 | 0.58 | 0.93 | 0.00 | 0.68 | Leaves only | Microbacteriaceae | Herbiconiux |
| ASV_285 | 0.31 | 0.28 | -0.27 | 0.73 | 0.20 | 0.20 | 0.43 | 1.41 | -1.39 | 0.67 | 0.16 | 0.17 | 1.00 | 0.24 | 0.24 | Not different | Microbacteriaceae | Unclassified |
| ASV_294 | -0.46 | 0.58 | -0.60 | 0.76 | 0.20 | 0.21 | -0.60 | 2.96 | -2.92 | 0.55 | 0.01 | 0.01 | 0.95 | 0.01 | 0.02 | Leaves and Flowers | Oxalobacteraceae | Unclassified |
| ASV_295 | -1.23 | 1.58 | -0.14 | 0.77 | 0.20 | 0.20 | -1.60 | 8.02 | -0.73 | 0.13 | 0.00 | 0.48 | 0.50 | 0.00 | 0.58 | Leaves only | Sphingobacteriaceae | Pedobacter |
| ASV_297 | -3.59 | -0.03 | 1.30 | 0.77 | 0.20 | 0.20 | -4.64 | -0.13 | 6.42 | 0.00 | 0.90 | 0.00 | 0.02 | 1.00 | 0.00 | Flowers only | Unclassified | Unclassified |
| ASV_298 | -2.11 | -0.19 | 1.57 | 0.74 | 0.19 | 0.21 | -2.84 | -1.00 | 7.34 | 0.01 | 0.32 | 0.00 | 0.13 | 0.43 | 0.00 | Flowers only | Pseudomonadaceae | Pseudomonas |
| ASV_3 | -0.84 | 2.83 | 0.82 | 0.56 | 0.20 | 0.21 | -1.51 | 14.22 | 3.87 | 0.13 | 0.00 | 0.00 | 0.50 | 0.00 | 0.00 | Leaves and Flowers | Oxalobacteraceae | Janthinobacterium |
| ASV_30 | -1.11 | 1.71 | 2.31 | 0.66 | 0.21 | 0.23 | -1.68 | 8.27 | 9.93 | 0.10 | 0.00 | 0.00 | 0.43 | 0.00 | 0.00 | Leaves and Flowers | Oxalobacteraceae | Duganella |
| ASV_301 | 0.52 | -0.42 | -0.63 | 0.73 | 0.20 | 0.20 | 0.72 | -2.13 | -3.12 | 0.47 | 0.04 | 0.00 | 0.93 | 0.06 | 0.01 | Flowers only | Nakamurellaceae | Nakamurella |
| ASV_304 | -0.23 | -1.04 | 0.25 | 0.75 | 0.21 | 0.21 | -0.31 | -4.92 | 1.19 | 0.76 | 0.00 | 0.24 | 1.00 | 0.00 | 0.33 | Leaves only | Unclassified | Unclassified |
| ASV_309 | 2.08 | 0.83 | -2.10 | 0.76 | 0.20 | 0.21 | 2.75 | 4.11 | -10.05 | 0.01 | 0.00 | 0.00 | 0.16 | 0.00 | 0.00 | Leaves and Flowers | Comamonadaceae | Variovorax |
| ASV_31 | -0.84 | 0.08 | 1.82 | 0.60 | 0.23 | 0.24 | -1.39 | 0.33 | 7.58 | 0.17 | 0.74 | 0.00 | 0.56 | 0.86 | 0.00 | Flowers only | Pseudomonadaceae | Pseudomonas |
| ASV_312 | -0.35 | 1.07 | 0.15 | 0.73 | 0.20 | 0.20 | -0.48 | 5.29 | 0.76 | 0.64 | 0.00 | 0.45 | 1.00 | 0.00 | 0.56 | Leaves only | Propionibacteriaceae | Cutibacterium |
| ASV_32 | -1.18 | -2.02 | -0.33 | 0.71 | 0.20 | 0.23 | -1.66 | -10.00 | -1.47 | 0.10 | 0.00 | 0.15 | 0.43 | 0.00 | 0.21 | Leaves only | Rhizobiaceae | Allorhizobium. |
| ASV_321 | 0.36 | -1.48 | 0.31 | 0.75 | 0.20 | 0.20 | 0.49 | -7.53 | 1.56 | 0.63 | 0.00 | 0.13 | 1.00 | 0.00 | 0.19 | Leaves only | Comamonadaceae | Leptothrix |
| ASV_324 | -0.81 | -0.23 | -0.53 | 0.77 | 0.19 | 0.20 | -1.06 | -1.19 | -2.69 | 0.31 | 0.25 | 0.02 | 0.78 | 0.34 | 0.03 | Flowers only | Sphingobacteriaceae | Pedobacter |
| ASV_325 | -1.79 | 1.37 | 1.59 | 0.74 | 0.19 | 0.19 | -2.41 | 7.22 | 8.17 | 0.02 | 0.00 | 0.00 | 0.22 | 0.00 | 0.00 | Leaves and Flowers | Sphingomonadaceae | Unclassified |
| ASV_331 | 0.05 | -0.42 | 0.09 | 0.72 | 0.19 | 0.20 | 0.06 | -2.21 | 0.43 | 0.95 | 0.03 | 0.67 | 1.00 | 0.05 | 0.77 | Not different | Mycobacteriaceae | Mycobacterium |
| ASV_333 | NA | NA | NA | NA | NA | NA | NA | NA | NA | 1.00 | 1.00 | 1.00 | 1.00 | 1.00 | 1.00 | Not different | Burkholderiaceae | Burkholderia. |
| ASV_335 | NA | NA | NA | NA | NA | NA | NA | NA | NA | 1.00 | 1.00 | 1.00 | 1.00 | 1.00 | 1.00 | Not different | Unclassified | Unclassified |
| ASV_336 | NA | NA | NA | NA | NA | NA | NA | NA | NA | 1.00 | 1.00 | 1.00 | 1.00 | 1.00 | 1.00 | Not different | Rhizobiaceae | Candidatus Liberibacter |
| ASV_338 | -0.08 | -1.55 | -0.53 | 0.75 | 0.19 | 0.20 | -0.10 | -7.93 | -2.63 | 0.92 | 0.00 | 0.01 | 1.00 | 0.00 | 0.02 | Leaves and Flowers | Unclassified | Unclassified |
| ASV_339 | NA | NA | NA | NA | NA | NA | NA | NA | NA | 1.00 | 1.00 | 1.00 | 1.00 | 1.00 | 1.00 | Not different | Unclassified | Unclassified |
| ASV_34 | -1.05 | 1.80 | 1.39 | 0.61 | 0.19 | 0.20 | -1.71 | 9.51 | 6.93 | 0.09 | 0.00 | 0.00 | 0.41 | 0.00 | 0.00 | Leaves and Flowers | Oxalobacteraceae | Massilia |
| ASV_35 | 1.06 | -1.14 | -1.41 | 0.74 | 0.26 | 0.23 | 1.43 | -4.47 | -6.12 | 0.16 | 0.00 | 0.00 | 0.55 | 0.00 | 0.00 | Leaves and Flowers | Rhizobiaceae | Candidatus Liberibacter |
| ASV_356 | 0.71 | -1.17 | -0.79 | 0.76 | 0.19 | 0.20 | 0.94 | -6.05 | -3.97 | 0.36 | 0.00 | 0.00 | 0.84 | 0.00 | 0.00 | Leaves and Flowers | Rhizobiaceae | Unclassified |
| ASV_36 | -2.00 | 0.05 | 2.11 | 0.74 | 0.20 | 0.22 | -2.72 | 0.24 | 9.73 | 0.01 | 0.81 | 0.00 | 0.16 | 0.93 | 0.00 | Flowers only | Pseudomonadaceae | Pseudomonas |
| ASV_361 | -0.44 | -0.91 | -1.12 | 0.72 | 0.19 | 0.19 | -0.61 | -4.78 | -5.82 | 0.55 | 0.00 | 0.00 | 0.95 | 0.00 | 0.00 | Leaves and Flowers | Sphingobacteriaceae | Mucilaginibacter |
| ASV_37 | -0.79 | -1.07 | -0.56 | 0.69 | 0.20 | 0.24 | -1.14 | -5.24 | -2.30 | 0.26 | 0.00 | 0.02 | 0.72 | 0.00 | 0.04 | Leaves and Flowers | Comamonadaceae | Unclassified |
| ASV_379 | 0.84 | -0.15 | -0.35 | 0.77 | 0.19 | 0.20 | 1.08 | -0.80 | -1.76 | 0.30 | 0.44 | 0.10 | 0.78 | 0.56 | 0.15 | Not different | Oxalobacteraceae | Unclassified |
| ASV_39 | -0.90 | -0.49 | -0.15 | 0.71 | 0.20 | 0.23 | -1.27 | -2.42 | -0.66 | 0.21 | 0.02 | 0.51 | 0.63 | 0.03 | 0.61 | Leaves only | Burkholderiaceae | Unclassified |
| ASV_391 | -0.52 | 1.01 | 0.71 | 0.73 | 0.20 | 0.20 | -0.71 | 5.13 | 3.64 | 0.48 | 0.00 | 0.00 | 0.93 | 0.00 | 0.00 | Leaves and Flowers | Propionibacteriaceae | Cutibacterium |
| ASV_393 | -0.63 | 0.28 | 0.33 | 0.69 | 0.20 | 0.22 | -0.91 | 1.38 | 1.51 | 0.36 | 0.17 | 0.14 | 0.84 | 0.24 | 0.20 | Not different | Rhizobiaceae | Allorhizobium. |
| ASV_394 | -1.65 | 1.22 | 0.56 | 0.76 | 0.19 | 0.19 | -2.16 | 6.33 | 2.90 | 0.04 | 0.00 | 0.01 | 0.30 | 0.00 | 0.02 | Leaves and Flowers | Rhizobiaceae | Aureimonas |
| ASV_4 | -1.55 | 3.14 | 2.43 | 0.56 | 0.23 | 0.30 | -2.76 | 13.70 | 8.20 | 0.01 | 0.00 | 0.00 | 0.13 | 0.00 | 0.00 | Leaves and Flowers | Burkholderiaceae | Ralstonia |

| | | | | | | | | | | | | | | | | | | |
|---|---|---|---|---|---|---|---|---|---|---|---|---|---|---|---|---|---|---|
| ASV_402 | -0.50 | 0.02 | -0.22 | 0.75 | 0.19 | 0.20 | -0.67 | 0.09 | -1.09 | 0.50 | 0.93 | 0.28 | 0.93 | 1.00 | 0.38 | Not different | Sphingobacteriaceae | Mucilaginibacter |
| ASV_405 | 0.07 | -0.08 | -0.20 | 0.76 | 0.19 | 0.20 | 0.09 | -0.43 | -0.98 | 0.93 | 0.67 | 0.33 | 1.00 | 0.81 | 0.44 | Not different | Unclassified | Unclassified |
| ASV_406 | 0.32 | 0.36 | -0.70 | 0.73 | 0.20 | 0.20 | 0.43 | 1.85 | -3.51 | 0.67 | 0.07 | 0.00 | 1.00 | 0.11 | 0.00 | Flowers only | Caulobacteraceae | Brevundimonas |
| ASV_41 | -1.37 | 0.01 | 2.33 | 0.64 | 0.23 | 0.23 | -2.14 | 0.03 | 10.05 | 0.03 | 0.97 | 0.00 | 0.29 | 1.00 | 0.00 | Flowers only | Pseudomonadaceae | Pseudomonas |
| ASV_42 | -1.42 | 0.28 | 1.69 | 0.69 | 0.24 | 0.23 | -2.06 | 1.17 | 7.19 | 0.04 | 0.24 | 0.00 | 0.30 | 0.34 | 0.00 | Flowers only | Pseudomonadaceae | Pseudomonas |
| ASV_428 | 0.12 | -0.12 | 1.16 | 0.74 | 0.21 | 0.22 | 0.16 | -0.56 | 5.30 | 0.87 | 0.58 | 0.00 | 1.00 | 0.71 | 0.00 | Flowers only | Unclassified | Unclassified |
| ASV_431 | -0.62 | 1.12 | 0.74 | 0.75 | 0.21 | 0.22 | -0.82 | 5.25 | 3.44 | 0.42 | 0.00 | 0.00 | 0.91 | 0.00 | 0.00 | Leaves and Flowers | Oxalobacteraceae | Unclassified |
| ASV_433 | 0.02 | 0.20 | 0.02 | 0.77 | 0.19 | 0.20 | 0.02 | 1.06 | 0.08 | 0.98 | 0.31 | 0.94 | 1.00 | 0.42 | 1.00 | Not different | Oxalobacteraceae | Unclassified |
| ASV_436 | -0.37 | -0.80 | 1.42 | 0.75 | 0.19 | 0.20 | -0.50 | -4.10 | 6.99 | 0.62 | 0.00 | 0.00 | 1.00 | 0.00 | 0.00 | Leaves and Flowers | Flavobacteriaceae | Unclassified |
| ASV_44 | -0.18 | 0.40 | 1.19 | 0.66 | 0.25 | 0.26 | -0.28 | 1.56 | 4.55 | 0.78 | 0.12 | 0.00 | 1.00 | 0.18 | 0.00 | Flowers only | Pseudomonadaceae | Pseudomonas |
| ASV_442 | -2.14 | 1.84 | 0.66 | 0.76 | 0.19 | 0.20 | -2.80 | 9.58 | 3.23 | 0.01 | 0.00 | 0.00 | 0.16 | 0.00 | 0.01 | Leaves and Flowers | Comamonadaceae | Xylophilus |
| ASV_444 | -0.17 | 0.29 | 0.17 | 0.74 | 0.19 | 0.19 | -0.23 | 1.53 | 0.87 | 0.82 | 0.13 | 0.39 | 1.00 | 0.20 | 0.50 | Not different | Comamonadaceae | Unclassified |
| ASV_46 | -1.16 | 1.33 | 2.00 | 0.67 | 0.19 | 0.22 | -1.73 | 6.86 | 9.22 | 0.09 | 0.00 | 0.00 | 0.41 | 0.00 | 0.00 | Leaves and Flowers | Oxalobacteraceae | Duganella |
| ASV_461 | 0.53 | 0.75 | -0.30 | 0.73 | 0.20 | 0.21 | 0.73 | 3.77 | -1.43 | 0.47 | 0.00 | 0.16 | 0.93 | 0.00 | 0.22 | Leaves only | Nakamurellaceae | Nakamurella |
| ASV_47 | -0.77 | 1.13 | 1.34 | 0.62 | 0.19 | 0.20 | -1.23 | 5.95 | 6.67 | 0.22 | 0.00 | 0.00 | 0.65 | 0.00 | 0.00 | Leaves and Flowers | Oxalobacteraceae | Unclassified |
| ASV_472 | NA | NA | NA | NA | NA | NA | NA | NA | NA | 1.00 | 1.00 | 1.00 | 1.00 | 1.00 | 1.00 | Not different | Unclassified | Unclassified |
| ASV_474 | NA | NA | NA | NA | NA | NA | NA | NA | NA | 1.00 | 1.00 | 1.00 | 1.00 | 1.00 | 1.00 | Not different | Microbacteriaceae | Unclassified |
| ASV_477 | 1.15 | -1.07 | -0.23 | 0.77 | 0.19 | 0.20 | 1.49 | -5.60 | -1.16 | 0.16 | 0.00 | 0.27 | 0.56 | 0.00 | 0.36 | Leaves only | Microbacteriaceae | Microbacterium |
| ASV_478 | 0.31 | 0.07 | -0.67 | 0.73 | 0.19 | 0.20 | 0.42 | 0.39 | -3.37 | 0.67 | 0.70 | 0.00 | 1.00 | 0.82 | 0.00 | Flowers only | Sphingobacteriaceae | Mucilaginibacter |
| ASV_483 | -0.46 | 0.15 | -0.51 | 0.76 | 0.19 | 0.20 | -0.61 | 0.76 | -2.60 | 0.55 | 0.45 | 0.02 | 0.95 | 0.58 | 0.03 | Flowers only | Sphingobacteriaceae | Mucilaginibacter |
| ASV_487 | -0.19 | 1.29 | 1.00 | 0.76 | 0.19 | 0.20 | -0.24 | 6.69 | 5.04 | 0.81 | 0.00 | 0.00 | 1.00 | 0.00 | 0.00 | Leaves and Flowers | Oxalobacteraceae | Unclassified |
| ASV_488 | NA | NA | NA | NA | NA | NA | NA | NA | NA | 1.00 | 1.00 | 1.00 | 1.00 | 1.00 | 1.00 | Not different | Rhodanobacteraceae | Unclassified |
| ASV_49 | -1.02 | 0.04 | 1.38 | 0.64 | 0.26 | 0.26 | -1.61 | 0.14 | 5.29 | 0.11 | 0.89 | 0.00 | 0.46 | 1.00 | 0.00 | Flowers only | Pseudomonadaceae | Pseudomonas |
| ASV_492 | -0.21 | 1.29 | 0.74 | 0.76 | 0.20 | 0.20 | -0.27 | 6.63 | 3.70 | 0.79 | 0.00 | 0.00 | 1.00 | 0.00 | 0.00 | Leaves and Flowers | Rhizobiaceae | Allorhizobium. |
| ASV_495 | NA | NA | NA | NA | NA | NA | NA | NA | NA | 1.00 | 1.00 | 1.00 | 1.00 | 1.00 | 1.00 | Not different | Unclassified | Unclassified |
| ASV_5 | -0.89 | 0.57 | 1.37 | 0.52 | 0.23 | 0.24 | -1.71 | 2.50 | 5.74 | 0.09 | 0.01 | 0.00 | 0.41 | 0.02 | 0.00 | Leaves and Flowers | Pseudomonadaceae | Pseudomonas |
| ASV_50 | 1.03 | -1.31 | 0.82 | 0.74 | 0.22 | 0.23 | 1.38 | -6.05 | 3.63 | 0.17 | 0.00 | 0.00 | 0.56 | 0.00 | 0.00 | Leaves and Flowers | Pseudomonadaceae | Pseudomonas |
| ASV_505 | -0.36 | -0.72 | -0.11 | 0.74 | 0.20 | 0.20 | -0.49 | -3.66 | -0.53 | 0.63 | 0.00 | 0.60 | 1.00 | 0.00 | 0.70 | Leaves only | Sphingomonadaceae | Sphingomonas |
| ASV_51 | 1.56 | -1.18 | -1.23 | 0.67 | 0.22 | 0.23 | 2.33 | -5.36 | -5.28 | 0.02 | 0.00 | 0.00 | 0.22 | 0.00 | 0.00 | Leaves and Flowers | Rhizobiaceae | Allorhizobium. |
| ASV_517 | 0.54 | 0.07 | -0.05 | 0.76 | 0.20 | 0.20 | 0.72 | 0.37 | -0.25 | 0.48 | 0.71 | 0.80 | 0.93 | 0.84 | 0.89 | Not different | Acetobacteraceae | Unclassified |
| ASV_52 | -1.31 | -0.34 | 0.93 | 0.72 | 0.20 | 0.21 | -1.82 | -1.73 | 4.44 | 0.07 | 0.09 | 0.00 | 0.37 | 0.14 | 0.00 | Flowers only | Pseudomonadaceae | Pseudomonas |
| ASV_521 | -0.06 | -0.29 | 0.82 | 0.74 | 0.19 | 0.20 | -0.08 | -1.52 | 4.05 | 0.94 | 0.14 | 0.00 | 1.00 | 0.20 | 0.00 | Flowers only | Solirubrobacteraceae | Unclassified |
| ASV_523 | -1.15 | 0.53 | 0.71 | 0.77 | 0.19 | 0.20 | -1.49 | 2.73 | 3.49 | 0.15 | 0.01 | 0.00 | 0.53 | 0.02 | 0.00 | Leaves and Flowers | Erwiniaceae | Pantoea |
| ASV_528 | 1.51 | 0.35 | 1.16 | 0.77 | 0.19 | 0.20 | 1.97 | 1.86 | 5.78 | 0.06 | 0.08 | 0.00 | 0.36 | 0.13 | 0.00 | Flowers only | Unclassified | Unclassified |
| ASV_536 | -0.77 | 1.14 | 0.45 | 0.73 | 0.20 | 0.21 | -1.05 | 5.81 | 2.16 | 0.30 | 0.00 | 0.04 | 0.78 | 0.00 | 0.06 | Leaves only | Microbacteriaceae | Curtobacterium |
| ASV_54 | 0.02 | -0.42 | -0.19 | 0.65 | 0.23 | 0.24 | 0.03 | -1.85 | -0.77 | 0.98 | 0.07 | 0.44 | 1.00 | 0.11 | 0.55 | Not different | Oxalobacteraceae | Unclassified |
| ASV_543 | -0.07 | -0.98 | -0.07 | 0.76 | 0.21 | 0.21 | -0.09 | -4.79 | -0.36 | 0.93 | 0.00 | 0.73 | 1.00 | 0.00 | 0.82 | Leaves only | Oxalobacteraceae | Duganella |
| ASV_544 | 0.94 | -0.51 | -0.48 | 0.76 | 0.19 | 0.20 | 1.23 | -2.63 | -2.42 | 0.23 | 0.01 | 0.02 | 0.65 | 0.03 | 0.04 | Leaves and Flowers | Rhizobiaceae | Allorhizobium. |
| ASV_55 | 0.14 | 0.31 | 0.25 | 0.69 | 0.24 | 0.25 | 0.21 | 1.28 | 1.01 | 0.83 | 0.20 | 0.31 | 1.00 | 0.28 | 0.41 | Not different | Unclassified | Unclassified |
| ASV_550 | -0.40 | 1.12 | 0.81 | 0.75 | 0.19 | 0.19 | -0.53 | 5.91 | 4.17 | 0.60 | 0.00 | 0.00 | 1.00 | 0.00 | 0.00 | Leaves and Flowers | Comamonadaceae | Pelomonas |
| ASV_553 | -0.33 | 0.49 | 0.57 | 0.76 | 0.19 | 0.20 | -0.43 | 2.55 | 2.89 | 0.67 | 0.02 | 0.01 | 1.00 | 0.03 | 0.02 | Leaves and Flowers | Pseudomonadaceae | Pseudomonas |
| ASV_56 | NA | NA | NA | NA | NA | NA | NA | NA | NA | 1.00 | 1.00 | 1.00 | 1.00 | 1.00 | 1.00 | Not different | Unclassified | Unclassified |
| ASV_564 | 0.23 | -0.67 | -0.65 | 0.76 | 0.20 | 0.20 | 0.30 | -3.44 | -3.23 | 0.77 | 0.00 | 0.00 | 1.00 | 0.01 | 0.01 | Leaves and Flowers | Micromonosporaceae | Actinoplanes |
| ASV_565 | -0.11 | -0.66 | 0.14 | 0.75 | 0.19 | 0.20 | -0.15 | -3.42 | 0.72 | 0.88 | 0.00 | 0.48 | 1.00 | 0.00 | 0.58 | Leaves only | Rhizobiaceae | Allorhizobium. |
| ASV_57 | -0.60 | -0.54 | 1.32 | 0.67 | 0.28 | 0.30 | -0.89 | -1.90 | 4.44 | 0.38 | 0.06 | 0.00 | 0.85 | 0.10 | 0.00 | Flowers only | Pseudomonadaceae | Pseudomonas |
| ASV_572 | 0.51 | -1.76 | -0.75 | 0.75 | 0.19 | 0.20 | 0.68 | -9.17 | -3.73 | 0.50 | 0.00 | 0.00 | 0.93 | 0.00 | 0.00 | Leaves and Flowers | Xanthobacteraceae | Bradyrhizobium |
| ASV_573 | -0.11 | 0.05 | 0.13 | 0.74 | 0.19 | 0.20 | -0.15 | 0.25 | 0.66 | 0.88 | 0.81 | 0.51 | 1.00 | 0.93 | 0.61 | Not different | Nakamurellaceae | Nakamurella |
| ASV_575 | 0.31 | -0.72 | 0.08 | 0.77 | 0.20 | 0.20 | 0.40 | -3.66 | 0.39 | 0.69 | 0.00 | 0.70 | 1.00 | 0.01 | 0.80 | Leaves only | Comamonadaceae | Unclassified |
| ASV_59 | -1.25 | -1.17 | 1.58 | 0.74 | 0.19 | 0.21 | -1.68 | -6.11 | 7.41 | 0.10 | 0.00 | 0.00 | 0.43 | 0.00 | 0.00 | Leaves and Flowers | Pseudomonadaceae | Pseudomonas |
| ASV_6 | -1.02 | 0.19 | 1.49 | 0.53 | 0.24 | 0.25 | -1.92 | 0.77 | 5.95 | 0.06 | 0.44 | 0.00 | 0.36 | 0.56 | 0.00 | Flowers only | Pseudomonadaceae | Pseudomonas |
| ASV_60 | -1.41 | 0.33 | 0.51 | 0.69 | 0.25 | 0.26 | -2.05 | 1.32 | 1.96 | 0.04 | 0.19 | 0.05 | 0.30 | 0.27 | 0.08 | Not different | Erwiniaceae | Erwinia |
| ASV_61 | -1.55 | -0.16 | 1.57 | 0.66 | 0.21 | 0.22 | -2.36 | -0.76 | 7.05 | 0.02 | 0.45 | 0.00 | 0.22 | 0.57 | 0.00 | Flowers only | Pseudomonadaceae | Pseudomonas |

| | | | | | | | | | | | | | | | | | | |
|---|---|---|---|---|---|---|---|---|---|---|---|---|---|---|---|---|---|---|
| ASV_62 | -0.82 | 1.36 | 2.04 | 0.70 | 0.20 | 0.21 | -1.17 | 6.71 | 9.81 | 0.25 | 0.00 | 0.00 | 0.69 | 0.00 | 0.00 | Leaves and Flowers | Oxalobacteraceae | Duganella |
| ASV_63 | -2.19 | -0.87 | 1.66 | 0.74 | 0.20 | 0.23 | -2.95 | -4.27 | 7.34 | 0.00 | 0.00 | 0.00 | 0.13 | 0.00 | 0.00 | Leaves and Flowers | Pseudomonadaceae | Pseudomonas |
| ASV_634 | NA | NA | NA | NA | NA | NA | NA | NA | NA | 1.00 | 1.00 | 1.00 | 1.00 | 1.00 | 1.00 | Not different | Unclassified | Unclassified |
| ASV_636 | -0.36 | 1.06 | 0.78 | 0.77 | 0.20 | 0.20 | -0.47 | 5.41 | 3.92 | 0.64 | 0.00 | 0.00 | 1.00 | 0.00 | 0.00 | Leaves and Flowers | Oxalobacteraceae | Unclassified |
| ASV_641 | -2.14 | 1.38 | 0.36 | 0.77 | 0.20 | 0.20 | -2.77 | 6.82 | 1.79 | 0.01 | 0.00 | 0.09 | 0.18 | 0.00 | 0.14 | Leaves only | Rhizobiaceae | Candidatus Liberibacter |
| ASV_642 | -1.73 | -0.14 | 0.16 | 0.76 | 0.20 | 0.21 | -2.27 | -0.67 | 0.76 | 0.03 | 0.51 | 0.46 | 0.28 | 0.63 | 0.56 | Not different | Burkholderiaceae | Unclassified |
| ASV_65 | -0.78 | 1.71 | 1.00 | 0.61 | 0.21 | 0.21 | -1.28 | 8.24 | 4.85 | 0.20 | 0.00 | 0.00 | 0.62 | 0.00 | 0.00 | Leaves and Flowers | Burkholderiaceae | Burkholderia. |
| ASV_66 | -0.18 | -1.38 | -1.75 | 0.71 | 0.21 | 0.22 | -0.25 | -6.55 | -8.00 | 0.80 | 0.00 | 0.00 | 1.00 | 0.00 | 0.00 | Leaves and Flowers | Kineosporiaceae | Kineosporia |
| ASV_67 | -0.07 | -0.58 | 0.31 | 0.67 | 0.20 | 0.23 | -0.10 | -2.90 | 1.36 | 0.92 | 0.00 | 0.18 | 1.00 | 0.01 | 0.25 | Leaves only | Rhodanobacteraceae | Luteibacter |
| ASV_678 | -0.57 | -0.02 | -0.50 | 0.76 | 0.19 | 0.20 | -0.75 | -0.10 | -2.54 | 0.46 | 0.92 | 0.02 | 0.93 | 1.00 | 0.03 | Flowers only | Sphingobacteriaceae | Mucilaginibacter |
| ASV_69 | 0.90 | -1.16 | -1.50 | 0.66 | 0.20 | 0.22 | 1.36 | -5.73 | -6.96 | 0.18 | 0.00 | 0.00 | 0.56 | 0.00 | 0.00 | Leaves and Flowers | Microbacteriaceae | Galbitalea |
| ASV_7 | -0.20 | -1.08 | 1.08 | 0.54 | 0.26 | 0.25 | -0.38 | -4.23 | 4.36 | 0.71 | 0.00 | 0.00 | 1.00 | 0.00 | 0.00 | Leaves and Flowers | Pseudomonadaceae | Pseudomonas |
| ASV_706 | -0.06 | -0.50 | 0.54 | 0.74 | 0.20 | 0.21 | -0.08 | -2.43 | 2.59 | 0.93 | 0.02 | 0.01 | 1.00 | 0.03 | 0.02 | Leaves and Flowers | Comamonadaceae | Polaromonas |
| ASV_71 | 0.01 | 0.72 | 0.11 | 0.66 | 0.19 | 0.19 | 0.01 | 3.89 | 0.57 | 0.99 | 0.00 | 0.57 | 1.00 | 0.00 | 0.67 | Leaves only | Oxalobacteraceae | Unclassified |
| ASV_710 | 0.28 | 0.61 | 0.63 | 0.70 | 0.19 | 0.20 | 0.40 | 3.15 | 3.10 | 0.69 | 0.00 | 0.00 | 1.00 | 0.01 | 0.01 | Leaves and Flowers | Oxalobacteraceae | Janthinobacterium |
| ASV_712 | -0.16 | -0.16 | 0.91 | 0.76 | 0.19 | 0.20 | -0.21 | -0.80 | 4.54 | 0.83 | 0.43 | 0.00 | 1.00 | 0.56 | 0.00 | Flowers only | Mycobacteriaceae | Mycobacterium |
| ASV_73 | -0.60 | 0.94 | 2.10 | 0.69 | 0.19 | 0.22 | -0.86 | 4.91 | 9.71 | 0.39 | 0.00 | 0.00 | 0.87 | 0.00 | 0.00 | Leaves and Flowers | Pseudomonadaceae | Pseudomonas |
| ASV_74 | -3.64 | -0.70 | -2.15 | 0.76 | 0.20 | 0.20 | -4.80 | -3.52 | -10.65 | 0.00 | 0.00 | 0.00 | 0.01 | 0.00 | 0.00 | Leaves and Flowers | Oxalobacteraceae | Duganella |
| ASV_75 | -0.44 | 1.23 | 0.16 | 0.63 | 0.20 | 0.19 | -0.71 | 6.26 | 0.82 | 0.48 | 0.00 | 0.41 | 0.93 | 0.00 | 0.52 | Leaves only | Unclassified | Unclassified |
| ASV_751 | 0.01 | 0.51 | -0.59 | 0.76 | 0.19 | 0.19 | 0.01 | 2.60 | -3.04 | 0.99 | 0.02 | 0.01 | 1.00 | 0.03 | 0.01 | Leaves and Flowers | Staphylococcaceae | Staphylococcus |
| ASV_755 | 0.40 | 0.25 | 0.53 | 0.77 | 0.19 | 0.19 | 0.52 | 1.29 | 2.73 | 0.61 | 0.22 | 0.02 | 1.00 | 0.30 | 0.03 | Flowers only | Rhizobiaceae | Allorhizobium. |
| ASV_77 | -0.03 | -1.96 | -1.74 | 0.72 | 0.21 | 0.21 | -0.04 | -9.55 | -8.19 | 0.97 | 0.00 | 0.00 | 1.00 | 0.00 | 0.00 | Leaves and Flowers | Cryptosporangiaceae | Cryptosporangium |
| ASV_78 | NA | NA | NA | NA | NA | NA | NA | NA | NA | 1.00 | 1.00 | 1.00 | 1.00 | 1.00 | 1.00 | Not different | Unclassified | Unclassified |
| ASV_785 | 0.11 | 0.08 | 0.43 | 0.78 | 0.20 | 0.21 | 0.14 | 0.42 | 2.07 | 0.89 | 0.68 | 0.05 | 1.00 | 0.82 | 0.09 | Not different | Pseudomonadaceae | Pseudomonas |
| ASV_79 | -0.46 | 0.35 | 0.50 | 0.71 | 0.19 | 0.20 | -0.66 | 1.82 | 2.47 | 0.51 | 0.07 | 0.02 | 0.94 | 0.12 | 0.03 | Flowers only | Oxalobacteraceae | Janthinobacterium |
| ASV_8 | -0.03 | -0.38 | 1.32 | 0.59 | 0.23 | 0.26 | -0.06 | -1.62 | 5.07 | 0.96 | 0.11 | 0.00 | 1.00 | 0.17 | 0.00 | Flowers only | Pseudomonadaceae | Pseudomonas |
| ASV_80 | -0.49 | -1.41 | 0.44 | 0.73 | 0.20 | 0.22 | -0.67 | -7.08 | 2.05 | 0.51 | 0.00 | 0.05 | 0.93 | 0.00 | 0.07 | Leaves only | Pseudomonadaceae | Pseudomonas |
| ASV_83 | -0.57 | 0.25 | 0.81 | 0.73 | 0.21 | 0.22 | -0.79 | 1.19 | 3.70 | 0.43 | 0.24 | 0.00 | 0.91 | 0.33 | 0.00 | Flowers only | Oxalobacteraceae | Unclassified |
| ASV_84 | 0.27 | -2.00 | -0.06 | 0.73 | 0.21 | 0.21 | 0.37 | -9.49 | -0.29 | 0.71 | 0.00 | 0.77 | 1.00 | 0.00 | 0.86 | Leaves only | Kineosporiaceae | Kineosporia |
| ASV_85 | 0.15 | 0.30 | 0.61 | 0.70 | 0.20 | 0.20 | 0.21 | 1.51 | 3.02 | 0.83 | 0.13 | 0.00 | 1.00 | 0.20 | 0.01 | Flowers only | Oxalobacteraceae | Unclassified |
| ASV_87 | NA | NA | NA | NA | NA | NA | NA | NA | NA | 1.00 | 1.00 | 1.00 | 1.00 | 1.00 | 1.00 | Not different | Unclassified | Unclassified |
| ASV_879 | 0.82 | 1.27 | -0.23 | 0.77 | 0.20 | 0.20 | 1.07 | 6.40 | -1.11 | 0.30 | 0.00 | 0.28 | 0.78 | 0.00 | 0.38 | Leaves only | Micrococcaceae | Arthrobacter |
| ASV_883 | 0.22 | -0.29 | 0.20 | 0.77 | 0.19 | 0.20 | 0.29 | -1.46 | 0.99 | 0.78 | 0.16 | 0.34 | 1.00 | 0.24 | 0.44 | Not different | Nakamurellaceae | Nakamurella |
| ASV_9 | -0.14 | -0.93 | -0.11 | 0.63 | 0.25 | 0.27 | -0.23 | -3.66 | -0.42 | 0.82 | 0.00 | 0.67 | 1.00 | 0.00 | 0.77 | Leaves only | Rhizobiaceae | Allorhizobium. |
| ASV_92 | 1.10 | 0.66 | 0.94 | 0.70 | 0.23 | 0.24 | 1.57 | 2.85 | 3.86 | 0.12 | 0.01 | 0.00 | 0.48 | 0.01 | 0.00 | Leaves and Flowers | Unclassified | Unclassified |
| ASV_93 | 0.68 | -0.39 | -0.28 | 0.72 | 0.22 | 0.22 | 0.94 | -1.73 | -1.24 | 0.35 | 0.09 | 0.22 | 0.84 | 0.14 | 0.30 | Not different | Flavobacteriaceae | Flavobacterium |
| ASV_940 | 1.00 | -1.20 | -0.60 | 0.72 | 0.21 | 0.20 | 1.39 | -5.68 | -2.96 | 0.17 | 0.00 | 0.00 | 0.56 | 0.00 | 0.01 | Leaves and Flowers | Oxalobacteraceae | Massilia |
| ASV_941 | 0.20 | -0.82 | 0.00 | 0.76 | 0.19 | 0.20 | 0.26 | -4.26 | 0.02 | 0.80 | 0.00 | 0.99 | 1.00 | 0.00 | 1.00 | Leaves only | Solirubrobacteraceae | Unclassified |
| ASV_950 | -0.29 | 0.66 | 0.62 | 0.76 | 0.19 | 0.20 | -0.37 | 3.47 | 3.13 | 0.71 | 0.00 | 0.01 | 1.00 | 0.01 | 0.01 | Leaves and Flowers | Mycobacteriaceae | Mycobacterium |
| ASV_97 | -1.35 | 1.15 | 1.32 | 0.71 | 0.19 | 0.20 | -1.89 | 6.03 | 6.70 | 0.06 | 0.00 | 0.00 | 0.36 | 0.00 | 0.00 | Leaves and Flowers | Oxalobacteraceae | Massilia |
| ASV_99 | 0.08 | 0.92 | 1.17 | 0.72 | 0.22 | 0.23 | 0.12 | 4.15 | 5.02 | 0.91 | 0.00 | 0.00 | 1.00 | 0.00 | 0.00 | Leaves and Flowers | Yersiniaceae | Rahnella1 |

**B**

| ASV | lfc_ INT | lfc_ L | lfc_ F | se_ INT | se_ L | se_ F | W_ INT | W_ L | W_ F | p_ INT | p_ L | p_ F | q_ INT | q_ L | q_ F | Tissue_ diff_abund | Family | Genus |
|---|---|---|---|---|---|---|---|---|---|---|---|---|---|---|---|---|---|---|
| ASV_1 | -0.45 | 3.17 | -0.59 | 0.40 | 0.34 | 0.31 | -1.10 | 9.31 | -1.92 | 0.27 | 0.00 | 0.06 | 0.43 | 0.00 | 0.10 | Leaves only | Enterobacteriaceae | Escherichia-Shigella |
| ASV_10 | -1.16 | 1.83 | -0.17 | 0.37 | 0.28 | 0.26 | -3.15 | 6.50 | -0.68 | 0.00 | 0.00 | 0.50 | 0.01 | 0.00 | 0.61 | Leaves only | Enterobacteriaceae | Escherichia-Shigella |
| ASV_100 | -0.02 | -0.55 | -0.58 | 0.39 | 0.31 | 0.26 | -0.04 | -1.79 | -2.19 | 0.97 | 0.09 | 0.04 | 1.00 | 0.15 | 0.07 | Not different | Flavobacteriaceae | Flavobacterium |
| ASV_102 | -2.75 | 0.65 | 2.69 | 0.39 | 0.32 | 0.33 | -7.05 | 2.01 | 8.10 | 0.00 | 0.05 | 0.00 | 0.00 | 0.09 | 0.00 | Flowers only | Pseudomonadaceae | Pseudomonas |
| ASV_104 | -1.77 | 0.01 | 1.32 | 0.40 | 0.29 | 0.31 | -4.39 | 0.05 | 4.29 | 0.00 | 0.96 | 0.00 | 0.00 | 1.00 | 0.00 | Flowers only | Pseudomonadaceae | Pseudomonas |
| ASV_106 | -0.73 | 0.07 | 0.84 | 0.39 | 0.35 | 0.30 | -1.85 | 0.20 | 2.81 | 0.07 | 0.84 | 0.01 | 0.14 | 0.93 | 0.01 | Flowers only | Pseudomonadaceae | Pseudomonas |
| ASV_11 | -2.63 | 0.31 | 2.11 | 0.36 | 0.29 | 0.31 | -7.25 | 1.08 | 6.81 | 0.00 | 0.28 | 0.00 | 0.00 | 0.39 | 0.00 | Flowers only | Pseudomonadaceae | Pseudomonas |
| ASV_110 | 0.20 | 0.35 | 0.04 | 0.42 | 0.31 | 0.30 | 0.48 | 1.14 | 0.12 | 0.64 | 0.26 | 0.90 | 0.78 | 0.37 | 0.97 | Not different | Oxalobacteraceae | Unclassified |
| ASV_113 | -0.43 | 0.77 | 0.99 | 0.37 | 0.27 | 0.26 | -1.17 | 2.91 | 3.76 | 0.25 | 0.01 | 0.00 | 0.41 | 0.02 | 0.00 | Leaves and Flowers | Oxalobacteraceae | Unclassified |
| ASV_1140 | 0.90 | -0.71 | -0.36 | 0.40 | 0.29 | 0.29 | 2.27 | -2.45 | -1.24 | 0.03 | 0.02 | 0.22 | 0.08 | 0.04 | 0.29 | Leaves only | Unclassified | Unclassified |
| ASV_115 | 0.72 | -0.69 | -1.44 | 0.39 | 0.31 | 0.27 | 1.83 | -2.23 | -5.31 | 0.07 | 0.03 | 0.00 | 0.15 | 0.06 | 0.00 | Flowers only | Comamonadaceae | Variovorax |
| ASV_1157 | -0.89 | 1.50 | 1.25 | 0.38 | 0.27 | 0.29 | -2.33 | 5.53 | 4.29 | 0.03 | 0.00 | 0.00 | 0.08 | 0.00 | 0.00 | Leaves and Flowers | Unclassified | Unclassified |
| ASV_117 | -0.30 | 0.21 | -1.48 | 0.39 | 0.30 | 0.27 | -0.77 | 0.72 | -5.43 | 0.45 | 0.48 | 0.00 | 0.61 | 0.61 | 0.00 | Flowers only | Microbacteriaceae | Galbitalea |
| ASV_118 | -4.08 | 0.16 | 1.99 | 0.36 | 0.27 | 0.28 | -11.29 | 0.57 | 7.02 | 0.00 | 0.57 | 0.00 | 0.00 | 0.67 | 0.00 | Flowers only | Pseudomonadaceae | Pseudomonas |
| ASV_12 | 0.83 | -0.41 | -1.42 | 0.49 | 0.34 | 0.33 | 1.69 | -1.19 | -4.25 | 0.10 | 0.24 | 0.00 | 0.19 | 0.35 | 0.00 | Flowers only | Rhizobiaceae | Allorhizobium. |
| ASV_121 | 0.09 | -0.14 | -0.57 | 0.50 | 0.33 | 0.33 | 0.19 | -0.41 | -1.71 | 0.85 | 0.68 | 0.10 | 0.97 | 0.79 | 0.14 | Not different | Comamonadaceae | Variovorax |
| ASV_123 | 0.21 | 1.08 | 0.13 | 0.40 | 0.29 | 0.28 | 0.53 | 3.72 | 0.48 | 0.60 | 0.00 | 0.64 | 0.76 | 0.00 | 0.72 | Leaves only | Flavobacteriaceae | Flavobacterium |
| ASV_124 | -1.31 | -0.04 | 1.19 | 0.36 | 0.28 | 0.30 | -3.60 | -0.15 | 3.96 | 0.00 | 0.88 | 0.00 | 0.00 | 0.96 | 0.00 | Flowers only | Unclassified | Unclassified |
| ASV_125 | 0.05 | 0.49 | 0.10 | 0.37 | 0.28 | 0.28 | 0.13 | 1.77 | 0.38 | 0.90 | 0.09 | 0.71 | 1.00 | 0.15 | 0.79 | Not different | Oxalobacteraceae | Janthinobacterium |
| ASV_13 | -1.60 | 1.47 | 2.02 | 0.38 | 0.29 | 0.33 | -4.16 | 5.12 | 6.06 | 0.00 | 0.00 | 0.00 | 0.00 | 0.00 | 0.00 | Leaves and Flowers | Oxalobacteraceae | Duganella |
| ASV_132 | 0.72 | 0.86 | -0.23 | 0.41 | 0.28 | 0.29 | 1.77 | 3.05 | -0.78 | 0.10 | 0.01 | 0.45 | 0.19 | 0.02 | 0.55 | Leaves only | Oxalobacteraceae | Duganella |
| ASV_134 | 0.76 | -0.80 | 0.46 | 0.40 | 0.29 | 0.28 | 1.88 | -2.78 | 1.66 | 0.08 | 0.01 | 0.11 | 0.15 | 0.03 | 0.17 | Leaves only | Oxalobacteraceae | Unclassified |
| ASV_135 | -1.08 | 1.01 | -0.41 | 0.35 | 0.28 | 0.26 | -3.06 | 3.56 | -1.54 | 0.00 | 0.00 | 0.13 | 0.02 | 0.00 | 0.19 | Leaves only | Microbacteriaceae | Frondihabitans |
| ASV_136 | -0.21 | 0.73 | 1.00 | 0.37 | 0.26 | 0.28 | -0.56 | 2.81 | 3.61 | 0.58 | 0.01 | 0.00 | 0.74 | 0.02 | 0.00 | Leaves and Flowers | Erwiniaceae | Unclassified |
| ASV_1376 | 0.93 | -1.76 | -2.22 | 0.39 | 0.28 | 0.27 | 2.39 | -6.37 | -8.19 | 0.03 | 0.00 | 0.00 | 0.08 | 0.00 | 0.00 | Leaves and Flowers | Oxalobacteraceae | Unclassified |
| ASV_138 | 0.80 | -1.35 | -0.16 | 0.38 | 0.28 | 0.30 | 2.11 | -4.74 | -0.53 | 0.05 | 0.00 | 0.60 | 0.10 | 0.00 | 0.70 | Leaves only | Pseudomonadaceae | Pseudomonas |
| ASV_14 | -1.55 | 2.95 | 0.13 | 0.39 | 0.29 | 0.27 | -3.97 | 10.24 | 0.49 | 0.00 | 0.00 | 0.63 | 0.00 | 0.00 | 0.71 | Leaves only | Enterobacteriaceae | Unclassified |
| ASV_141 | 0.23 | 0.39 | 0.63 | 0.40 | 0.28 | 0.27 | 0.58 | 1.39 | 2.31 | 0.57 | 0.17 | 0.03 | 0.73 | 0.26 | 0.05 | Not different | Oxalobacteraceae | Unclassified |
| ASV_1416 | 0.10 | -1.24 | -0.40 | 0.38 | 0.28 | 0.27 | 0.27 | -4.49 | -1.48 | 0.79 | 0.00 | 0.16 | 0.93 | 0.00 | 0.22 | Leaves only | Unclassified | Unclassified |
| ASV_144 | 0.96 | -1.27 | -1.80 | 0.42 | 0.31 | 0.29 | 2.27 | -4.09 | -6.14 | 0.03 | 0.00 | 0.00 | 0.08 | 0.00 | 0.00 | Leaves and Flowers | Xanthomonadaceae | Pseudoxanthomonas |
| ASV_145 | NA | NA | NA | NA | NA | NA | NA | NA | NA | 1.00 | 1.00 | 1.00 | 1.00 | 1.00 | 1.00 | Not different | Unclassified | Unclassified |
| ASV_147 | 0.04 | 0.34 | -0.06 | 0.40 | 0.30 | 0.29 | 0.10 | 1.15 | -0.20 | 0.92 | 0.26 | 0.84 | 1.00 | 0.37 | 0.92 | Not different | Oxalobacteraceae | Duganella |
| ASV_148 | -0.74 | -0.12 | 0.27 | 0.38 | 0.28 | 0.30 | -1.96 | -0.42 | 0.91 | 0.06 | 0.68 | 0.37 | 0.12 | 0.79 | 0.46 | Not different | Pseudomonadaceae | Pseudomonas |
| ASV_150 | -2.26 | 1.06 | 1.10 | 0.38 | 0.29 | 0.28 | -6.02 | 3.69 | 3.89 | 0.00 | 0.00 | 0.00 | 0.00 | 0.00 | 0.00 | Leaves and Flowers | Unclassified | Unclassified |
| ASV_152 | -0.25 | 0.19 | -0.73 | 0.39 | 0.29 | 0.28 | -0.65 | 0.66 | -2.59 | 0.52 | 0.51 | 0.01 | 0.70 | 0.62 | 0.03 | Flowers only | Sphingobacteriaceae | Mucilaginibacter |
| ASV_157 | -1.48 | 1.41 | 1.54 | 0.40 | 0.29 | 0.29 | -3.73 | 4.82 | 5.28 | 0.00 | 0.00 | 0.00 | 0.01 | 0.00 | 0.00 | Leaves and Flowers | Yersiniaceae | Rahnella1 |
| ASV_16 | -2.40 | 1.70 | 2.47 | 0.39 | 0.31 | 0.31 | -6.09 | 5.46 | 7.93 | 0.00 | 0.00 | 0.00 | 0.00 | 0.00 | 0.00 | Leaves and Flowers | Oxalobacteraceae | Duganella |
| ASV_162 | -0.20 | 0.81 | 0.09 | 0.37 | 0.27 | 0.26 | -0.54 | 3.04 | 0.34 | 0.60 | 0.01 | 0.74 | 0.76 | 0.02 | 0.82 | Leaves only | Oxalobacteraceae | Unclassified |
| ASV_169 | -0.19 | -0.22 | -0.25 | 0.38 | 0.27 | 0.28 | -0.52 | -0.80 | -0.89 | 0.61 | 0.43 | 0.38 | 0.76 | 0.56 | 0.47 | Not different | Flavobacteriaceae | Flavobacterium |
| ASV_17 | -0.64 | 0.85 | 0.82 | 0.44 | 0.35 | 0.41 | -1.48 | 2.43 | 2.01 | 0.14 | 0.02 | 0.05 | 0.26 | 0.04 | 0.08 | Leaves only | Erwiniaceae | Erwinia |
| ASV_172 | -0.47 | 0.69 | 0.13 | 0.36 | 0.26 | 0.25 | -1.30 | 2.68 | 0.50 | 0.21 | 0.01 | 0.62 | 0.34 | 0.03 | 0.71 | Leaves only | Oxalobacteraceae | Unclassified |
| ASV_177 | NA | NA | NA | NA | NA | NA | NA | NA | NA | 1.00 | 1.00 | 1.00 | 1.00 | 1.00 | 1.00 | Not different | Unclassified | Unclassified |

| | | | | | | | | | | | | | | | | | | |
|---|---|---|---|---|---|---|---|---|---|---|---|---|---|---|---|---|---|---|
| ASV_179 | NA | NA | NA | NA | NA | NA | NA | NA | NA | 1.00 | 1.00 | 1.00 | 1.00 | 1.00 | 1.00 | Not different | Unclassified | Unclassified |
| ASV_183 | -0.97 | 1.28 | 1.00 | 0.38 | 0.28 | 0.33 | -2.53 | 4.56 | 3.05 | 0.02 | 0.00 | 0.00 | 0.05 | 0.00 | 0.01 | Leaves and Flowers | Pseudomonadaceae | Pseudomonas |
| ASV_186 | -0.09 | 0.17 | 0.82 | 0.36 | 0.26 | 0.28 | -0.24 | 0.64 | 2.99 | 0.81 | 0.52 | 0.00 | 0.93 | 0.63 | 0.01 | Flowers only | Erwiniaceae | Pantoea |
| ASV_188 | -1.22 | 1.04 | 0.47 | 0.37 | 0.27 | 0.26 | -3.31 | 3.90 | 1.77 | 0.00 | 0.00 | 0.09 | 0.01 | 0.00 | 0.14 | Leaves only | Pseudomonadaceae | Pseudomonas |
| ASV_189 | 0.80 | -1.24 | -1.37 | 0.38 | 0.27 | 0.27 | 2.11 | -4.52 | -5.03 | 0.04 | 0.00 | 0.00 | 0.10 | 0.00 | 0.00 | Leaves and Flowers | Caulobacteraceae | Caulobacter |
| ASV_19 | 0.00 | 1.79 | -0.35 | 0.42 | 0.34 | 0.31 | 0.01 | 5.26 | -1.15 | 0.99 | 0.00 | 0.25 | 1.00 | 0.00 | 0.33 | Leaves only | Sphingomonadaceae | Sphingomonas |
| ASV_195 | 0.17 | 0.28 | 0.60 | 0.41 | 0.29 | 0.32 | 0.41 | 0.95 | 1.89 | 0.68 | 0.35 | 0.07 | 0.82 | 0.47 | 0.11 | Not different | Pseudomonadaceae | Pseudomonas |
| ASV_2 | -4.39 | 1.40 | 3.11 | 0.40 | 0.32 | 0.32 | -11.07 | 4.32 | 9.67 | 0.00 | 0.00 | 0.00 | 0.00 | 0.00 | 0.00 | Leaves and Flowers | Pseudomonadaceae | Pseudomonas |
| ASV_20 | -3.98 | 1.12 | 2.99 | 0.36 | 0.30 | 0.30 | -10.94 | 3.78 | 10.04 | 0.00 | 0.00 | 0.00 | 0.00 | 0.00 | 0.00 | Leaves and Flowers | Pseudomonadaceae | Pseudomonas |
| ASV_201 | -0.26 | -0.48 | -1.07 | 0.40 | 0.30 | 0.30 | -0.64 | -1.59 | -3.56 | 0.53 | 0.14 | 0.00 | 0.70 | 0.21 | 0.01 | Flowers only | Flavobacteriaceae | Flavobacterium |
| ASV_204 | 0.30 | 0.10 | -0.73 | 0.41 | 0.30 | 0.29 | 0.73 | 0.32 | -2.54 | 0.47 | 0.75 | 0.02 | 0.64 | 0.84 | 0.03 | Flowers only | Flavobacteriaceae | Flavobacterium |
| ASV_209 | -0.10 | -0.20 | 0.16 | 0.40 | 0.28 | 0.31 | -0.26 | -0.70 | 0.50 | 0.80 | 0.49 | 0.62 | 0.93 | 0.61 | 0.71 | Not different | Pseudomonadaceae | Pseudomonas |
| ASV_21 | -2.09 | 0.79 | 2.13 | 0.37 | 0.31 | 0.30 | -5.65 | 2.51 | 7.05 | 0.00 | 0.01 | 0.00 | 0.00 | 0.03 | 0.00 | Leaves and Flowers | Pseudomonadaceae | Pseudomonas |
| ASV_210 | 0.40 | -0.71 | -1.84 | 0.38 | 0.28 | 0.27 | 1.04 | -2.54 | -6.85 | 0.31 | 0.02 | 0.00 | 0.46 | 0.03 | 0.00 | Leaves and Flowers | Xanthobacteraceae | Tardiphaga |
| ASV_212 | NA | NA | NA | NA | NA | NA | NA | NA | NA | 1.00 | 1.00 | 1.00 | 1.00 | 1.00 | 1.00 | Not different | Unclassified | Unclassified |
| ASV_218 | 0.47 | -0.84 | -2.18 | 0.37 | 0.27 | 0.26 | 1.28 | -3.10 | -8.33 | 0.21 | 0.00 | 0.00 | 0.34 | 0.01 | 0.00 | Leaves and Flowers | Xanthobacteraceae | Unclassified |
| ASV_22 | -0.20 | -0.43 | 0.82 | 0.41 | 0.41 | 0.39 | -0.50 | -1.05 | 2.10 | 0.62 | 0.30 | 0.04 | 0.77 | 0.41 | 0.07 | Not different | Pseudomonadaceae | Pseudomonas |
| ASV_220 | -0.69 | 0.93 | 0.85 | 0.38 | 0.27 | 0.27 | -1.81 | 3.42 | 3.15 | 0.08 | 0.00 | 0.00 | 0.16 | 0.01 | 0.01 | Leaves and Flowers | Oxalobacteraceae | Unclassified |
| ASV_225 | -0.40 | -0.11 | 1.12 | 0.44 | 0.31 | 0.31 | -0.90 | -0.36 | 3.61 | 0.38 | 0.73 | 0.00 | 0.55 | 0.82 | 0.01 | Flowers only | Pseudomonadaceae | Pseudomonas |
| ASV_23 | -3.06 | 1.05 | 2.10 | 0.38 | 0.31 | 0.32 | -7.99 | 3.40 | 6.49 | 0.00 | 0.00 | 0.00 | 0.00 | 0.00 | 0.00 | Leaves and Flowers | Pseudomonadaceae | Pseudomonas |
| ASV_239 | 0.06 | 0.68 | -0.04 | 0.38 | 0.31 | 0.28 | 0.15 | 2.24 | -0.16 | 0.88 | 0.03 | 0.87 | 1.00 | 0.06 | 0.95 | Not different | Cellulomonadaceae | Cellulomonas |
| ASV_24 | -2.25 | 1.50 | 2.88 | 0.41 | 0.34 | 0.38 | -5.44 | 4.37 | 7.49 | 0.00 | 0.00 | 0.00 | 0.00 | 0.00 | 0.00 | Leaves and Flowers | Oxalobacteraceae | Duganella |
| ASV_242 | 0.33 | 0.07 | 0.41 | 0.40 | 0.27 | 0.27 | 0.83 | 0.24 | 1.49 | 0.42 | 0.81 | 0.15 | 0.59 | 0.90 | 0.22 | Not different | Oxalobacteraceae | Unclassified |
| ASV_244 | NA | NA | NA | NA | NA | NA | NA | NA | NA | 1.00 | 1.00 | 1.00 | 1.00 | 1.00 | 1.00 | Not different | Unclassified | Unclassified |
| ASV_246 | -0.07 | 0.51 | 0.48 | 0.41 | 0.32 | 0.31 | -0.18 | 1.60 | 1.57 | 0.86 | 0.12 | 0.13 | 0.98 | 0.19 | 0.18 | Not different | Flavobacteriaceae | Flavobacterium |
| ASV_254 | -0.74 | 0.44 | -0.16 | 0.38 | 0.28 | 0.28 | -1.93 | 1.57 | -0.57 | 0.07 | 0.13 | 0.57 | 0.14 | 0.20 | 0.68 | Not different | Flavobacteriaceae | Flavobacterium |
| ASV_256 | -0.46 | -0.53 | 0.55 | 0.39 | 0.28 | 0.28 | -1.19 | -1.86 | 1.96 | 0.24 | 0.07 | 0.06 | 0.39 | 0.12 | 0.09 | Not different | Unclassified | Unclassified |
| ASV_263 | 0.88 | -0.19 | -0.58 | 0.41 | 0.28 | 0.28 | 2.16 | -0.69 | -2.06 | 0.04 | 0.50 | 0.05 | 0.09 | 0.61 | 0.08 | Not different | Sphingobacteriaceae | Pedobacter |
| ASV_267 | -0.86 | 0.58 | 1.91 | 0.39 | 0.29 | 0.29 | -2.23 | 2.00 | 6.50 | 0.03 | 0.05 | 0.00 | 0.08 | 0.10 | 0.00 | Flowers only | Unclassified | Unclassified |
| ASV_27 | -1.67 | 3.05 | -0.57 | 0.40 | 0.32 | 0.34 | -4.21 | 9.61 | -1.70 | 0.00 | 0.00 | 0.10 | 0.00 | 0.00 | 0.15 | Leaves only | Oxalobacteraceae | Janthinobacterium |
| ASV_272 | NA | NA | NA | NA | NA | NA | NA | NA | NA | 1.00 | 1.00 | 1.00 | 1.00 | 1.00 | 1.00 | Not different | Unclassified | Unclassified |
| ASV_28 | -1.03 | 1.57 | 0.58 | 0.36 | 0.27 | 0.27 | -2.86 | 5.89 | 2.18 | 0.01 | 0.00 | 0.03 | 0.02 | 0.00 | 0.06 | Leaves only | Oxalobacteraceae | Janthinobacterium |
| ASV_284 | 0.37 | -2.00 | -0.86 | 0.38 | 0.28 | 0.27 | 0.97 | -7.22 | -3.22 | 0.34 | 0.00 | 0.00 | 0.50 | 0.00 | 0.01 | Leaves and Flowers | Microbacteriaceae | Herbiconiux |
| ASV_29 | -4.90 | 3.12 | 4.22 | 0.39 | 0.30 | 0.32 | -12.69 | 10.47 | 13.31 | 0.00 | 0.00 | 0.00 | 0.00 | 0.00 | 0.00 | Leaves and Flowers | Pseudomonadaceae | Pseudomonas |
| ASV_294 | -0.94 | -0.21 | -1.47 | 0.39 | 0.30 | 0.30 | -2.43 | -0.69 | -4.98 | 0.03 | 0.50 | 0.00 | 0.08 | 0.61 | 0.00 | Flowers only | Oxalobacteraceae | Unclassified |
| ASV_295 | -0.55 | 1.33 | -0.74 | 0.38 | 0.28 | 0.26 | -1.43 | 4.80 | -2.81 | 0.18 | 0.00 | 0.02 | 0.32 | 0.00 | 0.03 | Leaves and Flowers | Sphingobacteriaceae | Pedobacter |
| ASV_297 | NA | NA | NA | NA | NA | NA | NA | NA | NA | 1.00 | 1.00 | 1.00 | 1.00 | 1.00 | 1.00 | Not different | Unclassified | Unclassified |
| ASV_298 | -0.98 | -0.50 | 0.67 | 0.38 | 0.27 | 0.30 | -2.60 | -1.88 | 2.21 | 0.02 | 0.07 | 0.04 | 0.05 | 0.13 | 0.07 | Not different | Pseudomonadaceae | Pseudomonas |
| ASV_3 | -0.99 | 2.57 | 0.34 | 0.35 | 0.29 | 0.27 | -2.85 | 9.01 | 1.25 | 0.01 | 0.00 | 0.22 | 0.02 | 0.00 | 0.29 | Leaves only | Oxalobacteraceae | Janthinobacterium |
| ASV_30 | -0.88 | 1.10 | 1.67 | 0.38 | 0.28 | 0.32 | -2.31 | 3.94 | 5.26 | 0.02 | 0.00 | 0.00 | 0.07 | 0.00 | 0.00 | Leaves and Flowers | Oxalobacteraceae | Duganella |
| ASV_304 | 0.41 | -0.91 | 0.49 | 0.38 | 0.29 | 0.30 | 1.08 | -3.11 | 1.65 | 0.29 | 0.00 | 0.11 | 0.45 | 0.01 | 0.16 | Leaves only | Unclassified | Unclassified |
| ASV_306 | -0.25 | 0.50 | 0.39 | 0.39 | 0.27 | 0.27 | -0.66 | 1.84 | 1.45 | 0.53 | 0.10 | 0.19 | 0.70 | 0.17 | 0.25 | Not different | Oxalobacteraceae | Janthinobacterium |
| ASV_31 | -1.42 | 0.89 | 2.21 | 0.37 | 0.33 | 0.29 | -3.87 | 2.72 | 7.53 | 0.00 | 0.01 | 0.00 | 0.00 | 0.02 | 0.00 | Leaves and Flowers | Pseudomonadaceae | Pseudomonas |
| ASV_313 | 0.81 | -1.10 | -1.52 | 0.39 | 0.27 | 0.27 | 2.08 | -4.08 | -5.66 | 0.05 | 0.00 | 0.00 | 0.10 | 0.00 | 0.00 | Leaves and Flowers | Rhizobiaceae | Allorhizobium. |
| ASV_32 | 0.18 | -0.54 | -1.71 | 0.38 | 0.29 | 0.28 | 0.48 | -1.89 | -6.11 | 0.63 | 0.07 | 0.00 | 0.78 | 0.12 | 0.00 | Flowers only | Rhizobiaceae | Allorhizobium. |
| ASV_324 | 0.57 | 0.24 | -0.61 | 0.38 | 0.27 | 0.26 | 1.49 | 0.88 | -2.33 | 0.15 | 0.39 | 0.03 | 0.27 | 0.51 | 0.05 | Not different | Sphingobacteriaceae | Pedobacter |
| ASV_325 | -1.89 | 1.42 | 1.48 | 0.36 | 0.26 | 0.26 | -5.17 | 5.37 | 5.64 | 0.00 | 0.00 | 0.00 | 0.00 | 0.00 | 0.00 | Leaves and Flowers | Sphingomonadaceae | Unclassified |
| ASV_327 | 0.24 | -1.24 | -0.26 | 0.38 | 0.27 | 0.28 | 0.63 | -4.57 | -0.93 | 0.54 | 0.00 | 0.37 | 0.71 | 0.00 | 0.46 | Leaves only | Flavobacteriaceae | Flavobacterium |

| | | | | | | | | | | | | | | | | | |
|---|---|---|---|---|---|---|---|---|---|---|---|---|---|---|---|---|---|
| ASV_331 | 0.79 | -1.25 | -0.94 | 0.37 | 0.27 | 0.26 | 2.14 | -4.67 | -3.54 | 0.04 | 0.00 | 0.00 | 0.09 | 0.00 | 0.00 | Leaves and Flowers | Mycobacteriaceae | Mycobacterium |
| ASV_338 | 0.01 | -2.40 | -0.70 | 0.38 | 0.28 | 0.26 | 0.02 | -8.66 | -2.65 | 0.99 | 0.00 | 0.01 | 1.00 | 0.00 | 0.03 | Leaves and Flowers | Unclassified | Unclassified |
| ASV_339 | NA | NA | NA | NA | NA | NA | NA | NA | NA | 1.00 | 1.00 | 1.00 | 1.00 | 1.00 | 1.00 | Not different | Unclassified | Unclassified |
| ASV_34 | -1.66 | 2.29 | 1.76 | 0.36 | 0.28 | 0.27 | -4.56 | 8.20 | 6.40 | 0.00 | 0.00 | 0.00 | 0.00 | 0.00 | 0.00 | Leaves and Flowers | Oxalobacteraceae | Massilia |
| ASV_35 | 0.50 | -1.79 | -1.60 | 0.44 | 0.30 | 0.29 | 1.13 | -5.94 | -5.57 | 0.27 | 0.00 | 0.00 | 0.43 | 0.00 | 0.00 | Leaves and Flowers | Rhizobiaceae | Candidatus Liberibacter |
| ASV_36 | -4.57 | 1.04 | 3.28 | 0.39 | 0.29 | 0.29 | -11.87 | 3.57 | 11.39 | 0.00 | 0.00 | 0.00 | 0.00 | 0.00 | 0.00 | Leaves and Flowers | Pseudomonadaceae | Pseudomonas |
| ASV_37 | 0.44 | -1.24 | -1.59 | 0.39 | 0.29 | 0.29 | 1.11 | -4.28 | -5.55 | 0.28 | 0.00 | 0.00 | 0.43 | 0.00 | 0.00 | Leaves and Flowers | Comamonadaceae | Unclassified |
| ASV_385 | NA | NA | NA | NA | NA | NA | NA | NA | NA | 1.00 | 1.00 | 1.00 | 1.00 | 1.00 | 1.00 | Not different | Unclassified | Unclassified |
| ASV_39 | -0.03 | -0.02 | -0.39 | 0.39 | 0.28 | 0.27 | -0.07 | -0.07 | -1.40 | 0.94 | 0.95 | 0.18 | 1.00 | 1.00 | 0.24 | Not different | Burkholderiaceae | Unclassified |
| ASV_391 | -0.85 | 0.70 | 0.02 | 0.37 | 0.27 | 0.26 | -2.29 | 2.59 | 0.09 | 0.03 | 0.02 | 0.93 | 0.08 | 0.04 | 0.99 | Leaves only | Propionibacteriaceae | Cutibacterium |
| ASV_393 | 0.32 | 0.20 | -0.73 | 0.41 | 0.30 | 0.29 | 0.80 | 0.65 | -2.55 | 0.43 | 0.52 | 0.01 | 0.60 | 0.63 | 0.03 | Flowers only | Rhizobiaceae | Allorhizobium. |
| ASV_4 | -1.17 | 3.20 | 1.11 | 0.43 | 0.31 | 0.38 | -2.74 | 10.16 | 2.93 | 0.01 | 0.00 | 0.00 | 0.03 | 0.00 | 0.01 | Leaves and Flowers | Burkholderiaceae | Ralstonia |
| ASV_404 | -0.11 | -0.82 | -1.30 | 0.39 | 0.29 | 0.28 | -0.28 | -2.87 | -4.61 | 0.78 | 0.01 | 0.00 | 0.93 | 0.02 | 0.00 | Leaves and Flowers | Rhizobiaceae | Allorhizobium. |
| ASV_405 | -0.03 | -0.28 | -0.16 | 0.37 | 0.26 | 0.28 | -0.08 | -1.07 | -0.56 | 0.94 | 0.29 | 0.58 | 1.00 | 0.41 | 0.68 | Not different | Unclassified | Unclassified |
| ASV_406 | 0.61 | 0.04 | -0.70 | 0.38 | 0.28 | 0.26 | 1.60 | 0.16 | -2.67 | 0.12 | 0.87 | 0.01 | 0.23 | 0.96 | 0.03 | Flowers only | Caulobacteraceae | Brevundimonas |
| ASV_41 | -2.83 | 0.73 | 2.41 | 0.40 | 0.35 | 0.32 | -7.08 | 2.12 | 7.50 | 0.00 | 0.04 | 0.00 | 0.00 | 0.07 | 0.00 | Flowers only | Pseudomonadaceae | Pseudomonas |
| ASV_42 | -3.84 | 1.58 | 2.49 | 0.38 | 0.37 | 0.33 | -10.18 | 4.28 | 7.57 | 0.00 | 0.00 | 0.00 | 0.00 | 0.00 | 0.00 | Leaves and Flowers | Pseudomonadaceae | Pseudomonas |
| ASV_428 | -0.94 | 0.20 | 1.75 | 0.37 | 0.27 | 0.29 | -2.55 | 0.75 | 6.10 | 0.02 | 0.46 | 0.00 | 0.05 | 0.59 | 0.00 | Flowers only | Unclassified | Unclassified |
| ASV_436 | -1.25 | 0.04 | 1.38 | 0.40 | 0.28 | 0.29 | -3.15 | 0.14 | 4.75 | 0.00 | 0.89 | 0.00 | 0.02 | 0.97 | 0.00 | Flowers only | Flavobacteriaceae | Unclassified |
| ASV_44 | -0.44 | 0.35 | 0.91 | 0.43 | 0.38 | 0.31 | -1.03 | 0.93 | 2.92 | 0.31 | 0.36 | 0.00 | 0.46 | 0.48 | 0.01 | Flowers only | Pseudomonadaceae | Pseudomonas |
| ASV_46 | -1.35 | 1.41 | 1.53 | 0.37 | 0.27 | 0.30 | -3.63 | 5.31 | 5.13 | 0.00 | 0.00 | 0.00 | 0.00 | 0.00 | 0.00 | Leaves and Flowers | Oxalobacteraceae | Duganella |
| ASV_47 | -0.88 | 1.44 | 1.47 | 0.36 | 0.26 | 0.27 | -2.45 | 5.47 | 5.54 | 0.02 | 0.00 | 0.00 | 0.05 | 0.00 | 0.00 | Leaves and Flowers | Oxalobacteraceae | Unclassified |
| ASV_477 | 0.33 | -1.84 | -0.54 | 0.38 | 0.28 | 0.28 | 0.88 | -6.67 | -1.93 | 0.39 | 0.00 | 0.07 | 0.57 | 0.00 | 0.12 | Leaves only | Microbacteriaceae | Microbacterium |
| ASV_487 | -0.64 | 1.48 | 0.32 | 0.39 | 0.27 | 0.27 | -1.66 | 5.47 | 1.16 | 0.12 | 0.00 | 0.27 | 0.22 | 0.00 | 0.34 | Leaves only | Oxalobacteraceae | Unclassified |
| ASV_49 | -1.22 | 0.42 | 1.73 | 0.40 | 0.36 | 0.33 | -3.05 | 1.18 | 5.26 | 0.00 | 0.24 | 0.00 | 0.01 | 0.35 | 0.00 | Flowers only | Pseudomonadaceae | Pseudomonas |
| ASV_5 | -1.03 | 0.35 | 1.58 | 0.36 | 0.34 | 0.30 | -2.81 | 1.04 | 5.24 | 0.01 | 0.30 | 0.00 | 0.02 | 0.41 | 0.00 | Flowers only | Pseudomonadaceae | Pseudomonas |
| ASV_50 | -2.53 | 0.23 | 2.11 | 0.38 | 0.30 | 0.29 | -6.73 | 0.78 | 7.29 | 0.00 | 0.44 | 0.00 | 0.00 | 0.56 | 0.00 | Flowers only | Pseudomonadaceae | Pseudomonas |
| ASV_51 | 1.44 | -0.52 | -1.66 | 0.39 | 0.30 | 0.30 | 3.71 | -1.76 | -5.46 | 0.00 | 0.08 | 0.00 | 0.00 | 0.14 | 0.00 | Flowers only | Rhizobiaceae | Allorhizobium. |
| ASV_52 | -1.03 | -0.64 | 1.14 | 0.36 | 0.27 | 0.29 | -2.85 | -2.39 | 3.88 | 0.01 | 0.02 | 0.00 | 0.02 | 0.04 | 0.00 | Leaves and Flowers | Pseudomonadaceae | Pseudomonas |
| ASV_523 | -0.41 | 0.51 | 0.38 | 0.38 | 0.26 | 0.28 | -1.09 | 1.94 | 1.38 | 0.29 | 0.07 | 0.18 | 0.45 | 0.12 | 0.25 | Not different | Erwiniaceae | Pantoea |
| ASV_536 | -0.92 | 0.92 | -0.50 | 0.39 | 0.27 | 0.28 | -2.36 | 3.34 | -1.78 | 0.03 | 0.00 | 0.09 | 0.08 | 0.01 | 0.14 | Leaves only | Microbacteriaceae | Curtobacterium |
| ASV_54 | 0.59 | 0.14 | -0.19 | 0.42 | 0.34 | 0.31 | 1.40 | 0.40 | -0.63 | 0.17 | 0.69 | 0.53 | 0.29 | 0.79 | 0.64 | Not different | Oxalobacteraceae | Unclassified |
| ASV_543 | -0.27 | -1.03 | 0.29 | 0.39 | 0.29 | 0.28 | -0.68 | -3.53 | 1.05 | 0.51 | 0.00 | 0.31 | 0.69 | 0.01 | 0.40 | Leaves only | Oxalobacteraceae | Duganella |
| ASV_55 | -0.53 | 0.58 | 0.59 | 0.41 | 0.33 | 0.34 | -1.28 | 1.76 | 1.72 | 0.21 | 0.09 | 0.09 | 0.34 | 0.15 | 0.14 | Not different | Unclassified | Unclassified |
| ASV_553 | -0.89 | 0.48 | 0.79 | 0.38 | 0.27 | 0.27 | -2.35 | 1.76 | 2.97 | 0.04 | 0.10 | 0.01 | 0.09 | 0.17 | 0.02 | Flowers only | Pseudomonadaceae | Pseudomonas |
| ASV_56 | NA | NA | NA | NA | NA | NA | NA | NA | NA | 1.00 | 1.00 | 1.00 | 1.00 | 1.00 | 1.00 | Not different | Unclassified | Unclassified |
| ASV_57 | -1.94 | 0.54 | 2.91 | 0.45 | 0.40 | 0.35 | -4.29 | 1.35 | 8.40 | 0.00 | 0.18 | 0.00 | 0.00 | 0.27 | 0.00 | Flowers only | Pseudomonadaceae | Pseudomonas |
| ASV_58 | -6.67 | 0.45 | 3.04 | 0.37 | 0.28 | 0.28 | -18.03 | 1.61 | 10.87 | 0.00 | 0.12 | 0.00 | 0.00 | 0.19 | 0.00 | Flowers only | Pseudomonadaceae | Pseudomonas |
| ASV_59 | -5.17 | 0.95 | 3.19 | 0.37 | 0.30 | 0.29 | -13.99 | 3.19 | 10.85 | 0.00 | 0.00 | 0.00 | 0.00 | 0.01 | 0.00 | Leaves and Flowers | Pseudomonadaceae | Pseudomonas |
| ASV_595 | -0.89 | 0.27 | -0.56 | 0.38 | 0.27 | 0.28 | -2.33 | 0.99 | -2.01 | 0.05 | 0.35 | 0.08 | 0.10 | 0.47 | 0.12 | Not different | Oxalobacteraceae | Unclassified |
| ASV_6 | -0.34 | 0.19 | 1.99 | 0.36 | 0.33 | 0.33 | -0.96 | 0.57 | 6.04 | 0.34 | 0.57 | 0.00 | 0.50 | 0.67 | 0.00 | Flowers only | Pseudomonadaceae | Pseudomonas |
| ASV_60 | 0.14 | 1.87 | 1.66 | 0.39 | 0.31 | 0.32 | 0.35 | 6.13 | 5.13 | 0.73 | 0.00 | 0.00 | 0.87 | 0.00 | 0.00 | Leaves and Flowers | Erwiniaceae | Erwinia |
| ASV_61 | -2.74 | 0.70 | 2.26 | 0.35 | 0.30 | 0.29 | -7.72 | 2.30 | 7.87 | 0.00 | 0.02 | 0.00 | 0.00 | 0.05 | 0.00 | Leaves and Flowers | Pseudomonadaceae | Pseudomonas |
| ASV_62 | -1.31 | 1.23 | 0.93 | 0.37 | 0.27 | 0.27 | -3.51 | 4.49 | 3.48 | 0.00 | 0.00 | 0.00 | 0.01 | 0.00 | 0.00 | Leaves and Flowers | Oxalobacteraceae | Duganella |
| ASV_63 | -4.17 | 0.45 | 2.70 | 0.36 | 0.27 | 0.29 | -11.66 | 1.67 | 9.41 | 0.00 | 0.10 | 0.00 | 0.00 | 0.17 | 0.00 | Flowers only | Pseudomonadaceae | Pseudomonas |
| ASV_65 | -1.23 | 2.14 | 0.75 | 0.36 | 0.28 | 0.26 | -3.40 | 7.51 | 2.87 | 0.00 | 0.00 | 0.01 | 0.01 | 0.00 | 0.01 | Leaves and Flowers | Burkholderiaceae | Burkholderia. |
| ASV_67 | 0.54 | -0.40 | -1.21 | 0.36 | 0.28 | 0.27 | 1.48 | -1.40 | -4.56 | 0.15 | 0.17 | 0.00 | 0.27 | 0.26 | 0.00 | Flowers only | Rhodanobacteraceae | Luteibacter |
| ASV_675 | -0.88 | -0.19 | -0.28 | 0.40 | 0.28 | 0.28 | -2.19 | -0.69 | -1.00 | 0.05 | 0.50 | 0.33 | 0.10 | 0.61 | 0.42 | Not different | Flavobacteriaceae | Flavobacterium |

| | | | | | | | | | | | | | | | | | | |
|---|---|---|---|---|---|---|---|---|---|---|---|---|---|---|---|---|---|---|
| ASV_69 | 1.01 | -1.00 | -2.15 | 0.39 | 0.30 | 0.29 | 2.57 | -3.33 | -7.31 | 0.01 | 0.00 | 0.00 | 0.04 | 0.00 | 0.00 | Leaves and Flowers | Microbacteriaceae | Galbitalea |
| ASV_7 | -1.00 | -0.12 | 1.97 | 0.37 | 0.33 | 0.28 | -2.69 | -0.38 | 6.93 | 0.01 | 0.71 | 0.00 | 0.03 | 0.80 | 0.00 | Flowers only | Pseudomonadaceae | Pseudomonas |
| ASV_71 | -0.82 | 1.19 | 0.13 | 0.36 | 0.27 | 0.25 | -2.28 | 4.46 | 0.52 | 0.03 | 0.00 | 0.61 | 0.08 | 0.00 | 0.71 | Leaves only | Oxalobacteraceae | Unclassified |
| ASV_710 | -0.31 | 0.43 | 0.36 | 0.37 | 0.27 | 0.29 | -0.84 | 1.57 | 1.25 | 0.41 | 0.12 | 0.22 | 0.58 | 0.19 | 0.29 | Not different | Oxalobacteraceae | Janthinobacterium |
| ASV_75 | -0.95 | 1.69 | 0.11 | 0.36 | 0.27 | 0.26 | -2.63 | 6.23 | 0.41 | 0.01 | 0.00 | 0.68 | 0.04 | 0.00 | 0.77 | Leaves only | Unclassified | Unclassified |
| ASV_79 | -0.72 | 0.86 | 0.51 | 0.37 | 0.28 | 0.28 | -1.94 | 3.07 | 1.84 | 0.06 | 0.00 | 0.07 | 0.12 | 0.01 | 0.12 | Leaves only | Oxalobacteraceae | Janthinobacterium |
| ASV_8 | 0.00 | 0.26 | 1.36 | 0.38 | 0.33 | 0.33 | 0.01 | 0.80 | 4.09 | 0.99 | 0.43 | 0.00 | 1.00 | 0.56 | 0.00 | Flowers only | Pseudomonadaceae | Pseudomonas |
| ASV_80 | -0.83 | -0.56 | 0.79 | 0.37 | 0.27 | 0.28 | -2.25 | -2.05 | 2.80 | 0.03 | 0.05 | 0.01 | 0.08 | 0.09 | 0.02 | Flowers only | Pseudomonadaceae | Pseudomonas |
| ASV_83 | 0.59 | 0.13 | 0.42 | 0.41 | 0.30 | 0.30 | 1.45 | 0.45 | 1.39 | 0.16 | 0.66 | 0.17 | 0.27 | 0.77 | 0.24 | Not different | Oxalobacteraceae | Unclassified |
| ASV_84 | 0.79 | -1.20 | -1.71 | 0.40 | 0.31 | 0.31 | 1.95 | -3.87 | -5.47 | 0.06 | 0.00 | 0.00 | 0.12 | 0.00 | 0.00 | Leaves and Flowers | Kineosporiaceae | Kineosporia |
| ASV_85 | -0.34 | 0.73 | 0.52 | 0.37 | 0.28 | 0.27 | -0.92 | 2.63 | 1.91 | 0.36 | 0.01 | 0.06 | 0.53 | 0.03 | 0.10 | Leaves only | Oxalobacteraceae | Unclassified |
| ASV_861 | -0.31 | 0.47 | -0.07 | 0.38 | 0.27 | 0.26 | -0.82 | 1.73 | -0.28 | 0.43 | 0.11 | 0.78 | 0.60 | 0.18 | 0.86 | Not different | Oxalobacteraceae | Unclassified |
| ASV_879 | -0.98 | 1.31 | -0.51 | 0.39 | 0.28 | 0.28 | -2.52 | 4.72 | -1.83 | 0.03 | 0.00 | 0.10 | 0.08 | 0.00 | 0.15 | Leaves only | Micrococcaceae | Arthrobacter |
| ASV_9 | 0.92 | -0.62 | -1.00 | 0.42 | 0.36 | 0.33 | 2.18 | -1.71 | -3.05 | 0.03 | 0.09 | 0.00 | 0.08 | 0.15 | 0.01 | Flowers only | Rhizobiaceae | Allorhizobium. |
| ASV_92 | -0.55 | 0.95 | 1.37 | 0.38 | 0.29 | 0.33 | -1.45 | 3.27 | 4.21 | 0.15 | 0.00 | 0.00 | 0.27 | 0.01 | 0.00 | Leaves and Flowers | Unclassified | Unclassified |
| ASV_93 | 0.53 | -0.43 | -0.21 | 0.42 | 0.36 | 0.30 | 1.28 | -1.19 | -0.72 | 0.21 | 0.24 | 0.48 | 0.34 | 0.35 | 0.58 | Not different | Flavobacteriaceae | Flavobacterium |
| ASV_950 | 0.10 | 1.17 | 0.37 | 0.38 | 0.27 | 0.26 | 0.25 | 4.40 | 1.40 | 0.80 | 0.00 | 0.19 | 0.93 | 0.00 | 0.26 | Leaves only | Mycobacteriaceae | Mycobacterium |
| ASV_96 | -3.66 | 0.99 | 2.37 | 0.37 | 0.28 | 0.28 | -9.96 | 3.50 | 8.40 | 0.00 | 0.00 | 0.00 | 0.00 | 0.00 | 0.00 | Leaves and Flowers | Pseudomonadaceae | Pseudomonas |
| ASV_97 | -0.90 | 1.55 | 1.37 | 0.37 | 0.28 | 0.26 | -2.43 | 5.46 | 5.21 | 0.02 | 0.00 | 0.00 | 0.06 | 0.00 | 0.00 | Leaves and Flowers | Oxalobacteraceae | Massilia |
| ASV_99 | -0.17 | 1.46 | 0.48 | 0.39 | 0.33 | 0.33 | -0.43 | 4.42 | 1.45 | 0.67 | 0.00 | 0.16 | 0.81 | 0.00 | 0.22 | Leaves only | Yersiniaceae | Rahnella1 |

**Table S13.** Summary of network properties of endophytic bacterial co-occurrence networks of alpine Rosaceae plants. (**A**) Networks for each tissue (flower, leaf, and root) and (**B**) network comparison between them (flower vs root, flower vs leaf, and leaf vs root) are reported. Hub nodes were identified based on eigenvector centrality values. The Jaccard index was used for assessing how different the sets of most central nodes are between the two networks (0 if the sets are completely different and 1 for exactly equal sets).

## A

### Flower network

Component sizes

size: 128
#: 1

Global network properties

Whole network:

| | |
|---|---|
| Number of components | 1 |
| Clustering coefficient | 0.19297 |
| Modularity | 0.55942 |
| Positive edge percentage | 79.6238 |
| Edge density | 0.03925 |
| Natural connectivity | 0.01057 |
| Vertex connectivity | 1 |
| Edge connectivity | 1 |
| Average dissimilarity* | 0.987 |
| Average path distance** | 2.39021 |

-----

*: Dissimilarity = 1 - edge weight

**: Path distance = Units with average dissimilarity

Clusters
- In the whole network
- Algorithm: cluster_fast_greedy

name: 1 2 3 4 5 6 7 8 9 10
#: 30 21 19 7 4 7 11 6 10 13

Hubs
- In alphabetical/numerical order
- Based on empirical quantiles of centralities

ASV_1
ASV_102
ASV_11
ASV_36
ASV_58
ASV_59
ASV_63

### Leaf network

Component sizes

size: 104 1
#: 1 4

Global network properties

Largest connected component (LCC):

| | |
|---|---|
| Relative LCC size | 0.96296 |
| Clustering coefficient | 0.18975 |
| Modularity | 0.60229 |
| Positive edge percentage | 75.13228 |
| Edge density | 0.03529 |
| Natural connectivity | 0.01214 |
| Vertex connectivity | 1 |
| Edge connectivity | 1 |
| Average dissimilarity* | 0.98857 |
| Average path distance** | 2.82114 |

Whole network:

| | |
|---|---|
| Number of components | 5 |
| Clustering coefficient | 0.18975 |
| Modularity | 0.60229 |
| Positive edge percentage | 75.13228 |
| Edge density | 0.03271 |
| Natural connectivity | 0.01161 |

-----

*: Dissimilarity = 1 - edge weight

**: Path distance = Units with average dissimilarity

Clusters
- In the whole network
- Algorithm: cluster_fast_greedy

name: 0 1 2 3 4 5 6 7 8
#: 4 12 23 13 21 10 6 17 2

Hubs
- In alphabetical/numerical order
- Based on empirical quantiles of centralities

ASV_10
ASV_18
ASV_2
ASV_38
ASV_4
ASV_7

### Root network

Component sizes

size: 273 1
#: 1 1

Global network properties

Largest connected component (LCC):

| | |
|---|---|
| Relative LCC size | 0.99635 |
| Clustering coefficient | 0.12387 |
| Modularity | 0.45535 |
| Positive edge percentage | 73.5471 |
| Edge density | 0.02688 |
| Natural connectivity | 0.00523 |
| Vertex connectivity | 1 |
| Edge connectivity | 1 |
| Average dissimilarity* | 0.99172 |
| Average path distance** | 2.26361 |

Whole network:

| | |
|---|---|
| Number of components | 2 |
| Clustering coefficient | 0.12387 |
| Modularity | 0.45535 |
| Positive edge percentage | 73.5471 |
| Edge density | 0.02668 |
| Natural connectivity | 0.0052 |

-----

*: Dissimilarity = 1 - edge weight

**: Path distance = Units with average dissimilarity

Clusters
- In the whole network
- Algorithm: cluster_fast_greedy

name: 0 1 2 3 4 5 6 7 8
#: 1 21 30 54 22 62 74 8 2

Hubs
- In alphabetical/numerical order
- Based on empirical quantiles of centralities

ASV_109
ASV_160
ASV_200
ASV_206
ASV_277
ASV_280
ASV_314
ASV_363
ASV_39
ASV_407
ASV_504
ASV_514
ASV_560
ASV_684

Centrality measures
- In decreasing order
- Computed for the complete network
``````````````````````````````````````````

Degree (normalized):

| | |
|---|---|
| ASV_145 | 0.09449 |
| ASV_58 | 0.09449 |
| ASV_1 | 0.08661 |
| ASV_102 | 0.08661 |
| ASV_34 | 0.08661 |
| ASV_59 | 0.07874 |
| ASV_11 | 0.07087 |
| ASV_36 | 0.07087 |
| ASV_42 | 0.07087 |
| ASV_63 | 0.07087 |

Betweenness centrality (normalized):

| | |
|---|---|
| ASV_145 | 0.13748 |
| ASV_58 | 0.09336 |
| ASV_1 | 0.07787 |
| ASV_34 | 0.07637 |
| ASV_225 | 0.06474 |
| ASV_14 | 0.06349 |
| ASV_22 | 0.06074 |
| ASV_325 | 0.05699 |
| ASV_147 | 0.05124 |
| ASV_59 | 0.05087 |

Closeness centrality (normalized):

| | |
|---|---|
| ASV_1 | 0.63145 |
| ASV_58 | 0.6201 |
| ASV_145 | 0.61711 |
| ASV_102 | 0.59977 |
| ASV_34 | 0.59665 |
| ASV_14 | 0.59619 |
| ASV_36 | 0.59524 |
| ASV_11 | 0.58783 |
| ASV_63 | 0.58201 |
| ASV_59 | 0.58109 |

Eigenvector centrality (normalized):

| | |
|---|---|
| ASV_58 | 1 |
| ASV_1 | 0.80757 |
| ASV_59 | 0.76521 |
| ASV_36 | 0.70757 |
| ASV_102 | 0.62688 |
| ASV_63 | 0.60194 |
| ASV_11 | 0.58977 |
| ASV_118 | 0.57014 |
| ASV_29 | 0.52907 |
| ASV_96 | 0.51392 |

Centrality measures
- In decreasing order
- Centrality of disconnected components is zero
``````````````````````````````````````````````````````````

Degree (normalized):

| | |
|---|---|
| ASV_4 | 0.1028 |
| ASV_18 | 0.08411 |
| ASV_2 | 0.08411 |
| ASV_7 | 0.08411 |
| ASV_75 | 0.08411 |
| ASV_24 | 0.07477 |
| ASV_17 | 0.06542 |
| ASV_21 | 0.06542 |
| ASV_36 | 0.06542 |
| ASV_10 | 0.05607 |

Betweenness centrality (normalized):

| | |
|---|---|
| ASV_4 | 0.23053 |
| ASV_18 | 0.13649 |
| ASV_2 | 0.12926 |
| ASV_8 | 0.1167 |
| ASV_7 | 0.11631 |
| ASV_62 | 0.1087 |
| ASV_145 | 0.10261 |
| ASV_75 | 0.09556 |
| ASV_24 | 0.08757 |
| ASV_27 | 0.08338 |

Closeness centrality (normalized):

| | |
|---|---|
| ASV_4 | 0.62181 |
| ASV_18 | 0.5981 |
| ASV_7 | 0.58533 |
| ASV_2 | 0.57609 |
| ASV_38 | 0.55536 |
| ASV_10 | 0.54805 |
| ASV_27 | 0.54323 |
| ASV_17 | 0.54094 |
| ASV_8 | 0.54094 |
| ASV_75 | 0.53602 |

Eigenvector centrality (normalized):

| | |
|---|---|
| ASV_18 | 1 |
| ASV_4 | 0.96263 |
| ASV_7 | 0.94067 |
| ASV_2 | 0.91416 |
| ASV_38 | 0.74368 |
| ASV_10 | 0.73488 |
| ASV_11 | 0.70635 |
| ASV_17 | 0.61272 |
| ASV_20 | 0.60195 |
| ASV_14 | 0.5983 |

Centrality measures
- In decreasing order
- Centrality of disconnected components is zero
``````````````````````````````````````````````````````````

Degree (normalized):

| | |
|---|---|
| ASV_560 | 0.07692 |
| ASV_407 | 0.07326 |
| ASV_109 | 0.0696 |
| ASV_370 | 0.0696 |
| ASV_200 | 0.06593 |
| ASV_284 | 0.06227 |
| ASV_314 | 0.06227 |
| ASV_363 | 0.06227 |
| ASV_451 | 0.06227 |
| ASV_120 | 0.05861 |

Betweenness centrality (normalized):

| | |
|---|---|
| ASV_109 | 0.04366 |
| ASV_370 | 0.04005 |
| ASV_257 | 0.03864 |
| ASV_284 | 0.03788 |
| ASV_2 | 0.03769 |
| ASV_407 | 0.03633 |
| ASV_560 | 0.03419 |
| ASV_200 | 0.03112 |
| ASV_451 | 0.02898 |
| ASV_37 | 0.02778 |

Closeness centrality (normalized):

| | |
|---|---|
| ASV_109 | 0.63511 |
| ASV_407 | 0.63451 |
| ASV_200 | 0.61499 |
| ASV_560 | 0.61319 |
| ASV_363 | 0.60936 |
| ASV_684 | 0.60096 |
| ASV_370 | 0.60019 |
| ASV_160 | 0.59932 |
| ASV_314 | 0.5977 |
| ASV_284 | 0.59482 |

Eigenvector centrality (normalized):

| | |
|---|---|
| ASV_407 | 1 |
| ASV_560 | 0.89941 |
| ASV_200 | 0.8097 |
| ASV_314 | 0.7715 |
| ASV_206 | 0.75889 |
| ASV_109 | 0.75037 |
| ASV_684 | 0.72239 |
| ASV_280 | 0.72091 |
| ASV_39 | 0.69692 |
| ASV_504 | 0.64367 |

## B

### Flower vs leaf network

Comparison of Network Properties
----------------------------------
Global network properties
``````````````````````````

Whole network:

| | Flowers | Leaves | difference |
|---|---|---|---|
| Number of components | 1 | 3 | 2 |
| Clustering coefficient | 0.2 | 0.209 | 0 |
| Modularity | 0.541 | 0.583 | 0 |
| Positive edge percentage | 81.081 | 76.606 | 4 |
| Edge density | 0.042 | 0.036 | 0 |
| Natural connectivity | 0.012 | 0.012 | 0 |
| Vertex connectivity | 1 | 1 | 0 |

### Flower vs root network

Comparison of Network Properties
----------------------------------
Global network properties
``````````````````````````

Whole network:

| | Flowers | Roots | difference |
|---|---|---|---|
| Number of componer | 1 | 2 | 1 |
| Clustering coefficient | 0.244 | 0.195 | 0.049 |
| Modularity | 0.589 | 0.509 | 0.079 |
| Positive edge percen | 88.592 | 71.503 | 17.09 |
| Edge density | 0.032 | 0.031 | 0.002 |
| Natural connectivity | 0.009 | 0.008 | 0 |
| Vertex connectivity | 1 | 1 | 0 |
``````````````````````````

| | | | |
|---|---|---|---|
| Edge connectivity | 1 | 1 | 0 |
| Average dissimilarity* | 0.986 | 0.988 | 0 |
| Average path distance** | 2.427 | 2.838 | 0 |

-----
*: Dissimilarity = 1 - edge weight
**: Path distance = Units with average dissimilarity

Jaccard index (similarity betw. sets of most central nodes)

| | Jacc | P(<=Jacc) | P(>=Jacc) |
|---|---|---|---|
| degree | 0.2 | 0.063388 . | 0.972691 |
| betweenness centr. | 0.098 | 0.000096*** | 0.999981 |
| closeness centr. | 0.302 | 0.400476 | 0.719168 |
| eigenvec. centr. | 0.6 | 0.999668 | 0.001091** |
| hub taxa | 0.333 | 0.650307 | 0.622822 |

-----
Jaccard index in [0,1] (1 indicates perfect agreement)

Adjusted Rand index (similarity betw. clusterings)

| | wholeNet | LCC |
|---|---|---|
| ARI | 0.253 | 0.253 |
| p-value | 0.000 | 0.000 |

-----
ARI in [-1,1] with ARI=1: perfect agreement betw. clusterings
ARI=0: expected for two random clusterings
p-value: permutation test (n=1000) with null hypothesis ARI=0

Graphlet Correlation Distance

| | wholeNet | LCC |
|---|---|---|
| GCD | 0.617 | 0.602 |

-----
GCD >= 0 (GCD=0 indicates perfect agreement between GCMs)

Centrality measures
- In decreasing order
- Computed for the whole network

Degree (normalized):

| | Flowers | Leaves | abs.diff. |
|---|---|---|---|
| ASV_102 | 0.081 | 0.009 | 0 |
| ASV_4 | 0.027 | 0.09 | 0 |
| ASV_21 | 0.018 | 0.072 | 0 |
| ASV_444 | 0.081 | 0.027 | 0 |
| ASV_59 | 0.09 | 0.036 | 0 |
| ASV_1 | 0.081 | 0.036 | 0 |
| ASV_47 | 0.054 | 0.009 | 0 |
| ASV_75 | 0.027 | 0.072 | 0 |
| ASV_65 | 0.072 | 0.027 | 0 |
| ASV_2 | 0.045 | 0.081 | 0 |

Betweenness centrality (normalized):

| | Flowers | Leaves | abs.diff. |
|---|---|---|---|
| ASV_4 | 0.012 | 0.155 | 0 |
| ASV_38 | 0.006 | 0.107 | 0 |
| ASV_2 | 0.002 | 0.099 | 0 |
| ASV_308 | 0.004 | 0.085 | 0 |
| ASV_21 | 0.002 | 0.083 | 0 |
| ASV_58 | 0.071 | 0.141 | 0 |
| ASV_14 | 0.093 | 0.024 | 0 |
| ASV_62 | 0.029 | 0.095 | 0 |
| ASV_20 | 0.019 | 0.08 | 0 |
| ASV_444 | 0.058 | 0 | 0 |

Closeness centrality (normalized):

| | Flowers | Leaves | abs.diff. |
|---|---|---|---|
| ASV_125 | 0.508 | 0 | 1 |
| ASV_312 | 0.451 | 0 | 0 |
| ASV_102 | 0.586 | 0.306 | 0 |
| ASV_47 | 0.525 | 0.276 | 0 |
| ASV_106 | 0.538 | 0.306 | 0 |

| | | | |
|---|---|---|---|
| Edge connectivity | 1 | 1 | 0 |
| Average dissimilarity* | 0.989 | 0.99 | 0.001 |
| Average path distanc | 2.65 | 9 2.672 | 0.013 |

-----
*: Dissimilarity = 1 - edge weight
**: Path distance = Units with average dissimilarity

Jaccard index (similarity betw. sets of most central nodes)

| | Jacc | P(<=Jacc) | | P(>=Jacc) |
|---|---|---|---|---|
| degree | 0.152 | 0.000737 | *** | 0.99976 |
| betweenness centr. | 0.067 | 0 | *** | 1 |
| closeness centr. | 0.194 | 0.008832 | ** | 0.9961 |
| eigenvec. centr. | 0.194 | 0.008832 | ** | 0.9961 |
| hub taxa | 0 | 0.001522 | ** | 1 |

-----
Jaccard index in [0,1] (1 indicates perfect agreement)

Adjusted Rand index (similarity betw. clusterings)

| | wholeNet | LCC |
|---|---|---|
| ARI | 0.156 | 0.156 |
| p-value | 0.000 | 0.000 |

-----
ARI in [-1,1] with ARI=1: perfect agreement betw. clusterings
ARI=0: expected for two random clusterings
p-value: permutation test (n=1000) with null hypothesis ARI=0

Graphlet Correlation Distance

| | wholeNet | LCC |
|---|---|---|
| GCD | 0.854 | 0.844 |

-----
GCD >= 0 (GCD=0 indicates perfect agreement between GCMs)

Centrality measures
- In decreasing order
- Computed for the whole network

Degree (normalized):

| | Flowers | Roots | abs.diff. |
|---|---|---|---|
| ASV_109 | 0.05 | 0.164 | 0.113 |
| ASV_238 | 0.082 | 0.006 | 0.075 |
| ASV_1635 | 0.082 | 0.013 | 0.069 |
| ASV_338 | 0.019 | 0.082 | 0.063 |
| ASV_226 | 0.094 | 0.038 | 0.057 |
| ASV_139 | 0.019 | 0.069 | 0.05 |
| ASV_210 | 0.069 | 0.019 | 0.05 |
| ASV_331 | 0.057 | 0.006 | 0.05 |
| ASV_20 | 0.025 | 0.075 | 0.05 |
| ASV_370 | 0.025 | 0.075 | 0.05 |

Betweenness centrality (normalized):

| | Flowers | Roots | abs.diff. |
|---|---|---|---|
| ASV_109 | 0.024 | 0.153 | 0.13 |
| ASV_139 | 0 | 0.086 | 0.086 |
| ASV_226 | 0.097 | 0.013 | 0.084 |
| ASV_20 | 0.017 | 0.098 | 0.082 |
| ASV_399 | 0.083 | 0.004 | 0.079 |
| ASV_406 | 0.074 | 0.004 | 0.07 |
| ASV_257 | 0.004 | 0.07 | 0.066 |
| ASV_59 | 0.082 | 0.018 | 0.064 |
| ASV_3 | 0.021 | 0.084 | 0.064 |
| ASV_130 | 0.002 | 0.059 | 0.057 |

Closeness centrality (normalized):

| | Flowers | Roots | abs.diff. |
|---|---|---|---|
| ASV_478 | 0.5 | 0 | 0.5 |
| ASV_238 | 0.547 | 0.249 | 0.298 |
| ASV_1635 | 0.568 | 0.304 | 0.263 |
| ASV_97 | 0.506 | 0.312 | 0.194 |
| ASV_85 | 0.503 | 0.312 | 0.191 |

| | | | | | | | |
|---|---|---|---|---|---|---|---|
| ASV_148 | 0.518 | 0.322 | 0 | ASV_338 | 0.406 | 0.593 | 0.187 |
| ASV_104 | 0.575 | 0.387 | 0 | ASV_139 | 0.359 | 0.542 | 0.182 |
| ASV_940 | 0.417 | 0.239 | 0 | ASV_82 | 0.518 | 0.35 | 0.168 |
| ASV_205 | 0.479 | 0.325 | 0 | ASV_109 | 0.51 | 0.675 | 0.165 |
| ASV_441 | 0.465 | 0.317 | 0 | ASV_218 | 0.499 | 0.336 | 0.163 |

Significance codes: ***: 0.001, **: 0.01, *: 0.05, .: 0.1

Significance codes: ***: 0.001, **: 0.01, *: 0.05, .: 0.1

| Eigenve | ctor cent | rality (n | ormalized): |
|---|---|---|---|
| | group '' | group '' | abs.diff. |
| ASV_102 | 0.566 | 0.005 | 1 |
| ASV_59 | 0.884 | 0.358 | 1 |
| ASV_21 | 0.063 | 0.53 | 0 |
| ASV_1 | 0.787 | 0.364 | 0 |
| ASV_4 | 0.292 | 0.67 | 0 |
| ASV_106 | 0.371 | 0.005 | 0 |
| ASV_14 | 0.673 | 0.341 | 0 |
| ASV_2 | 0.469 | 0.792 | 0 |
| ASV_104 | 0.425 | 0.113 | 0 |
| ASV_38 | 0.163 | 0.475 | 0 |

Significance codes: ***: 0.001, **: 0.01, *: 0.05,

Eigenvector centrality (normalized):

| | group '' | group '' | abs.diff. |
|---|---|---|---|
| ASV_1635 | 1 | 0.001 | 0.999 |
| ASV_238 | 0.847 | 0 | 0.847 |
| ASV_109 | 0.235 | 1 | 0.765 |
| ASV_199 | 0.911 | 0.221 | 0.69 |
| ASV_39 | 0.041 | 0.564 | 0.523 |
| ASV_226 | 0.585 | 0.076 | 0.509 |
| ASV_115 | 0.025 | 0.516 | 0.491 |
| ASV_229 | 0.497 | 0.023 | 0.474 |
| ASV_609 | 0.476 | 0.01 | 0.466 |
| ASV_270 | 0.117 | 0.536 | 0.419 |

Significance codes: ***: 0.001, **: 0.01, *: 0.05, .: 0.1

## Leaf vs root network

Comparison of Network Properties
----------------------------------
Global network properties
````````````````````````

Largest connected component (LCC):

| | Leaves | Roots | difference |
|---|---|---|---|
| Relative LCC size | 0.973 | 0.987 | 0 |
| Clustering coefficient | 0.294 | 0.189 | 0 |
| Modularity | 0.559 | 0.536 | 0 |
| Positive edge percentage | 88 | 70.69 | 17 |
| Edge density | 0.035 | 0.032 | 0 |
| Natural connectivity | 0.011 | 0.009 | 0 |
| Vertex connectivity | 1 | 1 | 0 |
| Edge connectivity | 1 | 1 | 0 |
| Average dissimilarity* | 0.989 | 0.99 | 0 |
| Average path distance** | 2.79 | 7 2.722 | 0 |

Whole network:

| | Leaves | Roots | difference |
|---|---|---|---|
| Number of components | 5 | 3 | 2 |
| Clustering coefficient | 0.294 | 0.189 | 0 |
| Modularity | 0.559 | 0.536 | 0 |
| Positive edge percentage | 88 | 70.69 | 17 |
| Edge density | 0.034 | 0.031 | 0 |
| Natural connectivity | 0.01 | 0.009 | 0 |

-----
*: Dissimilarity = 1 - edge weight
**: Path distance = Units with average dissimilarity

Jaccard index (similarity betw. sets of most central nodes)
```````````````````````````````````````````````````````

| | Jacc | P(<=Jacc) | | P(>=Jacc) |
|---|---|---|---|---|
| degree | 0.177 | 0.004969 | ** | 0.998031 |
| betweenness centr. | 0.194 | 0.011343 | * | 0.995031 |
| closeness centr. | 0.152 | 0.000737 | *** | 0.999755 |
| eigenvec. centr. | 0.267 | 0.169197 | | 0.89289 |
| hub taxa | 0 | 0.001522 | ** | 1 |

-----
Jaccard index in [0,1] (1 indicates perfect agreement)

Adjusted Rand index (similarity betw. clusterings)
``````````````````````````````````````````````

| | wholeNet | LCC |
|---|---|---|
| ARI | 0.123 | 0.123 |
| p-value | 0 | 0 |

-----
ARI in [-1,1] with ARI=1: perfect agreement betw. clusterings
ARI=0: expected for two random clusterings
p-value: permutation test (n=1000) with null hypothesis ARI=0
``````````````````````````````````````````````

Graphlet Correlation Distance
````````````````````````````

| | wholeNet | LCC |
|---|---|---|
| GCD | 0.95 | 1.008 |

-----
GCD >= 0 (GCD=0 indicates perfect agreement between GCMs)

Centrality measures
- In decreasing order
- Centrality of disconnected components is zero
`````````````````````````````````````````````

Degree (normalized):

| | Leaves | Roots | abs.diff. |
|---|---|---|---|
| ASV_238 | 0.188 | 0.013 | 0 |
| ASV_609 | 0.181 | 0.007 | 0 |
| ASV_367 | 0.181 | 0.02 | 0 |
| ASV_585 | 0.181 | 0.02 | 0 |
| ASV_316 | 0.181 | 0.027 | 0 |
| ASV_152 | 0.034 | 0.134 | 0 |
| ASV_363 | 0.007 | 0.094 | 0 |
| ASV_32 | 0.027 | 0.107 | 0 |
| ASV_51 | 0.02 | 0.087 | 0 |
| ASV_1635 | 0.074 | 0.007 | 0 |

Betweenness centrality (normalized):

| | Leaves | Roots | abs.diff. |
|---|---|---|---|
| ASV_238 | 0.292 | 0.001 | 0 |
| ASV_1635 | 0.166 | 0 | 0 |
| ASV_20 | 0.008 | 0.113 | 0 |
| ASV_75 | 0.109 | 0.013 | 0 |
| ASV_145 | 0.016 | 0.109 | 0 |
| ASV_7 | 0.096 | 0.003 | 0 |
| ASV_152 | 0.025 | 0.113 | 0 |
| ASV_51 | 0.013 | 0.1 | 0 |
| ASV_32 | 0.019 | 0.104 | 0 |
| ASV_169 | 0.089 | 0.005 | 0 |

Closeness centrality (normalized):

| | Leaves | Roots | abs.diff. |
|---|---|---|---|
| ASV_47 | 0 | 0.504 | 1 |
| ASV_62 | 0.478 | 0 | 0 |
| ASV_234 | 0 | 0.47 | 0 |
| ASV_940 | 0 | 0.408 | 0 |
| ASV_478 | 0.383 | 0 | 0 |
| ASV_125 | 0 | 0.37 | 0 |
| ASV_609 | 0.669 | 0.331 | 0 |
| ASV_238 | 0.703 | 0.386 | 0 |
| ASV_285 | 0.233 | 0.533 | 0 |
| ASV_1635 | 0.593 | 0.299 | 0 |

Significance codes: ***: 0.001, **: 0.01, *: 0.05,

Eigenvector centrality (normalized):

| | group '' | group '' | abs.diff. |
|---|---|---|---|
| ASV_152 | 0.001 | 1 | 1 |
| ASV_238 | 1 | 0.039 | 1 |
| ASV_609 | 0.85 | 0.004 | 1 |
| ASV_367 | 0.843 | 0.063 | 1 |
| ASV_32 | 0.005 | 0.738 | 1 |
| ASV_363 | 0.005 | 0.737 | 1 |
| ASV_585 | 0.842 | 0.14 | 1 |
| ASV_316 | 0.881 | 0.189 | 1 |
| ASV_51 | 0.004 | 0.645 | 1 |
| ASV_115 | 0.003 | 0.622 | 1 |

Significance codes: ***: 0.001, **: 0.01, *: 0.05,

**Table S15.** Analysis of variance (ANOVA) of linear models (LMs) on the abundance of culturable psychrotolerant bacterial endophytes for the first dataset (**A**; *Alchemilla* sp. and *G. montanum* from six collection sites) and second dataset (**B**; *Alchemilla* sp., *D. octopetala,* and *G. montanum* from two collection sites) of samples from alpine Rosaceae plants. Results of the model performance to select the most appropriate model (i.e., model with either fixed effect or with interactions having the lowest root mean squared error (RMSE)) for the analysis are shown in the top. A post-hoc analysis with estimated marginal mean (EMM) comparisons was carried-out to better highlight differences between levels in each factor (alpine Rosaceae plant, plant tissue, collection site, and exposure) ($P \leq 0.05$).

## A

**Summary of model performance**

| | | | |
|---|---|---|---|
| LM with fixed effects: | RMSE | R squared | MAE |
| | 0.8441426 | 0.4284579 | 0.657992 |
| LM with interactions: | RMSE | R squared | MAE |
| | 0.7749192 | 0.5715198 | 0.56681 |

**LM on bacterial abundance**

ANOVA of LM based on fixed effects + interactions.

| Analysis of Variance Table | **Df** | **Sum Sq** | **Mean Sq** | **F value** | **Pr(>F)** | **Significance** |
|---|---|---|---|---|---|---|
| alpine_Rosaceae_plant | 1 | 3.823 | 3.823 | 10.31 | 0.0016 | ** |
| Tissue | 2 | 76.235 | 38.118 | 102.80 | 0.0000 | *** |
| Collection site | 5 | 38.407 | 7.681 | 20.72 | 0.0000 | *** |
| Exposition | 1 | 0.399 | 0.399 | 1.08 | 0.3010 | NS |
| alpine_Rosaceae_plant:Tissue | 2 | 8.026 | 4.013 | 10.82 | 0.0000 | *** |
| alpine_Rosaceae_plant:Collection site | 5 | 9.561 | 1.912 | 5.16 | 0.0002 | *** |
| Tissue:Collection site | 10 | 41.323 | 4.132 | 11.14 | 0.0000 | *** |
| alpine_Rosaceae_plant:Exposition | 1 | 0.48 | 0.48 | 1.29 | 0.2571 | NS |
| Tissue:Exposition | 2 | 1.006 | 0.503 | 1.36 | 0.2610 | NS |
| Collection_site:Exposition | 5 | 3.324 | 0.665 | 1.79 | 0.1179 | NS |
| alpine_Rosaceae_plant:Tissue:Collection_site | 10 | 14.325 | 1.432 | 3.86 | 0.0001 | *** |
| alpine_Rosaceae_plant:Tissue:Exposition | 2 | 2.063 | 1.031 | 2.78 | 0.0653 | NS |
| alpine_Rosaceae_plant:Collection_site:Exposition | 5 | 1.735 | 0.347 | 0.94 | 0.4598 | NS |
| Tissue:Collection_site:Exposition | 10 | 5.908 | 0.591 | 1.59 | 0.1141 | NS |
| alpine_Rosaceae_plant:Tissue:Collection_site:Exposition | 10 | 4.465 | 0.447 | 1.20 | 0.2930 | NS |
| Residuals | 144 | 53.395 | 0.371 | | | |

**Significance**

| ***p*** **value** | **Code** |
|---|---|
| > 0.05 | not significative (NS) |
| < 0.05 | * |
| < 0.01 | ** |
| < 0.001 | *** |

**Pairwise comparisons of abundance values**

| **alpine Rosaceae plant** | **emmean** | **SE** | **df** | **lower.CL** | **upper.CL** | **.group** |
|---|---|---|---|---|---|---|
| *Geum montanum* | 4.05 | 0.0586 | 144 | 3.92 | 4.19 | a |
| *Alchemilla* sp. | 4.32 | 0.0586 | 144 | 4.19 | 4.45 | b |

Results are averaged over the levels of: Tissue, Collection site, Exposure
Confidence level used: 0.95
Conf-level adjustment: bonferroni method for 2 estimates
significance level used: alpha = 0.05
NOTE: If two or more means share the same grouping symbol,
then we cannot show them to be different.
But we also did not show them to be the same.

| **Tissue** | **emmean** | **SE** | **df** | **lower.CL** | **upper.CL** | **.group** |
|---|---|---|---|---|---|---|
| Leaves | 3.35 | 0.0718 | 144 | 3.17 | 3.52 | a |
| Roots | 4.6 | 0.0718 | 144 | 4.42 | 4.77 | b |
| Flower | 4.61 | 0.0718 | 144 | 4.44 | 4.79 | b |

Results are averaged over the levels of: alpine Rosaceae plant, Collection site, Exposure
Confidence level used: 0.95
Conf-level adjustment: bonferroni method for 3 estimates
P value adjustment: fdr method for 3 tests
significance level used: alpha = 0.05
NOTE: If two or more means share the same grouping symbol,
then we cannot show them to be different.

But we also did not show them to be the same.

| Collection site | emmean | SE | df | lower.CL | upper.CL | .group |
|---|---|---|---|---|---|---|
| Site B | 3.65 | 0.101 | 144 | 3.38 | 3.92 | a |
| Site A | 3.84 | 0.101 | 144 | 3.57 | 4.11 | ab |
| Site C | 4.06 | 0.101 | 144 | 3.79 | 4.33 | bc |
| Site F | 4.18 | 0.101 | 144 | 3.91 | 4.46 | cd |
| Site D | 4.42 | 0.101 | 144 | 4.15 | 4.69 | d |
| Site G | 4.96 | 0.101 | 144 | 4.68 | 5.23 | e |

Results are averaged over the levels of: alpine Rosaceae plant, Tissue, Exposure
Confidence level used: 0.95
Conf-level adjustment: bonferroni method for 6 estimates
P value adjustment: fdr method for 15 tests
significance level used: alpha = 0.05
NOTE: If two or more means share the same grouping symbol,
then we cannot show them to be different.
But we also did not show them to be the same.

| Exposure | emmean | SE | df | lower.CL | upper.CL | .group |
|---|---|---|---|---|---|---|
| North | 4.14 | 0.0586 | 144 | 4.01 | 4.28 | a |
| South | 4.23 | 0.0586 | 144 | 4.1 | 4.36 | a |

Results are averaged over the levels of: alpine Rosaceae plant, Tissue, Collection site
Confidence level used: 0.95
Conf-level adjustment: bonferroni method for 2 estimates
significance level used: alpha = 0.05
NOTE: If two or more means share the same grouping symbol,
then we cannot show them to be different.
But we also did not show them to be the same.

## B

**Summary of model performance**

| | | | |
|---|---|---|---|
| LM with fixed effects: | RMSE | R squared | MAE |
| | 0.5937808 | 0.5846177 | 0.445659 |
| LM with interactions: | RMSE | R squared | MAE |
| | 0.6440968 | 0.5812054 | 0.452345 |

**LM on bacterial abundance**

ANOVA of LM based on fixed effects.

| Analysis of Variance Table | Df | Sum Sq | Mean Sq | F value | Pr(>F) | Significance |
|---|---|---|---|---|---|---|
| alpine_Rosaceae_plant | 2 | 0.215 | 0.1074 | 0.30 | 0.7426 | NS |
| Tissue | 2 | 38.708 | 19.3542 | 53.80 | <0.00000000( | *** |
| Collection site | 1 | 10.247 | 10.2467 | 28.48 | 5.825E-07 | *** |
| Exposition | 1 | 0.177 | 0.1767 | 0.49 | 0.4851 | NS |
| Residuals | 101 | 36.336 | 0.3598 | | | |

**Significance**

| ***p* value** | **Code** |
|---|---|
| > 0.05 | not significative (NS) |
| < 0.05 | * |
| < 0.01 | ** |
| < 0.001 | *** |

**Pairwise comparisons of abundance values**

| alpine Rosaceae plant | emmean | SE | df | lower.CL | .group |
|---|---|---|---|---|---|
| *Alchemilla* sp. | 4.64 | 0.1 | 101 | 4.4 | a |
| *Geum montanum* | 4.74 | 0.1 | 101 | 4.49 | a |
| *Dryas octopetala* | 4.74 | 0.1 | 101 | 4.5 | a |

Results are averaged over the levels of: Tissue, Collection site, Exposure
Confidence level used: 0.95
Conf-level adjustment: bonferroni method for 3 estimates
P value adjustment: fdr method for 3 tests
significance level used: alpha = 0.05
NOTE: If two or more means share the same grouping symbol,
then we cannot show them to be different.
But we also did not show them to be the same.

| Tissue | emmean | SE | df | lower.CL | .group |
|---|---|---|---|---|---|
| Leaves | 3.87 | 0.1 | 101 | 3.62 | a |
| Roots | 5.04 | 0.1 | 101 | 4.8 | b |

| | | | | | |
|---|---|---|---|---|---|
| Flower | 5.21 | 0.1 | 101 | 4.97 | b |

Results are averaged over the levels of: alpine Rosaceae plant, Collection site, Exposure
Confidence level used: 0.95
Conf-level adjustment: bonferroni method for 3 estimates
P value adjustment: fdr method for 3 tests
significance level used: alpha = 0.05
NOTE: If two or more means share the same grouping symbol,
then we cannot show them to be different.
But we also did not show them to be the same.

| **Collection site** | **emmean** | **SE** | **df** | **lower.CL** | **.group** |
|---|---|---|---|---|---|
| Site D | 4.4 | 0.0816 | 101 | 4.21 | a |
| Site G | 5.01 | 0.0816 | 101 | 4.83 | b |

Results are averaged over the levels of: alpine Rosaceae plant, Tissue, Exposure
Confidence level used: 0.95
Conf-level adjustment: bonferroni method for 2 estimates
significance level used: alpha = 0.05
NOTE: If two or more means share the same grouping symbol,
then we cannot show them to be different.
But we also did not show them to be the same.

| **Exposure** | **emmean** | **SE** | **df** | **lower.CL** | **.group** |
|---|---|---|---|---|---|
| South | 4.67 | 0.0816 | 101 | 4.48 | a |
| North | 4.75 | 0.0816 | 101 | 4.56 | a |

Results are averaged over the levels of: alpine Rosaceae plant, Tissue, Collection site
Confidence level used: 0.95
Conf-level adjustment: bonferroni method for 2 estimates
significance level used: alpha = 0.05
NOTE: If two or more means share the same grouping symbol,
then we cannot show them to be different.
But we also did not show them to be the same.

**Table S17.** PCR programs and conditions used for (**A**) 16S rRNA gene amplicon sequencing and (**B**) Sanger sequencing.

## A

First PCR
Flowers

| Component | Volume (µl) | Concentration |
|---|---|---|
| $H_2O$ Sterile | 17.875 | |
| DMSO | 1.000 | 0.01 g/ml |
| BSA | 0.250 | |
| dNTPs | 0.375 | 2.5 mM |
| Reaction Buffer with 18 mM MgCl2 | 2.500 | |
| Primer Fw | 0.750 | 10 µM |
| Primer Rev | 0.750 | 10 µM |
| DNA | 1.000 | |
| Enzyme Blend | 0.500 | 5 U/µl |
| VOLUME | 25.00 | |

| THERMAL PROFILE PCR | | | |
|---|---|---|---|
| 95°C | x | 5' | |
| 95°C | x | 30'' | X36 |
| 52°C | x | 30'' | |
| 72°C | x | 1' | |
| 72°C | x | 5' | |
| 8°C | x | ∞ | |

First PCR
Leaves and roots

| Component | Volume (µl) | Concentration |
|---|---|---|
| $H_2O$ Sterile | 15.375 | |
| DMSO | 1.000 | 0.01 g/ml |
| BSA | 0.250 | |
| dNTPs | 0.375 | 2.5 mM |
| Reaction Buffer without MgCl2 | 2.500 | |
| MgCl2 | 2.500 | 25 mM |
| Primer Fw | 0.750 | 10 µM |
| Primer Rev | 0.750 | 10 µM |
| DNA | 1.000 | |
| Enzyme Blend | 0.500 | 5 U/µl |
| VOLUME | 25.00 | |

| THERMAL PROFILE PCR | | | |
|---|---|---|---|
| 95°C | x | 5' | |
| 95°C | x | 30'' | X40 |
| 52°C | x | 30'' | |
| 72°C | x | 1' | |
| 72°C | x | 5' | |
| 8°C | x | ∞ | |

Second PCR
All tissues

| Componente | Volume (µl) | Concentration |
|---|---|---|
| $H_2O$ Sterile | 17.750 | |
| DMSO | 1.000 | 0.01 g/ml |
| BSA | 0.250 | |
| dNTPs | 0.250 | 2.5 mM |
| Reaction Buffer with 18 mM MgCl2 | 2.500 | |
| Primer Fw | 0.500 | 10 µM |
| Primer Rev | 0.500 | 10 µM |
| DNA | 2.000 | |
| Enzyme Blend | 0.250 | 5 U/µl |
| VOLUME | 25.00 | |

| THERMAL PROFILE PCR | | | |
|---|---|---|---|
| 95°C | x | 5' | |
| 95°C | x | 30'' | X36 |
| 52°C | x | 30'' | |
| 72°C | x | 1' | |
| 72°C | x | 5' | |
| 8°C | x | ∞ | |

## B

| Component | Volume (µl) | Concentration |
|---|---|---|
| $H_2O$ Sterile | 19.60 | |
| PCR Buffer | 2.50 | |
| dNTPs | 0.20 | |
| Primer Fw | 0.60 | 10 µM |
| Primer Rev | 0.60 | 10 µM |
| DNA | 1.00 | |
| DreamTaq | 0.50 | |
| VOLUME | 25.00 | |

| THERMAL PROFILE PCR | | | |
|---|---|---|---|
| 95°C | x | 5' | |
| 95°C | x | 30'' | X30 |
| 55°C | x | 30'' | |
| 72°C | x | 1' | |
| 72°C | x | 10' | |
| 8°C | x | ∞ | |
